# Dynamic transcriptomic profiles of zebrafish gills in response to zinc depletion

**DOI:** 10.1186/1471-2164-11-548

**Published:** 2010-10-08

**Authors:** Dongling Zheng, Peter Kille, Graham P Feeney, Phil Cunningham, Richard D Handy, Christer Hogstrand

**Affiliations:** 1 Mineral Metabolism Group, Nutritional Sciences Division, King's College London, London SE1 9NH, UK; 2 School of Biosciences, University of Cardiff, Cardiff, CF10 3TL, UK; 3 School of Biological Sciences, University of Plymouth, Plymouth, PL4 8AA, UK; 4 Current Address: Division of Infection and Immunity, Windeyer Institute of Medical Sciences, University College London, London W1T 4JF

## Abstract

**Background:**

Zinc deficiency is detrimental to organisms, highlighting its role as an essential micronutrient contributing to numerous biological processes. To investigate the underlying molecular events invoked by zinc depletion we performed a temporal analysis of transcriptome changes observed within the zebrafish gill. This tissue represents a model system for studying ion absorption across polarised epithelial cells as it provides a major pathway for fish to acquire zinc directly from water whilst sharing a conserved zinc transporting system with mammals.

**Results:**

Zebrafish were treated with either zinc-depleted (water = 2.61 μg L^-1^; diet = 26 mg kg^-1^) or zinc-adequate (water = 16.3 μg L^-1^; diet = 233 mg kg^-1^) conditions for two weeks. Gill samples were collected at five time points and transcriptome changes analysed in quintuplicate using a 16K oligonucleotide array. Of the genes represented the expression of a total of 333 transcripts showed differential regulation by zinc depletion (having a fold-change greater than 1.8 and an adjusted P-value less than 0.1, controlling for a 10% False Discovery Rate). Down-regulation was dominant at most time points and distinct sets of genes were regulated at different stages. Annotation enrichment analysis revealed that 'Developmental Process' was the most significantly overrepresented Biological Process GO term (*P *= 0.0006), involving 26% of all regulated genes. There was also significant bias for annotations relating to development, cell cycle, cell differentiation, gene regulation, butanoate metabolism, lysine degradation, protein tyrosin phosphatases, nucleobase, nucleoside and nucleotide metabolism, and cellular metabolic processes. Within these groupings genes associated with diabetes, bone/cartilage development, and ionocyte proliferation were especially notable. Network analysis of the temporal expression profile indicated that transcription factors *foxl1, wt1, nr5a1, nr6a1*, and especially, *hnf4a *may be key coordinators of the homeostatic response to zinc depletion.

**Conclusions:**

The study revealed the complex regulatory pathways that allow the organism to subtly respond to the low-zinc condition. Many of the processes affected reflected a fundamental restructuring of the gill epithelium through reactivation of developmental programs leading to stem cell differentiation. The specific regulation of genes known to be involved in development of diabetes provides new molecular links between zinc deficiency and this disease. The present study demonstrates the importance of including the time-dimension in microarray studies.

## Background

Zinc is an essential trace element for all organisms, and is involved in a variety of biological functions [1-3]. It has been recognised as a cofactor for more than 300 catalytic enzymes, and is required for structural and functional integrity of more than 2000 transcription factors involved in the expression of various genes [3,4]. Therefore, almost every signalling and metabolic pathway is in some way dependent on at least one, and often several, zinc-requiring proteins. In humans zinc deficiency has become a world-wide problem and causes a variety of symptoms including retarded growth, diarrhoea, anorexia, impaired immunity, skin lesions, and abnormal development [5]. Zinc has also been implicated in many diseases including immune system defects [6], neurodegeneration [7], diabetes [8], and cancer [9]. Therefore, elucidation of the molecular targets of zinc becomes exceedingly important.

Intracellular accumulation of zinc is the outcome of influx and efflux processes via zinc transporter proteins. These are mainly from the Slc39 (ZIP) transporter family, which transports zinc into the cytosol, and the Slc30 (ZnT) transporter family, responsible for the flux of zinc away from the cytosol, either into organelles or out of the cell [10]. In addition, metallothioneins (MTs) have high binding affinity for zinc and play a very important role in maintaining stable intracellular zinc availability through the binding or releasing zinc [11]. Levels of MT proteins depend on zinc availability and are, at least partially, regulated at the mRNA level. A well-known mechanism is through the metal-responsive transcription factor 1 (Mtf1), a recognised zinc-sensory transcriptional activator. Upon binding to zinc Mtf1 is able to bind metal-response elements (MREs), and further induces transcription of key target genes such as metallothioneins (MTs) and zinc transporter-1 Slc30a1 (*Znt1*) [12,13]. Mtf1 may also function as a transcriptional repressor as exemplified by its down-regulation of *zip10 *[14,15]. Mtf1-regulated genes also include several genes not related to zinc in an obvious way [14,16,17].

Changes in mRNA expression patterns due to zinc deficiency in some mammalian cells and tissues, such as intestine, liver, hepatocytes and leukocytes, have been investigated using microarray technology [18-23]. Some genes identified in these studies are involved in growth and energy metabolism, hepatic lipid metabolism, and signal transduction pathways that control immune responses. These data have provided a starting point to investigate molecular mechanisms mediating zinc deficiency-derived metabolic disturbances. However, although distinct sets of genes appear to be regulated by zinc deficiency, the response seems to vary from tissue to tissue, between cell types, and treatment conditions. We argue that zinc regulation is not only tissue-specific but also time-dependent, such that groups of genes with distinct functionalities may be regulated at different time-points following a change in zinc availability. In addition, as zinc is mainly obtained from diet in mammals it takes time to reach a state of zinc deficiency. It can also be difficult to distinguish the effect of zinc deficiency from other disturbances associated with poor nutrition.

In addition to absorbing zinc from the diet, fish take up zinc across the gills directly from the water. It is evident from previous studies that zinc regulation in the fish gill is indeed highly dynamic and efficient [15,24,25] and that zinc transporters in fish play similar functions as in mammals [26-29]. With the genomic resources available for zebrafish this is an experimental organism of choice for molecular studies on zinc. The aims of this study were to further elucidate the effects of zinc deficiency and underlying regulation mechanisms, particularly the temporal components of gene expression. Our approach therefore employs a comprehensive microarray (in-house 16K oligonucleotide arrays) to explore the time course of transcription profiles in the zebrafish gill. Zebrafish were maintained under both normal and zinc depleted conditions, and transcriptomic analysis phenotypically anchored by physiological measurements of whole body nutrient composition, zinc status and uptake. Transcriptional profiles from gill samples collected at five time points were obtained and analysed in quintuplicate. Expression of a total of 333 transcripts showed differential regulation by zinc deficiency. These were enriched with genes involved in biological processes such as development, cell cycle, cell differentiation, gene regulation, butanoate metabolism, lysine degradation, protein dephosphorylation, nucleobase, nucleoside and nucleotide metabolism, and cellular metabolic processes. DNA-binding proteins was one of the largest molecular function groups among regulated genes, and bioinformatic analysis of the temporal gene expression profile indicated that transcription factors *foxl1*, *wt1*, *nr5a1*, *nr6a1*, and especially, *hnf4a *may be key coordinators of the homeostatic response to zinc depletion.

## Results

### Physiological characterisation of zinc treatment

**Figure 1:**
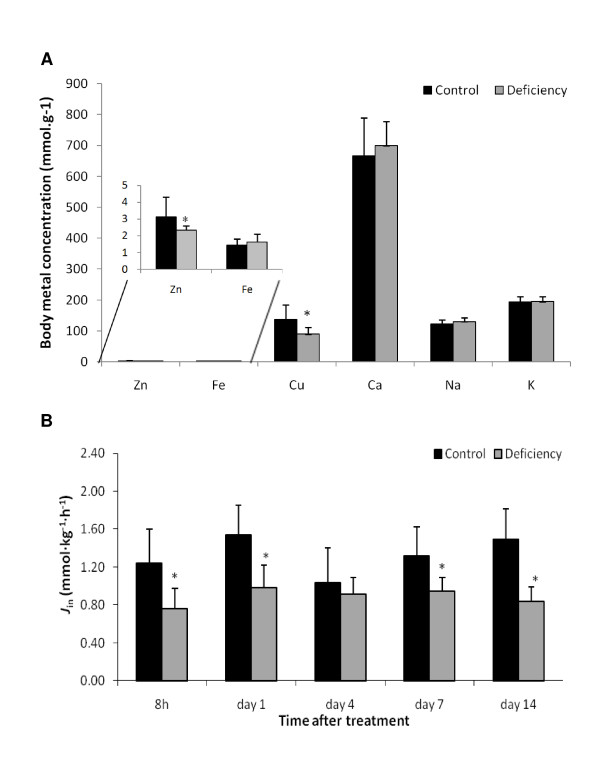
**Physiological changes after zinc depletion**. (A) Elemental composition of whole body (minus gill) determined by ICP-MS. (B) Unidirectional Zn^2+ ^influx across the gills. Zebrafish were treated with zinc depleted feed and water for 14 days and compared with fish kept at the standard control condition. The bars represent the mean of nine fish for elemental composition or eight fish for Zn^2+ ^influx analysis; error bars denote standard deviation (SD). A statistical significance between control and zinc deficiency is shown by an asterisk (*P *< 0.05).

To elucidate the underlying molecular effects of zinc deficiency we conducted a time-course experiment on zebrafish subjected for 14 days to either zinc-depleted water and low zinc diet, or zinc-adequate water and diet as a control. The efficacy of the zinc depleted conditions in producing zinc deficiency was demonstrated by a 25% reduction in body zinc content (excluding gill) (Figure 1A). It was recently shown that the same treatment also resulted in persistently decreased levels of zinc in gills [15]. The body copper content was also significantly reduced, while the body iron content showed a non-significant tendency to increase (*P *= 0.16). This indicates the occurrence of interplay between regulation of zinc and other metals. There was no significant change observed in body weight, lipid and protein content between the two groups at the end of two-week experiment (data not shown).

The fish gill is a major pathway for zinc acquisition directly from water. To test the effect of zinc depletion on the zinc up-take capacity across the gill, unidirectional zinc influx was measured at an external zinc concentration of 5 μM Zn^2+ ^for eight fish from each group at each time point. Fish on the zinc-deficiency treatment showed a 30-40% lower zinc uptake compared to the normal controls at all time points except Day 4 (Figure 1B).

### Validation of microarray method by quantitative PCR

The qPCR of mRNA abundance for 10 genes, using nine biological replicates (gills from 9 fish) per group per time point including the five gill samples used for microarrays, verified the accuracy and validity of our array results. Considering the large number of samples (90 in this study and 135 in combination with zinc supplementation), validation was only applied to several genes regulated on Days 1 and 7, except *mt2 *(Dr.121290) and *slc30a1 *(*znt1 *- Dr.12303) which were extended to cover all time points. The data were combined with those from genes selected in a parallel study [30] and shown in Table 1. The measurements from qPCR analysis were in agreement, at least in direction, with the microarray results. It was, therefore, concluded that quantification of transcripts by microarrays provided a good representation of the relative expression of the genes in zebrafish gills.

**Table 1:** Primers used in qPCR validation and the result from qPCR and microarray measurements.

		Primers		**Depletion**^**b**^			
		
**Gene ID (symbol)**^**a**^	Time point	Forward (5'-3')	Reverse (5'-3')	Array	qPCR	Array	qPCR
Dr.134300 (ppara)	24 h	GAACGCAACTGCAAGATCCAG	CCAAAACGAATAGCGTTGTGG	1.21 ± 0.5	0.99 ± 0.2	1.98 ± 0.2	1.52 ± 0.1
Dr.15390 (foxl1)	24 h	TGGTGTTTGGCATCCTCGAC	CTTGAAAGCGGTGTGTGAGTG	2.42 ± 1.0	0.97 ± 0.2	1.47 ± 0.4	0.84 ± 0.1
Dr.77183 (hmgcs1)	24 h	GTCACTCAAGATGGCACACCTG	CTCTGAGCTTCATTGTCTCAGC	1.36 ± 0.1	1.31 ± 0.4	1.57 ± 0.8	1.46 ± 0.8
Dr.76570 (apob)	24 h	TCAGCAATGCTATTCGCATGAG	CACTTATCCCATCACTCCGAGC	1.36 ± 0.5	1.04 ± 0.8	2.25 ± 0.6	2.06 ± 1.0
Dr.80336 (cyp11a1)	7d	GCAGCCCTGAAAGAAACTCTCA	TCCCTGCTGGAATGTGGTAATT	0.72 ± 0.4	2.72 ± 1.0	0.53 ± 0.2	0.41 ± 0.1
Dr.429 (dlx5)	7d	ACGCGAGCTCTTCGTGGTATT	CAGTACAACGTTCCTGATCCGA	0.38 ± 0.1	0.56 ± 0.2	0.56 ± 0.3	0.96 ± 0.5
Dr.2 (dlx2)	7d	GCCTCACGCAAACACAGGTT	AGACTCACCGGAGGCCACAT	1.08 ± 0.6	1.20 ± 0.5	1.87 ± 0.6	2.28 ± 1.2
Dr.78697 (rfx2)	14d	CCGGAGATCATCAGCACTAAGG	GGTCGCTGAGCATCTGATTGA	1.26 ± 0.8	0.75 ± 0.1	2.07 ± 0.3	2.99 ± 1.5
Dr.121290 (mt)	8 h	AATGGACCCCTGCGAAT	GGTAGCACCACAGTTGCAA	0.32 ± 0.3	0.30 ± 0.1	1.91 ± 1.5	1.87 ± 1.2
	24 h			0.21 ± 0.2	0.81 ± 0.4	3.67 ± 0.6	2.72 ± 0.9
	4d			0.53 ± 0.2	0.44 ± 0.2	4.90 ± 1.7	4.91 ± 2.9
	7d			0.15 ± 0.1	0.38 ± 0.1	7.48 ± 1.6	5.65 ± 1.4
	14d			0.91 ± 0.7	0.28 ± 0.1	13.82 ± 3.3	12.52 ± 4.4
Dr.12303 (znt1)	8 h	AGTGCCCGAGCAGATCGA	GCTAGAACTCCATCCAGGCTCTT	0.74 ± 0.2	0.82 ± 0.1	1.11 ± 0.0	1.30 ± 0.4
	24 h			0.60 ± 0.4	0.94 ± 0.6	1.42 ± 0.4	1.11 ± 0.4
	4d			0.89 ± 0.0	0.78 ± 0.2	0.88 ± 0.2	1.22 ± 0.2
	7d			0.98 ± 0.0	0.55 ± 0.2	2.01 ± 0.6	1.57 ± 0.4
	14d			0.92 ± 0.3	0.96 ± 0.4	1.35 ± 0.6	2.71 ± 0.9
18s		CGGAGGTTCGAAGACGATCA	CGGGTCGGCATCGTTTAC				

^a^Gene IDs are provided as UnigeneID and ZNC gene symbols

^b^Expression levels are expressed as mean ± SD of fish treated with zinc depletion or supplementation compared to control as analysed by the two methods for the durations indicated

^c^Supplementation was conducted as a parallel experiment [30].

### General effect of zinc depletion on differential gene expression in gills

Five fish were withdrawn from either the zinc depleted or adequate conditions at each of five time points: 8 hours, 1, 4, 7, and 14 days. Gills were dissected and RNA extracted from each pair of gills. A total of 16,331 gene targets on the zebrafish arrays were evaluated for a total of 49 RNA samples (25 from the zinc depleted fish and 24 from the controls (one control sample from Day 4 was removed because of failure to pass quality control, *N *= 4 or 5).

A total of 12,095 probe sets showed significant hybridisation signals (73%), which covered approximately 9,610 genes with Unigene IDs. Genes that showed a fold-change greater than 1.8 and an adjusted P-value less than 0.1, controlling for a 10% false discovery rate (FDR) with the Benjamini-Hochberg method, were regarded as differentially regulated by zinc depletion compared to the control group. We found that a total of 376 reporters, 3.1% of all significant signals in gill, were significantly regulated at one or more time points. Of these, 335 transcripts corresponding to 330 Unigene IDs were analysed further (Additional file 1, Table S1). Treating each time point as a separate event 36.6% of all significant changes in gene expression represented up-regulation and 63.4% down-regulation.

**Figure 2:**
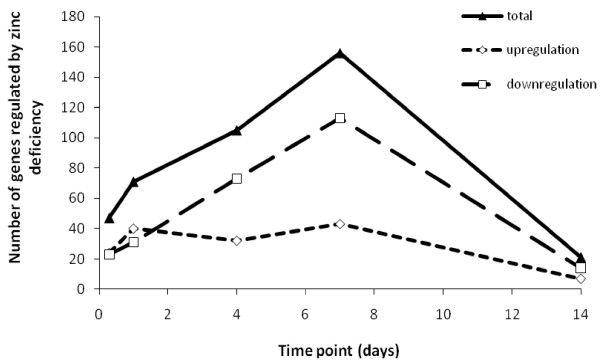
**Time-course of numbers of genes called regulated upon zinc depletion**. The triangles and solid line show the total number of regulated genes (>1.8-fold change; FDR = 0.1; *N *= 4 or 5) at each time-point, diamonds and short-dashed line give the numbers of up-regulated genes, and the numbers of down-regulated genes are indicated by rectangles and long-dash line.

A temporal profile of the overall numbers of up- or down-regulated transcripts showed that changes in gene expression occurred as early as 8 hours following introduction of the zinc depleted condition, reached the peak level at Day 7, and then subsided by Day 14 (Figure 2). At the first two sampling points, 8 and 24 hours, the numbers of genes that were up- or down-regulated were similar. Down-regulation dominated for the remainder of the 14-day period with the number of genes down-regulated compared to the control being three times higher than those up-regulated. These results suggest that zinc deficiency has a negative influence on the overall gene expression level in gill and that a new steady-state in zinc homeostasis is established within 14 days. There was little continuity in regulation of individual genes, in fact, *mt2 *was the only gene consistently and significantly regulated in a single direction (down-regulated) throughout the experiment. Many other genes were significantly up-regulated relative to the control at one time point and down-regulated at another.

### Functional classification of the regulated genes

The functional enrichment analysis was performed using the functional annotation tools of DAVID (the Database for Annotation, Visualisation and Integrated Discovery [31]) to determine if zinc deficiency resulted in regulation of genes of any particular type or function. The zebrafish genome is still relatively sparsely represented in the DAVID database so that when a combined list of all 330 regulated genes with unique Unigene IDs was submitted only 75 were returned with annotations. Categories that were overrepresented among these 75 genes included protein dephosphorylation and embryonic development (*P *< 0.05, Table 2). Another group of interesting genes (although they did not cluster on DAVID) consisted of three major histocompatibility complex genes, *zgc:103700 *(a MHCII protein), *mhc1ufa *and *mhc1uda*. These genes were significantly regulated on Days 4 and 14, suggesting that antigen presentation might be affected by zinc deficiency.

**Table 2:** Annotation enrichment among regulated genes at all time-points using zebrafish Unigene gene identifiers.

Annotation type	**Category Name**^**a**^	Genes	P-value
INTERPRO	Protein-tyrosine phosphatase, Tyr-specific/dual-specificity type	3	2.90E-03
GOTERM_BP_ALL	cartilage development	3	1.00E-02
GOTERM_BP_ALL	tissue development	4	1.10E-02
GOTERM_BP_ALL	cell migration involved in gastrulation	3	1.60E-02
GOTERM_BP_ALL	anatomical structure development	8	1.80E-02
GOTERM_BP_ALL	multicellular organismal development	9	2.10E-02
GOTERM_BP_ALL	skeletal development	3	2.40E-02
GOTERM_BP_ALL	gastrulation	3	3.60E-02
GOTERM_MF_ALL	methyltransferase activity	3	4.00E-02
GOTERM_MF_ALL	transferase activity, transferring one-carbon groups	3	4.10E-02
GOTERM_MF_ALL	phosphoprotein phosphatase activity	3	4.10E-02
GOTERM_BP_ALL	protein amino acid dephosphorylation	3	4.20E-02

^a^Categories enriched in the data-set at *P *< 0.05 and containing three or more genes are listed.

To investigate functional systems and pathways regulated by zinc deficiency in more details we took advantage of the similarities between human and zebrafish genes and used the zebrafish Unigene clusters associated with our reporters to retrieve the corresponding human orthologs. Of the 330 regulated genes with unique Unigene IDs 245 human orthologs were matched (74%, Additional file 1, Table S1) and submitted to DAVID. A total of 227 entries were returned with annotations. Genes regulated at each time point were also classified and analysed in the same manner.

**Figure 3:**
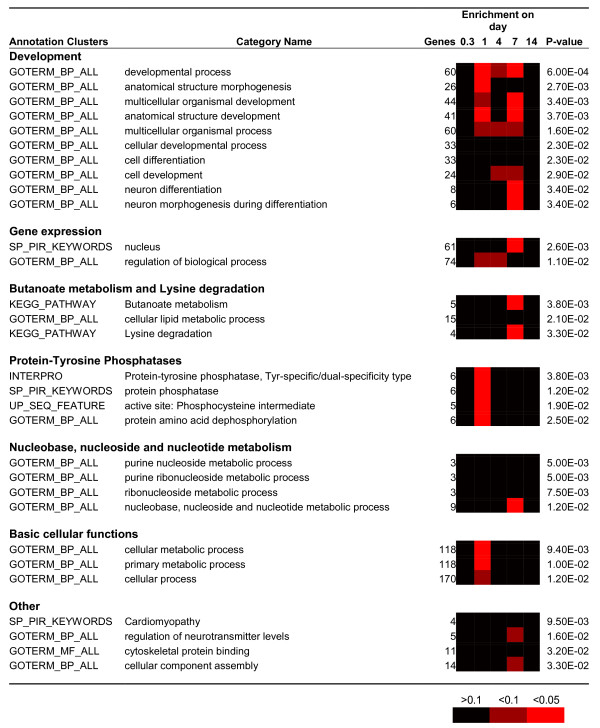
**Gene Ontology enrichment analysis of genes regulated in zebrafish gill by zinc depletion**. The closest human homologue to each regulated zebrafish gene at each time point was submitted to the online DAVID functional annotation classification tool and significantly overrepresented GO terms and other semantic descriptors were extracted and presented as clusters based on interrelationships between terms calculated by the tool. Probability for enrichment of each category is given in numerals for the entire data set (all genes from all time points). Levels of probability for enrichment at discrete time points are indicated by the heat-map.

Figure 3 lists the 30 most statistically significant annotation categories which either showed statistical enrichment in the combined analysis across all time points or at a single time point. These categories are similar to those overrepresented when using zebrafish gene identifiers, indicating the validity of the approach to use human orthologs as proxies in this analysis. We have omitted from Figure 3 all Gene Ontology (GO) terms for cellular component. Although these were some of the most significant categories they were not deemed to add additional information. There were six clusters of related annotations associating regulated genes with relatively defined functions (Figure 3). An additional four overrepresented annotations were also significant, but not obviously related.

'Developmental Process' was the most significantly overrepresented Biological Process GO term (*P *= 0.0006), involving 27% of all regulated genes (with annotation in DAVID). The 'gene expression' cluster grouped genes annotated for 'nucleus' (61 genes) and 'regulation of biological processes' (74 genes). Although not shown in Figure 3, these included 29 genes involved in 'transcription regulation', a Swiss-Prot keyword that was itself overrepresented (*P *= 0.048). The 'butanoate metabolism and lysine degradation' cluster comprised five genes which mapped onto the KEGG pathway for butanoate metabolism, four of which also appear in the KEGG pathway for lysine degradation. Four annotations from separate databases (InterPro domain, SwissProt Protein Information Resource, UniProt sequence feature, UniProt keyword, Gene Ontology Consortium) relating to 'protein-tyrosine phosphatases' appeared at a disproportionately higher frequency among regulated genes than expected to occur by chance (*P *= 0.0038 - 0.025). Four hierarchically linked GO terms relating to 'nucleobase, nucleoside and nucleotide metabolic process' were overrepresented among the regulated genes. A significantly disproportionate 75% of the regulated genes were labelled with the GO term 'cellular process' and related children terms. Among the listed top 30 most significant categories there were also four annotation terms that did not appear to be directly related to each other or any of the aforementioned clusters. These included terms such as 'regulation of neurotransmitter levels' (5 genes, *P *= 0.016) and 'cellular lipid metabolic process' (15 genes, *P *= 0.021).

**Figure 4:**
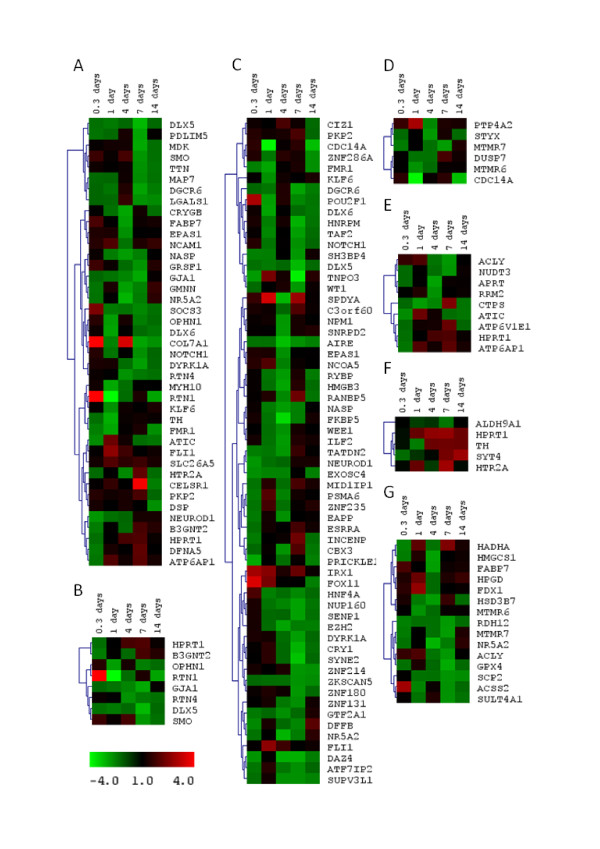
**Expression profiles for regulated genes, sharing enriched annotation categories, during zinc depletion**. Gene expression in gills at 0.3, 1, 4, 7, and 14 days of zinc depletion is given as fold-change ranging from -4 (green) to +4 (red, see colour bar) relative zinc adequate control fish. Gene trees were generated using Pearson Correlation and Average Linkage Clustering. The represented categories are (A) 'anatomical structure morphogenesis', (B) 'neuron differentiation', (C) 'nucleus', (D) 'Protein-tyrosine phosphatase, Tyr-specific/dual-specificity type', (E) 'nucleobase, nucleoside and nucleotide metabolic process', (F) 'regulation of neurotransmitter levels', and (G) 'cellular lipid metabolic process' as detailed in Figure 3.

Analysis across time points revealed that distinct functional groups were regulated at different stages of the progression of zinc deficiency (Figure 3 and 4). Significant overrepresentation of annotation terms (*P *< 0.05) within gene lists from specific sampling times was only observed for Days 1 and 7. For Day 0.3 (8 hours) and 14 this may be linked to the low power of the analysis because of the small numbers of genes. However, the paucity of enriched annotations in the gene list from Day 4 is intriguing since this time-point held the second highest number of regulated genes (Figure 2). Terms relating to protein-tyrosine phosphatases and to metabolic processes were overrepresented only at Day 1 (Figure 3). The former group consisted of six Class I cysteine-based protein-tyrosine phosphatases (Figure 4D), two of which were dual-specificity phosphatases (*dusp7*, *dusp12*) and two of which belong to the myotubularian subgroup (*mtmr6*, *mtmr7*). Genes involved in butanoate metabolism and lysine degradation were regulated on Day 4 or 7 and all five genes showed decreased expression in response to zinc deprivation. Regulation of developmental genes was prominent on both Days 1 and 7 (Figure 3 and 4A), but those involved in neuronal differentiation were only overrepresented on Day 7 (Figure 3 and 4B). The genes within the category 'regulation of neurotransmitter levels' were interesting because they tended to be down-regulated during the first 24 h of the experiment, followed by a later increase in expression (Figure 4F). Some functional categories, such as 'cardiomyopathy', 'cellular lipid metabolic process' and 'cytoskeletal protein binding', were not overrepresented at any of the time-points but statistically enriched in the combined list of all regulated genes (Figure 3).

Nuclear proteins, such as transcription factors, were substantially overrepresented (Figure 3). The zebrafish and human 'orthologue' identities of genes classified as "transcription factors" are shown in Table 3, and temporal expression profiles of those annotated with the term 'nucleus' illustrated in Figure 4C. Their general temporal profile was similar to that observed globally for all genes, with similar percentages of up- and down-regulated genes at the 8 hours, 1, and 14 day time points, and the majority being down-regulated on Days 4 and 7. There was also no particular bias for transcription factors with zinc motifs to be down-regulated. Like other genes most of the regulated zinc-finger and RING-finger genes were down-regulated, but the genes for the zinc-finger proteins *hnf4a*, *znf235 *(LOC100005466) and *esrra (esrra1) *were induced by zinc deficiency (Table 3, Figure 4C). In this context *hnf4a *became particularly interesting because increased expression of this gene was already observed 8 hours into the experiment but at no time-point thereafter. This quick and transient regulation indicates that Hnf4a may be of importance for the early response to zinc-deprivation. The other transcription factors whose expression were up-regulated during the first 8 hours of zinc-deprivation included *pou2f1b*, *foxl1*, and *irx1*. Of these only *foxl1 *remained induced after 24 hours, along with *fli1 *and *znf235 *(LOC100005466). On Days 4 and 14 there were no transcription factors up-regulated, but on Day 7 expression of *cbx3a*, *nkx2.1a *and *esrra *were increased relative to the control.

**Table 3:** Transcription factors regulated (>1.8-fold and FDR = 0.1) by zinc depletion for 8 hours to 14 days.

Time point	Unigene ID	Gene Description	**Human homologue**^**a**^	**Fold Change**^**b**^
8 h	Dr.15390	Forkhead box L1 (foxl1)	FOXL1	3.59
	Dr.77946	Wu:fc07d06 (wu:fc07d06)	POU2F1	3.01
	Dr.77235	Iroquois homeobox protein 1, b (irx1b)	IRX1	2.94
	Dr.34224	Si:ch211-272f15.2 (si:ch211-272f15.2)	CRSP3	2.13
	Dr.107820	Hepatocyte nuclear factor 4, alpha (hnf4a)	HNF4A	1.90
	Dr.75801	Neurogenic differentiation (neurod)	NEUROD1	-1.96
				
1d	Dr.78408	Friend leukemia integration 1a (fli1a)	FLI1	2.68
	Dr.15390	Forkhead box L1 (foxl1)	FOXL1	2.42
	Dr.77786	Hypothetical protein LOC100005466 (LOC100005466)	ZNF235	1.82
	Dr.158	Distal-less homeobox gene 4a (dlx4a)	DLX6	-2.23
	Dr.77265	Core promoter element binding protein (copeb)	KLF6	-1.83
	Dr.32618	Homeo box A3a (hoxa3a)	HOX3A	-1.82
				
4d	Dr.15418	Hypothetical protein LOC792149 (wu:fc23f06)	TCF2	-2.62
	Dr.53400	Nuclear receptor subfamily 5, group A, member 1a (nr5a1a)	NR5A2	-2.53
	Dr.75880	Zgc:112073 (zgc:112073)	HMGB3	-2.25
	Dr.76424	Enhancer of zeste homolog 2 (Drosophila) (ezh2)	EZH2	-2.21
	Dr.77670	RING1 and YY1 binding protein (rybp)	RYBP	-2.04
	Dr.76824	Interleukin enhancer binding factor 2 (ilf2)	ILF2	-2.04
	Dr.105034	Zgc:153397 (cbx3)	CBX3	-1.92
	Dr.78440	General transcription factor IIA, 1 (gtf2a1)	GTF2A1	-1.82
	Dr.83118	Similar to hypoxia-inducible factor 2 alpha (LOC566886)	EPAS1	-1.82
				
7d	Dr.105034	Zgc:153397 (zgc:153397)	CBX3	1.97
	Dr.82318	NK2 homeobox 1a (nkx2.1a)	NKX2-4	1.88
	Dr.30382	Estrogen-related receptor alpha-like (esrral)	ESRRA	1.80
	Dr.429	Distal-less homeobox gene 5a (dlx5a)	DLX5	-2.63
	Dr.132864	Wilms tumor 1a (wt1a)	WT1	-1.99
	Dr.78812	Hypothetical LOC568537 (LOC568537)	ZNF214	-1.95
	Dr.23936	Si:rp71-1g18.1 (si:rp71-1g18.1)	ZKSCAN5	-1.89
	Dr.118285	Similar to PHD finger protein 12 (LOC560119)	PHF12	-1.84
	Dr.75753	Notch homolog 1a (notch1a)	NOTCH1	-1.83
	Dr.116189	Hypothetical LOC573287 (LOC573287)	ZNF131	-1.81
				
14d	Dr.78812	Hypothetical LOC568537 (LOC568537)	ZNF214	-1.83

^a^The closest human homologue with sequence similarity higher than 45% are listed.

^b ^Positive numbers indicate up-regulation and negative numbers represent down-regulation

### Analysis of transcription factor binding sites (TFBS) in the 5' region of regulated genes

**Figure 5:**
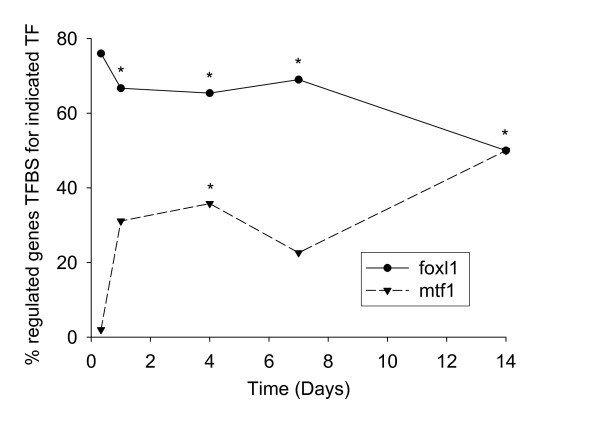
**Frequency of transcription factor binding sites (TFBS) to Foxl1 and Mtf1 among regulated genes**. The binding sites are located within 2000 bp DNA sequence upstream the ATG of genes that were regulated by zinc depletion at different time-points. TFBS to Foxl1 and Mtf1 were present in 90.4% and 18.7%, respectively, of 198 randomly selected control genes that showed stable expression throughout the experiment. A significant difference relative to that in the control genes is indicated by an asterisk (*P *< 0.05, z-test).

To investigate the cascade effects that might result from changes in expression of the TFs listed in Table 3 we extracted the upstream DNA sequence of the regulated genes and searched for the occurrences of their binding site (TFBS) as defined by the Transfac database [32] Of the TFBS for the transcriptionally regulated TFs mapped to entries in the Transfac (2005) database (using gene names and synonyms) only genes with TFBSs for *foxl1 *and *hnf4a *were significantly overrepresented among regulated genes relative to the non-regulated control genes (*P *< 0.05; Figure 5). Interestingly, both *foxl1 *and *hnf4a *were already differently regulated at 8 hours after the start of zinc restriction and were among the few TFs up-regulated (Table 3 and Figure 4C). Over 11% of the genes regulated at 8 hours had at least one TFBS to *hnf4a *within 2000 bp upstream of the translation start site, compared with only 1.5% of the control genes. Curiously, the effect of zinc deficiency on the frequency of upstream Foxl1 binding sites appeared to be opposite; from 24h and onwards the frequency of TFBS to Foxl1 was significantly reduced. Although *mtf1 *mRNA expression did not change under zinc deficiency in this experiment Mtf1 is a well-recognised regulator of cellular zinc homeostasis [33]. Hence binding motifs for Mtf1 (MREs) were also included in the analysis. The frequency of MREs among regulated genes generally increased during the time of zinc deficiency treatment, but was only statistically higher than that in non-regulated genes on Day 4. Among the genes that showed decreased mRNA levels were *mt2 *and *znt1*, both known to be regulated by Mtf1 [29,33].

### Protein interaction analysis reveals an important pathway in response to zinc depletion

**Figure 6:**
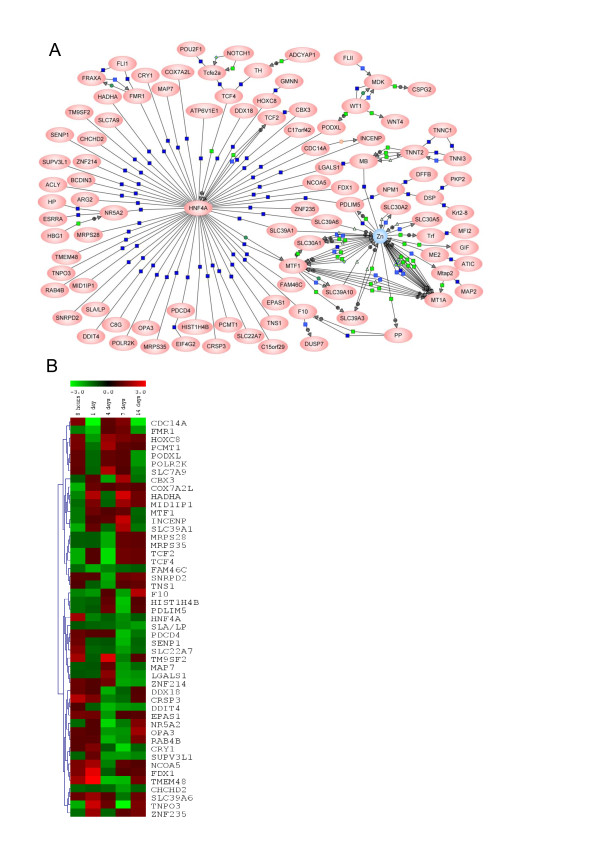
**Molecular interactions between zinc and proteins encoded by genes changed under zinc depletion**. MTF1 was added to the input list although its mRNA expression was not changed in the present study. (A) A Direct Interaction Network based on curated interactions contained within the PathwayArchitect database (Additional file 2, Figure S1). Red ovals represent proteins and the blue circle symbolises Zn(II). Dark blue squares denote 'binding', and light blue squares 'expression'; green squares stand for 'regulation', green diamonds for 'metabolism', and green circles for 'promoter binding'. Arrow heads indicate directionality of the interaction where annotated. (B) Heatmap of expression levels of transcripts that interact directly with HNF4A during 14 days of zinc depletion.

The molecular interactions between zinc and proteins encoded by regulated transcripts were explored and visualised using the PathwayArchitect^® ^software. A continuous 'Direct Interaction Network' was generated consisting of 106 nodes and 157 edges (Figure 6). An interactive version of the network is presented where nodes and edges can be individually explored (Additional file 2, Figure S1). By far the highest numbers of interactions were found for connections with zinc and for connections with Hnf4a, hepatonuclear factor 4 alpha. Hnf4a was positioned as a hub interacting with 55 nodes including Slc39a1 (Zip1), Slc39a6 (Zip6), and Mtf1 (Figure 6). Interestingly there is experimental evidence for binding of Hnf4a to promoter elements in the majority of these genes [34]. This analysis further implicates Hnf4a in zebrafish gill as a key regulator during acclimation to a zinc deficient condition. Zinc itself was a second hub in the network interacting with a number of proteins including Mtf1, Mt, and several zinc transporters of the Slc30a and Slc39a families, which were found to be regulated either by microarray or qPCR (Table 1, [15]).

## Discussion

The fish gill provides an elegant experimental system for studying mineral absorption *in vivo *since the concentration and speciation of metal ions on the apical side can be carefully controlled, and this organ provides the major uptake pathway for ions, including zinc [35]. The teleost fish gill epithelium is comprised of at least five types of cells, including respiratory pavement cells, ion transporting ionocytes (also termed 'chloride cells'), mucous cells, and neural epithelial cells [36]. These are mounted on a cartilage scaffold forming the gill filaments and secondary lamellae where gas and ion exchange with water takes place [36]. Gill zinc uptake is transcellular and primarily takes place in the discrete ionocytes which transport zinc apically from the water and extrude it basolaterally to the opposite side of the epithelium [37]. Furthermore, the gill is highly dynamic, responding to changes in the external environment to which it is in intimate contact, by remodelling its structure and cellular metabolism in order to maintain the homeostasis of essential micronutrients [30,38,39]. In the present study the genomic resources of the zebrafish have been exploited to investigate the molecular sequences of events invoked under zinc depleted conditions. This aims to reveal the temporal sequence of events that follow treatment with zinc depletion and provides an opportunity to identify possible global regulators through bioinformatic reverse engineering.

We have established that zinc deficiency was efficiently induced in zebrafish after a 14-day depletion of zinc in both the water and diet. This indicates that any compensatory changes invoked were insufficient to fully maintain whole body zinc concentrations, which were significantly reduced at the end of the experimental period (Figure 1). Intriguingly the unidirectional gill zinc influx was found to be suppressed throughout the experimental period. This suppression of zinc uptake is counterintuitive since it may be expected that under conditions of reduced zinc availability proteins involved in zinc acquisition, such as zinc importers, would be up-regulated in an attempt to maintain zinc status. Indeed, we have shown previously data from the same experiment demonstrating increased expression of the zinc importers, *zip3*, *zip10 *and *znt5*, in response to zinc depletion [15]. However, there was also down-regulation of the basolateral zinc exporter *znt1*, which would limit zinc transfer into the blood stream. Overall our data would suggest that zinc uptake was rate-limited by the basolateral transfer. However, further detailed physiological studies are required to confirm this interpretation.

The low zinc supply also had an effect on whole body copper status, which interestingly was reduced. The use of zinc to treat copper hyper-accumulation in Wilson's disease patients provides evidence that zinc inhibits copper uptake [40], an observation confirmed with our own zinc supplementation experiments [30]. This may seem to contradict the observation that zinc deficiency also reduced whole body copper status. One possible explanation is that the reduction in zinc stores probably reduced expression of Mt, which binds copper as well as zinc. Mt binds about one third of the copper in a typical fish liver, but zinc is a much better inducer of *mt *expression [41].

The exploitation of microarray technology identified a total of 331 genes representing the molecular response of the gill epithelium to zinc depletion. These gene expression profiles from gill tissue provide an integrated image of zinc regulation occurring in a collection of cell types. Despite Zn^2+ ^influx and whole body zinc content remaining low by the end of the 14-day experimental period, the time-course of overall gene expression indicated establishment of a new steady-state, only 21 genes remained regulated on Day 14 compared with 156 genes on Day 7. The genes regulated during the acclimation to zinc depletion are involved in a variety of biological processes, reflecting the multifaceted biochemical functions of zinc. The transitory nature of the acclimation process was characterised by a succession of functional gene categories that were regulated; transcripts for protein tyrosine phosphatases, anatomical structure morphogenesis, and cellular metabolic processes being preferentially expressed early in the experiment (Day 1), while enrichment of transcripts for proteins involved in nuclear functions, neuron differentiation, and cellular component assembly occurred much later (Day 7). Other functional categories such as 'purine ribonucleoside metabolism process', 'cardiomyopathy', and 'cellular lipid metabolic process' were significantly enriched over the entire time period but not at any particular time-point. Enrichment analysis using zebrafish gene identifiers was limited by the relatively sparse representation of zebrafish genes in the DAVID database. However, the preferential regulation of genes involved in development could be confirmed and a specific enrichment of genes involved in cell migration during development (i.e. *slc39a6*, *wnt4a*, and *esrra*) was observed. The latter group may be of considerable interest considering recent studies implicating zinc as a regulator of the ectoderm-mesenchyme transition (EMT) during zebrafish gastrulation and human cancer [42,43].

The majority (72%) of the 29 regulated transcription factors were down-regulated under the zinc depleted condition. It is notable that among these genes there were 11 zinc finger proteins, and it may therefore be speculated that the effect of zinc deficiency is a feed-back response to a rather non-specific depletion of zinc from zinc finger domains. However, eight out of the 11 zinc finger proteins were down-regulated, which means that the percentage of down-regulated genes for zinc finger proteins (73%) was practically the same as that for the general cohort of regulated transcription factors. Nearly half (12) of the regulated TFs are involved in regulation of development, including *foxl1, neurod1, fli1, nr5a1a, klf6, hmgb3b *(*zgc:11207*3), *epas1, nkx2.1a, irx1, dlx4a, dlx5a, and notch1a*. This may help explain the fact that developmental impairment is one of the key features of zinc deficiency [5]. Furthermore, knockout of *Mtf1 *is lethal to mouse embryos but not at post-embryonic stages [44]. It is possible that zinc signalling during development may normally modulate the expression of these TFs and their downstream targets. In terms of the significance to the fish in our experiment, it is likely that we are observing the signalling for stem-cells in the gill to differentiate and proliferate to structurally modify the gill in an attempt to regain homeostasis. The fish gill is an extremely dynamic structure shown to be reprogrammed and structurally remodelled when faced with environmental changes such as hypercapnia [45] and hypothermia [39]. We propose that similar changes are occurring during zinc depletion.

Four key regulators of development containing the homeobox sequences were identified as regulated by zinc deficiency. Among these were *dlx4a *and *dlx5a *(closest human homologues *DLX6 *and *DLX5*, respectively), homologous to the Drosophila distal-less gene (*dll*). Zinc deficiency caused down-regulation of *dlx4a *and *dlx5a *(Figure 4) while zinc supplementation has been found to induce expression of *dlx2a *(closest human homologue, *DLX2*) another closely related gene in the same family [30]. All members of the *dlx *family may be involved in formation of cartilage and/or bone and contribute to the differentiation of mineralising cell types, including osteoblasts and chondrocytes [46,47]. This is of interest because zinc is abundant in bone and cartilage and recent studies have demonstrated the involvement of zinc transporters in the development of such tissues [48-50]. Specifically, *Znt5*-null mice develop hunched backs, show impaired growth, and decrease in bone density due to osteopenia [50]. Foetuses of mice lacking one of the essential zinc importers, *slc39a4 *(*zip4*), become critically zinc deficient and show severely underdeveloped and deformed craniofacial and limb features [48]. Knock-out for another zinc transporter of the same family, *slc39a13 *(*zip13*), in mice results in a phenotype with defects in bone, teeth, and connective tissue, linked to changes in the BMP signalling pathway [48,49]. This pathway involves both DLX5 and DLX6, and targeted inactivation of *Dlx5 *and *Dlx6 *genes in mice results in abnormalities very similar to those observed in *Slc39a4 *null mice [51]. The direct effect of zinc on osteogenic differentiation is strongly supported by another recent study which showed that zinc deficiency suppresses matrix mineralisation and delays osteogenic activity in mouse osteoblastic MC3T3-E1 cells through downregulation of *Runx2 *(runt-related transcription factor 2) [52], while Dlx5 has been shown to induce expression of *Runx2 *and initiate osteogenic differenciation in chick calvaria osteoblasts *in vitro *[53]. Together these data suggest that zinc deficiency, caused by reduced zinc uptake or disruption of zinc transporters, leads to dysregulation of mineralising cells in a process that involves suppression of DLX5/6 expression and downstream effects on osteoblast differentiation.

A major objective of this study was to identify possible key master regulators responsible for orchestrating the response to zinc depletion. In the present study Hnf4a (hepatocyte nuclear factor 4, alpha) was repeatedly implicated as being of potential importance for coordination of gene expression during the early stages of acclimation to zinc restriction. First, *hnf4a *was one of four transcription factors up-regulated upon zinc depletion at the first sampling point 8 hours (Table 3 and Figure 4C). Second, in a 'Direct Interaction Network' assembled by PathwayArchitect™ from all regulated genes during the experiment, *hnf4a *was positioned as a hub interacting with 55 nodes, including genes for the zinc-regulatory proteins Slc30a1 (Zip1), Slc30a6 (Zip6) and Mtf1. Third, analysis of promoter sequences of the regulated genes revealed an overrepresentation of genes with TFBSs associated with Hnf4a. Hnf4a does not require binding of a ligand to be activated, instead, binding to its cognate regulatory DNA elements (DR-1) is modified by serine/threonine and tyrosine phosphorylation, which can be catalysed by several protein kinases [54]. Spatial and temporal specificity of target gene transactivation is believed to be controlled by expression of cofactors interacting with Hnf4a. Transcription of *hnf4a *can be repressed by Snai1a (Snail, snail homolog 1a (*Drosophila*)) [55], which has been shown in zebrafish to respond an increase in cellular labile Zn^2+ ^concentration by nuclearisation and repression of target genes [43]. Hence, the early increase in *hnf4a *expression is plausibly a result of a reduced inhibition by Snail, as it is likely that the sudden depletion of zinc from the water would reduce the concentration of labile Zn^2+ ^in the gill cells. However, it was not investigated if during the first 8 hours of the experiment there was an increase in Hnf4a protein level large enough to impact expression of Hnf4a targets. Because of the profound influence of Zn^2+ ^on protein phosphatase activities [56] it is also possible that a change in Hnf4a phosphorylation state contributed to the high representation of Hnf4a regulated genes. Hnf4a is known as a key regulator in the maintenance of hepatocyte differentiation and the control of lipid homeostasis [57]. A recent study has shown that zinc supplementation can partially attenuate alcohol-induced lipid accumulation in mice by reversing alcohol-mediated inactivation of Hnf4a and Ppara [58]. Zinc deprivation inactivated the DNA binding ability of Hnf4a and Ppara, thus regulating its target genes involved in lipid metabolism. These data not only provide further support for the key function of hnf4a in zinc deficiency found in the present study, but are also consistent with our findings of significant regulation of genes involved in 'cellular lipid metabolic process' (*P *= 0.021).

One of the principal mechanisms of non-genomic zinc signalling may be through its ability to inhibit protein phosphatases at nanomolar concentrations, leading to increased phosphorylation and activity of several MAP and tyrosine kinases [56]. There is increasing evidence that this may be a common mechanism for zinc involvement in a variety of conditions including diabetes, neuronal damage during post-transfusional ischemia [59], and cancer [60]. In the present study tyrosine-specific/dual-specificity phosphatases were overrepresented among genes regulated 24 hours after introduction of zinc depletion. Effects of zinc depletion on phosphatase activities in gill cells would be expected to occur within minutes [56]. Hence, the changes in abundance of mRNA for phosphatases, primarily at the 24 hour time point, could represent a delayed transcriptional response to an earlier change in regulation of phosphatases by zinc at the protein level. Such a component of zinc signalling would be of significance to understanding of the overall effects of zinc on phosphatases and consequently MAP kinase and tyrosine kinase activities.

## Conclusions

In conclusion, the experimental design to generate transcription profiles for gills at five time points over a 14-day period allowed us to study the changes in expression of individual genes during the period of acclimation to a zinc depleted environment. A striking feature of the transcription profiles was that although there was a continuum of functional and structural categories of genes regulated during the experiment, there was little continuity in regulation of individual genes except *mt2*. This oscillating pattern of gene regulation may reflect a homeostatic control mechanism that is gradually adjusting the gill to the new zinc depleted condition. We know from qPCR analysis that zinc importers were maximally up-regulated at day 7, coinciding with maximum down-regulation of the basolateral zinc exporter *znt1 *[15]. Network analysis of transcriptomics data indicated that effects on phosphatases and a few key transcriptional factors, such as Hnf4a, coordinated early (up to Day 4) responses and may be involved in direct regulation of *slc39a1 *(*zip1*) and *slc39a6 *(*zip6*) and other zinc effector molecules. Subsequent events, which culminated on day 7, appeared to entail stem cell proliferation and differentiation in attempt to regain zinc homeostasis.

It is common to see microarray studies with only a single time point represented. While such studies often provide very useful information the present study, which contained the resolution of 49 microarray samples spread over two conditions and five time-points, clearly demonstrates that genes may be regulated in the opposite direction at different time-points and that sets of genes regulated at different time points may have similar ontologies but largely different identities.

## Methods

### Animal husbandry and experimentation

The zebrafish husbandry and treatment were described previously [15]. Briefly, juvenile zebrafish, *Danio rerio *(0.44 ± 0.06 g), were obtained from Neil Hardy Aquatica Ltd. (Surrey, UK). The fish were divided into three experimental groups with each group held in four identical tanks (40 fish per tank), and supplied with a continuous flow of aerated reconstituted reverse osmosis water at 26-28°C. The reconstituted water was composed of 0.6 mM NaCl, 41 μM Na_2_SO_4_, 13.6 μM KCl, 150 μM CaCl_2_, 3.4 μM NaHCO_3_, 78 μM MgCl_2_. In addition, each tank was equipped with a dosing system which added Zn, as ZnSO_4_.7H_2_O (BDH Chemicals), to provide a nominal zinc concentration in the tanks of 0.25 μM (16.3 μg L^-1^). The fish were fed a purified mash diet (Fish Nutrition Unit, University of Plymouth, UK) containing an analysed zinc concentration of 233 mg kg^-^1 (3.56 mmol kg^-1^) at a rate of 4% of their body mass per day. After one-week acclimation zinc deficiency was induced to a group of fish spread over four tanks by changing to reconstituted water without additional zinc supplementation and providing a low zinc (26 mg kg^-1^) diet. The water and diet conditions for one group of four tanks remained as before, serving as the control. A third group was cultured under zinc supplementation conditions, and the results of this experiment are presented in Zheng et al. [30]. The zinc concentrations in the water were monitored daily using Inductively Coupled Plasma Mass Spectroscopy (PerkinElmer Elan DRC ICP-MS). The measured zinc concentrations for zinc depletion and control groups were 0.04 ± 0.05 μM (2.61 μg L^-1^) and 0.25 ± 0.09 μM (16.3 μg L^-1^), respectively. The experiment continued for 14 days and no mortalities or necropsies were observed.

### Micronutrient measurement of whole body

At the end of two weeks nine fish from each group were killed by overdose of benzocaine (Sigma, USA) and analysed for whole body electrolyte and trace elements according to Handy et al. [61] with modifications (gills were dissected for gene expression analysis). Briefly, the individual fish was over dried at 100°C to a constant weight (for 48 h) and then digested in 2 ml of concentrated nitric acid at 70°C for 8 h. The digested samples were diluted to 6 ml with ultrapure water and then analysed for Ca, Cu, Fe, K, Na, and Zn using inductively coupled plasma optical emission spectrophotometry (ICP-OES, Varian 725-ES) against matrix matched standards. Nutritional parameters for moisture, protein, ash, and lipid were also determined, in triplicate where possible, according to Baker and Davies [62] with modifications. A micro-Kjeldahl digestion system was used with a 100 ml digestion tube according to the manufacturer's specification. Two fish were pooled for each protein determination and samples prepared using a Gerhardt KB40S digestion block and a Vapodest 40 distillation unit.

### Zinc influx assay

Unidirectional whole body influx of Zn^2+ ^was measured as described previously [25]. Under the assay conditions the whole body uptake of Zn^2+ ^from the water represents almost exclusively influx across the gills. Each of the two flux bags was filled with 2 L of the reconstituted water with 5 μM Zn^2+^. The flux bags were equipped with an airline and placed in a thermostatically controlled water bath set at 28°C. For each time point a total of eight fish from each treatment group (two from each of the four tanks) were transferred into a flux bag. Half an hour later, 0.25 MBq of carrier-free ^65^Zn^2+ ^was added to each flux bag. Water samples were withdrawn from the flux-bags before and after the introduction of ^65^Zn^2+^. After three hours incubation with ^65^Zn^2+ ^the fish were killed by benzocaine, rinsed in a 50 μM solution of zinc to remove surface-bound ^65^Zn^2+^, blotted dry and weighed. The ^65^Zn^2+ ^radioactivity of each fish and water sample were measured in an LKB1282 CompuGamma counter, and the actual zinc concentration from the non-radioactive water sample measured by ICP-MS. The unidirectional influx of Zn^2+ ^was calculated according to the formula, J_in_= cpm (SA bw t)^-1^, where SA is the specific activity of ^65^Zn^2+ ^in the water, calculated as [^65^Zn^2+^] divided by the total [Zn^2+^] (cpm pmol^-1^), bw is the individual fish body weight, and t is the duration of flux (h).

### RNA labelling and microarray

Total RNA was extracted from dissected gill samples of individual fish using TRIzol Reagent (Invitrogen) according to the manufacturer's protocol. The RNA samples were further subject to DNase I digestion using a DNA-free kit (Ambion). The quality of the RNA samples was assessed using the Agilent 2100 Bioanalyser (Agilent Technologies). The reference RNA was purified from whole bodies of untreated zebrafish. The amino-allyl indirect labelling method was used to obtain Cy3 (reference) or Cy5 (samples)-labelled cDNA. The Cy3- or Cy5- cDNA sample was individually purified using QIAquick PCR purification columns (Qiagen).

The zebrafish oligonucleotides arrays were spotted on UltraGAPS™ coated slides (Corning Life Sciences, Promega), using a Qarray2 robot (Genetix Ltd) at the King's College London Genomics Centre, UK. The 16,399 oligonucleotides were designed and synthesised by Compugen and Sigma Genesys as a Zebrafish OligoLibrary ready set, which represent 15,806 LEADS™ clusters plus 171 controls. In addition, 331 customised oligonucleotides and 23 Cl Scorecard™ were added to the array set.

The arrayed slides were pre-hybridised in a buffer containing 25% formamide, 5× SSC, 0.1% SDS, and 1 mg/ml BSA at 42°C for 45 min, washed in double-distilled H_2_O three times and then air-dried. Equal amounts of Cy3 and Cy5-labelled cDNA were combined and hybridised in 1× hybridisation buffer, containing 25% formamide, 5× SSC, 0.5× Denhardt's, 0.1 μg/μl yeast tRNA, and 0.1% SDS, at 42°C for 16-20 hours in a sealed humid hybridisation chamber (Camlab). The slides were washed for 5 min sequentially in 2× SSC/0.1% SDS buffer (at 42°C), 0.1× SSC/0.1% SDS buffer and 0.1× SSC buffer, air-dried and scanned using a ScanArray (Perkin Elmer).

### Data analysis

A total of 49 microarrays, with RNA from one fish per array, were analysed across two treatment groups and five time points. Spot intensities from each scanned image were quantified using Bluefuse software (BlueGnome) which is based on the Bayesian statistical method. Lowess normalisation was applied to the quantified data set. Probe intensities with low confidence (*P *< 0.05) were filtered out and the normalised data uploaded onto GeneSpring 7.3X software (Agilent Technologies). The dataset is available from Gene Expression Omnibus (GEO) under accession numbers GSE21894 and GSE21914. Profiles generated from five fish of each group at each time point were processed and regarded as biological replicates, with the exception of the day 4 time-point for the control fish, which had four biological replicates. Only genes with expression data from at least three replicate samples were considered expressed and used for subsequent differential expression analysis. Gene expression profiles of the zinc deficient group were compared to those from the control group at the corresponding time point and the significant difference determined by *t*-test with Benjamini-Hochberg multiple testing correction (false discovery rate, FDR). The genes were deemed as differentially expressed with a fold change (FC) greater than 1.8 and P-value less than 0.1, controlling for a 10% FDR between two groups at each time point.

### Gene annotation and functional analysis

The oligonucleotide reporters were mapped at the sequence level (using megablast allowing 2 bp mismatches) to Ensembl (Zv5), Refseq, and Genebank. Genebank matches were used to retrieve the relevant Unigene Cluster ID which provided linkage to appropriate human orthologues. Due to the greater proportion of human genes with association to Gene Ontology (GO) terms, functional analysis of genes of interest was performed with the ontology associated with the corresponding human orthologues (with sequence similarity higher than 45%), together with those associated with the Zebrafish Unigene IDs. The enrichment of differentially expressed genes in GO terms and other functional and structural descriptors was carried out using the online Functional Annotation Tool DAVID (Database for Annotation, Visualisation and Integrated Discovery) http://david.abcc.ncifcrf.gov/[31], with the zebrafish or human genome used as the background population. DAVID interprets the biological functions of listed gene and provides statistical methods for discovering enriched annotations within gene lists. Significance is calculated by a modified Fisher's Exact test with Benjamini correction.

The interactions between zinc and regulated genes were evaluated using the PathwayArchitect^® ^software (Stratagene). The closest human orthologue to each of the genes designated as significantly regulated at one or more time points were imported into the software. Expression of zinc transporters in zebrafish gills from the same animal experiment have been analysed by qPCR and published [15]. Therefore, the significantly regulated zinc transporters, *slc39a *(*zip*) *1*, *3*, and *10*, and *slc30a *(*znt*) *1 *and *5 *were added to the list of objects. Because of its established role in zinc regulation metal-responsive transcription factor-1 (Mtf1) was also added to the component list. A Direct Interaction Network was generated by adding interactions (edges) between the entered components (nodes) from the PathwayArchitecht^® ^database.

### In silico analysis of regulated genes with binding sites for regulated transcription factors

Upsteam DNA sequences of genes that were differentially regulated at each sampling time were obtained from Ensembl (Zv5). Consequently, only those oligonucleotide entries mapped to Ensembl genes could be used for subsequent analysis of promoter and binding motifs for transcription factors (TFs). The number of analysed sequences from the 8 hour, 1, 4, 7, and 14 day time points were 26, 46, 82, 83, and 6, respectively. In addition to the regulated genes, upstream sequence was also obtained for a cohort of 198 randomly selected genes not significantly regulated at any sampling point during the experiment. DNA sequence corresponding to 2000 bp upstream of the translation start site were downloaded to a local server and used for analysis. Differentially regulated TFs (See Table 3) were identified by their GO annotation. The FindPatterns routine of the Wisconsin Package was used to locate the transcription factor binding site (TFBS) motifs (patterns and regular expressions), as defined by the Transfac (2005) database [32].

A perl script was used to parse the results, counting the occurrence of each TFBS found among genes in each list (Both the perl script and the GCG output file are available on request). The TFBSs that corresponded to differentially regulated TFs were recorded. The TFBS to Mtf1 was also included. At each time point the percentage of differentially expressed genes with TFBSs corresponding to each regulated transcription factor was calculated. To assess the general frequency of occurrence of specific TFBSs in the genome, the same procedure was repeated for 198 randomly selected, non-regulated genes. The frequency of genes with TFBS to a given TF was then compared between the lists of regulated and non-regulated genes to statistically assess any deviation from a random distribution (*P *< 0.05, z-test).

### Real-time Quantitative PCR (qPCR)

Total RNA (2 μg) from nine gill samples collected from each group at each time point was reverse transcribed into cDNA and subjected to qPCR analysis using the SYBR^® ^GreenER™ qPCR SuperMix kit (Invitrogen) according to the manufacturer's protocol except that 20 μl reaction volume was used. The qPCR validation was performed for 10 genes: *mt2*, *slc30a1 *(*znt1*), *ppara*, *foxl1*, *hmgcs1*, *apob*, *cyp11a1*, *dlx5*, *dlx2*, and *rfx2 *for which the primer sets are listed in Table 1. As a further validation of the microarray method the same set of genes were measured by qPCR on gill samples from zinc supplemented fish also analysed with the same microarray assay as that used in the present study [30]. The qPCR assays were performed on an ABI prism 7000 with cycling conditions as follows: 5 minutes of denaturation at 95°C and then 40 cycles of 95°C for 30s, 60°C for 1 minute. A standard curve was generated for each gene using serial dilutions of a concentrated cDNA mixture to assess the amplification efficiency. The relative copy number was deduced from the corresponding standard curve using the C_**t **_value. To correct for variation in the input RNA concentration the relative gene copies were further normalised to the expression of the 18s rRNA gene by dividing the number of gene copies with the relative copies of 18s. The statistical significance of differences between experimental groups was determined with two-tailed unpaired Student's *t*-test (*P *< 0.05).

## List of Abbreviations

MT: Metallothionein; MTF1: metal-responsive transcription factor 1; TF: transcription factor; TFBS: transcription factor binding sites; MRE: metal response element.

## Authors' contributions

DZ was responsible for the majority of the wet laboratory experimentation presented including both the microarray and physiological components. RH designed the experimental diets and performed the nutritional profiling, including metal analyses of fish. GF deigned a suite of custom array reporters representing the complete family of zebrafish zinc transporters and other zinc regulatory genes. PK performed the annotation of the array reporters and PC executed the bioinformatics TFBS queries. CH performed the interpretation of the TFBS query data including the reverse engineering of TF pathways and bioinformatics underpinning the network analysis. DZ, PK and CH were responsible for the statistical analysis of the array data and its subsequent functional interpretation. PK, GF, DZ and CH participated in conception and design and interpretation of the study. PK and CH drafted the manuscript and together with DZ and RH contributed to the iterative refinement of the article. All authors have read and approved the submitted version.

## Supplementary Material

Additional file 1 **Table S1 - Genes called regulated (>1.8-fold change, FDR = 0.1) in gills of zebrafish subjected to zinc depletion**. An annotated list of genes called significantly regulated with more than 1.8-fold change, compared to control, and FDR = 0.1 (*N *= 4 or 5) at one or more time points between 8 hours and 14 days. Annotation provided was determined by mapping the reporter sequences using megablast allowing 2 bp mismatches to Ensembl (Zv5), Refseq and Genebank. Genebank matches were used to retrieve the relevant Unigene Cluster ID and gene names (as provide in the table) and used to determine the associated human orthologues for which the unigene ID and gene symbol is also provided. The fold change in expression at each time point is also provided expressed relative to time match control. Click here for file

Additional file 2 **Figure S1 - Interactive Direct Interaction Network of responses to zinc depletion**. Mini web-site containing index.html and hyperlinked pages in subdirectory. The web site is an interactive version of Figure 6A containing curated interactions between regulated genes and respective proteins. Legend: Molecular interactions between zinc and proteins encoded by genes changed under zinc depletion. A Direct Interaction Network was created based on curated interactions contained within the PathwayArchitect database and provided through hyperlinks. Red ovals represent proteins and the blue circle symbolizes Zn(II). Dark blue squares denote 'binding', and light blue squares 'expression'; green squares stand for 'regulation', green diamonds for 'metabolism', and green circles for 'promoter binding'. Arrow heads indicate directionality of the interaction where annotated. Click here for file
