# Supplementary material for: Dynamic transcriptomic profiles of zebrafish gills in response to zinc depletion
Source: BMC Genomics. 2010 Oct 8;11:548. doi: 10.1186/1471-2164-11-548 (PMC3091697; doi:10.1186/1471-2164-11-548)
Supplement: Additional file 2 — Figure S1 - Interactive Direct Interaction Network of responses to zinc depletion. Mini web-site containing index.html and hyperlinked pages in subdirectory. The web site is an interactive version of Figure 6A containing curated interactions between regulated genes and respective proteins. Legend: Molecular interactions between zinc and proteins encoded by genes changed under zinc depletion. A Direct Interaction Network was created based on curated interactions contained within the PathwayArchitect database and provided through hyperlinks. Red ovals represent proteins and the blue circle symbolizes Zn(II). Dark blue squares denote 'binding', and light blue squares 'expression'; green squares stand for 'regulation', green diamonds for 'metabolism', and green circles for 'promoter binding'. Arrow heads indicate directionality of the interaction where annotated. [file 1471-2164-11-548-S2.ZIP › PathwayArchitect Zn def DIN2/100630.html]

# PROTEIN: MAP2

|  |  |
| --- | --- |
| Name | MAP2 |
| Type | PROTEIN |
| Description | microtubule-associated protein 2 |
| Note | This gene encodes a protein that belongs to the microtubule-associated protein family. The proteins of this family are thought to be involved in microtubule assembly, which is an essential step in neurogenesis. The exact function of this gene is still unknown. The products of similar genes in rat and mouse are neuron-specific cytoskeletal proteins that are enriched in dentrites, implicating a role in determining and stabilizing dentritic shape during neuron development. A number of alternatively spliced variants of this gene have been described, but the full length nature of some of them has not been determined. |
| Alias | MAP2B |
|  | MAP-2 |
|  | MAP 2 |
|  | MAP2 |
|  | MAP2C |
|  | MAP2A |


---

|  |  |
| --- | --- |
| GO Component | microtubule associated complex |
|  | microtubule |
|  | cytoskeleton |


---

|  |  |
| --- | --- |
| GO ID | GO:0005856 |
|  | GO:0005875 |
|  | GO:0005874 |
|  | GO:0007026 |
|  | GO:0005516 |
|  | GO:0005198 |


---

|  |  |
| --- | --- |
| MIM | MIM:157130 |


---

|  |  |
| --- | --- |
| Connectivity | 68 |


---

|  |  |
| --- | --- |
| Entrez ID | 4133 |


---

|  |  |
| --- | --- |
| Agilent ID | A\_14\_P110374 |
|  | A\_14\_P132994 |
|  | A\_14\_P112368 |
|  | A\_23\_P153920 |
|  | A\_23\_P153917 |
|  | A\_24\_P231483 |
|  | A\_14\_P133615 |


---

|  |  |
| --- | --- |
| Cellular Localization | Cytoskeleton |
|  | Microtubule |
|  | Organelle |
|  | Cell |


---

|  |  |
| --- | --- |
| Pathway | Zn def RIN |
|  | Zn def DIN |


---

|  |  |
| --- | --- |
| GO Process | negative regulation of microtubule depolymerization |


---

|  |  |
| --- | --- |
| UniGene | Hs.368281 |


---

|  |  |
| --- | --- |
| Affymetrix Probeset ID | 222892\_s\_at |
|  | 183\_at |
|  | 1972\_s\_at |
|  | 210015\_s\_at |
|  | 219\_i\_at |
|  | 220\_r\_at |
|  | 225540\_at |
|  | 35422\_at |
|  | 54897\_at |
|  | 68497\_r\_at |
|  | 73409\_f\_at |
|  | 84865\_at |
|  | 89576\_at |
|  | g1850616\_3p\_a\_at |
|  | Hs.169387.1.S1\_3p\_at |
|  | S76756\_s\_at |
|  | U01828\_at |
|  | 73407\_i\_at |
|  | 38742\_s\_at |
|  | 47941\_at |
|  | 75078\_at |
|  | Hs.16740.0.S1\_3p\_a\_at |
|  | Hs.191856.0.A1\_3p\_at |
|  | RC\_AA148603\_at |
|  | RC\_AA436618\_at |
|  | RC\_T90345\_at |


---

|  |  |
| --- | --- |
| GO Function | structural molecule activity |
|  | calmodulin binding |


---

|  |  |
| --- | --- |
| Nucleotide | NM\_031845 |
|  | BC066648 |
|  | BC027583 |
|  | M25668 |
|  | S76756 |
|  | NM\_031847 |
|  | BC038857 |
|  | NM\_002374 |
|  | AB209330 |
|  | U34061 |
|  | U89329 |
|  | AV718243 |
|  | NM\_031846 |
|  | U01828 |
|  | U89330 |


---

|  |  |
| --- | --- |
| Protein | AAH27583 |
|  | NP\_002365 |
|  | NP\_114033 |
|  | NP\_114034 |
|  | AAH38857 |
|  | AAA03354 |
|  | NP\_114035 |
|  | AAB48098 |
|  | AAH66648 |
|  | AAB48097 |
|  | BAD92567 |
|  | AAB33379 |
|  | AAA59552 |
|  | P11137 |


---

|  |  |
| --- | --- |
| Organism | Mammal |


---

|  |  |
| --- | --- |
| Location | chromosome 2, 2q34-q35 (Homo sapiens) |


---

|  |  |
| --- | --- |
