# Supplementary material for: Dynamic transcriptomic profiles of zebrafish gills in response to zinc depletion
Source: BMC Genomics. 2010 Oct 8;11:548. doi: 10.1186/1471-2164-11-548 (PMC3091697; doi:10.1186/1471-2164-11-548)
Supplement: Additional file 2 — Figure S1 - Interactive Direct Interaction Network of responses to zinc depletion. Mini web-site containing index.html and hyperlinked pages in subdirectory. The web site is an interactive version of Figure 6A containing curated interactions between regulated genes and respective proteins. Legend: Molecular interactions between zinc and proteins encoded by genes changed under zinc depletion. A Direct Interaction Network was created based on curated interactions contained within the PathwayArchitect database and provided through hyperlinks. Red ovals represent proteins and the blue circle symbolizes Zn(II). Dark blue squares denote 'binding', and light blue squares 'expression'; green squares stand for 'regulation', green diamonds for 'metabolism', and green circles for 'promoter binding'. Arrow heads indicate directionality of the interaction where annotated. [file 1471-2164-11-548-S2.ZIP › PathwayArchitect Zn def DIN2/106662.html]

# PROTEIN: TCF2

|  |  |
| --- | --- |
| Name | TCF2 |
| Type | PROTEIN |
| Description | transcription factor 2, hepatic; LF-B3; variant hepatic nuclear factor |
| Note | TCF2 encodes transcription factor 2, a liver-specific factor of the homeobox-containing basic helix-turn-helix family. The TCF2 protein is believed to form heterodimers with another liver-specific member of this transcription factor family, TCF1; depending on the TCF2 isoform, the result may be to activate or inhibit transcription of target genes. Mutation of TCF2 that disrupts normal function has been identified as the cause of MODY5 (Maturity-Onset of Diabetes, Type 5). A third human transcript variant is believed to exist based on such a variant in the rat: however, to date such an mRNA species has not been isolated. |
| Alias | TCF-2 |
|  | Hnf1b |
|  | Hnf-1b |
|  | vHNF1 |
|  | HNF-1B |
|  | VHNF1 |
|  | Transcription factor 2 hepatic; LF-B3; variant hepatic nuclear factor |
|  | HNF-1beta |
|  | hepatocyte nuclear factor-1 beta |
|  | MODY5 |
|  | Tcf2 |
|  | Transcription factor 2 |
|  | HNF-1Beta |
|  | transcription factor 2, hepatic |
|  | HNF2 |
|  | Tcf-2 |
|  | TCF2 |
|  | Transcription factor 2, hepatic; LF-B3; variant hepatic nuclear factor |
|  | HNF1B |
|  | variant hepatic nuclear factor |
|  | AI987804 |
|  | Variant hepatic nuclear factor 1 |
|  | Hnf1beta |
|  | LF-B3 |
|  | MGC93549 |
|  | HNF1beta |
|  | AI385728 |
|  | LFB3 |
|  | Homeoprotein LFB3 |
|  | transcription factor 2 |


---

|  |  |
| --- | --- |
| GO Component | transcription factor complex |
|  | nucleus |


---

|  |  |
| --- | --- |
| GO ID | GO:0009952 |
|  | GO:0005634 |
|  | GO:0003677 |
|  | GO:0045941 |
|  | GO:0006355 |
|  | GO:0046982 |
|  | GO:0016563 |
|  | GO:0045893 |
|  | GO:0006350 |
|  | GO:0045944 |
|  | GO:0005667 |
|  | GO:0003700 |
|  | GO:0045449 |
|  | GO:0030902 |


---

|  |  |
| --- | --- |
| MIM | MIM:137920 |
|  | MIM:189907 |
|  | MIM:604284 |
|  | MIM:125853 |


---

|  |  |
| --- | --- |
| Connectivity | 138 |


---

|  |  |
| --- | --- |
| Entrez ID | 21410 |
|  | 25640 |
|  | 6928 |


---

|  |  |
| --- | --- |
| Agilent ID | A\_24\_P330822 |
|  | A\_52\_P177699 |
|  | A\_14\_P138396 |
|  | A\_14\_P104045 |
|  | A\_42\_P668858 |
|  | A\_43\_P11714 |
|  | A\_23\_P409287 |
|  | A\_53\_P133162 |
|  | A\_51\_P484311 |
|  | A\_23\_P207557 |


---

|  |  |
| --- | --- |
| Cellular Localization | Nucleus |
|  | Organelle |
|  | Cell |


---

|  |  |
| --- | --- |
| Pathway | Zn def RIN |
|  | Master Regulators |
|  | Zn def DIN |


---

|  |  |
| --- | --- |
| GO Process | positive regulation of transcription, DNA-dependent |
|  | positive regulation of transcription |
|  | hindbrain development |
|  | regulation of transcription, DNA-dependent |
|  | transcription |
|  | regulation of transcription |
|  | anterior/posterior pattern formation |
|  | positive regulation of transcription from RNA polymerase II promoter |


---

|  |  |
| --- | --- |
| UniGene | Hs.191144 |
|  | Rn.11342 |
|  | Mm.7226 |


---

|  |  |
| --- | --- |
| Affymetrix Probeset ID | 101396\_at |
|  | 1369682\_at |
|  | 1421224\_a\_at |
|  | 1441484\_at |
|  | 1451687\_a\_at |
|  | 162159\_i\_at |
|  | 205313\_at |
|  | 208135\_at |
|  | 240935\_at |
|  | 33621\_at |
|  | 38506\_at |
|  | 85744\_at |
|  | 88949\_at |
|  | AA733226\_at |
|  | g4507396\_3p\_at |
|  | g6031204\_3p\_at |
|  | X55842\_s\_at |
|  | X56546\_at |
|  | X71348\_at |
|  | 100160\_at |


---

|  |  |
| --- | --- |
| GO Function | transcriptional activator activity |
|  | transcription factor activity |
|  | protein heterodimerization activity |
|  | DNA binding |


---

|  |  |
| --- | --- |
| Nucleotide | BC081826 |
|  | X56546 |
|  | AL669868 |
|  | AB008175 |
|  | NM\_006481 |
|  | X71348 |
|  | BC025189 |
|  | AB008177 |
|  | X55842 |
|  | AK004837 |
|  | AB008176 |
|  | AB052659 |
|  | BC017714 |
|  | BT007126 |
|  | NM\_013103 |
|  | AB026732 |
|  | X58840 |
|  | AB008174 |
|  | NM\_000458 |
|  | U90287 |
|  | AK019258 |
|  | CR536572 |
|  | NM\_009330 |


---

|  |  |
| --- | --- |
| Protein | P27889 |
|  | CAI25051 |
|  | BAA77719 |
|  | NP\_033356 |
|  | CAB59223 |
|  | NP\_006472 |
|  | BAA77718 |
|  | CAI35313 |
|  | NP\_000449 |
|  | CAG38809 |
|  | CAI35312 |
|  | AAC63388 |
|  | AAH25189 |
|  | AAH17714 |
|  | BAA77721 |
|  | BAB31632 |
|  | AAH81826 |
|  | NP\_037235 |
|  | BAB23604 |
|  | CAA39886 |
|  | P35680 |
|  | CAI25052 |
|  | BAA77720 |
|  | CAA39358 |
|  | AAP35790 |
|  | CAA41652 |
|  | BAB60814 |
|  | P23899 |


---

|  |  |
| --- | --- |
| Organism | Mammal |


---

|  |  |
| --- | --- |
| Location | chromosome 10, 10q26 (Rattus norvegicus) |
|  | chromosome 17, 17cen-q21.3 (Homo sapiens) |
|  | chromosome 11, 11 44.0 cM, 11 C (Mus musculus) |
|  | 11 44.0 cM (Mus musculus) |


---

|  |  |
| --- | --- |
