# Supplementary material for: Dynamic transcriptomic profiles of zebrafish gills in response to zinc depletion
Source: BMC Genomics. 2010 Oct 8;11:548. doi: 10.1186/1471-2164-11-548 (PMC3091697; doi:10.1186/1471-2164-11-548)
Supplement: Additional file 2 — Figure S1 - Interactive Direct Interaction Network of responses to zinc depletion. Mini web-site containing index.html and hyperlinked pages in subdirectory. The web site is an interactive version of Figure 6A containing curated interactions between regulated genes and respective proteins. Legend: Molecular interactions between zinc and proteins encoded by genes changed under zinc depletion. A Direct Interaction Network was created based on curated interactions contained within the PathwayArchitect database and provided through hyperlinks. Red ovals represent proteins and the blue circle symbolizes Zn(II). Dark blue squares denote 'binding', and light blue squares 'expression'; green squares stand for 'regulation', green diamonds for 'metabolism', and green circles for 'promoter binding'. Arrow heads indicate directionality of the interaction where annotated. [file 1471-2164-11-548-S2.ZIP › PathwayArchitect Zn def DIN2/107463.html]

# PROTEIN: NCOA5

|  |  |
| --- | --- |
| Name | NCOA5 |
| Type | PROTEIN |
| Description | nuclear receptor coactivator 5 |
| Note | This gene encodes a coregulator for the alpha and beta estrogen receptors and the orphan nuclear receptor NR1D2. The protein localizes to the nucleus, and is thought to have both coactivator and corepressor functions. Its interaction with nuclear receptors is independent of the AF2 domain on the receptors, which is known to regulate interaction with other coreceptors. Two alternatively spliced transcript variants for this gene have been described. However, the full length nature of one of the variants has not been determined. |
| Alias | NCoA-5 |
|  | CIA |
|  | NCOA5 |
|  | bA465L10.6 |
|  | Ncoa5 |
|  | MGC28864 |
|  | coactivator independent of AF-2 |
|  | Coactivator independent of AF-2 |
|  | KIAA1637 |


---

|  |  |
| --- | --- |
| GO Component | nucleus |


---

|  |  |
| --- | --- |
| GO ID | GO:0005634 |
|  | GO:0005524 |
|  | GO:0006355 |
|  | GO:0006350 |
|  | GO:0006412 |
|  | GO:0004812 |


---

|  |  |
| --- | --- |
| Connectivity | 29 |


---

|  |  |
| --- | --- |
| Entrez ID | 228869 |
|  | 57727 |


---

|  |  |
| --- | --- |
| Agilent ID | A\_51\_P180862 |
|  | A\_52\_P223080 |
|  | A\_14\_P116340 |
|  | A\_14\_P138273 |
|  | A\_23\_P210515 |
|  | A\_53\_P117989 |
|  | A\_53\_P160403 |
|  | A\_23\_P315843 |


---

|  |  |
| --- | --- |
| Cellular Localization | Nucleus |
|  | Organelle |
|  | Cell |


---

|  |  |
| --- | --- |
| Pathway | Zn def RIN |
|  | Master Regulators |
|  | Zn def DIN |


---

|  |  |
| --- | --- |
| GO Process | regulation of transcription, DNA-dependent |
|  | transcription |
|  | protein biosynthesis |


---

|  |  |
| --- | --- |
| UniGene | Hs.25669 |
|  | Mm.233080 |


---

|  |  |
| --- | --- |
| Affymetrix Probeset ID | 1424055\_at |
|  | 1456997\_at |
|  | 225145\_at |
|  | 229583\_at |
|  | 234471\_s\_at |
|  | 239815\_3p\_at |
|  | 239815\_at |
|  | 43758\_at |
|  | 43950\_at |
|  | 51099\_s\_at |
|  | 53207\_at |
|  | 55003\_at |
|  | 87531\_at |
|  | 97966\_at |
|  | AA267869\_s\_at |
|  | Hs.190075.0.A1\_3p\_at |
|  | Hs.190075.0.A1\_3p\_x\_at |
|  | Hs.25669.0.A1\_3p\_at |
|  | Hs.288140.0.S1\_3p\_at |
|  | Hs.288140.1.S1\_3p\_a\_at |
|  | AA479996\_at |
|  | H43245\_at |
|  | RC\_AA262750\_at |
|  | RC\_AA357964\_at |
|  | RC\_AA454115\_s\_at |
|  | TC36757\_at |


---

|  |  |
| --- | --- |
| GO Function | tRNA ligase activity |
|  | ATP binding |


---

|  |  |
| --- | --- |
| Nucleotide | NM\_020967 |
|  | AK049242 |
|  | AI848209 |
|  | AF470686 |
|  | AL162458 |
|  | NM\_144892 |
|  | AB046857 |
|  | AK173226 |
|  | AL035662 |
|  | BC022624 |
|  | BC056872 |
|  | BC017152 |
|  | AF230533 |


---

|  |  |
| --- | --- |
| Protein | CAI42972 |
|  | AAH17152 |
|  | AAH56872 |
|  | Q91W39 |
|  | CAH74052 |
|  | AAO33457 |
|  | BAD32504 |
|  | AAG36793 |
|  | Q9HCD5 |
|  | NP\_659141 |
|  | BAC33631 |
|  | BAB13463 |
|  | NP\_066018 |
|  | CAI42971 |


---

|  |  |
| --- | --- |
| Organism | Mammal |


---

|  |  |
| --- | --- |
| Location | chromosome 20, 20q12-q13.12 (Homo sapiens) |
|  | chromosome 2, 2 H3 (Mus musculus) |


---

|  |  |
| --- | --- |
