# Supplementary material for: Dynamic transcriptomic profiles of zebrafish gills in response to zinc depletion
Source: BMC Genomics. 2010 Oct 8;11:548. doi: 10.1186/1471-2164-11-548 (PMC3091697; doi:10.1186/1471-2164-11-548)
Supplement: Additional file 2 — Figure S1 - Interactive Direct Interaction Network of responses to zinc depletion. Mini web-site containing index.html and hyperlinked pages in subdirectory. The web site is an interactive version of Figure 6A containing curated interactions between regulated genes and respective proteins. Legend: Molecular interactions between zinc and proteins encoded by genes changed under zinc depletion. A Direct Interaction Network was created based on curated interactions contained within the PathwayArchitect database and provided through hyperlinks. Red ovals represent proteins and the blue circle symbolizes Zn(II). Dark blue squares denote 'binding', and light blue squares 'expression'; green squares stand for 'regulation', green diamonds for 'metabolism', and green circles for 'promoter binding'. Arrow heads indicate directionality of the interaction where annotated. [file 1471-2164-11-548-S2.ZIP › PathwayArchitect Zn def DIN2/107866.html]

# PROTEIN: TCF4

|  |  |
| --- | --- |
| Name | TCF4 |
| Type | PROTEIN |
| Description | transcription factor 4 |
| Note | TCF4 encodes transcription factor 4, a basic helix-turn-helix transcription factor. The protein recognizes an Ephrussi-box ('E-box') binding site ('CANNTG') - a motif first identified in immunoglobulin enhancers. TCF4 is expressed predominantly in pre-B-cells, although it is found in other tissues as well. TCF4 is known to produce multiple transcripts; however as the complete structure is only known for the transcript that encodes the b isoform, that is the variant presented here. |
| Alias | SEF2 |
|  | Sef2 |
|  | ITF2 |
|  | SEF-2 |
|  | Tcf-4 |
|  | ASP-I2 |
|  | RITF-2 |
|  | SL3-3 enhancer factor 2 |
|  | Class A helix-loop-helix transcription factor ME2 |
|  | R8f DNA-binding protein |
|  | SEF2-1A |
|  | ITF-2 |
|  | MITF-2B |
|  | MITF-2 |
|  | SEF2-1B |
|  | Hnf-4 |
|  | E2.2 |
|  | immunoglobulin transcription factor-2 |
|  | Tcf4 |
|  | TFE |
|  | transcription factor-4 (immunoglobulin transcription factor-2) |
|  | ME2 |
|  | ITF-2b |
|  | SEF2-1 |
|  | MITF-2A |
|  | TCF4 |
|  | 5730422P05Rik |
|  | Fragment |
|  | E2-2 |
|  | Itf2 |
|  | Immunoglobulin transcription factor 2 |


---

|  |  |
| --- | --- |
| GO Component | transcription factor complex |
|  | nucleus |


---

|  |  |
| --- | --- |
| GO ID | GO:0003677 |
|  | GO:0003714 |
|  | GO:0006355 |
|  | GO:0030528 |
|  | GO:0003690 |
|  | GO:0006357 |
|  | GO:0005515 |
|  | GO:0005667 |
|  | GO:0003700 |
|  | GO:0005634 |
|  | GO:0003702 |
|  | GO:0007275 |
|  | GO:0045944 |
|  | GO:0006350 |
|  | GO:0045449 |
|  | GO:0008013 |


---

|  |  |
| --- | --- |
| MIM | MIM:602272 |


---

|  |  |
| --- | --- |
| Connectivity | 231 |


---

|  |  |
| --- | --- |
| Entrez ID | 21413 |
|  | 6925 |
|  | 84382 |


---

|  |  |
| --- | --- |
| Agilent ID | A\_23\_P27332 |
|  | A\_14\_P136688 |
|  | A\_53\_P128196 |
|  | A\_52\_P521101 |
|  | A\_42\_P524370 |
|  | A\_51\_P265137 |
|  | A\_52\_P424847 |
|  | A\_53\_P143287 |
|  | A\_53\_P129982 |
|  | A\_14\_P125347 |
|  | A\_14\_P120853 |
|  | A\_14\_P132228 |
|  | A\_14\_P104100 |


---

|  |  |
| --- | --- |
| Cellular Localization | Nucleus |
|  | Organelle |
|  | Cell |


---

|  |  |
| --- | --- |
| Pathway | Wnt Signaling (Calcium) |
|  | Zn def RIN |
|  | Master Regulators |
|  | Zn def DIN |


---

|  |  |
| --- | --- |
| GO Process | development |
|  | regulation of transcription, DNA-dependent |
|  | regulation of transcription from RNA polymerase II promoter |
|  | transcription |
|  | regulation of transcription |
|  | positive regulation of transcription from RNA polymerase II promoter |


---

|  |  |
| --- | --- |
| UniGene | Mm.4269 |
|  | Rn.23354 |
|  | Hs.200285 |


---

|  |  |
| --- | --- |
| Affymetrix Probeset ID | 111083\_at |
|  | 112795\_at |
|  | 1368841\_at |
|  | 1368842\_at |
|  | 1391992\_at |
|  | 1396157\_at |
|  | 1396516\_at |
|  | 1396660\_at |
|  | 1397286\_at |
|  | 1416723\_at |
|  | 1416724\_x\_at |
|  | 1416725\_at |
|  | 1424089\_a\_at |
|  | 1434148\_at |
|  | 1434149\_at |
|  | 1439336\_at |
|  | 1440106\_at |
|  | 1446386\_at |
|  | 1458201\_at |
|  | 160483\_at |
|  | 203753\_at |
|  | 212382\_at |
|  | 212385\_at |
|  | 212386\_at |
|  | 212387\_at |
|  | 213891\_s\_at |
|  | 215164\_at |
|  | 222146\_s\_at |
|  | 228837\_at |
|  | 32872\_at |
|  | 36605\_at |
|  | 1396577\_at |
|  | 53331\_at |
|  | 56694\_at |
|  | 56764\_at |
|  | 56847\_at |
|  | 1391179\_at |
|  | 68263\_at |
|  | 68265\_g\_at |
|  | 85843\_at |
|  | 89997\_at |
|  | g4507398\_3p\_a\_at |
|  | g4507398\_3p\_x\_at |
|  | Hs.106932.0.A1\_3p\_at |
|  | 1373734\_at |
|  | Hs.289068.0.S1\_3p\_at |
|  | Hs.289068.0.S2\_3p\_at |
|  | Hs.289068.0.S3\_3p\_at |
|  | Hs.289068.0.S4\_3p\_a\_at |
|  | Hs.289068.0.S4\_3p\_x\_at |
|  | Hs.289068.1.S1\_3p\_a\_at |
|  | Hs.289068.1.S1\_3p\_x\_at |
|  | M74719\_at |
|  | rc\_AA849929\_at |
|  | rc\_AI008415\_at |
|  | rc\_AI030087\_at |
|  | rc\_AI069987\_at |
|  | rc\_AI639517\_at |
|  | U09228\_at |
|  | u16322\_s\_at |
|  | 1446953\_at |
|  | 237749\_at |
|  | 240321\_at |
|  | 244480\_at |
|  | 74751\_at |
|  | Hs.127037.0.A1\_3p\_at |
|  | Hs.132277.0.A1\_3p\_at |
|  | Hs.252740.0.A1\_3p\_at |
|  | rc\_AA956941\_at |
|  | 67545\_at |
|  | 77098\_at |
|  | 99588\_at |
|  | RC\_AA287097\_at |
|  | RC\_AA290666\_at |
|  | RC\_N75542\_at |
|  | RC\_N93521\_at |
|  | RC\_T73792\_at |
|  | RC\_W69216\_at |
|  | TC17771\_at |
|  | TC29821\_at |
|  | TC38365\_at |
|  | TC39876\_r\_at |
|  | TC41740\_at |
|  | W76416\_at |
|  | rc\_AI101171\_at |


---

|  |  |
| --- | --- |
| GO Function | double-stranded DNA binding |
|  | protein binding |
|  | beta-catenin binding |
|  | RNA polymerase II transcription factor activity |
|  | transcription factor activity |
|  | transcription regulator activity |
|  | transcription corepressor activity |
|  | DNA binding |


---

|  |  |
| --- | --- |
| Nucleotide | AK017601 |
|  | BC031056 |
|  | AK081012 |
|  | AB209741 |
|  | AK051958 |
|  | S75870 |
|  | AK032345 |
|  | AK026674 |
|  | U75701 |
|  | AK045400 |
|  | AK145285 |
|  | AK014343 |
|  | X52079 |
|  | AK021120 |
|  | AK132140 |
|  | M74718 |
|  | AK052198 |
|  | AK078780 |
|  | BC014293 |
|  | X91753 |
|  | AK017365 |
|  | AK151433 |
|  | AK095041 |
|  | AK133885 |
|  | M74719 |
|  | AK089036 |
|  | AF149284 |
|  | AK040346 |
|  | NM\_013685 |
|  | U16322 |
|  | AK028748 |
|  | AK021032 |
|  | AK096862 |
|  | NM\_053369 |
|  | AK083152 |
|  | BC043050 |
|  | AK047926 |
|  | U09228 |
|  | AK122765 |
|  | U16321 |
|  | AK039181 |
|  | NM\_003199 |
|  | AK081147 |


---

|  |  |
| --- | --- |
| Protein | P15884 |
|  | AAC52415 |
|  | BAC34821 |
|  | AAH43050 |
|  | AAK26670 |
|  | AAA60310 |
|  | BAC26095 |
|  | AAA60311 |
|  | BAD92978 |
|  | AAB32662 |
|  | BAE21912 |
|  | NP\_038713 |
|  | AAH14293 |
|  | Q60722 |
|  | AAC52414 |
|  | Q62655 |
|  | AAC51824 |
|  | CAA36298 |
|  | BAE30396 |
|  | BAE26343 |
|  | AAA21122 |
|  | BAB29285 |
|  | BAC38116 |
|  | CAA62868 |
|  | NP\_445821 |
|  | NP\_003190 |


---

|  |  |
| --- | --- |
| Organism | Mammal |


---

|  |  |
| --- | --- |
| Location | chromosome 18 (Mus musculus) |
|  | chromosome 18, 18q21.1 (Homo sapiens) |
|  | chromosome 18, 18q12.1 (Rattus norvegicus) |


---

|  |  |
| --- | --- |
