# Supplementary material for: Dynamic transcriptomic profiles of zebrafish gills in response to zinc depletion
Source: BMC Genomics. 2010 Oct 8;11:548. doi: 10.1186/1471-2164-11-548 (PMC3091697; doi:10.1186/1471-2164-11-548)
Supplement: Additional file 2 — Figure S1 - Interactive Direct Interaction Network of responses to zinc depletion. Mini web-site containing index.html and hyperlinked pages in subdirectory. The web site is an interactive version of Figure 6A containing curated interactions between regulated genes and respective proteins. Legend: Molecular interactions between zinc and proteins encoded by genes changed under zinc depletion. A Direct Interaction Network was created based on curated interactions contained within the PathwayArchitect database and provided through hyperlinks. Red ovals represent proteins and the blue circle symbolizes Zn(II). Dark blue squares denote 'binding', and light blue squares 'expression'; green squares stand for 'regulation', green diamonds for 'metabolism', and green circles for 'promoter binding'. Arrow heads indicate directionality of the interaction where annotated. [file 1471-2164-11-548-S2.ZIP › PathwayArchitect Zn def DIN2/108806.html]

# PROTEIN: INCENP

|  |  |
| --- | --- |
| Name | INCENP |
| Type | PROTEIN |
| Description | inner centromere protein antigens 135/155kDa |
| Note | In mammalian cells, 2 broad groups of centromere-interacting proteins have been described: constitutively binding centromere proteins and 'passenger,' or transiently interacting, proteins (reviewed by Choo, 1997). The constitutive proteins include CENPA (centromere protein A; MIM 117139), CENPB (MIM 117140), CENPC1 (MIM 117141), and CENPD (MIM 117142). The term 'passenger proteins' encompasses a broad collection of proteins that localize to the centromere during specific stages of the cell cycle (Earnshaw and Mackay, 1994). These include CENPE (MIM 117143); MCAK (MIM 604538); KID (MIM 603213); cytoplasmic dynein (e.g., MIM 600112); CliPs (e.g., MIM 179838); and CENPF/mitosin (MIM 600236). The inner centromere proteins (INCENPs) (Earnshaw and Cooke, 1991), the initial members of the passenger protein group, display a broad localization along chromosomes in the early stages of mitosis but gradually become concentrated at centromeres as the cell cycle progresses into mid-metaphase. During telophase, the proteins are located within the midbody in the intercellular bridge, where they are discarded after cytokinesis (Cutts et al., 1999).[supplied by OMIM] |
| Alias | inner centromere protein INCENP |
|  | C77457 |
|  | INCENP |
|  | AU019509 |
|  | 2700067E22Rik |
|  | Inner centromere protein |
|  | Incenp |
|  | C130081E20 |
|  | chromosomal passenger protein |
|  | binds and activates aurora-B and -C in vivo and in vitro |
|  | inner centromere protein antigens (135kD, 155kD) |
|  | MGC111393 |


---

|  |  |
| --- | --- |
| GO Component | centric heterochromatin |
|  | central element |
|  | synaptonemal complex |
|  | midbody |
|  | chromosome, pericentric region |
|  | microtubule |
|  | nucleus |
|  | spindle |


---

|  |  |
| --- | --- |
| GO ID | GO:0005634 |
|  | GO:0005874 |
|  | GO:0000775 |
|  | GO:0007067 |
|  | GO:0030496 |
|  | GO:0005515 |
|  | GO:0000801 |
|  | GO:0005721 |
|  | GO:0000069 |
|  | GO:0051301 |
|  | GO:0005819 |
|  | GO:0000795 |
|  | GO:0007049 |


---

|  |  |
| --- | --- |
| MIM | MIM:604411 |


---

|  |  |
| --- | --- |
| Connectivity | 45 |


---

|  |  |
| --- | --- |
| Entrez ID | 16319 |
|  | 3619 |


---

|  |  |
| --- | --- |
| Agilent ID | A\_14\_P201658 |
|  | A\_14\_P131263 |
|  | A\_53\_P166847 |
|  | A\_51\_P264064 |
|  | A\_53\_P151062 |
|  | A\_23\_P116387 |
|  | A\_53\_P155766 |
|  | A\_53\_P166632 |


---

|  |  |
| --- | --- |
| Cellular Localization | Nucleus |
|  | Microtubule |
|  | Cytoskeleton |
|  | Chromosome |
|  | Cell |
|  | Organelle |


---

|  |  |
| --- | --- |
| Pathway | Master Regulators |
|  | Zn def RIN |
|  | Zn xs inventory |
|  | Zn xs DIN |
|  | Zn def DIN |


---

|  |  |
| --- | --- |
| GO Process | mitosis |
|  | centromere and kinetochore complex maturation |
|  | cell cycle |
|  | cell division |


---

|  |  |
| --- | --- |
| UniGene | Mm.29755 |
|  | Hs.142179 |


---

|  |  |
| --- | --- |
| Affymetrix Probeset ID | 104468\_at |
|  | 113149\_at |
|  | 1423092\_at |
|  | 1423093\_at |
|  | 1439252\_at |
|  | 1439436\_x\_at |
|  | 1441314\_at |
|  | 1566043\_at |
|  | 1566044\_at |
|  | 164272\_at |
|  | 166306\_at |
|  | 167338\_i\_at |
|  | 170509\_at |
|  | 219769\_at |
|  | 242787\_at |
|  | 244862\_at |
|  | 43768\_at |
|  | 76984\_at |
|  | 88327\_at |
|  | 93758\_at |
|  | aa014535\_s\_at |
|  | aa139030\_s\_at |
|  | g9910375\_3p\_at |
|  | Hs.142179.0.A1\_3p\_at |
|  | Hs2.384653.1.S1\_3p\_at |
|  | 140409\_f\_at |
|  | 128626\_f\_at |
|  | 112046\_at |
|  | 60489\_r\_at |
|  | 85405\_at |
|  | RC\_AA132545\_at |
|  | RC\_AA149634\_at |
|  | TC40529\_at |


---

|  |  |
| --- | --- |
| GO Function | protein binding |


---

|  |  |
| --- | --- |
| Nucleotide | BC021761 |
|  | BC052414 |
|  | AK081841 |
|  | AB100433 |
|  | AF282265 |
|  | AK034764 |
|  | AF117610 |
|  | BC098576 |
|  | NM\_016692 |
|  | AK088627 |
|  | NM\_020238 |
|  | AK056195 |
|  | AB100432 |
|  | AY714053 |
|  | BC032678 |
|  | AA823653 |
|  | BC037011 |
|  | AK012497 |
|  | AF116187 |


---

|  |  |
| --- | --- |
| Protein | AAU04398 |
|  | AAD26202 |
|  | AAF87584 |
|  | BAC55880 |
|  | AAD32094 |
|  | NP\_064623 |
|  | Q9WU62 |
|  | AAH98576 |
|  | BAC40462 |
|  | BAC38346 |
|  | AAH37011 |
|  | Q9NQS7 |
|  | NP\_057901 |
|  | BAC55879 |
|  | AAH52414 |


---

|  |  |
| --- | --- |
| Organism | Mammal |


---

|  |  |
| --- | --- |
| Location | chromosome 11, 11q12-q13 (Homo sapiens) |
|  | chromosome 19, 19 0.0 cM, 19 A (Mus musculus) |
|  | 19 0.0 cM (Mus musculus) |


---

|  |  |
| --- | --- |
