# Supplementary material for: Dynamic transcriptomic profiles of zebrafish gills in response to zinc depletion
Source: BMC Genomics. 2010 Oct 8;11:548. doi: 10.1186/1471-2164-11-548 (PMC3091697; doi:10.1186/1471-2164-11-548)
Supplement: Additional file 2 — Figure S1 - Interactive Direct Interaction Network of responses to zinc depletion. Mini web-site containing index.html and hyperlinked pages in subdirectory. The web site is an interactive version of Figure 6A containing curated interactions between regulated genes and respective proteins. Legend: Molecular interactions between zinc and proteins encoded by genes changed under zinc depletion. A Direct Interaction Network was created based on curated interactions contained within the PathwayArchitect database and provided through hyperlinks. Red ovals represent proteins and the blue circle symbolizes Zn(II). Dark blue squares denote 'binding', and light blue squares 'expression'; green squares stand for 'regulation', green diamonds for 'metabolism', and green circles for 'promoter binding'. Arrow heads indicate directionality of the interaction where annotated. [file 1471-2164-11-548-S2.ZIP › PathwayArchitect Zn def DIN2/110868.html]

# PROTEIN: RAB4B

|  |  |
| --- | --- |
| Name | RAB4B |
| Type | PROTEIN |
| Description | RAB4B, member RAS oncogene family |
| Note | May play a role in regulation of membrane trafficking [RGD] |
| Alias | small GTP binding protein RAB4B |
|  | Ras-related protein Rab-4B |
|  | Rab4b |
|  | ras-related GTP-binding protein 4b |
|  | 1500031G17Rik |
|  | MGC52123 |
|  | RAB4B |


---

|  |  |
| --- | --- |
| GO Component | intracellular |


---

|  |  |
| --- | --- |
| GO ID | GO:0006810 |
|  | GO:0008565 |
|  | GO:0005622 |
|  | GO:0016192 |
|  | GO:0003924 |
|  | GO:0006886 |
|  | GO:0000166 |
|  | GO:0007264 |
|  | GO:0005525 |
|  | GO:0015031 |


---

|  |  |
| --- | --- |
| Connectivity | 8 |


---

|  |  |
| --- | --- |
| Entrez ID | 53916 |
|  | 19342 |
|  | 50866 |


---

|  |  |
| --- | --- |
| Agilent ID | A\_14\_P137410 |
|  | A\_51\_P258620 |
|  | A\_14\_P109886 |
|  | A\_32\_P19135 |
|  | A\_23\_P16119 |
|  | A\_42\_P634910 |
|  | A\_23\_P16116 |
|  | A\_14\_P126025 |


---

|  |  |
| --- | --- |
| Cellular Localization | Cell |


---

|  |  |
| --- | --- |
| Pathway | Master Regulators |
|  | Zn def RIN |
|  | Zn def DIN |


---

|  |  |
| --- | --- |
| GO Process | intracellular protein transport |
|  | transport |
|  | vesicle-mediated transport |
|  | protein transport |
|  | small GTPase mediated signal transduction |


---

|  |  |
| --- | --- |
| UniGene | Hs.25367 |
|  | Rn.7181 |
|  | Mm.262447 |


---

|  |  |
| --- | --- |
| Affymetrix Probeset ID | 1367955\_at |
|  | 1451643\_a\_at |
|  | 219807\_x\_at |
|  | 228521\_s\_at |
|  | 233385\_x\_at |
|  | 237257\_at |
|  | 39272\_g\_at |
|  | 53177\_at |
|  | 61358\_at |
|  | 91126\_at |
|  | 91128\_g\_at |
|  | 99505\_at |
|  | AFFX-hum\_alu\_at |
|  | g7706672\_3p\_s\_at |
|  | Hs.25367.0.A1\_3p\_at |
|  | Hs.279771.1.A1\_3p\_s\_at |
|  | hum\_alu\_at |
|  | Msa.17007.0\_f\_at |
|  | Msa.5416.0\_s\_at |
|  | rc\_AA818114\_at |
|  | X78605\_at |
|  | RC\_AA496001\_at |
|  | RC\_H26742\_at |
|  | TC31131\_at |


---

|  |  |
| --- | --- |
| GO Function | protein transporter activity |
|  | GTP binding |
|  | GTPase activity |
|  | nucleotide binding |


---

|  |  |
| --- | --- |
| Nucleotide | BC007147 |
|  | AK024845 |
|  | NG\_000008 |
|  | BC096654 |
|  | AF408432 |
|  | AK005314 |
|  | AF498935 |
|  | AF165522 |
|  | AF087861 |
|  | AF217985 |
|  | AK170552 |
|  | NM\_029391 |
|  | X78605 |
|  | BC046927 |
|  | NM\_017355 |
|  | NM\_016154 |
|  | AK140513 |


---

|  |  |
| --- | --- |
| Protein | AAM21083 |
|  | AAG17228 |
|  | AAH07147 |
|  | Q91ZR1 |
|  | NP\_059051 |
|  | BAB23948 |
|  | AAL11725 |
|  | P51146 |
|  | BAE41875 |
|  | CAA55339 |
|  | AAP97171 |
|  | P61018 |
|  | NP\_083667 |
|  | NP\_057238 |
|  | AAD45923 |
|  | BAE24410 |
|  | AAH96654 |
|  | AAH46927 |


---

|  |  |
| --- | --- |
| Organism | Mammal |


---

|  |  |
| --- | --- |
| Location | chromosome 19, 19q13.2 (Homo sapiens) |
|  | chromosome 7, 7 7.4 cM, 7 A3 (Mus musculus) |
|  | chromosome 1, 1q21 (Rattus norvegicus) |
|  | 7 7.4 cM (Mus musculus) |


---

|  |  |
| --- | --- |
