# Supplementary material for: Dynamic transcriptomic profiles of zebrafish gills in response to zinc depletion
Source: BMC Genomics. 2010 Oct 8;11:548. doi: 10.1186/1471-2164-11-548 (PMC3091697; doi:10.1186/1471-2164-11-548)
Supplement: Additional file 2 — Figure S1 - Interactive Direct Interaction Network of responses to zinc depletion. Mini web-site containing index.html and hyperlinked pages in subdirectory. The web site is an interactive version of Figure 6A containing curated interactions between regulated genes and respective proteins. Legend: Molecular interactions between zinc and proteins encoded by genes changed under zinc depletion. A Direct Interaction Network was created based on curated interactions contained within the PathwayArchitect database and provided through hyperlinks. Red ovals represent proteins and the blue circle symbolizes Zn(II). Dark blue squares denote 'binding', and light blue squares 'expression'; green squares stand for 'regulation', green diamonds for 'metabolism', and green circles for 'promoter binding'. Arrow heads indicate directionality of the interaction where annotated. [file 1471-2164-11-548-S2.ZIP › PathwayArchitect Zn def DIN2/111197.html]

# PROTEIN: GIF

|  |  |
| --- | --- |
| Name | GIF |
| Type | PROTEIN |
| Description | gastric intrinsic factor (vitamin B synthesis) |
| Note | This gene is a member of the cobalamin transport protein family. It encodes a glycoprotein secreted by parietal cells of the gastric mucosa and is required for adequate absorption of vitamin B12. Vitamin B12 is necessary for erythrocyte maturation and mutations in this gene may lead to congenital pernicious anemia. |
| Alias | IFMH |
|  | INF |
|  | IF |
|  | AV073125 |
|  | Intrinsic factor |
|  | Gif |


---

|  |  |
| --- | --- |
| GO Component | extracellular space |


---

|  |  |
| --- | --- |
| GO ID | GO:0006810 |
|  | GO:0015087 |
|  | GO:0005615 |
|  | GO:0019842 |
|  | GO:0006811 |
|  | GO:0005488 |
|  | GO:0050897 |
|  | GO:0006824 |


---

|  |  |
| --- | --- |
| MIM | MIM:261000 |
|  | MIM:609342 |


---

|  |  |
| --- | --- |
| Connectivity | 269 |


---

|  |  |
| --- | --- |
| Entrez ID | 2694 |
|  | 14603 |
|  | 29319 |


---

|  |  |
| --- | --- |
| Agilent ID | A\_23\_P52974 |
|  | A\_51\_P280697 |
|  | A\_14\_P139562 |
|  | A\_53\_P139175 |
|  | A\_42\_P624212 |
|  | A\_53\_P164713 |


---

|  |  |
| --- | --- |
| Cellular Localization | Extracellular region |


---

|  |  |
| --- | --- |
| Pathway | Zn def RIN |
|  | Master Regulators |
|  | Zn def DIN |


---

|  |  |
| --- | --- |
| GO Process | transport |
|  | cobalt ion transport |
|  | ion transport |


---

|  |  |
| --- | --- |
| UniGene | Rn.34903 |
|  | Mm.456 |
|  | Hs.110014 |


---

|  |  |
| --- | --- |
| Affymetrix Probeset ID | 1387595\_at |
|  | 1419020\_at |
|  | 207033\_at |
|  | 32470\_at |
|  | 92690\_at |
|  | D45199cds\_s\_at |
|  | g4826743\_3p\_at |
|  | J03577\_at |
|  | L24191\_s\_at |
|  | M63154\_at |


---

|  |  |
| --- | --- |
| GO Function | cobalt ion transporter activity |
|  | binding |
|  | cobalt ion binding |
|  | vitamin binding |


---

|  |  |
| --- | --- |
| Nucleotide | J03577 |
|  | AK078933 |
|  | L24191 |
|  | NM\_008118 |
|  | L24192 |
|  | X76562 |
|  | NM\_005142 |
|  | BC037958 |
|  | D45200 |
|  | NM\_017162 |


---

|  |  |
| --- | --- |
| Protein | AAA37881 |
|  | BAC37468 |
|  | NP\_058858 |
|  | CAA54061 |
|  | AAA37882 |
|  | NP\_005133 |
|  | BAA08140 |
|  | AAH37958 |
|  | P52787 |
|  | P27352 |
|  | P17267 |
|  | NP\_032144 |
|  | AAA41361 |


---

|  |  |
| --- | --- |
| Organism | Mammal |


---

|  |  |
| --- | --- |
| Location | chromosome 11, 11q13 (Homo sapiens) |
|  | chromosome 19, 19 A (Mus musculus) |
|  | chromosome 1, 1q43 (Rattus norvegicus) |


---

|  |  |
| --- | --- |
