# Supplementary material for: Dynamic transcriptomic profiles of zebrafish gills in response to zinc depletion
Source: BMC Genomics. 2010 Oct 8;11:548. doi: 10.1186/1471-2164-11-548 (PMC3091697; doi:10.1186/1471-2164-11-548)
Supplement: Additional file 2 — Figure S1 - Interactive Direct Interaction Network of responses to zinc depletion. Mini web-site containing index.html and hyperlinked pages in subdirectory. The web site is an interactive version of Figure 6A containing curated interactions between regulated genes and respective proteins. Legend: Molecular interactions between zinc and proteins encoded by genes changed under zinc depletion. A Direct Interaction Network was created based on curated interactions contained within the PathwayArchitect database and provided through hyperlinks. Red ovals represent proteins and the blue circle symbolizes Zn(II). Dark blue squares denote 'binding', and light blue squares 'expression'; green squares stand for 'regulation', green diamonds for 'metabolism', and green circles for 'promoter binding'. Arrow heads indicate directionality of the interaction where annotated. [file 1471-2164-11-548-S2.ZIP › PathwayArchitect Zn def DIN2/1132536.html]

# BINDING:

|  |  |
| --- | --- |
| Type | BINDING |
| Effect | None |


---

|  |  |
| --- | --- |
| Score | 0 |


---

|  |  |
| --- | --- |
| Reference Count | 3 |


---

|  |  |
| --- | --- |
| Mechanism | Unknown |


---

|  |  |
| --- | --- |
| Reference:0 || Sentence | "The interactions of bovine brain MAP2 and tau, recombinant murine MAP2C, and recombinant human tau with phosphatidylinositol vesicles yield apparent Kd values of 51 +/- 6 nM, 2.4 +/- 0.6 microM, 1.4 +/- 0.1 microM, and 1.6 +/- 0.2 microM, respectively." |
| PMID | 8025110 |
| Year | 1994 |
| Species | Mouse |
|  | Human |
| Journal | Biochemistry |
| RefScore | 0 |
| Source | PArchNLP |
  |
|


---

|  |  |
| --- | --- |
 Reference:1 || Sentence | "A low-affinity site (Kd = 1.5-2.5 microM) is located within the C-terminal domain and affects the nonspecific interaction of MAP2, MAP2C, and tau with anionic phospholipids." |
| PMID | 8025110 |
| Year | 1994 |
| Species | Mouse |
|  | Human |
| Journal | Biochemistry |
| RefScore | 0 |
| Source | PArchNLP |
  ||


---

|  |  |
| --- | --- |
 Reference:2 || Sentence | "Recent evidence [Surridge and Burns, Biochemistry (1994) 33, 8051-8057] on the interaction of native and recombinant tau, recombinant MAP2c, and native MAP2 with vesicles prepared from phosphatidylinositol (PtdIns) and other phospholipids demonstrate that MAP2 differs from MAP2c and from tau in having a high-affinity PtdIns-binding site." |
| PMID | 7758753 |
| Year | 1995 |
| Species | Human |
|  | Mouse |
| Journal | Biochem Soc Trans |
| RefScore | 1 |
| Source | PArchNLP |
  |


---

|  |  |
| --- | --- |
