# Supplementary material for: Dynamic transcriptomic profiles of zebrafish gills in response to zinc depletion
Source: BMC Genomics. 2010 Oct 8;11:548. doi: 10.1186/1471-2164-11-548 (PMC3091697; doi:10.1186/1471-2164-11-548)
Supplement: Additional file 2 — Figure S1 - Interactive Direct Interaction Network of responses to zinc depletion. Mini web-site containing index.html and hyperlinked pages in subdirectory. The web site is an interactive version of Figure 6A containing curated interactions between regulated genes and respective proteins. Legend: Molecular interactions between zinc and proteins encoded by genes changed under zinc depletion. A Direct Interaction Network was created based on curated interactions contained within the PathwayArchitect database and provided through hyperlinks. Red ovals represent proteins and the blue circle symbolizes Zn(II). Dark blue squares denote 'binding', and light blue squares 'expression'; green squares stand for 'regulation', green diamonds for 'metabolism', and green circles for 'promoter binding'. Arrow heads indicate directionality of the interaction where annotated. [file 1471-2164-11-548-S2.ZIP › PathwayArchitect Zn def DIN2/118583.html]

# PROTEIN: HNF4A

|  |  |
| --- | --- |
| Name | HNF4A |
| Type | PROTEIN |
| Description | hepatocyte nuclear factor 4, alpha |
| Note | The protein encoded by this gene is a nuclear transcription factor which binds DNA as a homodimer. The encoded protein controls the expression of several genes, including hepatocyte nuclear factor 1 alpha, a transcription factor which regulates the expression of several hepatic genes. This gene may play a role in development of the liver, kidney, and intestines. Mutations in this gene have been associated with monogenic autosomal dominant non-insulin-dependent diabetes mellitus type I. Alternative splicing of this gene results in multiple transcript variants. |
| Alias | hepatic nuclear factor 4 alpha |
|  | TCF |
|  | Transcription factor 14 |
|  | Tcf14 |
|  | NR2A21 |
|  | hepatic nuclear factor 4 |
|  | Transcription factor HNF-4 |
|  | Nuclear receptor 2A1 |
|  | HNF4 |
|  | Nr2a1 |
|  | NR2A1 |
|  | D19Mgi10 |
|  | HNF4a9 |
|  | HNF-4 |
|  | HNF4-alpha |
|  | Hnf-4 |
|  | HNF4a7 |
|  | Tcf4 |
|  | Hnf4 |
|  | hepatic nuclear factor 4 (alpha transcription factor 4) |
|  | TCF14 |
|  | HNF-4-alpha |
|  | FLJ39654 |
|  | HNF4a8 |
|  | Hnf4a |
|  | HNF4A |
|  | HNF4 alpha |
|  | transcription factor-14 |
|  | MODY |
|  | hepatocyte nuclear factor 4 alpha |
|  | MODY1 |


---

|  |  |
| --- | --- |
| GO Component | transcription factor complex |
|  | nucleus |


---

|  |  |
| --- | --- |
| GO ID | GO:0003707 |
|  | GO:0003677 |
|  | GO:0007596 |
|  | GO:0006355 |
|  | GO:0006357 |
|  | GO:0005667 |
|  | GO:0003700 |
|  | GO:0005496 |
|  | GO:0005634 |
|  | GO:0004872 |
|  | GO:0003702 |
|  | GO:0004879 |
|  | GO:0046872 |
|  | GO:0045944 |
|  | GO:0006350 |
|  | GO:0006629 |


---

|  |  |
| --- | --- |
| MIM | MIM:600281 |
|  | MIM:125850 |
|  | MIM:125853 |


---

|  |  |
| --- | --- |
| Connectivity | 3137 |


---

|  |  |
| --- | --- |
| Entrez ID | 25735 |
|  | 15378 |
|  | 3172 |


---

|  |  |
| --- | --- |
| Agilent ID | A\_23\_P424096 |
|  | A\_53\_P177431 |
|  | A\_43\_P12146 |
|  | A\_43\_P16149 |
|  | A\_32\_P169688 |
|  | A\_52\_P69656 |
|  | A\_52\_P683991 |
|  | A\_23\_P28761 |
|  | A\_14\_P114317 |
|  | A\_51\_P198473 |
|  | A\_44\_P419188 |
|  | A\_32\_P783798 |
|  | A\_24\_P10751 |


---

|  |  |
| --- | --- |
| Cellular Localization | Nucleus |
|  | Organelle |
|  | Cell |


---

|  |  |
| --- | --- |
| Pathway | Zn def RIN |
|  | Master Regulators |
|  | Zn def DIN |


---

|  |  |
| --- | --- |
| GO Process | blood coagulation |
|  | lipid metabolism |
|  | regulation of transcription, DNA-dependent |
|  | regulation of transcription from RNA polymerase II promoter |
|  | transcription |
|  | positive regulation of transcription from RNA polymerase II promoter |


---

|  |  |
| --- | --- |
| UniGene | Rn.44442 |
|  | Hs.116462 |
|  | Mm.202383 |


---

|  |  |
| --- | --- |
| Affymetrix Probeset ID | 107590\_at |
|  | 1369289\_at |
|  | 1421983\_s\_at |
|  | 1427000\_at |
|  | 1427001\_s\_at |
|  | 1450447\_at |
|  | 208429\_x\_at |
|  | 214832\_at |
|  | 214851\_at |
|  | 216889\_s\_at |
|  | 230914\_at |
|  | 36721\_s\_at |
|  | 36722\_s\_at |
|  | 36723\_at |
|  | 1391485\_at |
|  | 74797\_at |
|  | 92713\_at |
|  | D10554\_s\_at |
|  | D29015\_s\_at |
|  | g4504442\_3p\_a\_at |
|  | Hs.54424.1.S1\_3p\_at |
|  | Hs.54424.2.A1\_3p\_at |
|  | Hs.54424.2.A1\_3p\_x\_at |
|  | Hs.54424.3.S1\_3p\_a\_at |
|  | Hs.54424.3.S1\_3p\_x\_at |
|  | X87870\_at |
|  | X87871\_s\_at |
|  | Z49825\_s\_at |
|  | 234774\_at |
|  | 233061\_at |
|  | 1563265\_at |
|  | X57133mRNA\_at |
|  | X57133mRNA\_g\_at |
|  | 47224\_at |
|  | 70611\_at |
|  | Hs.54424.2.S1\_3p\_at |
|  | Hs2.384043.1.S1\_3p\_at |
|  | RC\_T90794\_at |
|  | TC16125\_at |
|  | rc\_AI639043\_at |


---

|  |  |
| --- | --- |
| GO Function | ligand-dependent nuclear receptor activity |
|  | DNA binding |
|  | RNA polymerase II transcription factor activity |
|  | transcription factor activity |
|  | steroid hormone receptor activity |
|  | steroid binding |
|  | receptor activity |
|  | metal ion binding |


---

|  |  |
| --- | --- |
| Nucleotide | NM\_001030004 |
|  | AY680697 |
|  | NM\_178850 |
|  | D31720 |
|  | AF015275 |
|  | X87872 |
|  | X57133 |
|  | AY680696 |
|  | X76930 |
|  | NM\_178849 |
|  | AY680698 |
|  | AW935533 |
|  | U72967 |
|  | AK096973 |
|  | NM\_175914 |
|  | AK143948 |
|  | BC024551 |
|  | NM\_022180 |
|  | AF329936 |
|  | NM\_001030003 |
|  | BC039220 |
|  | Z49825 |
|  | D10554 |
|  | AL132772 |
|  | AF509467 |
|  | NM\_000457 |
|  | U72969 |
|  | CB163637 |
|  | AA710281 |
|  | D29015 |
|  | X87870 |
|  | AL117382 |
|  | X87871 |
|  | NM\_008261 |


---

|  |  |
| --- | --- |
| Protein | P41235 |
|  | NP\_000448 |
|  | CAA61133 |
|  | AAT91239 |
|  | P22449 |
|  | CAI23113 |
|  | AAT91238 |
|  | NP\_032287 |
|  | NP\_787110 |
|  | NP\_849180 |
|  | CAA54248 |
|  | NP\_849181 |
|  | NP\_071516 |
|  | CAA89989 |
|  | NP\_001025175 |
|  | BAA01411 |
|  | BAC04917 |
|  | CAA40412 |
|  | AAH39220 |
|  | AAT91237 |
|  | CAI18856 |
|  | CAI18857 |
|  | AAM34296 |
|  | CAC01303 |
|  | CAA61135 |
|  | P49698 |
|  | CAI18863 |
|  | AAB48083 |
|  | BAE25624 |
|  | AAC02906 |
|  | NP\_001025174 |
|  | BAA06101 |
|  | AAB48082 |
|  | AAK39433 |
|  | CAA61134 |


---

|  |  |
| --- | --- |
| Organism | Mammal |


---

|  |  |
| --- | --- |
| Location | chromosome 2, 2 94.0 cM, 2 H2-H3 (Mus musculus) |
|  | chromosome 20, 20q12-q13.1 (Homo sapiens) |
|  | 2 94.0 cM (Mus musculus) |
|  | chromosome 3 (Rattus norvegicus) |


---

|  |  |
| --- | --- |
