# Supplementary material for: Dynamic transcriptomic profiles of zebrafish gills in response to zinc depletion
Source: BMC Genomics. 2010 Oct 8;11:548. doi: 10.1186/1471-2164-11-548 (PMC3091697; doi:10.1186/1471-2164-11-548)
Supplement: Additional file 2 — Figure S1 - Interactive Direct Interaction Network of responses to zinc depletion. Mini web-site containing index.html and hyperlinked pages in subdirectory. The web site is an interactive version of Figure 6A containing curated interactions between regulated genes and respective proteins. Legend: Molecular interactions between zinc and proteins encoded by genes changed under zinc depletion. A Direct Interaction Network was created based on curated interactions contained within the PathwayArchitect database and provided through hyperlinks. Red ovals represent proteins and the blue circle symbolizes Zn(II). Dark blue squares denote 'binding', and light blue squares 'expression'; green squares stand for 'regulation', green diamonds for 'metabolism', and green circles for 'promoter binding'. Arrow heads indicate directionality of the interaction where annotated. [file 1471-2164-11-548-S2.ZIP › PathwayArchitect Zn def DIN2/119161.html]

# PROTEIN: TNNT2

|  |  |
| --- | --- |
| Name | TNNT2 |
| Type | PROTEIN |
| Description | troponin T2, cardiac |
| Note | The protein encoded by this gene is the tropomyosin-binding subunit of the troponin complex, which is located on the thin filament of striated muscles and regulates muscle contraction in response to alterations in intracellular calcium ion concentration. Mutations in this gene have been associated with familial hypertrophic cardiomyopathy as well as with dilated cardiomyopathy. Transcripts for this gene undergo alternative splicing that results in many tissue-specific isoforms, however, the full length nature of some of these variants has not yet been determined. |
| Alias | troponin T |
|  | cTnT |
|  | troponin T, cardiac muscle |
|  | CTTG |
|  | CMD1D |
|  | TnTc |
|  | CMH2 |
|  | TNNT2 |
|  | Ctt |
|  | Troponin T cardiac |
|  | cardiac TnT |
|  | Tnnt3 |
|  | troponin-T2, cardiac |
|  | cardiac troponin T |
|  | Cardiac muscle troponin T |
|  | TnTC |
|  | RATCTTG |
|  | Tnt |
|  | MGC3889 |
|  | Tnnt2 |


---

|  |  |
| --- | --- |
| GO Component | troponin complex |
|  | sarcomere |
|  | cytoplasm |


---

|  |  |
| --- | --- |
| GO ID | GO:0006936 |
|  | GO:0005200 |
|  | GO:0007517 |
|  | GO:0005737 |
|  | GO:0030017 |
|  | GO:0006937 |
|  | GO:0008016 |
|  | GO:0005861 |


---

|  |  |
| --- | --- |
| MIM | MIM:191045 |
|  | MIM:115195 |
|  | MIM:601494 |


---

|  |  |
| --- | --- |
| Connectivity | 203 |


---

|  |  |
| --- | --- |
| Entrez ID | 7139 |
|  | 24837 |
|  | 21956 |


---

|  |  |
| --- | --- |
| Agilent ID | A\_44\_P994686 |
|  | A\_53\_P146062 |
|  | A\_14\_P135734 |
|  | A\_53\_P150185 |
|  | A\_24\_P927304 |
|  | A\_23\_P34700 |
|  | A\_51\_P338262 |
|  | A\_24\_P257022 |
|  | A\_14\_P129845 |
|  | A\_53\_P111826 |
|  | A\_52\_P430110 |
|  | A\_42\_P542380 |


---

|  |  |
| --- | --- |
| Cellular Localization | Cytoplasm |
|  | Cytoskeleton |
|  | Cell |
|  | Organelle |


---

|  |  |
| --- | --- |
| Pathway | Zn def RIN |
|  | Zn xs inventory |
|  | Zn xs DIN |
|  | Zn xs RIN |
|  | Zn def DIN |


---

|  |  |
| --- | --- |
| GO Process | regulation of heart contraction rate |
|  | muscle contraction |
|  | regulation of muscle contraction |
|  | muscle development |


---

|  |  |
| --- | --- |
| UniGene | Hs.533613 |
|  | Rn.9965 |
|  | Mm.247470 |


---

|  |  |
| --- | --- |
| Affymetrix Probeset ID | 100593\_at |
|  | 1367592\_at |
|  | 1390061\_at |
|  | 1418726\_a\_at |
|  | 1424967\_x\_at |
|  | 1440424\_at |
|  | 1563655\_3p\_at |
|  | 1563655\_at |
|  | 171189\_r\_at |
|  | 215389\_s\_at |
|  | 38793\_at |
|  | AFFX-hum\_alu\_at |
|  | Hs2.407132.1.S1\_3p\_at |
|  | Hs2.407132.1.S1\_3p\_x\_at |
|  | Hs.296865.1.S1\_3p\_a\_at |
|  | hum\_alu\_at |
|  | l47599\_s\_at |
|  | M80829\_at |
|  | X74819\_at |
|  | rc\_AA924146\_at |


---

|  |  |
| --- | --- |
| GO Function | structural constituent of cytoskeleton |


---

|  |  |
| --- | --- |
| Nucleotide | Y09628 |
|  | NM\_012676 |
|  | S71128 |
|  | X79859 |
|  | L47599 |
|  | L47600 |
|  | AY160216 |
|  | AB052890 |
|  | AL832707 |
|  | L47570 |
|  | X83743 |
|  | AK164592 |
|  | AK146945 |
|  | X74819 |
|  | NM\_001001432 |
|  | L46872 |
|  | L47553 |
|  | Y09627 |
|  | L47549 |
|  | S64668 |
|  | L47550 |
|  | AY277394 |
|  | AK125236 |
|  | Y09626 |
|  | L47551 |
|  | AK168393 |
|  | BC063753 |
|  | L47552 |
|  | AY044273 |
|  | NM\_001001430 |
|  | NM\_011619 |
|  | X79856 |
|  | AK163786 |
|  | M26051 |
|  | AF004415 |
|  | M26052 |
|  | X79858 |
|  | M80829 |
|  | AK169061 |
|  | X79855 |
|  | L40162 |
|  | NM\_000364 |
|  | AK055533 |
|  | S71126 |
|  | BC002653 |
|  | NM\_001001431 |
|  | X79861 |
|  | AF004422 |


---

|  |  |
| --- | --- |
| Protein | CAA70840 |
|  | AAA85347 |
|  | AAA85351 |
|  | AAB07676 |
|  | BAE37494 |
|  | NP\_001001432 |
|  | AAB30957 |
|  | AAC39590 |
|  | P50753 |
|  | AAA85346 |
|  | AAH63753 |
|  | AAK92231 |
|  | BAB19881 |
|  | NP\_001001431 |
|  | AAA67422 |
|  | AAP96757 |
|  | CAA52818 |
|  | CAA70841 |
|  | BAC86093 |
|  | CAA56240 |
|  | AAA42297 |
|  | AAA85350 |
|  | AAB27731 |
|  | CAA56239 |
|  | AAB30956 |
|  | NP\_000355 |
|  | AAA42296 |
|  | AAA85348 |
|  | AAA85345 |
|  | AAN71651 |
|  | NP\_035749 |
|  | P50752 |
|  | NP\_036808 |
|  | AAH02653 |
|  | CAA56235 |
|  | AAA85349 |
|  | CAA56238 |
|  | BAE27554 |
|  | BAE40318 |
|  | P45379 |
|  | CAA70839 |
|  | CAA56236 |
|  | AAA85352 |
|  | NP\_001001430 |


---

|  |  |
| --- | --- |
| Organism | Mammal |


---

|  |  |
| --- | --- |
| Location | chromosome 1, 1 60.0 cM, 1 E4 (Mus musculus) |
|  | 1 60.0 cM (Mus musculus) |
|  | chromosome 13, 13q13 (Rattus norvegicus) |
|  | chromosome 1, 1q32 (Homo sapiens) |


---

|  |  |
| --- | --- |
