# Supplementary material for: Dynamic transcriptomic profiles of zebrafish gills in response to zinc depletion
Source: BMC Genomics. 2010 Oct 8;11:548. doi: 10.1186/1471-2164-11-548 (PMC3091697; doi:10.1186/1471-2164-11-548)
Supplement: Additional file 2 — Figure S1 - Interactive Direct Interaction Network of responses to zinc depletion. Mini web-site containing index.html and hyperlinked pages in subdirectory. The web site is an interactive version of Figure 6A containing curated interactions between regulated genes and respective proteins. Legend: Molecular interactions between zinc and proteins encoded by genes changed under zinc depletion. A Direct Interaction Network was created based on curated interactions contained within the PathwayArchitect database and provided through hyperlinks. Red ovals represent proteins and the blue circle symbolizes Zn(II). Dark blue squares denote 'binding', and light blue squares 'expression'; green squares stand for 'regulation', green diamonds for 'metabolism', and green circles for 'promoter binding'. Arrow heads indicate directionality of the interaction where annotated. [file 1471-2164-11-548-S2.ZIP › PathwayArchitect Zn def DIN2/120497.html]

# PROTEIN: FDX1

|  |  |
| --- | --- |
| Name | FDX1 |
| Type | PROTEIN |
| Description | ferredoxin 1 |
| Note | The product of this gene is a small iron-sulfur protein that transfers electrons from NADPH through ferredoxin reductase to a terminal cytochrome P450. This particular oxidation/reduction system is found in steroidogenic tissues, and is involved with the synthesis of bile acid and vitamin D. In addition to the expressed gene at this chromosomal locus (11q22), there are pseudogenes located on chromosomes 20 and 21. This gene product has been identified in a number of different tissues but all forms have been shown to be identical and are not tissue specific. |
| Alias | Adrenal ferredoxin |
|  | Ferredoxin 1 |
|  | ADRENODOXIN |
|  | adrenodoxin |
|  | Fdx1 |
|  | Hepatoredoxin |
|  | FDX1 |
|  | FDX |
|  | ADX |


---

|  |  |
| --- | --- |
| GO Component | mitochondrion |
|  | extracellular space |


---

|  |  |
| --- | --- |
| GO ID | GO:0009055 |
|  | GO:0006810 |
|  | GO:0020037 |
|  | GO:0051537 |
|  | GO:0005615 |
|  | GO:0006766 |
|  | GO:0005506 |
|  | GO:0005489 |
|  | GO:0006118 |
|  | GO:0051536 |
|  | GO:0008202 |
|  | GO:0046872 |
|  | GO:0005739 |


---

|  |  |
| --- | --- |
| MIM | MIM:103260 |


---

|  |  |
| --- | --- |
| Connectivity | 184 |


---

|  |  |
| --- | --- |
| Entrez ID | 14148 |
|  | 2230 |
|  | 29189 |


---

|  |  |
| --- | --- |
| Agilent ID | A\_23\_P98375 |
|  | A\_14\_P134766 |
|  | A\_52\_P582105 |
|  | A\_23\_P357780 |
|  | A\_42\_P624403 |
|  | A\_51\_P291129 |
|  | A\_53\_P120048 |
|  | A\_14\_P101166 |
|  | A\_51\_P291135 |
|  | A\_53\_P118013 |


---

|  |  |
| --- | --- |
| Cellular Localization | Mitochondrion |
|  | Extracellular region |
|  | Cytoplasm |
|  | Organelle |
|  | Cell |


---

|  |  |
| --- | --- |
| Pathway | Master Regulators |
|  | Zn def RIN |
|  | Zn def DIN |


---

|  |  |
| --- | --- |
| GO Process | transport |
|  | electron transport |
|  | vitamin metabolism |
|  | steroid metabolism |


---

|  |  |
| --- | --- |
| UniGene | Mm.1061 |
|  | Rn.6946 |
|  | Hs.744 |


---

|  |  |
| --- | --- |
| Affymetrix Probeset ID | 1368336\_at |
|  | 1389827\_at |
|  | 1449108\_at |
|  | 160062\_i\_at |
|  | 203646\_at |
|  | 203647\_s\_at |
|  | 239161\_at |
|  | 36893\_at |
|  | 45831\_r\_at |
|  | 92587\_at |
|  | D50436\_at |
|  | g13677224\_3p\_at |
|  | g182493\_3p\_a\_at |
|  | Hs.27295.0.A1\_3p\_at |
|  | l29123\_at |
|  | M23668\_at |
|  | Msa.1607.0\_f\_at |
|  | rc\_AI044488\_at |
|  | 92588\_at |
|  | RC\_AA250934\_s\_at |
|  | RC\_F02515\_at |
|  | TC26730\_at |


---

|  |  |
| --- | --- |
| GO Function | electron carrier activity |
|  | iron-sulfur cluster binding |
|  | 2 iron, 2 sulfur cluster binding |
|  | iron ion binding |
|  | electron transporter activity |
|  | heme binding |
|  | metal ion binding |


---

|  |  |
| --- | --- |
| Nucleotide | M34785 |
|  | NM\_007996 |
|  | AK004879 |
|  | L29123 |
|  | BT006681 |
|  | NM\_004109 |
|  | AK054524 |
|  | BC017063 |
|  | M34788 |
|  | J03548 |
|  | BQ650029 |
|  | M23668 |
|  | M23665 |
|  | D43690 |
|  | NM\_017126 |
|  | BC010284 |
|  | M34786 |
|  | BC099518 |
|  | D50436 |
|  | AK148115 |
|  | M18003 |
|  | D43689 |


---

|  |  |
| --- | --- |
| Protein | BAB23637 |
|  | NP\_004100 |
|  | NP\_058822 |
|  | AAA35856 |
|  | AAA35855 |
|  | AAA50462 |
|  | BAA07787 |
|  | AAH17063 |
|  | NP\_032022 |
|  | BAA08927 |
|  | P24483 |
|  | AAA76853 |
|  | AAA96806 |
|  | P46656 |
|  | AAA35829 |
|  | AAH99518 |
|  | P10109 |
|  | BAA07786 |
|  | AAH10284 |
|  | AAA74303 |
|  | AAP35327 |
|  | BAE28353 |


---

|  |  |
| --- | --- |
| Organism | Mammal |


---

|  |  |
| --- | --- |
| Location | chromosome 9, 9 B (Mus musculus) |
|  | chromosome 8, 8q24 (Rattus norvegicus) |
|  | chromosome 11, 11q22 (Homo sapiens) |


---

|  |  |
| --- | --- |
