# Supplementary material for: Dynamic transcriptomic profiles of zebrafish gills in response to zinc depletion
Source: BMC Genomics. 2010 Oct 8;11:548. doi: 10.1186/1471-2164-11-548 (PMC3091697; doi:10.1186/1471-2164-11-548)
Supplement: Additional file 2 — Figure S1 - Interactive Direct Interaction Network of responses to zinc depletion. Mini web-site containing index.html and hyperlinked pages in subdirectory. The web site is an interactive version of Figure 6A containing curated interactions between regulated genes and respective proteins. Legend: Molecular interactions between zinc and proteins encoded by genes changed under zinc depletion. A Direct Interaction Network was created based on curated interactions contained within the PathwayArchitect database and provided through hyperlinks. Red ovals represent proteins and the blue circle symbolizes Zn(II). Dark blue squares denote 'binding', and light blue squares 'expression'; green squares stand for 'regulation', green diamonds for 'metabolism', and green circles for 'promoter binding'. Arrow heads indicate directionality of the interaction where annotated. [file 1471-2164-11-548-S2.ZIP › PathwayArchitect Zn def DIN2/121637.html]

# PROTEIN: ACLY

|  |  |
| --- | --- |
| Name | ACLY |
| Type | PROTEIN |
| Description | ATP citrate lyase |
| Note | ATP citrate lyase is the primary enzyme responsible for the synthesis of cytosolic acetyl-CoA in many tissues. The enzyme is a tetramer (relative molecular weight approximately 440,000) of apparently identical subunits. It catalyzes the formation of acetyl-CoA and oxaloacetate from citrate and CoA with a concomitant hydrolysis of ATP to ADP and phosphate. The product, acetyl-CoA, serves several important biosynthetic pathways, including lipogenesis and cholesterogenesis. In nervous tissue, ATP citrate-lyase may be involved in the biosynthesis of acetylcholine. Two transcript variants encoding distinct isoforms have been identified for this gene. |
| Alias | Citrate cleavage enzyme |
|  | Clatp |
|  | CLATP |
|  | AW538652 |
|  | ATPCL |
|  | MGC124629 |
|  | ATP-citrate (pro-S-)-lyase |
|  | ACL |
|  | A730098H14Rik |
|  | ACLY |
|  | Acly |
|  | ATP-citrate lyase |


---

|  |  |
| --- | --- |
| GO Component | citrate lyase complex |
|  | intracellular |


---

|  |  |
| --- | --- |
| GO ID | GO:0000287 |
|  | GO:0005524 |
|  | GO:0015936 |
|  | GO:0046912 |
|  | GO:0016874 |
|  | GO:0006099 |
|  | GO:0016829 |
|  | GO:0000166 |
|  | GO:0009346 |
|  | GO:0008610 |
|  | GO:0006085 |
|  | GO:0006101 |
|  | GO:0003878 |
|  | GO:0008152 |
|  | GO:0005622 |
|  | GO:0003824 |
|  | GO:0046872 |
|  | GO:0006092 |
|  | GO:0006200 |
|  | GO:0016740 |
|  | GO:0004108 |
|  | GO:0006629 |


---

|  |  |
| --- | --- |
| MIM | MIM:108728 |


---

|  |  |
| --- | --- |
| Connectivity | 131 |


---

|  |  |
| --- | --- |
| Entrez ID | 104112 |
|  | 24159 |
|  | 47 |


---

|  |  |
| --- | --- |
| Agilent ID | A\_53\_P171926 |
|  | A\_43\_P11761 |
|  | A\_44\_P1004037 |
|  | A\_53\_P171501 |
|  | A\_14\_P113387 |
|  | A\_14\_P108004 |
|  | A\_51\_P261718 |
|  | A\_53\_P148847 |
|  | A\_23\_P66787 |
|  | A\_52\_P373556 |


---

|  |  |
| --- | --- |
| Cellular Localization | Cytoplasm |
|  | Cell |


---

|  |  |
| --- | --- |
| DbXref | Reactome##172710##Lipid metabolism##http://www.reactome.org/cgi-bin/eventbrowser?DB=gk\_current&ID=172710 |
|  | KEGG pathway##00020##Citrate cycle (TCA cycle)##http://www.genome.jp/dbget-bin/show\_pathway?rno00020+24159 |
|  | KEGG pathway##00020##Citrate cycle (TCA cycle)##http://www.genome.jp/dbget-bin/show\_pathway?hsa00020+47 |
|  | KEGG pathway##00020##Citrate cycle (TCA cycle)##http://www.genome.jp/dbget-bin/show\_pathway?mmu00020+104112 |
|  | Reactome##73923##Lipid metabolism##http://www.reactome.org/cgi-bin/eventbrowser?DB=gk\_current&ID=73923 |


---

|  |  |
| --- | --- |
| Pathway | Zn def RIN |
|  | Master Regulators |
|  | Zn def DIN |


---

|  |  |
| --- | --- |
| GO Process | main pathways of carbohydrate metabolism |
|  | coenzyme A metabolism |
|  | acetyl-CoA biosynthesis |
|  | metabolism |
|  | citrate metabolism |
|  | tricarboxylic acid cycle |
|  | ATP catabolism |
|  | lipid metabolism |
|  | lipid biosynthesis |


---

|  |  |
| --- | --- |
| UniGene | Hs.387567 |
|  | Rn.29771 |
|  | Mm.282039 |


---

|  |  |
| --- | --- |
| Affymetrix Probeset ID | 1367854\_at |
|  | 1395841\_at |
|  | 1425326\_at |
|  | 1438389\_x\_at |
|  | 1439445\_x\_at |
|  | 1439459\_x\_at |
|  | 1446315\_at |
|  | 1451666\_at |
|  | 160207\_at |
|  | 170897\_r\_at |
|  | 201127\_s\_at |
|  | 201128\_s\_at |
|  | 210337\_s\_at |
|  | 40881\_at |
|  | aa271471\_s\_at |
|  | g4501864\_3p\_s\_at |
|  | g603073\_3p\_s\_at |
|  | J05210\_at |
|  | J05210\_g\_at |
|  | L27075\_at |
|  | L27075\_g\_at |
|  | Msa.17426.0\_s\_at |
|  | Msa.18310.0\_s\_at |
|  | Msa.28960.0\_s\_at |
|  | Msa.7212.0\_s\_at |
|  | Msa.8346.0\_at |
|  | X64330\_at |
|  | 1388419\_at |
|  | 95519\_at |
|  | RC\_H08548\_s\_at |
|  | rc\_AI171307\_at |


---

|  |  |
| --- | --- |
| EC Number | EC 2.3.3.8 |


---

|  |  |
| --- | --- |
| GO Function | lyase activity |
|  | magnesium ion binding |
|  | transferase activity, transferring acyl groups, acyl groups converted into alkyl on transfer |
|  | transferase activity |
|  | nucleotide binding |
|  | catalytic activity |
|  | ligase activity |
|  | ATP binding |
|  | citrate (Si)-synthase activity |
|  | ATP citrate synthase activity |
|  | metal ion binding |


---

|  |  |
| --- | --- |
| Nucleotide | BC065805 |
|  | L47303 |
|  | L47320 |
|  | L47313 |
|  | AK162364 |
|  | AF332052 |
|  | L47316 |
|  | BC100618 |
|  | X64330 |
|  | NM\_134037 |
|  | L47308 |
|  | L47317 |
|  | L47305 |
|  | BC005533 |
|  | L47306 |
|  | AK190254 |
|  | AK078680 |
|  | AF332051 |
|  | AK132755 |
|  | BC006195 |
|  | BG037168 |
|  | AF063905 |
|  | AK136812 |
|  | AK215793 |
|  | AK095084 |
|  | AK169703 |
|  | L47318 |
|  | L47315 |
|  | L47310 |
|  | AK043466 |
|  | L47302 |
|  | L47309 |
|  | AK195919 |
|  | L47314 |
|  | AB210035 |
|  | L47311 |
|  | L47307 |
|  | L47300 |
|  | BC021502 |
|  | AK149660 |
|  | NM\_198830 |
|  | NM\_016987 |
|  | L47301 |
|  | L47304 |
|  | BC056378 |
|  | L47312 |
|  | J05210 |
|  | AK135528 |
|  | AK043434 |
|  | NM\_001096 |
|  | L47319 |
|  | U18197 |


---

|  |  |
| --- | --- |
| Protein | CAA45614 |
|  | BAE29010 |
|  | BAE41315 |
|  | AAL34316 |
|  | AAK56081 |
|  | AAB60340 |
|  | AAC95334 |
|  | AAH65805 |
|  | AAK56080 |
|  | BAE36874 |
|  | NP\_001087 |
|  | BAE23135 |
|  | AAH56378 |
|  | NP\_942127 |
|  | P53396 |
|  | BAC04484 |
|  | AAH21502 |
|  | AAI00619 |
|  | NP\_058683 |
|  | BAE06117 |
|  | NP\_598798 |
|  | P16638 |
|  | AAH05533 |
|  | Q91V92 |
|  | AAA74463 |
|  | AAH06195 |
|  | BAE21336 |


---

|  |  |
| --- | --- |
| Organism | Mammal |


---

|  |  |
| --- | --- |
| Location | chromosome 10, 10q32.1 (Rattus norvegicus) |
|  | chromosome 11, 11 D (Mus musculus) |
|  | chromosome 17, 17q12-q21 (Homo sapiens) |


---

|  |  |
| --- | --- |
