# Supplementary material for: Dynamic transcriptomic profiles of zebrafish gills in response to zinc depletion
Source: BMC Genomics. 2010 Oct 8;11:548. doi: 10.1186/1471-2164-11-548 (PMC3091697; doi:10.1186/1471-2164-11-548)
Supplement: Additional file 2 — Figure S1 - Interactive Direct Interaction Network of responses to zinc depletion. Mini web-site containing index.html and hyperlinked pages in subdirectory. The web site is an interactive version of Figure 6A containing curated interactions between regulated genes and respective proteins. Legend: Molecular interactions between zinc and proteins encoded by genes changed under zinc depletion. A Direct Interaction Network was created based on curated interactions contained within the PathwayArchitect database and provided through hyperlinks. Red ovals represent proteins and the blue circle symbolizes Zn(II). Dark blue squares denote 'binding', and light blue squares 'expression'; green squares stand for 'regulation', green diamonds for 'metabolism', and green circles for 'promoter binding'. Arrow heads indicate directionality of the interaction where annotated. [file 1471-2164-11-548-S2.ZIP › PathwayArchitect Zn def DIN2/122591.html]

# PROTEIN: HADHA

|  |  |
| --- | --- |
| Name | HADHA |
| Type | PROTEIN |
| Description | hydroxyacyl-Coenzyme A dehydrogenase/3-ketoacyl-Coenzyme A thiolase/enoyl-Coenzyme A hydratase (trifunctional protein), alpha subunit |
| Note | The HADHA and HADHB (MIM 143450) genes encode the alpha and beta subunits of the mitochondrial trifunctional protein, respectively. The heterocomplex contains 4 alpha and 4 beta subunits and catalyzes 3 steps in mitochondrial beta-oxidation of fatty acids, including the long-chain 3-hydroxyl-CoA dehydrogenase (LCHAD) step. The alpha subunit harbors the 3-hydroxyacyl-CoA dehydrogenase and enoyl-CoA hydratase activities (Kamijo et al., 1994).[supplied by OMIM] |
| Alias | Mtpa |
|  | LCHAD |
|  | hydroxyacyl dehydrogenase, subunit A |
|  | hydroxyacyl-Coenzyme A dehydrogenase/3-ketoacyl-Coenzyme A hiolase/enoyl-Coenzyme A hydratase (trifunctional protein), alpha subunit |
|  | hydroxyacyl-Coenzyme A dehydrogenase/3-ketoacyl-Coenzyme A hiolase/enoyl-Coenzyme A hydratase (trifunctional protein) alpha subunit |
|  | MGC105338 |
|  | TP-alpha |
|  | MTPA |
|  | GBP |
|  | C77020 |
|  | mitochondrial trifunctional protein, alpha subunit |
|  | Hadha |
|  | long-chain hydroxyacyl-CoA dehydrogenase |
|  | trifunctional protein, alpha subunit |


---

|  |  |
| --- | --- |
| GO Component | mitochondrion |
|  | mitochondrial inner membrane |
|  | fatty acid beta-oxidation multienzyme complex |


---

|  |  |
| --- | --- |
| GO ID | GO:0016507 |
|  | GO:0008152 |
|  | GO:0003824 |
|  | GO:0006635 |
|  | GO:0016829 |
|  | GO:0006631 |
|  | GO:0016509 |
|  | GO:0005743 |
|  | GO:0003985 |
|  | GO:0003857 |
|  | GO:0005739 |
|  | GO:0016508 |
|  | GO:0006629 |
|  | GO:0016491 |
|  | GO:0004300 |


---

|  |  |
| --- | --- |
| MIM | MIM:600890 |


---

|  |  |
| --- | --- |
| Connectivity | 30 |


---

|  |  |
| --- | --- |
| Entrez ID | 170670 |
|  | 97212 |
|  | 3030 |


---

|  |  |
| --- | --- |
| Agilent ID | A\_44\_P400202 |
|  | A\_52\_P338459 |
|  | A\_24\_P242688 |
|  | A\_42\_P794865 |
|  | A\_51\_P331549 |
|  | A\_24\_P353964 |
|  | A\_44\_P414469 |
|  | A\_14\_P130052 |
|  | A\_53\_P103330 |
|  | A\_53\_P154337 |
|  | A\_44\_P480296 |
|  | A\_52\_P193029 |
|  | A\_23\_P159510 |
|  | A\_52\_P658044 |


---

|  |  |
| --- | --- |
| Cellular Localization | Mitochondrion |
|  | Cytoplasm |
|  | Membrane |
|  | Organelle |
|  | Cell |


---

|  |  |
| --- | --- |
| DbXref | KEGG pathway##00640##Propanoate metabolism##http://www.genome.jp/dbget-bin/show\_pathway?hsa00640+3030 |
|  | KEGG pathway##00280##Valine, leucine and isoleucine degradation##http://www.genome.jp/dbget-bin/show\_pathway?hsa00280+3030 |
|  | KEGG pathway##00650##Butanoate metabolism##http://www.genome.jp/dbget-bin/show\_pathway?mmu00650+97212 |
|  | KEGG pathway##00903##Limonene and pinene degradation##http://www.genome.jp/dbget-bin/show\_pathway?mmu00903+97212 |
|  | KEGG pathway##00380##Tryptophan metabolism##http://www.genome.jp/dbget-bin/show\_pathway?mmu00380+97212 |
|  | KEGG pathway##00071##Fatty acid metabolism##http://www.genome.jp/dbget-bin/show\_pathway?hsa00071+3030 |
|  | KEGG pathway##00062##Fatty acid biosynthesis (path 2)##http://www.genome.jp/dbget-bin/show\_pathway?hsa00062+3030 |
|  | KEGG pathway##00062##Fatty acid elongation in mitochondria##http://www.genome.jp/dbget-bin/show\_pathway?mmu00062+97212 |
|  | KEGG pathway##00632##Benzoate degradation via CoA ligation##http://www.genome.jp/dbget-bin/show\_pathway?mmu00632+97212 |
|  | KEGG pathway##00310##Lysine degradation##http://www.genome.jp/dbget-bin/show\_pathway?mmu00310+97212 |
|  | KEGG pathway##00071##Fatty acid metabolism##http://www.genome.jp/dbget-bin/show\_pathway?mmu00071+97212 |
|  | KEGG pathway##00930##Caprolactam degradation##http://www.genome.jp/dbget-bin/show\_pathway?hsa00930+3030 |
|  | Reactome##73923##Lipid metabolism##http://www.reactome.org/cgi-bin/eventbrowser?DB=gk\_current&ID=73923 |
|  | KEGG pathway##00410##beta-Alanine metabolism##http://www.genome.jp/dbget-bin/show\_pathway?mmu00410+97212 |
|  | KEGG pathway##00410##beta-Alanine metabolism##http://www.genome.jp/dbget-bin/show\_pathway?hsa00410+3030 |
|  | KEGG pathway##00640##Propanoate metabolism##http://www.genome.jp/dbget-bin/show\_pathway?mmu00640+97212 |
|  | KEGG pathway##00650##Butanoate metabolism##http://www.genome.jp/dbget-bin/show\_pathway?hsa00650+3030 |
|  | KEGG pathway##00380##Tryptophan metabolism##http://www.genome.jp/dbget-bin/show\_pathway?hsa00380+3030 |
|  | KEGG pathway##00632##Benzoate degradation via CoA ligation##http://www.genome.jp/dbget-bin/show\_pathway?hsa00632+3030 |
|  | KEGG pathway##00930##Caprolactam degradation##http://www.genome.jp/dbget-bin/show\_pathway?mmu00930+97212 |
|  | KEGG pathway##00310##Lysine degradation##http://www.genome.jp/dbget-bin/show\_pathway?hsa00310+3030 |
|  | KEGG pathway##00280##Valine, leucine and isoleucine degradation##http://www.genome.jp/dbget-bin/show\_pathway?mmu00280+97212 |
|  | KEGG pathway##00903##Limonene and pinene degradation##http://www.genome.jp/dbget-bin/show\_pathway?hsa00903+3030 |


---

|  |  |
| --- | --- |
| Pathway | Zn def RIN |
|  | Master Regulators |
|  | Zn def DIN |


---

|  |  |
| --- | --- |
| GO Process | fatty acid beta-oxidation |
|  | metabolism |
|  | lipid metabolism |
|  | fatty acid metabolism |


---

|  |  |
| --- | --- |
| UniGene | Rn.3340 |
|  | Mm.200497 |
|  | Hs.516032 |


---

|  |  |
| --- | --- |
| Affymetrix Probeset ID | 1370164\_at |
|  | 1394763\_at |
|  | 1452173\_at |
|  | 208629\_s\_at |
|  | 208630\_at |
|  | 208631\_s\_at |
|  | 232590\_at |
|  | 233012\_at |
|  | 236120\_at |
|  | 36952\_at |
|  | 47193\_at |
|  | 47885\_r\_at |
|  | 52313\_at |
|  | 53287\_at |
|  | 88329\_at |
|  | aa616705\_s\_at |
|  | D16478\_at |
|  | D16478\_g\_at |
|  | D16480\_at |
|  | g595266\_3p\_a\_at |
|  | Hs.122521.0.S1\_3p\_at |
|  | Hs.29258.0.S1\_3p\_at |
|  | Hs.46525.0.A1\_3p\_at |
|  | Hs.75860.0.A1\_3p\_a\_at |
|  | Hs.75860.0.A3\_3p\_at |
|  | RC\_N48717\_at |
|  | RC\_T73472\_s\_at |
|  | RC\_T90223\_f\_at |
|  | RC\_T90223\_r\_at |
|  | RC\_W37424\_at |
|  | TC26597\_at |
|  | TC26597\_g\_at |
|  | X98225\_at |
|  | X98225\_s\_at |


---

|  |  |
| --- | --- |
| GO Function | long-chain-3-hydroxyacyl-CoA dehydrogenase activity |
|  | oxidoreductase activity |
|  | lyase activity |
|  | acetyl-CoA C-acetyltransferase activity |
|  | enoyl-CoA hydratase activity |
|  | long-chain-enoyl-CoA hydratase activity |
|  | 3-hydroxyacyl-CoA dehydrogenase activity |
|  | catalytic activity |


---

|  |  |
| --- | --- |
| Nucleotide | AK050856 |
|  | AK183181 |
|  | AK215560 |
|  | AK183872 |
|  | AK053986 |
|  | AK188123 |
|  | AK170478 |
|  | AK035399 |
|  | AK209573 |
|  | AK178409 |
|  | BC037009 |
|  | AK217494 |
|  | AK184204 |
|  | X98225 |
|  | BC091697 |
|  | AK207241 |
|  | AC011742 |
|  | AK194527 |
|  | AK218789 |
|  | AK035316 |
|  | NM\_178878 |
|  | AK179416 |
|  | NM\_000182 |
|  | AK170683 |
|  | AK150188 |
|  | BC027156 |
|  | AK189494 |
|  | AK213006 |
|  | AK178342 |
|  | AK183745 |
|  | AK188264 |
|  | AB020811 |
|  | AK171469 |
|  | D16480 |
|  | U04627 |
|  | AK209398 |
|  | D16478 |
|  | BC009235 |
|  | BC046978 |
|  | NM\_130826 |
|  | AK185277 |
|  | AC010896 |
|  | AK207119 |
|  | AK029017 |
|  | BC058569 |


---

|  |  |
| --- | --- |
| Protein | Q64428 |
|  | AAH37009 |
|  | BAE41956 |
|  | CAA66885 |
|  | NP\_849209 |
|  | BAA03939 |
|  | AAH27156 |
|  | AAH46978 |
|  | AAX93141 |
|  | AAH09235 |
|  | AAH58569 |
|  | AAY14643 |
|  | BAE42475 |
|  | BAC34435 |
|  | NP\_000173 |
|  | BAE41822 |
|  | P40939 |
|  | AAH91697 |
|  | NP\_570839 |
|  | BAA03941 |
|  | BAC26245 |
|  | AAA56664 |
|  | BAA76735 |


---

|  |  |
| --- | --- |
| Organism | Mammal |


---

|  |  |
| --- | --- |
| Location | chromosome 2, 2p23 (Homo sapiens) |
|  | chromosome 5, 5 B1 (Mus musculus) |
|  | chromosome 6, 6q12 (Rattus norvegicus) |


---

|  |  |
| --- | --- |
