# Supplementary material for: Dynamic transcriptomic profiles of zebrafish gills in response to zinc depletion
Source: BMC Genomics. 2010 Oct 8;11:548. doi: 10.1186/1471-2164-11-548 (PMC3091697; doi:10.1186/1471-2164-11-548)
Supplement: Additional file 2 — Figure S1 - Interactive Direct Interaction Network of responses to zinc depletion. Mini web-site containing index.html and hyperlinked pages in subdirectory. The web site is an interactive version of Figure 6A containing curated interactions between regulated genes and respective proteins. Legend: Molecular interactions between zinc and proteins encoded by genes changed under zinc depletion. A Direct Interaction Network was created based on curated interactions contained within the PathwayArchitect database and provided through hyperlinks. Red ovals represent proteins and the blue circle symbolizes Zn(II). Dark blue squares denote 'binding', and light blue squares 'expression'; green squares stand for 'regulation', green diamonds for 'metabolism', and green circles for 'promoter binding'. Arrow heads indicate directionality of the interaction where annotated. [file 1471-2164-11-548-S2.ZIP › PathwayArchitect Zn def DIN2/123993.html]

# PROTEIN: ARG2

|  |  |
| --- | --- |
| Name | ARG2 |
| Type | PROTEIN |
| Description | arginase, type II |
| Note | Arginase catalyzes the hydrolysis of arginine to ornithine and urea. At least two isoforms of mammalian arginase exists (types I and II) which differ in their tissue distribution, subcellular localization, immunologic crossreactivity and physiologic function. The type II isoform encoded by this gene, is located in the mitochondria and expressed in extra-hepatic tissues, especially kidney. The physiologic role of this isoform is poorly understood; it is thought to play a role in nitric oxide and polyamine metabolism. Transcript variants of the type II gene resulting from the use of alternative polyadenylation sites have been described. |
| Alias | L-arginine ureahydrolase |
|  | A-II |
|  | ARG2 |
|  | arginase type II |
|  | Arginase II |
|  | L-arginine amidinohydrolase |
|  | AII type II arginase |
|  | Non- hepatic arginase |
|  | AU022422 |
|  | Kidney-type arginase |
|  | Arg2 |
|  | nonhepatic arginase |
|  | AII |
|  | kidney arginase |


---

|  |  |
| --- | --- |
| GO Component | mitochondrion |


---

|  |  |
| --- | --- |
| GO ID | GO:0004053 |
|  | GO:0045428 |
|  | GO:0003824 |
|  | GO:0019547 |
|  | GO:0006525 |
|  | GO:0046872 |
|  | GO:0016787 |
|  | GO:0005739 |
|  | GO:0000050 |
|  | GO:0006809 |
|  | GO:0006527 |
|  | GO:0030145 |


---

|  |  |
| --- | --- |
| MIM | MIM:107830 |


---

|  |  |
| --- | --- |
| Connectivity | 153 |


---

|  |  |
| --- | --- |
| Entrez ID | 384 |
|  | 11847 |
|  | 29215 |


---

|  |  |
| --- | --- |
| Agilent ID | A\_44\_P296180 |
|  | A\_23\_P128728 |
|  | A\_53\_P145444 |
|  | A\_53\_P159886 |
|  | A\_52\_P374897 |
|  | A\_51\_P501632 |
|  | A\_14\_P102817 |
|  | A\_43\_P11960 |


---

|  |  |
| --- | --- |
| Cellular Localization | Mitochondrion |
|  | Cytoplasm |
|  | Organelle |
|  | Cell |


---

|  |  |
| --- | --- |
| DbXref | KEGG pathway##00220##Urea cycle and metabolism of amino groups##http://www.genome.jp/dbget-bin/show\_pathway?mmu00220+11847 |
|  | KEGG pathway##00330##Arginine and proline metabolism##http://www.genome.jp/dbget-bin/show\_pathway?mmu00330+11847 |
|  | KEGG pathway##00220##Urea cycle and metabolism of amino groups##http://www.genome.jp/dbget-bin/show\_pathway?hsa00220+384 |
|  | KEGG pathway##00330##Arginine and proline metabolism##http://www.genome.jp/dbget-bin/show\_pathway?rno00330+29215 |
|  | KEGG pathway##00330##Arginine and proline metabolism##http://www.genome.jp/dbget-bin/show\_pathway?hsa00330+384 |
|  | KEGG pathway##00220##Urea cycle and metabolism of amino groups##http://www.genome.jp/dbget-bin/show\_pathway?rno00220+29215 |


---

|  |  |
| --- | --- |
| Pathway | Zn def RIN |
|  | Master Regulators |
|  | Zn def DIN |


---

|  |  |
| --- | --- |
| GO Process | arginine catabolism to ornithine |
|  | urea cycle |
|  | arginine catabolism |
|  | regulation of nitric oxide biosynthesis |
|  | nitric oxide biosynthesis |
|  | arginine metabolism |


---

|  |  |
| --- | --- |
| UniGene | Mm.3506 |
|  | Rn.11055 |
|  | Hs.553485 |


---

|  |  |
| --- | --- |
| Affymetrix Probeset ID | 1368672\_at |
|  | 1376107\_at |
|  | 1378372\_at |
|  | 1381296\_at |
|  | 1393304\_at |
|  | 1418847\_at |
|  | 1438841\_s\_at |
|  | 203945\_at |
|  | 203946\_s\_at |
|  | 32722\_at |
|  | 52079\_at |
|  | 1394206\_s\_at |
|  | 1386416\_at |
|  | 98473\_at |
|  | af032466\_s\_at |
|  | g10947110\_3p\_at |
|  | g1763757\_3p\_a\_at |
|  | rc\_AA996521\_at |
|  | U82256\_at |
|  | U90887\_at |
|  | 101259\_at |
|  | 161246\_at |
|  | rc\_AI639084\_at |
|  | aa666971\_f\_at |


---

|  |  |
| --- | --- |
| EC Number | EC 3.5.3.1 |


---

|  |  |
| --- | --- |
| GO Function | hydrolase activity |
|  | manganese ion binding |
|  | arginase activity |
|  | catalytic activity |
|  | metal ion binding |


---

|  |  |
| --- | --- |
| Nucleotide | U90886 |
|  | U82256 |
|  | CR536550 |
|  | U90887 |
|  | AF045965 |
|  | AF508019 |
|  | AY074489 |
|  | AK137142 |
|  | D86928 |
|  | BC023349 |
|  | BC008464 |
|  | AF045963 |
|  | D86724 |
|  | NM\_019168 |
|  | NM\_009705 |
|  | BC001350 |
|  | AF045961 |
|  | NM\_001172 |
|  | AF045960 |
|  | AF045964 |
|  | AF032466 |
|  | BC029050 |
|  | AF045962 |
|  | AF044680 |
|  | AF045959 |
|  | U75667 |


---

|  |  |
| --- | --- |
| Protein | AAC51664 |
|  | NP\_033835 |
|  | O08701 |
|  | O08691 |
|  | AAC22548 |
|  | CAG38787 |
|  | AAH01350 |
|  | AAM34681 |
|  | BAA13158 |
|  | AAB39855 |
|  | AAB86959 |
|  | AAH29050 |
|  | AAL71548 |
|  | AAC22580 |
|  | NP\_062041 |
|  | AAC78460 |
|  | P78540 |
|  | AAH23349 |
|  | NP\_001163 |
|  | AAH08464 |
|  | BAA13183 |


---

|  |  |
| --- | --- |
| Organism | Mammal |


---

|  |  |
| --- | --- |
| Location | chromosome 14, 14q24.1-q24.3 (Homo sapiens) |
|  | chromosome 6, 6q24 (Rattus norvegicus) |
|  | chromosome 12, 12 C3 (Mus musculus) |


---

|  |  |
| --- | --- |
