# Supplementary material for: Dynamic transcriptomic profiles of zebrafish gills in response to zinc depletion
Source: BMC Genomics. 2010 Oct 8;11:548. doi: 10.1186/1471-2164-11-548 (PMC3091697; doi:10.1186/1471-2164-11-548)
Supplement: Additional file 2 — Figure S1 - Interactive Direct Interaction Network of responses to zinc depletion. Mini web-site containing index.html and hyperlinked pages in subdirectory. The web site is an interactive version of Figure 6A containing curated interactions between regulated genes and respective proteins. Legend: Molecular interactions between zinc and proteins encoded by genes changed under zinc depletion. A Direct Interaction Network was created based on curated interactions contained within the PathwayArchitect database and provided through hyperlinks. Red ovals represent proteins and the blue circle symbolizes Zn(II). Dark blue squares denote 'binding', and light blue squares 'expression'; green squares stand for 'regulation', green diamonds for 'metabolism', and green circles for 'promoter binding'. Arrow heads indicate directionality of the interaction where annotated. [file 1471-2164-11-548-S2.ZIP › PathwayArchitect Zn def DIN2/124376.html]

# PROTEIN: C8G

|  |  |
| --- | --- |
| Name | C8G |
| Type | PROTEIN |
| Description | complement component 8, gamma polypeptide |
| Note | C8G is one of the three polypeptides that constitute C8, a component of the complement system. C8 participates in the formation of Membrane Attack Complex (MAC). Patients with deficiency in C8 are vulnerable to certain bacteria infection. |
| Alias | Complement component C8 gamma chain precursor |
|  | C8C |
|  | C8G |


---

|  |  |
| --- | --- |
| GO Component | extracellular space |
|  | membrane attack complex |


---

|  |  |
| --- | --- |
| GO ID | GO:0006810 |
|  | GO:0006956 |
|  | GO:0005615 |
|  | GO:0019835 |
|  | GO:0006957 |
|  | GO:0005488 |
|  | GO:0019841 |
|  | GO:0006958 |
|  | GO:0005579 |
|  | GO:0006955 |
|  | GO:0005215 |


---

|  |  |
| --- | --- |
| MIM | MIM:120930 |


---

|  |  |
| --- | --- |
| Connectivity | 6 |


---

|  |  |
| --- | --- |
| Entrez ID | 733 |
|  | 110407 |


---

|  |  |
| --- | --- |
| Agilent ID | A\_23\_P20713 |
|  | A\_14\_P112925 |


---

|  |  |
| --- | --- |
| Cellular Localization | Extracellular region |
|  | Extracellular matrix |


---

|  |  |
| --- | --- |
| DbXref | KEGG pathway##04610##Complement and coagulation cascades##http://www.genome.jp/dbget-bin/show\_pathway?hsa04610+733 |


---

|  |  |
| --- | --- |
| Pathway | Zn def RIN |
|  | Master Regulators |
|  | Zn xs inventory |
|  | Zn def DIN |


---

|  |  |
| --- | --- |
| GO Process | complement activation, alternative pathway |
|  | transport |
|  | complement activation |
|  | immune response |
|  | cytolysis |
|  | complement activation, classical pathway |


---

|  |  |
| --- | --- |
| UniGene | Hs.1285 |


---

|  |  |
| --- | --- |
| Affymetrix Probeset ID | 210324\_at |
|  | 35524\_at |
|  | g179723\_3p\_at |
|  | U08198\_rna1\_at |
|  | RC\_AA344866\_s\_at |


---

|  |  |
| --- | --- |
| GO Function | transporter activity |
|  | binding |
|  | retinol binding |


---

|  |  |
| --- | --- |
| Nucleotide | AL807752 |
|  | U08198 |
|  | M17999 |
|  | M17263 |
|  | X06465 |
|  | NM\_000606 |


---

|  |  |
| --- | --- |
| Protein | AAA51888 |
|  | NP\_000597 |
|  | CAA29773 |
|  | CAI12749 |
|  | P07360 |
|  | AAA51863 |
|  | AAA18482 |


---

|  |  |
| --- | --- |
| Organism | Mammal |


---

|  |  |
| --- | --- |
| Location | chromosome 9, 9q34.3 (Homo sapiens) |
|  | 2 12.3 cM (Mus musculus) |
|  | chromosome 2, 2 12.3 cM, 2 (Mus musculus) |


---

|  |  |
| --- | --- |
