# Supplementary material for: Dynamic transcriptomic profiles of zebrafish gills in response to zinc depletion
Source: BMC Genomics. 2010 Oct 8;11:548. doi: 10.1186/1471-2164-11-548 (PMC3091697; doi:10.1186/1471-2164-11-548)
Supplement: Additional file 2 — Figure S1 - Interactive Direct Interaction Network of responses to zinc depletion. Mini web-site containing index.html and hyperlinked pages in subdirectory. The web site is an interactive version of Figure 6A containing curated interactions between regulated genes and respective proteins. Legend: Molecular interactions between zinc and proteins encoded by genes changed under zinc depletion. A Direct Interaction Network was created based on curated interactions contained within the PathwayArchitect database and provided through hyperlinks. Red ovals represent proteins and the blue circle symbolizes Zn(II). Dark blue squares denote 'binding', and light blue squares 'expression'; green squares stand for 'regulation', green diamonds for 'metabolism', and green circles for 'promoter binding'. Arrow heads indicate directionality of the interaction where annotated. [file 1471-2164-11-548-S2.ZIP › PathwayArchitect Zn def DIN2/125480.html]

# PROTEIN: TNNI3

|  |  |
| --- | --- |
| Name | TNNI3 |
| Type | PROTEIN |
| Description | troponin I, cardiac |
| Note | Troponin I (TnI) is one of 3 subunits that form the troponin complex of the thin filaments of striated muscle; see also TNNI1 (MIM 191042). The others are troponin T (TnT; see MIM 191041) and troponin C (TnC; see MIM 191040).[supplied by OMIM] |
| Alias | troponin 1 type 3 |
|  | TNNC1 |
|  | CMH7 |
|  | familial hypertrophic cardiomyopathy 7 |
|  | Tni |
|  | Tnni3 |
|  | Ctni |
|  | Tn1 |
|  | MGC116817 |
|  | cTnI |
|  | troponin 1, type 3 |
|  | TnI |
|  | cTNI |
|  | Cardiac troponin I |


---

|  |  |
| --- | --- |
| GO Component | troponin complex |
|  | sarcomere |
|  | cytoplasm |


---

|  |  |
| --- | --- |
| GO ID | GO:0019855 |
|  | GO:0016525 |
|  | GO:0007517 |
|  | GO:0005200 |
|  | GO:0005737 |
|  | GO:0030017 |
|  | GO:0006937 |
|  | GO:0003779 |
|  | GO:0005515 |
|  | GO:0008016 |
|  | GO:0005861 |


---

|  |  |
| --- | --- |
| MIM | MIM:115210 |
|  | MIM:191044 |


---

|  |  |
| --- | --- |
| Connectivity | 136 |


---

|  |  |
| --- | --- |
| Entrez ID | 21954 |
|  | 7137 |
|  | 29248 |


---

|  |  |
| --- | --- |
| Agilent ID | A\_23\_P67447 |
|  | A\_14\_P137726 |
|  | A\_42\_P676553 |
|  | A\_53\_P180566 |
|  | A\_52\_P563123 |
|  | A\_51\_P386983 |
|  | A\_23\_P67453 |


---

|  |  |
| --- | --- |
| Cellular Localization | Cytoplasm |
|  | Cytoskeleton |
|  | Cell |
|  | Organelle |


---

|  |  |
| --- | --- |
| Pathway | Zn def RIN |
|  | Zn def DIN |


---

|  |  |
| --- | --- |
| GO Process | negative regulation of angiogenesis |
|  | regulation of heart contraction rate |
|  | regulation of muscle contraction |
|  | muscle development |


---

|  |  |
| --- | --- |
| UniGene | Hs.512709 |
|  | Mm.27674 |
|  | Rn.64141 |


---

|  |  |
| --- | --- |
| Affymetrix Probeset ID | 100921\_at |
|  | 1386931\_at |
|  | 1422536\_at |
|  | 205742\_at |
|  | 36477\_at |
|  | g4507622\_3p\_at |
|  | M92074\_at |
|  | M92074\_g\_at |
|  | X90780\_rna1\_at |
|  | z22784\_s\_at |
|  | TC27049\_s\_at |


---

|  |  |
| --- | --- |
| GO Function | protein binding |
|  | actin binding |
|  | structural constituent of cytoskeleton |
|  | calcium channel inhibitor activity |


---

|  |  |
| --- | --- |
| Nucleotide | AF159424 |
|  | DQ062462 |
|  | X90780 |
|  | AK169223 |
|  | M64247 |
|  | NM\_017144 |
|  | BC061171 |
|  | NM\_000363 |
|  | U09181 |
|  | M92074 |
|  | M57679 |
|  | NM\_009406 |
|  | U77354 |
|  | BT019517 |
|  | Z22784 |
|  | BC096166 |
|  | BC096165 |
|  | CR541985 |
|  | X58499 |
|  | AK223451 |
|  | BC096167 |
|  | CR542012 |
|  | BC100590 |
|  | X54163 |
|  | BC099631 |


---

|  |  |
| --- | --- |
| Protein | NP\_000354 |
|  | CAA80459 |
|  | NP\_058840 |
|  | AAB52234 |
|  | AAH96167 |
|  | AAV38324 |
|  | AAY63993 |
|  | P23693 |
|  | AAF19152 |
|  | P48787 |
|  | BAD97171 |
|  | CAA41402 |
|  | AAI00591 |
|  | AAH96165 |
|  | P19429 |
|  | CAA38102 |
|  | AAA16157 |
|  | AAH61171 |
|  | AAH96166 |
|  | NP\_033432 |
|  | CAG46809 |
|  | BAE40992 |
|  | AAA63504 |
|  | AAA42294 |
|  | CAA62301 |
|  | AAH99631 |
|  | AAA19657 |
|  | CAG46782 |


---

|  |  |
| --- | --- |
| Organism | Mammal |


---

|  |  |
| --- | --- |
| Location | chromosome 19, 19q13.4 (Homo sapiens) |
|  | 7 9.0 cM (Mus musculus) |
|  | chromosome 1, 1q12 (Rattus norvegicus) |
|  | chromosome 7, 7 9.0 cM, 7 A1 (Mus musculus) |


---

|  |  |
| --- | --- |
