# Supplementary material for: Dynamic transcriptomic profiles of zebrafish gills in response to zinc depletion
Source: BMC Genomics. 2010 Oct 8;11:548. doi: 10.1186/1471-2164-11-548 (PMC3091697; doi:10.1186/1471-2164-11-548)
Supplement: Additional file 2 — Figure S1 - Interactive Direct Interaction Network of responses to zinc depletion. Mini web-site containing index.html and hyperlinked pages in subdirectory. The web site is an interactive version of Figure 6A containing curated interactions between regulated genes and respective proteins. Legend: Molecular interactions between zinc and proteins encoded by genes changed under zinc depletion. A Direct Interaction Network was created based on curated interactions contained within the PathwayArchitect database and provided through hyperlinks. Red ovals represent proteins and the blue circle symbolizes Zn(II). Dark blue squares denote 'binding', and light blue squares 'expression'; green squares stand for 'regulation', green diamonds for 'metabolism', and green circles for 'promoter binding'. Arrow heads indicate directionality of the interaction where annotated. [file 1471-2164-11-548-S2.ZIP › PathwayArchitect Zn def DIN2/127691.html]

# PROTEIN: PODXL

|  |  |
| --- | --- |
| Name | PODXL |
| Type | PROTEIN |
| Description | podocalyxin-like |
| Note | This gene encodes a member of the sialomucin protein family. The encoded protein was originally identified as an important component of glomerular podocytes. Podocytes are highly differentiated epithelial cells with interdigitating foot processes covering the outer aspect of the glomerular basement membrane. Other biological activities of the encoded protein include: binding in a membrane protein complex with Na+/H+ exchanger regulatory factor to intracellular cytoskeletal elements, playing a role in hematopoetic cell differentiation, and being expressed in vascular endothelium cells and binding to L-selectin. |
| Alias | podocalyxin |
|  | Podocalyxin-like protein 1 precursor |
|  | AW121214 |
|  | Pclp1 |
|  | PCLP |
|  | Gp200 |
|  | PC |
|  | Podocalyxin precursor |
|  | PCLP1 |
|  | Podxl |
|  | Ly102 |
|  | PODXL |


---

|  |  |
| --- | --- |
| GO Component | extracellular space |
|  | integral to membrane |
|  | plasma membrane |
|  | integral to plasma membrane |


---

|  |  |
| --- | --- |
| GO ID | GO:0005615 |
|  | GO:0005524 |
|  | GO:0007162 |
|  | GO:0050900 |
|  | GO:0006468 |
|  | GO:0005887 |
|  | GO:0004672 |
|  | GO:0016021 |
|  | GO:0005886 |


---

|  |  |
| --- | --- |
| MIM | MIM:602632 |


---

|  |  |
| --- | --- |
| Connectivity | 24 |


---

|  |  |
| --- | --- |
| Entrez ID | 192181 |
|  | 5420 |
|  | 27205 |


---

|  |  |
| --- | --- |
| Agilent ID | A\_53\_P167015 |
|  | A\_14\_P100222 |
|  | A\_53\_P127099 |
|  | A\_14\_P116146 |
|  | A\_14\_P118702 |
|  | A\_51\_P118284 |
|  | A\_23\_P215060 |
|  | A\_53\_P176481 |
|  | A\_14\_P125005 |
|  | A\_42\_P582467 |
|  | A\_14\_P135362 |
|  | A\_52\_P380263 |
|  | A\_14\_P127941 |
|  | A\_14\_P129566 |


---

|  |  |
| --- | --- |
| Cellular Localization | Plasma membrane |
|  | Membrane |
|  | Extracellular region |
|  | Cell |


---

|  |  |
| --- | --- |
| Pathway | Zn def RIN |
|  | Master Regulators |
|  | Zn def DIN |


---

|  |  |
| --- | --- |
| GO Process | negative regulation of cell adhesion |
|  | immune cell migration |
|  | protein amino acid phosphorylation |


---

|  |  |
| --- | --- |
| UniGene | Hs.16426 |
|  | Mm.89918 |
|  | Rn.13805 |


---

|  |  |
| --- | --- |
| Affymetrix Probeset ID | 100394\_at |
|  | 112828\_at |
|  | 1369895\_s\_at |
|  | 1385823\_s\_at |
|  | 1387933\_s\_at |
|  | 1417396\_at |
|  | 1448688\_at |
|  | 170691\_r\_at |
|  | 201578\_at |
|  | 40434\_at |
|  | g4885556\_3p\_at |
|  | rc\_AA899249\_at |
|  | rc\_AA964715\_at |
|  | rc\_AI111966\_at |
|  | TC34322\_at |
|  | U97519\_at |


---

|  |  |
| --- | --- |
| GO Function | protein kinase activity |
|  | ATP binding |


---

|  |  |
| --- | --- |
| Nucleotide | BC093730 |
|  | AB020726 |
|  | AF357453 |
|  | AF290208 |
|  | BX641124 |
|  | AF109393 |
|  | NM\_013723 |
|  | CH236950 |
|  | AB028048 |
|  | NM\_005397 |
|  | U97519 |
|  | AK055816 |
|  | BG006197 |
|  | BC070886 |
|  | NM\_138848 |
|  | BC042466 |
|  | NM\_001018111 |
|  | BC052442 |
|  | BC054530 |
|  | AK134723 |
|  | AF290209 |
|  | AK223573 |


---

|  |  |
| --- | --- |
| Protein | AAB61574 |
|  | Q9WTQ2 |
|  | AAH54530 |
|  | AAG02458 |
|  | AAF14238 |
|  | Q9R0M4 |
|  | BAE22258 |
|  | O00592 |
|  | AAH70886 |
|  | BAD97293 |
|  | AAH52442 |
|  | NP\_001018121 |
|  | BAA86912 |
|  | BAB71022 |
|  | EAL24080 |
|  | NP\_620203 |
|  | AAH93730 |
|  | NP\_038751 |
|  | NP\_005388 |
|  | AAL27890 |
|  | BAA78375 |


---

|  |  |
| --- | --- |
| Organism | Mammal |


---

|  |  |
| --- | --- |
| Location | 6 10.0 cM (Mus musculus) |
|  | chromosome 4, 4q22 (Rattus norvegicus) |
|  | chromosome 7, 7q32-q33 (Homo sapiens) |
|  | chromosome 6, 6 10.0 cM, 6 A3.3 (Mus musculus) |


---

|  |  |
| --- | --- |
