# Supplementary material for: Dynamic transcriptomic profiles of zebrafish gills in response to zinc depletion
Source: BMC Genomics. 2010 Oct 8;11:548. doi: 10.1186/1471-2164-11-548 (PMC3091697; doi:10.1186/1471-2164-11-548)
Supplement: Additional file 2 — Figure S1 - Interactive Direct Interaction Network of responses to zinc depletion. Mini web-site containing index.html and hyperlinked pages in subdirectory. The web site is an interactive version of Figure 6A containing curated interactions between regulated genes and respective proteins. Legend: Molecular interactions between zinc and proteins encoded by genes changed under zinc depletion. A Direct Interaction Network was created based on curated interactions contained within the PathwayArchitect database and provided through hyperlinks. Red ovals represent proteins and the blue circle symbolizes Zn(II). Dark blue squares denote 'binding', and light blue squares 'expression'; green squares stand for 'regulation', green diamonds for 'metabolism', and green circles for 'promoter binding'. Arrow heads indicate directionality of the interaction where annotated. [file 1471-2164-11-548-S2.ZIP › PathwayArchitect Zn def DIN2/129507.html]

# PROTEIN: FMR1

|  |  |
| --- | --- |
| Name | FMR1 |
| Type | PROTEIN |
| Description | fragile X mental retardation 1 |
| Note | X-linked mental retardation associated with marXq28, or fragile X syndrome, is characterized by moderate to severe mental retardation, macroorchidism, large ears, prominent jaw, and high-pitched jocular speech. Expression is variable, with mental retardation being the most common feature. This phenotype is associated with mutations in the FMR1 gene. McCabe et al. (1999) [PubMed 10398250] summarized the proceedings of a workshop on the fragile X syndrome held in December 1998.[supplied by OMIM] |
| Alias | FMR1 |
|  | Protein FMR-1 |
|  | ragile X mental retardation protein |
|  | FRAXA |
|  | FMRP |
|  | Fmr1 |
|  | fragile X mental retardation-1 protein |
|  | MGC87458 |
|  | fragile X mental retardation protein 1 |
|  | Fragile X mental retardation 1 |
|  | Fmr-1 |


---

|  |  |
| --- | --- |
| GO Component | nucleoplasm |
|  | ribosome |
|  | nucleus |
|  | soluble fraction |
|  | polysome |


---

|  |  |
| --- | --- |
| GO ID | GO:0005840 |
|  | GO:0006397 |
|  | GO:0006406 |
|  | GO:0006810 |
|  | GO:0003729 |
|  | GO:0005634 |
|  | GO:0003723 |
|  | GO:0005654 |
|  | GO:0006355 |
|  | GO:0007417 |
|  | GO:0005625 |
|  | GO:0003676 |
|  | GO:0006445 |
|  | GO:0005844 |


---

|  |  |
| --- | --- |
| MIM | MIM:309550 |


---

|  |  |
| --- | --- |
| Connectivity | 86 |


---

|  |  |
| --- | --- |
| Entrez ID | 24948 |
|  | 14265 |
|  | 2332 |


---

|  |  |
| --- | --- |
| Agilent ID | A\_52\_P493091 |
|  | A\_53\_P144569 |
|  | A\_24\_P40417 |
|  | A\_44\_P285507 |
|  | A\_44\_P180017 |
|  | A\_23\_P217148 |
|  | A\_14\_P103225 |
|  | A\_14\_P128706 |
|  | A\_51\_P378079 |
|  | A\_53\_P120385 |
|  | A\_14\_P201584 |
|  | A\_24\_P93967 |


---

|  |  |
| --- | --- |
| Cellular Localization | Ribosome |
|  | Nucleus |
|  | Cytoplasm |
|  | Organelle |
|  | Cell |


---

|  |  |
| --- | --- |
| Pathway | Master Regulators |
|  | Zn def RIN |
|  | Zn def DIN |


---

|  |  |
| --- | --- |
| GO Process | mRNA export from nucleus |
|  | transport |
|  | central nervous system development |
|  | mRNA processing |
|  | mRNA-nucleus export |
|  | regulation of transcription, DNA-dependent |
|  | regulation of translation |


---

|  |  |
| --- | --- |
| UniGene | Rn.40595 |
|  | Hs.103183 |
|  | Mm.3451 |


---

|  |  |
| --- | --- |
| Affymetrix Probeset ID | 104851\_at |
|  | 1393459\_at |
|  | 1423369\_at |
|  | 1426086\_a\_at |
|  | 1452550\_a\_at |
|  | 1561627\_at |
|  | 161875\_at |
|  | 203689\_s\_at |
|  | 215245\_x\_at |
|  | 37994\_at |
|  | 37995\_s\_at |
|  | 71147\_at |
|  | 74648\_at |
|  | 88124\_at |
|  | 98441\_at |
|  | Hs2.103183.1.S1\_3p\_at |
|  | Hs.89764.0.S1\_3p\_a\_at |
|  | L19493\_s\_at |
|  | L23971\_s\_at |
|  | M67468\_s\_at |
|  | U60145\_at |
|  | X69962\_s\_at |
|  | RC\_W73417\_at |


---

|  |  |
| --- | --- |
| GO Function | mRNA binding |
|  | RNA binding |
|  | nucleic acid binding |


---

|  |  |
| --- | --- |
| Nucleotide | L19484 |
|  | L19492 |
|  | NM\_052804 |
|  | X69962 |
|  | L19493 |
|  | NM\_008031 |
|  | AY630338 |
|  | AF435434 |
|  | L19478 |
|  | L23971 |
|  | L19481 |
|  | L19488 |
|  | AF170530 |
|  | L19476 |
|  | BC079671 |
|  | AK208300 |
|  | L19483 |
|  | AY240947 |
|  | L19487 |
|  | L19479 |
|  | AK046605 |
|  | AY630337 |
|  | AK080948 |
|  | AF461114 |
|  | AK053211 |
|  | S65791 |
|  | AK053701 |
|  | L19477 |
|  | BC038998 |
|  | L19485 |
|  | M67468 |
|  | AK080898 |
|  | AF251347 |
|  | L19486 |
|  | L19489 |
|  | S76590 |
|  | BC086957 |
|  | L19482 |
|  | L29074 |
|  | AB209188 |
|  | NM\_002024 |
|  | U60145 |
|  | L19480 |
|  | AK140343 |
|  | AK053829 |


---

|  |  |
| --- | --- |
| Protein | AAA62455 |
|  | AAH38998 |
|  | AAB28395 |
|  | NP\_032057 |
|  | AAB18833 |
|  | AAA62463 |
|  | BAD92425 |
|  | AAG44597 |
|  | AAT48081 |
|  | AAP15341 |
|  | AAA62462 |
|  | BAC35482 |
|  | AAA37635 |
|  | AAB18831 |
|  | AAA62452 |
|  | AAA62465 |
|  | AAB07073 |
|  | AAA62454 |
|  | BAC35545 |
|  | AAA62469 |
|  | AAL66364 |
|  | AAB18829 |
|  | AAA62468 |
|  | AAA62464 |
|  | NP\_002015 |
|  | AAA62453 |
|  | AAH86957 |
|  | AAA62461 |
|  | AAA52458 |
|  | Q06787 |
|  | AAA62460 |
|  | AAB18832 |
|  | CAA49586 |
|  | AAK56800 |
|  | AAA62458 |
|  | AAA62457 |
|  | AAD14228 |
|  | AAH79671 |
|  | AAB18830 |
|  | AAB18828 |
|  | AAT48080 |
|  | AAA62456 |
|  | NP\_434691 |
|  | AAL31971 |
|  | AAA62459 |
|  | P35922 |


---

|  |  |
| --- | --- |
| Organism | Mammal |


---

|  |  |
| --- | --- |
| Location | chromosome X, Xq27.3 (Homo sapiens) |
|  | X 24.5 cM (Mus musculus) |
|  | chromosome X, Xq37 (Rattus norvegicus) |
|  | chromosome X, X 24.5 cM, X A7.1 (Mus musculus) |


---

|  |  |
| --- | --- |
