# Supplementary material for: Dynamic transcriptomic profiles of zebrafish gills in response to zinc depletion
Source: BMC Genomics. 2010 Oct 8;11:548. doi: 10.1186/1471-2164-11-548 (PMC3091697; doi:10.1186/1471-2164-11-548)
Supplement: Additional file 2 — Figure S1 - Interactive Direct Interaction Network of responses to zinc depletion. Mini web-site containing index.html and hyperlinked pages in subdirectory. The web site is an interactive version of Figure 6A containing curated interactions between regulated genes and respective proteins. Legend: Molecular interactions between zinc and proteins encoded by genes changed under zinc depletion. A Direct Interaction Network was created based on curated interactions contained within the PathwayArchitect database and provided through hyperlinks. Red ovals represent proteins and the blue circle symbolizes Zn(II). Dark blue squares denote 'binding', and light blue squares 'expression'; green squares stand for 'regulation', green diamonds for 'metabolism', and green circles for 'promoter binding'. Arrow heads indicate directionality of the interaction where annotated. [file 1471-2164-11-548-S2.ZIP › PathwayArchitect Zn def DIN2/129519.html]

# PROTEIN: EIF4G2

|  |  |
| --- | --- |
| Name | EIF4G2 |
| Type | PROTEIN |
| Description | eukaryotic translation initiation factor 4 gamma, 2 |
| Note | Translation initiation is mediated by specific recognition of the cap structure by eukaryotic translation initiation factor 4F (eIF4F), which is a cap binding protein complex that consists of three subunits: eIF4A, eIF4E and eIF4G. The protein encoded by this gene shares similarity with the C-terminal region of eIF4G, that contains the binding sites for eIF4A and eIF3; eIF4G in addition, contains a binding site for eIF4E at the N-terminus. Unlike eIF4G which supports cap-dependent and independent translation, this gene product functions as a general repressor of translation by forming translationally inactive complexes. In vitro and in vivo studies indicate that translation of this mRNA initiates exclusively at a non-AUG (GUG) codon. |
| Alias | apobec-1 target 1 |
|  | death-associated protein 5 |
|  | NAT1 |
|  | p97 |
|  | DAP-5 |
|  | eukaryotic translation initiation factor 4G-like 1 |
|  | Nat1 |
|  | Death associated protein 5 |
|  | eIF-4G 2 |
|  | DAP5;ORFNames=OK/SW-cl.75 |
|  | translation repressor Nat1 |
|  | DAP5 |
|  | DRCF-6 |
|  | eIF4G 2 |
|  | MGC109314 |
|  | eIF-4-gamma 2 |
|  | Natm1 |
|  | EIF4G2 |


---

|  |  |
| --- | --- |
| GO Component | eukaryotic translation initiation factor 4F complex |
|  | cytoplasm |


---

|  |  |
| --- | --- |
| GO ID | GO:0003723 |
|  | GO:0008219 |
|  | GO:0005737 |
|  | GO:0007050 |
|  | GO:0006417 |
|  | GO:0003743 |
|  | GO:0006446 |
|  | GO:0006412 |
|  | GO:0006445 |
|  | GO:0016281 |


---

|  |  |
| --- | --- |
| MIM | MIM:602325 |


---

|  |  |
| --- | --- |
| Connectivity | 48 |


---

|  |  |
| --- | --- |
| Entrez ID | 171362 |
|  | 13690 |
|  | 1982 |


---

|  |  |
| --- | --- |
| Agilent ID | A\_53\_P106467 |
|  | A\_51\_P306066 |
|  | A\_14\_P107003 |
|  | A\_52\_P41668 |
|  | A\_23\_P104892 |
|  | A\_52\_P460526 |
|  | A\_14\_P103841 |
|  | A\_53\_P127024 |


---

|  |  |
| --- | --- |
| Cellular Localization | Cytoplasm |
|  | Cell |


---

|  |  |
| --- | --- |
| Pathway | Zn def RIN |
|  | Master Regulators |
|  | Zn xs inventory |
|  | Zn xs DIN |
|  | Zn xs RIN |
|  | Zn def DIN |


---

|  |  |
| --- | --- |
| GO Process | cell death |
|  | regulation of protein biosynthesis |
|  | cell cycle arrest |
|  | regulation of translational initiation |
|  | regulation of translation |
|  | protein biosynthesis |


---

|  |  |
| --- | --- |
| UniGene | Rn.103276 |
|  | Rn.121213 |
|  | Mm.185453 |
|  | Hs.183684 |


---

|  |  |
| --- | --- |
| Affymetrix Probeset ID | 240381\_at |
|  | 1415863\_at |
|  | 1452758\_s\_at |
|  | 1458431\_at |
|  | 1557964\_at |
|  | 165103\_f\_at |
|  | 200004\_at |
|  | 217607\_x\_at |
|  | 41785\_at |
|  | 1392715\_at |
|  | 80747\_at |
|  | 87748\_r\_at |
|  | 89110\_at |
|  | g4503538\_3p\_at |
|  | Hs.150904.0.S1\_3p\_at |
|  | Hs.150904.0.S1\_3p\_x\_at |
|  | Hs2.190503.1.S1\_3p\_at |
|  | u63323\_s\_at |
|  | U73824\_at |
|  | 1428363\_at |
|  | 1428362\_at |
|  | 100535\_at |
|  | 66990\_at |
|  | 68648\_at |
|  | 68650\_g\_at |
|  | 92014\_s\_at |
|  | 92017\_r\_at |
|  | 92018\_at |
|  | 92021\_g\_at |
|  | Hs.192440.0.A1\_3p\_at |
|  | 1388359\_at |
|  | TC33211\_at |
|  | TC33212\_at |
|  | TC33212\_g\_at |
|  | TC33213\_at |
|  | 1397520\_at |
|  | 1367469\_at |
|  | U95052UTR#1\_s\_at |
|  | rc\_AI101150\_at |
|  | rc\_AI179327\_at |


---

|  |  |
| --- | --- |
| GO Function | RNA binding |
|  | translation initiation factor activity |


---

|  |  |
| --- | --- |
| Nucleotide | AK151637 |
|  | BC039274 |
|  | BC039851 |
|  | BC057673 |
|  | BC092521 |
|  | BC040391 |
|  | AI874618 |
|  | BC056387 |
|  | BC010654 |
|  | U76111 |
|  | U95052 |
|  | BC043149 |
|  | NM\_001418 |
|  | U63323 |
|  | AK195045 |
|  | X89713 |
|  | AK223548 |
|  | BC018746 |
|  | AK144309 |
|  | BC018975 |
|  | AK141512 |
|  | BC065276 |
|  | NM\_001017374 |
|  | AB209267 |
|  | BC064810 |
|  | U73824 |
|  | BC091330 |
|  | BC014930 |
|  | AK150745 |
|  | BX647799 |
|  | AB063323 |
|  | BC043034 |
|  | U76112 |
|  | NM\_013507 |


---

|  |  |
| --- | --- |
| Protein | AAH10654 |
|  | AAH40391 |
|  | BAE30567 |
|  | CAA61857 |
|  | BAE24708 |
|  | BAD92504 |
|  | NP\_038535 |
|  | AAH64810 |
|  | NP\_001409 |
|  | AAH39851 |
|  | P78344 |
|  | AAH14930 |
|  | AAB49973 |
|  | NP\_001017374 |
|  | AAH43149 |
|  | AAC53095 |
|  | AAC53030 |
|  | BAE29817 |
|  | BAE25826 |
|  | AAH43034 |
|  | BAD97268 |
|  | Q62448 |
|  | AAH91330 |
|  | AAC51166 |
|  | BAB93515 |


---

|  |  |
| --- | --- |
| Organism | Mammal |


---

|  |  |
| --- | --- |
| Location | 7 51.52 cM (Mus musculus) |
|  | chromosome 7, 7 51.52 cM, 7 E3 (Mus musculus) |
|  | chromosome 1, 1q33 (Rattus norvegicus) |
|  | chromosome 11, 11p15 (Homo sapiens) |


---

|  |  |
| --- | --- |
