# Supplementary material for: Dynamic transcriptomic profiles of zebrafish gills in response to zinc depletion
Source: BMC Genomics. 2010 Oct 8;11:548. doi: 10.1186/1471-2164-11-548 (PMC3091697; doi:10.1186/1471-2164-11-548)
Supplement: Additional file 2 — Figure S1 - Interactive Direct Interaction Network of responses to zinc depletion. Mini web-site containing index.html and hyperlinked pages in subdirectory. The web site is an interactive version of Figure 6A containing curated interactions between regulated genes and respective proteins. Legend: Molecular interactions between zinc and proteins encoded by genes changed under zinc depletion. A Direct Interaction Network was created based on curated interactions contained within the PathwayArchitect database and provided through hyperlinks. Red ovals represent proteins and the blue circle symbolizes Zn(II). Dark blue squares denote 'binding', and light blue squares 'expression'; green squares stand for 'regulation', green diamonds for 'metabolism', and green circles for 'promoter binding'. Arrow heads indicate directionality of the interaction where annotated. [file 1471-2164-11-548-S2.ZIP › PathwayArchitect Zn def DIN2/130947.html]

# PROTEIN: ESRRA

|  |  |
| --- | --- |
| Name | ESRRA |
| Type | PROTEIN |
| Description | estrogen-related receptor alpha |
| Note | The protein encoded by this gene is a nuclear receptor that is closely related to the estrogen receptor. This protein acts as a site-specific transcription regulator and has been also shown to interact with estrogen and the transcripton factor TFIIB by direct protein-protein contact. The binding and regulatory activities of this protein have been demonstrated in the regulation of a variety of genes including lactoferrin, osteopontin, medium-chain acyl coenzyme A dehydrogenase (MCAD) and thyroid hormone receptor genes. A processed pseudogene of ESRRA is located on chromosome 13q12.1. |
| Alias | NR3B1 |
|  | ERRalpha |
|  | ESRRA |
|  | ERR- alpha |
|  | Estrogen receptor-like 1 |
|  | estrogen receptor-like 1 |
|  | estrogen receptor related 1 |
|  | Err1 |
|  | ERRa |
|  | orphan nuclear receptor |
|  | ERR1 |
|  | ESRL1 |
|  | Estrogen-related receptor, alpha |
|  | Estrra |
|  | Fragment |
|  | Nr3b1 |
|  | Errra |
|  | Esrra |


---

|  |  |
| --- | --- |
| GO Component | nucleus |
|  | membrane |


---

|  |  |
| --- | --- |
| GO ID | GO:0003707 |
|  | GO:0006810 |
|  | GO:0016020 |
|  | GO:0005634 |
|  | GO:0003677 |
|  | GO:0030284 |
|  | GO:0006355 |
|  | GO:0004872 |
|  | GO:0004879 |
|  | GO:0045669 |
|  | GO:0046872 |
|  | GO:0006350 |
|  | GO:0005496 |
|  | GO:0003700 |
|  | GO:0005215 |


---

|  |  |
| --- | --- |
| MIM | MIM:601998 |


---

|  |  |
| --- | --- |
| Connectivity | 154 |


---

|  |  |
| --- | --- |
| Entrez ID | 293701 |
|  | 2101 |
|  | 26379 |


---

|  |  |
| --- | --- |
| Agilent ID | A\_32\_P232445 |
|  | A\_53\_P145671 |
|  | A\_51\_P248580 |
|  | A\_32\_P372584 |
|  | A\_32\_P164352 |
|  | A\_53\_P144711 |
|  | A\_42\_P780882 |
|  | A\_53\_P160896 |
|  | A\_23\_P1585 |
|  | A\_32\_P538008 |
|  | A\_52\_P198949 |
|  | A\_14\_P105431 |


---

|  |  |
| --- | --- |
| Cellular Localization | Membrane |
|  | Nucleus |
|  | Cell |
|  | Organelle |


---

|  |  |
| --- | --- |
| Pathway | Zn def RIN |
|  | Zn def DIN |


---

|  |  |
| --- | --- |
| GO Process | transport |
|  | positive regulation of osteoblast differentiation |
|  | regulation of transcription, DNA-dependent |
|  | transcription |


---

|  |  |
| --- | --- |
| UniGene | Mm.371597 |
|  | Rn.2190 |
|  | Hs.110849 |


---

|  |  |
| --- | --- |
| Affymetrix Probeset ID | 102145\_f\_at |
|  | 103964\_at |
|  | 1374741\_at |
|  | 1460652\_at |
|  | 1487\_at |
|  | 203193\_at |
|  | g4758305\_3p\_s\_at |
|  | L38487\_at |
|  | rc\_AA799412\_at |
|  | rc\_AA799412\_g\_at |
|  | rc\_AI171726\_at |
|  | U85259\_at |
|  | 1442864\_at |


---

|  |  |
| --- | --- |
| GO Function | ligand-dependent nuclear receptor activity |
|  | transporter activity |
|  | DNA binding |
|  | estrogen receptor activity |
|  | transcription factor activity |
|  | steroid hormone receptor activity |
|  | steroid binding |
|  | receptor activity |
|  | metal ion binding |


---

|  |  |
| --- | --- |
| Nucleotide | X51416 |
|  | BC093720 |
|  | NM\_004451 |
|  | AK019222 |
|  | AK156371 |
|  | BC093722 |
|  | BC007915 |
|  | NM\_007953 |
|  | NM\_001008511 |
|  | BC063795 |
|  | BC033701 |
|  | U85259 |
|  | AY280663 |
|  | L38487 |
|  | AK200205 |
|  | BC092470 |
|  | BC011528 |
|  | AK192324 |
|  | BC039774 |
|  | AK008536 |
|  | AK046588 |


---

|  |  |
| --- | --- |
| Protein | NP\_004442 |
|  | AAQ17212 |
|  | CAA35778 |
|  | AAH93722 |
|  | NP\_031979 |
|  | AAH07915 |
|  | AAH92470 |
|  | AAB17015 |
|  | AAH11528 |
|  | P11474 |
|  | AAB51250 |
|  | AAH93720 |
|  | NP\_001008511 |
|  | AAH63795 |
|  | AAH33701 |
|  | BAE33690 |
|  | O08580 |


---

|  |  |
| --- | --- |
| Organism | Mammal |


---

|  |  |
| --- | --- |
| Location | chromosome 19, 19 3.0 cM, 19 A (Mus musculus) |
|  | chromosome 11, 11q13 (Homo sapiens) |
|  | chromosome 1, 1q43 (Rattus norvegicus) |
|  | 19 3.0 cM (Mus musculus) |


---

|  |  |
| --- | --- |
