# Supplementary material for: Dynamic transcriptomic profiles of zebrafish gills in response to zinc depletion
Source: BMC Genomics. 2010 Oct 8;11:548. doi: 10.1186/1471-2164-11-548 (PMC3091697; doi:10.1186/1471-2164-11-548)
Supplement: Additional file 2 — Figure S1 - Interactive Direct Interaction Network of responses to zinc depletion. Mini web-site containing index.html and hyperlinked pages in subdirectory. The web site is an interactive version of Figure 6A containing curated interactions between regulated genes and respective proteins. Legend: Molecular interactions between zinc and proteins encoded by genes changed under zinc depletion. A Direct Interaction Network was created based on curated interactions contained within the PathwayArchitect database and provided through hyperlinks. Red ovals represent proteins and the blue circle symbolizes Zn(II). Dark blue squares denote 'binding', and light blue squares 'expression'; green squares stand for 'regulation', green diamonds for 'metabolism', and green circles for 'promoter binding'. Arrow heads indicate directionality of the interaction where annotated. [file 1471-2164-11-548-S2.ZIP › PathwayArchitect Zn def DIN2/135170.html]

# PROTEIN: DFFB

|  |  |
| --- | --- |
| Name | DFFB |
| Type | PROTEIN |
| Description | DNA fragmentation factor, 40kDa, beta polypeptide (caspase-activated DNase) |
| Note | Apoptosis is a cell death process that removes toxic and/or useless cells during mammalian development. The apoptotic process is accompanied by shrinkage and fragmentation of the cells and nuclei and degradation of the chromosomal DNA into nucleosomal units. DNA fragmentation factor (DFF) is a heterodimeric protein of 40-kD (DFFB) and 45-kD (DFFA) subunits. DFFA is the substrate for caspase-3 and triggers DNA fragmentation during apoptosis. DFF becomes activated when DFFA is cleaved by caspase-3. The cleaved fragments of DFFA dissociate from DFFB, the active component of DFF. DFFB has been found to trigger both DNA fragmentation and chromatin condensation during apoptosis. Multiple alternatively spliced transcript variants encoding distinct isoforms have been found for this gene, but the biological validity of some variants has not been determined. |
| Alias | CAD |
|  | DFF40 |
|  | DNA fragmentation factor, 40 kD, beta polypeptide (caspase-activated DNase) |
|  | DNA fragmentation factor, 40 kD, beta polypeptide |
|  | DFF-40 |
|  | 40kDa |
|  | DFF2 |
|  | Didff |
|  | caspase-activated deoxyribonuclease |
|  | DNase inhibited by DNA fragmentation factor |
|  | 5730477D02Rik |
|  | DNA fragmentation factor 40 kD beta polypeptide (caspase-activated DNase) |
|  | caspase-activated nuclease |
|  | caspase-activated DNase |
|  | DNA fragmentation factor, 40 kD, beta subunit |
|  | Cad |
|  | CPAN |


---

|  |  |
| --- | --- |
| GO Component | cytosol |
|  | intracellular |
|  | nucleus |


---

|  |  |
| --- | --- |
| GO ID | GO:0005634 |
|  | GO:0003677 |
|  | GO:0004536 |
|  | GO:0005622 |
|  | GO:0006915 |
|  | GO:0004518 |
|  | GO:0004537 |
|  | GO:0005515 |
|  | GO:0004516 |
|  | GO:0016787 |
|  | GO:0005829 |
|  | GO:0006917 |
|  | GO:0007242 |
|  | GO:0006309 |


---

|  |  |
| --- | --- |
| MIM | MIM:601883 |


---

|  |  |
| --- | --- |
| Connectivity | 85 |


---

|  |  |
| --- | --- |
| Entrez ID | 84359 |
|  | 1677 |
|  | 13368 |


---

|  |  |
| --- | --- |
| Agilent ID | A\_43\_P12784 |
|  | A\_23\_P44546 |
|  | A\_44\_P266801 |
|  | A\_53\_P156581 |
|  | A\_51\_P233002 |
|  | A\_24\_P278684 |
|  | A\_14\_P106949 |
|  | A\_52\_P46742 |
|  | A\_53\_P105853 |
|  | A\_14\_P201751 |
|  | A\_14\_P108932 |
|  | A\_24\_P370626 |


---

|  |  |
| --- | --- |
| Cellular Localization | Nucleus |
|  | Cytosol |
|  | Cell |
|  | Cytoplasm |
|  | Organelle |


---

|  |  |
| --- | --- |
| DbXref | KEGG pathway##04210##Apoptosis##http://www.genome.jp/dbget-bin/show\_pathway?rno04210+84359 |
|  | KEGG pathway##04210##Apoptosis##http://www.genome.jp/dbget-bin/show\_pathway?mmu04210+13368 |
|  | KEGG pathway##04210##Apoptosis##http://www.genome.jp/dbget-bin/show\_pathway?hsa04210+1677 |


---

|  |  |
| --- | --- |
| Pathway | FAS Signaling |
|  | Death Receptor Signaling |
|  | Caspase Signaling |
|  | Zn def RIN |
|  | Zn def DIN |


---

|  |  |
| --- | --- |
| GO Process | intracellular signaling cascade |
|  | DNA fragmentation during apoptosis |
|  | apoptosis |
|  | induction of apoptosis |


---

|  |  |
| --- | --- |
| UniGene | Rn.67077 |
|  | Hs.133089 |
|  | Mm.86386 |


---

|  |  |
| --- | --- |
| Affymetrix Probeset ID | 100294\_at |
|  | 1387604\_at |
|  | 1421229\_at |
|  | 1437051\_at |
|  | 166801\_at |
|  | 206752\_s\_at |
|  | 34937\_at |
|  | g4758149\_3p\_s\_at |
|  | 137602\_at |
|  | TC35041\_at |


---

|  |  |
| --- | --- |
| GO Function | hydrolase activity |
|  | protein binding |
|  | nicotinate phosphoribosyltransferase activity |
|  | deoxyribonuclease activity |
|  | DNA binding |
|  | caspase-activated deoxyribonuclease activity |
|  | nuclease activity |


---

|  |  |
| --- | --- |
| Nucleotide | AF409061 |
|  | AB028912 |
|  | AF064019 |
|  | AF039210 |
|  | AB009377 |
|  | AK036094 |
|  | AL691523 |
|  | NM\_007859 |
|  | AF409062 |
|  | AB013918 |
|  | NM\_004402 |
|  | AF136598 |
|  | AK089267 |
|  | AB028913 |
|  | AI426042 |
|  | BC032827 |
|  | NM\_001004286 |
|  | AF409060 |
|  | BC048797 |
|  | AB028911 |
|  | AY438268 |
|  | AK077619 |
|  | NM\_053362 |
|  | BC053052 |
|  | NM\_001004285 |
|  | AY438267 |


---

|  |  |
| --- | --- |
| Protein | AAK16646 |
|  | O76075 |
|  | NP\_445814 |
|  | AAL02006 |
|  | CAI17370 |
|  | O54788 |
|  | BAB40449 |
|  | BAB40448 |
|  | BAC36905 |
|  | NP\_004393 |
|  | AAR05821 |
|  | AAH32827 |
|  | BAB40447 |
|  | AAL02005 |
|  | AAR05822 |
|  | Q99N34 |
|  | NP\_031885 |
|  | AAH48797 |
|  | AAC39920 |
|  | AAL02007 |
|  | CAI17371 |
|  | BAA32250 |
|  | NP\_001004285 |
|  | NP\_001004286 |
|  | BAA24977 |
|  | AAH53052 |
|  | AAC39709 |


---

|  |  |
| --- | --- |
| Organism | Mammal |


---

|  |  |
| --- | --- |
| Location | chromosome 1, 1p36.3 (Homo sapiens) |
|  | chromosome 4, 4 E2 (Mus musculus) |
|  | chromosome 5, 5q36 (Rattus norvegicus) |


---

|  |  |
| --- | --- |
