# Supplementary material for: Dynamic transcriptomic profiles of zebrafish gills in response to zinc depletion
Source: BMC Genomics. 2010 Oct 8;11:548. doi: 10.1186/1471-2164-11-548 (PMC3091697; doi:10.1186/1471-2164-11-548)
Supplement: Additional file 2 — Figure S1 - Interactive Direct Interaction Network of responses to zinc depletion. Mini web-site containing index.html and hyperlinked pages in subdirectory. The web site is an interactive version of Figure 6A containing curated interactions between regulated genes and respective proteins. Legend: Molecular interactions between zinc and proteins encoded by genes changed under zinc depletion. A Direct Interaction Network was created based on curated interactions contained within the PathwayArchitect database and provided through hyperlinks. Red ovals represent proteins and the blue circle symbolizes Zn(II). Dark blue squares denote 'binding', and light blue squares 'expression'; green squares stand for 'regulation', green diamonds for 'metabolism', and green circles for 'promoter binding'. Arrow heads indicate directionality of the interaction where annotated. [file 1471-2164-11-548-S2.ZIP › PathwayArchitect Zn def DIN2/136413.html]

# PROTEIN: SLC22A7

|  |  |
| --- | --- |
| Name | SLC22A7 |
| Type | PROTEIN |
| Description | solute carrier family 22 (organic anion transporter), member 7 |
| Note | The protein encoded by this gene is involved in the sodium-independent transport and excretion of organic anions, some of which are potentially toxic. The encoded protein is an integral membrane protein and appears to be localized to the basolateral membrane of the kidney. Two transcript variants encoding different isoforms have been found for this gene. |
| Alias | solute carrier family 22 member 7 |
|  | liver-specific transporter |
|  | organic anion transporter 2 |
|  | MGC45202 |
|  | OAT2 |
|  | Oat2 |
|  | MGC18877 |
|  | MGC24091 |
|  | NLT |


---

|  |  |
| --- | --- |
| GO Component | integral to membrane |
|  | integral to plasma membrane |
|  | membrane fraction |
|  | membrane |


---

|  |  |
| --- | --- |
| GO ID | GO:0016020 |
|  | GO:0005524 |
|  | GO:0015075 |
|  | GO:0031402 |
|  | GO:0015293 |
|  | GO:0005887 |
|  | GO:0000166 |
|  | GO:0016021 |
|  | GO:0005624 |
|  | GO:0006814 |
|  | GO:0006810 |
|  | GO:0015711 |
|  | GO:0006811 |
|  | GO:0008514 |
|  | GO:0015347 |
|  | GO:0005215 |


---

|  |  |
| --- | --- |
| MIM | MIM:604995 |


---

|  |  |
| --- | --- |
| Connectivity | 42 |


---

|  |  |
| --- | --- |
| Entrez ID | 108114 |
|  | 10864 |
|  | 89776 |


---

|  |  |
| --- | --- |
| Agilent ID | A\_53\_P152530 |
|  | A\_42\_P554831 |
|  | A\_23\_P93217 |
|  | A\_53\_P150622 |
|  | A\_23\_P93213 |
|  | A\_14\_P123299 |
|  | A\_44\_P318674 |
|  | A\_51\_P395856 |
|  | A\_24\_P243560 |


---

|  |  |
| --- | --- |
| Cellular Localization | Membrane |
|  | Cell |
|  | Plasma membrane |


---

|  |  |
| --- | --- |
| Pathway | Zn def RIN |
|  | Master Regulators |
|  | Zn def DIN |


---

|  |  |
| --- | --- |
| GO Process | transport |
|  | sodium ion transport |
|  | ion transport |
|  | organic anion transport |


---

|  |  |
| --- | --- |
| UniGene | Hs.485438 |
|  | Mm.100765 |
|  | Rn.10009 |


---

|  |  |
| --- | --- |
| Affymetrix Probeset ID | 1398267\_at |
|  | 1451460\_a\_at |
|  | 1555553\_3p\_a\_at |
|  | 1555553\_a\_at |
|  | 220554\_at |
|  | 221661\_at |
|  | 221662\_s\_at |
|  | 231398\_at |
|  | 62538\_at |
|  | g12003292\_3p\_at |
|  | g5730048\_3p\_at |
|  | Hs2.251395.4.S1\_3p\_at |
|  | Hs2.251395.4.S1\_3p\_s\_at |
|  | Hs.6858.0.A1\_3p\_at |
|  | L27651\_at |
|  | L27651\_g\_at |
|  | RC\_N74422\_at |


---

|  |  |
| --- | --- |
| GO Function | ion transporter activity |
|  | transporter activity |
|  | nucleotide binding |
|  | ATP binding |
|  | sodium ion binding |
|  | symporter activity |
|  | organic anion transporter activity |
|  | sodium-independent organic anion transporter activity |


---

|  |  |
| --- | --- |
| Nucleotide | AF097518 |
|  | BC033805 |
|  | AY816233 |
|  | L27651 |
|  | NM\_053537 |
|  | BC026598 |
|  | BC013474 |
|  | NM\_144856 |
|  | NM\_153320 |
|  | AI928177 |
|  | AK143949 |
|  | AB069965 |
|  | BC026597 |
|  | BC017963 |
|  | BC025813 |
|  | NM\_006672 |
|  | AF210455 |
|  | AK082865 |
|  | AL583834 |
|  | AY050498 |
|  | BC024119 |


---

|  |  |
| --- | --- |
| Protein | AAG43523 |
|  | CAI14463 |
|  | NP\_445989 |
|  | AAD37091 |
|  | AAA57157 |
|  | AAH24119 |
|  | NP\_006663 |
|  | AAH33805 |
|  | CAI14464 |
|  | AAH25813 |
|  | CAI14462 |
|  | AAL12496 |
|  | BAC38659 |
|  | CAI14460 |
|  | CAI14466 |
|  | AAH17963 |
|  | AAH13474 |
|  | AAH26597 |
|  | BAE25625 |
|  | CAI14465 |
|  | AAV66454 |
|  | AAH26598 |
|  | NP\_659105 |
|  | NP\_696961 |
|  | BAC02736 |


---

|  |  |
| --- | --- |
| Organism | Mammal |


---

|  |  |
| --- | --- |
| Location | chromosome 9, 9q12 (Rattus norvegicus) |
|  | chromosome 17 (Mus musculus) |
|  | chromosome 6, 6p21.2-p21.1 (Homo sapiens) |


---

|  |  |
| --- | --- |
