# Supplementary material for: Dynamic transcriptomic profiles of zebrafish gills in response to zinc depletion
Source: BMC Genomics. 2010 Oct 8;11:548. doi: 10.1186/1471-2164-11-548 (PMC3091697; doi:10.1186/1471-2164-11-548)
Supplement: Additional file 2 — Figure S1 - Interactive Direct Interaction Network of responses to zinc depletion. Mini web-site containing index.html and hyperlinked pages in subdirectory. The web site is an interactive version of Figure 6A containing curated interactions between regulated genes and respective proteins. Legend: Molecular interactions between zinc and proteins encoded by genes changed under zinc depletion. A Direct Interaction Network was created based on curated interactions contained within the PathwayArchitect database and provided through hyperlinks. Red ovals represent proteins and the blue circle symbolizes Zn(II). Dark blue squares denote 'binding', and light blue squares 'expression'; green squares stand for 'regulation', green diamonds for 'metabolism', and green circles for 'promoter binding'. Arrow heads indicate directionality of the interaction where annotated. [file 1471-2164-11-548-S2.ZIP › PathwayArchitect Zn def DIN2/136968.html]

# PROTEIN: ZNF235

|  |  |
| --- | --- |
| Name | ZNF235 |
| Type | PROTEIN |
| Description | zinc finger protein 235 |
| Note | This gene product belongs to the zinc finger protein superfamily, members of which are regulatory proteins characterized by nucleic acid-binding zinc finger domains. The encoded protein is a member of the Kruppel family of zinc finger proteins, and contains Kruppel-associated box (KRAB) A and B domains and 15 tandemly arrayed C2H2-type zinc fingers. It is an ortholog of the mouse Zfp93 protein. This gene is located in a cluster of zinc finger genes on 19q13.2. |
| Alias | ZFP93 |
|  | Zinc finger protein HZF6 |
|  | zinc finger protein 93 homolog (mouse) |
|  | ANF270 |
|  | ZNF235 |
|  | HZF6 |
|  | zinc finger protein homologous to mouse Zfp93 |
|  | Zfp-93 |
|  | zinc finger protein homologous to Zfp93 in mouse |
|  | zinc finger protein 93 homolog |
|  | Fragment |
|  | ZNF270 |
|  | Zinc finger protein 93 homolog |


---

|  |  |
| --- | --- |
| MIM | MIM:604749 |


---

|  |  |
| --- | --- |
| Connectivity | 4 |


---

|  |  |
| --- | --- |
| Entrez ID | 9310 |


---

|  |  |
| --- | --- |
| Agilent ID | A\_23\_P208325 |
|  | A\_14\_P123934 |
|  | A\_24\_P151148 |


---

|  |  |
| --- | --- |
| Pathway | Zn def RIN |
|  | Master Regulators |
|  | Zn def DIN |


---

|  |  |
| --- | --- |
| UniGene | Hs.371335 |
|  | Hs.298089 |


---

|  |  |
| --- | --- |
| Affymetrix Probeset ID | 1560775\_at |
|  | 210595\_at |
|  | 220350\_at |
|  | 79695\_at |
|  | g12056481\_3p\_at |
|  | g12803656\_3p\_at |
|  | 69332\_at |
|  | Hs2.255363.1.S1\_3p\_at |


---

|  |  |
| --- | --- |
| Nucleotide | NM\_004234 |
|  | BC002663 |
|  | AF027514 |
|  | AC084239 |
|  | AF027513 |
|  | BC002800 |
|  | X78929 |


---

|  |  |
| --- | --- |
| Protein | Q14590 |
|  | NP\_004225 |
|  | AAG23969 |
|  | CAA55529 |
|  | AAD12729 |
|  | AAD12728 |


---

|  |  |
| --- | --- |
| Organism | Mammal |


---

|  |  |
| --- | --- |
| Location | chromosome 19, 19q13.2 (Homo sapiens) |


---

|  |  |
| --- | --- |
