# Supplementary material for: Dynamic transcriptomic profiles of zebrafish gills in response to zinc depletion
Source: BMC Genomics. 2010 Oct 8;11:548. doi: 10.1186/1471-2164-11-548 (PMC3091697; doi:10.1186/1471-2164-11-548)
Supplement: Additional file 2 — Figure S1 - Interactive Direct Interaction Network of responses to zinc depletion. Mini web-site containing index.html and hyperlinked pages in subdirectory. The web site is an interactive version of Figure 6A containing curated interactions between regulated genes and respective proteins. Legend: Molecular interactions between zinc and proteins encoded by genes changed under zinc depletion. A Direct Interaction Network was created based on curated interactions contained within the PathwayArchitect database and provided through hyperlinks. Red ovals represent proteins and the blue circle symbolizes Zn(II). Dark blue squares denote 'binding', and light blue squares 'expression'; green squares stand for 'regulation', green diamonds for 'metabolism', and green circles for 'promoter binding'. Arrow heads indicate directionality of the interaction where annotated. [file 1471-2164-11-548-S2.ZIP › PathwayArchitect Zn def DIN2/137268.html]

# PROTEIN: CDC14A

|  |  |
| --- | --- |
| Name | CDC14A |
| Type | PROTEIN |
| Description | CDC14 cell division cycle 14 homolog A (S. cerevisiae) |
| Note | The protein encoded by this gene is a member of the dual specificity protein tyrosine phosphatase family. This protein is highly similar to Saccharomyces cerevisiae Cdc14, a protein tyrosine phosphatase involved in the exit of cell mitosis and initiation of DNA replication, which suggests the role in cell cycle control. This protein has been shown to interact with and dephosphorylates tumor suppressor protein p53, and is thought to regulate the function of p53. Alternative splice of this gene results in 3 transcript variants encoding distinct isoforms. |
| Alias | CDC14a1 |
|  | hCDC14 |
|  | Cdc14 |
|  | Cdc14A1 |
|  | CDC14 cell division cycle 14 homolog A |
|  | CDC10 (cell division cycle 10, S. cerevisiae, homolog) |
|  | Cdc14a |
|  | A830059A17Rik |
|  | Cdc14A2 |
|  | CDC14A2 |
|  | cdc14 |
|  | CDC14A |
|  | CDC14 homolog A |


---

|  |  |
| --- | --- |
| GO Component | nucleus |


---

|  |  |
| --- | --- |
| GO ID | GO:0005634 |
|  | GO:0000074 |
|  | GO:0004725 |
|  | GO:0008283 |
|  | GO:0016787 |
|  | GO:0004721 |
|  | GO:0008138 |
|  | GO:0051301 |
|  | GO:0006470 |
|  | GO:0007049 |


---

|  |  |
| --- | --- |
| MIM | MIM:603504 |


---

|  |  |
| --- | --- |
| Connectivity | 15 |


---

|  |  |
| --- | --- |
| Entrez ID | 8556 |
|  | 229776 |


---

|  |  |
| --- | --- |
| Agilent ID | A\_14\_P110107 |
|  | A\_14\_P128130 |
|  | A\_14\_P102692 |
|  | A\_52\_P538145 |
|  | A\_23\_P405110 |
|  | A\_23\_P201921 |
|  | A\_14\_P131083 |
|  | A\_23\_P424472 |
|  | A\_52\_P319726 |
|  | A\_52\_P325900 |
|  | A\_52\_P16919 |
|  | A\_51\_P489560 |


---

|  |  |
| --- | --- |
| Cellular Localization | Nucleus |
|  | Organelle |
|  | Cell |


---

|  |  |
| --- | --- |
| DbXref | KEGG pathway##04110##Cell cycle##http://www.genome.jp/dbget-bin/show\_pathway?mmu04110+229776 |
|  | KEGG pathway##04110##Cell cycle##http://www.genome.jp/dbget-bin/show\_pathway?hsa04110+8556 |


---

|  |  |
| --- | --- |
| Pathway | Zn def RIN |
|  | Master Regulators |
|  | Zn xs inventory |
|  | Zn xs DIN |
|  | Zn def DIN |


---

|  |  |
| --- | --- |
| GO Process | cell proliferation |
|  | protein amino acid dephosphorylation |
|  | cell cycle |
|  | regulation of progression through cell cycle |
|  | cell division |


---

|  |  |
| --- | --- |
| UniGene | Mm.17647 |
|  | Hs.533582 |


---

|  |  |
| --- | --- |
| Affymetrix Probeset ID | 115348\_i\_at |
|  | 115349\_r\_at |
|  | 1436913\_at |
|  | 1443184\_at |
|  | 1446493\_at |
|  | 1459517\_at |
|  | 1567303\_at |
|  | 1567304\_at |
|  | 205288\_at |
|  | 210440\_s\_at |
|  | 210441\_at |
|  | 210742\_at |
|  | 210743\_s\_at |
|  | 243640\_x\_at |
|  | 40320\_at |
|  | 72539\_at |
|  | 80535\_r\_at |
|  | 87577\_at |
|  | g3136327\_3p\_a\_at |
|  | g3136327\_3p\_at |
|  | g3136329\_3p\_at |
|  | g4502696\_3p\_at |
|  | Hs2.385268.1.S1\_3p\_at |
|  | Hs.271443.0.A1\_3p\_at |
|  | RC\_AA002017\_at |
|  | RC\_AA283949\_at |
|  | TC38094\_at |


---

|  |  |
| --- | --- |
| EC Number | EC 3.1.3.16 |
|  | EC 3.1.3.48 |


---

|  |  |
| --- | --- |
| GO Function | hydrolase activity |
|  | protein tyrosine phosphatase activity |
|  | protein tyrosine/serine/threonine phosphatase activity |
|  | phosphoprotein phosphatase activity |


---

|  |  |
| --- | --- |
| Nucleotide | AF064103 |
|  | AL589990 |
|  | AK142294 |
|  | AB209857 |
|  | AK036556 |
|  | NM\_033312 |
|  | BC093918 |
|  | BC072644 |
|  | NM\_003672 |
|  | AF064102 |
|  | AI047562 |
|  | AK048250 |
|  | AK043956 |
|  | AK037269 |
|  | XM\_149387 |
|  | AK032515 |
|  | AK052560 |
|  | BC038979 |
|  | BC093916 |
|  | AF000367 |
|  | NM\_033313 |
|  | AY623111 |
|  | AF122013 |


---

|  |  |
| --- | --- |
| Protein | AAD49217 |
|  | BAD93094 |
|  | AAT38107 |
|  | AAH38979 |
|  | NP\_201569 |
|  | Q6GQT0 |
|  | Q9UNH5 |
|  | AAC16660 |
|  | AAB88277 |
|  | AAH93918 |
|  | CAH70070 |
|  | AAC16659 |
|  | BAC29476 |
|  | AAH72644 |
|  | XP\_149387 |
|  | NP\_201570 |
|  | NP\_003663 |
|  | CAH70069 |
|  | CAH70068 |
|  | AAH93916 |


---

|  |  |
| --- | --- |
| Organism | Mammal |


---

|  |  |
| --- | --- |
| Location | chromosome 3, 3 G1 (Mus musculus) |
|  | chromosome 1, 1p21 (Homo sapiens) |


---

|  |  |
| --- | --- |
