# Supplementary material for: Dynamic transcriptomic profiles of zebrafish gills in response to zinc depletion
Source: BMC Genomics. 2010 Oct 8;11:548. doi: 10.1186/1471-2164-11-548 (PMC3091697; doi:10.1186/1471-2164-11-548)
Supplement: Additional file 2 — Figure S1 - Interactive Direct Interaction Network of responses to zinc depletion. Mini web-site containing index.html and hyperlinked pages in subdirectory. The web site is an interactive version of Figure 6A containing curated interactions between regulated genes and respective proteins. Legend: Molecular interactions between zinc and proteins encoded by genes changed under zinc depletion. A Direct Interaction Network was created based on curated interactions contained within the PathwayArchitect database and provided through hyperlinks. Red ovals represent proteins and the blue circle symbolizes Zn(II). Dark blue squares denote 'binding', and light blue squares 'expression'; green squares stand for 'regulation', green diamonds for 'metabolism', and green circles for 'promoter binding'. Arrow heads indicate directionality of the interaction where annotated. [file 1471-2164-11-548-S2.ZIP › PathwayArchitect Zn def DIN2/138105.html]

# PROTEIN: WT1

|  |  |
| --- | --- |
| Name | WT1 |
| Type | PROTEIN |
| Description | Wilms tumor 1 |
| Note | This gene encodes a transcription factor that contains four zinc-finger motifs at the C-terminus and a proline/glutamine-rich DNA-binding domain at the N-terminus. It has an essential role in the normal development of the urogenital system, and it is mutated in a small subset of patients with Wilm's tumors. Multiple transcript variants, resulting from alternative splicing at two coding exons, have been well characterized. There is also evidence for the use of non-AUG (CUG) translation initiation site upstream of, and in-frame with the first AUG, leading to additional isoforms. |
| Alias | GUD |
|  | WT33 |
|  | WAGR |
|  | WIT-2 |
|  | Wilms' tumor protein homolog |
|  | D630046I19Rik |
|  | WT1 |
|  | Wt-1 |
|  | Wt1 |


---

|  |  |
| --- | --- |
| GO Component | nucleus |
|  | cytoplasm |


---

|  |  |
| --- | --- |
| GO ID | GO:0003677 |
|  | GO:0045786 |
|  | GO:0006355 |
|  | GO:0009888 |
|  | GO:0005737 |
|  | GO:0008584 |
|  | GO:0006357 |
|  | GO:0005515 |
|  | GO:0008270 |
|  | GO:0001747 |
|  | GO:0003700 |
|  | GO:0001823 |
|  | GO:0001656 |
|  | GO:0005634 |
|  | GO:0001654 |
|  | GO:0003676 |
|  | GO:0007281 |
|  | GO:0046872 |
|  | GO:0006350 |
|  | GO:0007049 |
|  | GO:0030855 |


---

|  |  |
| --- | --- |
| MIM | MIM:194070 |
|  | MIM:607102 |
|  | MIM:256370 |
|  | MIM:194080 |
|  | MIM:194072 |
|  | MIM:136680 |


---

|  |  |
| --- | --- |
| Connectivity | 473 |


---

|  |  |
| --- | --- |
| Entrez ID | 7490 |
|  | 22431 |
|  | 24883 |


---

|  |  |
| --- | --- |
| Agilent ID | A\_43\_P12576 |
|  | A\_14\_P123086 |
|  | A\_51\_P360809 |
|  | A\_44\_P176466 |
|  | A\_52\_P673458 |
|  | A\_53\_P136459 |
|  | A\_14\_P114899 |
|  | A\_23\_P116280 |
|  | A\_53\_P100038 |


---

|  |  |
| --- | --- |
| Cellular Localization | Cytoplasm |
|  | Nucleus |
|  | Cell |
|  | Organelle |


---

|  |  |
| --- | --- |
| Pathway | Zn def RIN |
|  | Zn xs inventory |
|  | Zn xs DIN |
|  | Zn xs RIN |
|  | Zn def DIN |


---

|  |  |
| --- | --- |
| GO Process | male gonad development |
|  | negative regulation of progression through cell cycle |
|  | germ cell development |
|  | tissue development |
|  | eye development (sensu Mammalia) |
|  | regulation of transcription, DNA-dependent |
|  | metanephros development |
|  | regulation of transcription from RNA polymerase II promoter |
|  | transcription |
|  | eye development |
|  | cell cycle |
|  | mesonephros development |
|  | epithelial cell differentiation |


---

|  |  |
| --- | --- |
| UniGene | Mm.246679 |
|  | Rn.92531 |
|  | Hs.555896 |


---

|  |  |
| --- | --- |
| Affymetrix Probeset ID | 135883\_at |
|  | 1369695\_at |
|  | 1425995\_s\_at |
|  | 1443221\_at |
|  | 1500\_at |
|  | 161258\_at |
|  | 1684\_s\_at |
|  | 206067\_s\_at |
|  | 216953\_s\_at |
|  | 93856\_at |
|  | g13386509\_3p\_a\_at |
|  | Hs.1145.2.S1\_3p\_a\_at |
|  | 1377223\_at |
|  | Msa.1292.0\_at |
|  | Msa.1292.0\_g\_at |
|  | rc\_AA965119\_at |
|  | S63358\_s\_at |
|  | X51630\_at |
|  | X69716\_at |
|  | RC\_AA130187\_s\_at |
|  | rc\_AA899753\_at |


---

|  |  |
| --- | --- |
| GO Function | protein binding |
|  | transcription factor activity |
|  | DNA binding |
|  | zinc ion binding |
|  | nucleic acid binding |
|  | metal ion binding |


---

|  |  |
| --- | --- |
| Nucleotide | U06486 |
|  | S61515 |
|  | AK052767 |
|  | X72314 |
|  | X69716 |
|  | NM\_024425 |
|  | NM\_031534 |
|  | NM\_000378 |
|  | BC032861 |
|  | AL049692 |
|  | AK093168 |
|  | NM\_024426 |
|  | NM\_024424 |
|  | S77896 |
|  | X74840 |
|  | U77682 |
|  | X51630 |
|  | X77549 |
|  | L25110 |
|  | NM\_144783 |
|  | S60755 |
|  | AY245105 |
|  | M30393 |
|  | AK013905 |
|  | S75264 |
|  | BC046461 |
|  | M80217 |
|  | X61631 |
|  | M55512 |


---

|  |  |
| --- | --- |
| Protein | P22561 |
|  | CAI95760 |
|  | P49952 |
|  | AAH32861 |
|  | NP\_659032 |
|  | CAC39220 |
|  | AAA40573 |
|  | CAA35956 |
|  | AAC60605 |
|  | NP\_077743 |
|  | P19544 |
|  | CAI95759 |
|  | AAB20110 |
|  | CAA51057 |
|  | CAA43819 |
|  | AAD14879 |
|  | CAI95758 |
|  | AAB33443 |
|  | NP\_113722 |
|  | NP\_077744 |
|  | NP\_000369 |
|  | AAO61088 |
|  | AAA36810 |
|  | AAH46461 |
|  | AAA62865 |
|  | NP\_077742 |
|  | AAA61299 |
|  | CAA49373 |


---

|  |  |
| --- | --- |
| Organism | Mammal |


---

|  |  |
| --- | --- |
| Location | 2 58.0 cM (Mus musculus) |
|  | chromosome 11, 11p13 (Homo sapiens) |
|  | chromosome 2, 2 58.0 cM, 2 E (Mus musculus) |
|  | chromosome 3, 3q32 (Rattus norvegicus) |


---

|  |  |
| --- | --- |
