# Supplementary material for: Dynamic transcriptomic profiles of zebrafish gills in response to zinc depletion
Source: BMC Genomics. 2010 Oct 8;11:548. doi: 10.1186/1471-2164-11-548 (PMC3091697; doi:10.1186/1471-2164-11-548)
Supplement: Additional file 2 — Figure S1 - Interactive Direct Interaction Network of responses to zinc depletion. Mini web-site containing index.html and hyperlinked pages in subdirectory. The web site is an interactive version of Figure 6A containing curated interactions between regulated genes and respective proteins. Legend: Molecular interactions between zinc and proteins encoded by genes changed under zinc depletion. A Direct Interaction Network was created based on curated interactions contained within the PathwayArchitect database and provided through hyperlinks. Red ovals represent proteins and the blue circle symbolizes Zn(II). Dark blue squares denote 'binding', and light blue squares 'expression'; green squares stand for 'regulation', green diamonds for 'metabolism', and green circles for 'promoter binding'. Arrow heads indicate directionality of the interaction where annotated. [file 1471-2164-11-548-S2.ZIP › PathwayArchitect Zn def DIN2/138249.html]

# PROTEIN: WNT4

|  |  |
| --- | --- |
| Name | WNT4 |
| Type | PROTEIN |
| Description | wingless-type MMTV integration site family, member 4 |
| Note | The WNT gene family consists of structurally related genes which encode secreted signaling proteins. These proteins have been implicated in oncogenesis and in several developmental processes, including regulation of cell fate and patterning during embryogenesis. This gene is a member of the WNT gene family, and is the first signaling molecule shown to influence the sex-determination cascade. It encodes a protein which shows 98% amino acid identity to the Wnt4 protein of mouse and rat. This gene and DAX1, a gene known to antagonize the testis-determining factor, play a concerted role in both the control of female development and the prevention of testes formation. This gene and another two family members, WNT2 and WNT7B, may be associated with abnormal proliferation in breast tissue. |
| Alias | wingless-type MMTV integration site family, member 4 |
|  | signal molecule |
|  | wingless-type MMTV integration site family member 4 |
|  | Wnt-4 protein precursor |
|  | MGC123964 |
|  | WNT4 |
|  | OTTHUMP00000044725 |
|  | WNT-4 |
|  | Wnt-4 |
|  | WNT-4 protein precursor |
|  | MGC112773 |
|  | Wnt4 |


---

|  |  |
| --- | --- |
| GO Component | extracellular space |
|  | extracellular region |


---

|  |  |
| --- | --- |
| GO ID | GO:0048599 |
|  | GO:0005615 |
|  | GO:0042445 |
|  | GO:0005102 |
|  | GO:0007292 |
|  | GO:0005515 |
|  | GO:0007223 |
|  | GO:0005576 |
|  | GO:0004871 |
|  | GO:0007276 |
|  | GO:0005201 |
|  | GO:0007165 |
|  | GO:0007267 |
|  | GO:0008585 |
|  | GO:0009887 |
|  | GO:0007548 |
|  | GO:0007275 |
|  | GO:0016055 |


---

|  |  |
| --- | --- |
| MIM | MIM:603490 |
|  | MIM:277000 |


---

|  |  |
| --- | --- |
| Connectivity | 82 |


---

|  |  |
| --- | --- |
| Entrez ID | 22417 |
|  | 54361 |
|  | 84426 |


---

|  |  |
| --- | --- |
| Agilent ID | A\_23\_P382607 |
|  | A\_53\_P130025 |
|  | A\_53\_P172295 |
|  | A\_42\_P559414 |
|  | A\_23\_P11787 |
|  | A\_51\_P130475 |
|  | A\_14\_P126410 |


---

|  |  |
| --- | --- |
| Cellular Localization | Extracellular region |


---

|  |  |
| --- | --- |
| DbXref | KEGG pathway##04340##Hedgehog signaling pathway##http://www.genome.jp/dbget-bin/show\_pathway?rno04340+84426 |
|  | KEGG pathway##04340##Hedgehog signaling pathway##http://www.genome.jp/dbget-bin/show\_pathway?hsa04340+54361 |
|  | KEGG pathway##04310##Wnt signaling pathway##http://www.genome.jp/dbget-bin/show\_pathway?hsa04310+54361 |
|  | KEGG pathway##04310##Wnt signaling pathway##http://www.genome.jp/dbget-bin/show\_pathway?mmu04310+22417 |
|  | KEGG pathway##04310##Wnt signaling pathway##http://www.genome.jp/dbget-bin/show\_pathway?rno04310+84426 |
|  | KEGG pathway##04340##Hedgehog signaling pathway##http://www.genome.jp/dbget-bin/show\_pathway?mmu04340+22417 |


---

|  |  |
| --- | --- |
| Pathway | Wnt Signaling (Canonical) |
|  | Zn def RIN |
|  | Zn def DIN |


---

|  |  |
| --- | --- |
| GO Process | gametogenesis |
|  | female gamete generation |
|  | cell-cell signaling |
|  | hormone metabolism |
|  | female gonad development |
|  | signal transduction |
|  | organogenesis |
|  | frizzled-2 signaling pathway |
|  | oocyte development |
|  | development |
|  | Wnt receptor signaling pathway |
|  | organ morphogenesis |
|  | sex differentiation |


---

|  |  |
| --- | --- |
| UniGene | Mm.20355 |
|  | Rn.34782 |
|  | Hs.25766 |


---

|  |  |
| --- | --- |
| Affymetrix Probeset ID | 103238\_at |
|  | 1368641\_at |
|  | 1441687\_at |
|  | 1450782\_at |
|  | 1556689\_3p\_a\_at |
|  | 1556689\_a\_at |
|  | 208606\_s\_at |
|  | 2091\_at |
|  | 230751\_at |
|  | 43311\_at |
|  | 45438\_at |
|  | 56707\_at |
|  | 64236\_at |
|  | g13540518\_3p\_s\_at |
|  | 1374690\_at |
|  | Hs2.185029.1.S1\_3p\_at |
|  | Hs2.185029.1.S1\_3p\_s\_at |
|  | Hs.25766.0.A1\_3p\_at |
|  | Msa.1286.0\_at |
|  | Msa.1286.0\_g\_at |
|  | rc\_AA900630\_s\_at |
|  | rc\_AI103240\_at |
|  | rc\_AI172290\_i\_at |
|  | 128692\_r\_at |
|  | RC\_AA195420\_at |
|  | RC\_AA393766\_at |
|  | RC\_H23429\_at |
|  | TC19390\_at |
|  | TC22401\_at |


---

|  |  |
| --- | --- |
| GO Function | extracellular matrix structural constituent |
|  | protein binding |
|  | receptor binding |
|  | signal transducer activity |


---

|  |  |
| --- | --- |
| Nucleotide | NM\_053402 |
|  | AL445253 |
|  | BC057781 |
|  | BC103562 |
|  | AA984007 |
|  | AY009398 |
|  | NM\_030761 |
|  | M89797 |
|  | AY358947 |
|  | AF086347 |
|  | BC103561 |
|  | BC103560 |
|  | BC101962 |
|  | AF316543 |
|  | AL031281 |
|  | NM\_009523 |
|  | AF414100 |
|  | AF188608 |
|  | AK012727 |
|  | AY033057 |
|  | AB061675 |
|  | BT020125 |
|  | AF335591 |
|  | BC098752 |


---

|  |  |
| --- | --- |
| Protein | AAI03562 |
|  | CAI19849 |
|  | Q9QXQ5 |
|  | NP\_110388 |
|  | AAK25765 |
|  | AAQ89306 |
|  | NP\_033549 |
|  | AAL10394 |
|  | AAI03563 |
|  | CAI19850 |
|  | AAA40566 |
|  | AAH57781 |
|  | P56705 |
|  | AAK51699 |
|  | AAK50427 |
|  | AAH98752 |
|  | AAI01963 |
|  | CAI22251 |
|  | NP\_445854 |
|  | P22724 |
|  | AAV38928 |
|  | BAC23080 |
|  | AAG38658 |
|  | AAF15589 |
|  | AAI03561 |


---

|  |  |
| --- | --- |
| Organism | Mammal |


---

|  |  |
| --- | --- |
| Location | chromosome 4, 4 D3 (Mus musculus) |
|  | chromosome 1, 1p36.23-p35.1 (Homo sapiens) |
|  | chromosome 5, 5q36 (Rattus norvegicus) |


---

|  |  |
| --- | --- |
