# Supplementary material for: Dynamic transcriptomic profiles of zebrafish gills in response to zinc depletion
Source: BMC Genomics. 2010 Oct 8;11:548. doi: 10.1186/1471-2164-11-548 (PMC3091697; doi:10.1186/1471-2164-11-548)
Supplement: Additional file 2 — Figure S1 - Interactive Direct Interaction Network of responses to zinc depletion. Mini web-site containing index.html and hyperlinked pages in subdirectory. The web site is an interactive version of Figure 6A containing curated interactions between regulated genes and respective proteins. Legend: Molecular interactions between zinc and proteins encoded by genes changed under zinc depletion. A Direct Interaction Network was created based on curated interactions contained within the PathwayArchitect database and provided through hyperlinks. Red ovals represent proteins and the blue circle symbolizes Zn(II). Dark blue squares denote 'binding', and light blue squares 'expression'; green squares stand for 'regulation', green diamonds for 'metabolism', and green circles for 'promoter binding'. Arrow heads indicate directionality of the interaction where annotated. [file 1471-2164-11-548-S2.ZIP › PathwayArchitect Zn def DIN2/139077.html]

# PROTEIN: DSP

|  |  |
| --- | --- |
| Name | DSP |
| Type | PROTEIN |
| Description | desmoplakin |
| Note | Desmosomes are intercellular junctions that tightly link adjacent cells. Desmoplakin is an obligate component of functional desmosomes that anchors intermediate filaments to desmosomal plaques. The N-terminus of desmoplakin is required for localization to the desmosome and interacts with the N-terminal region of plakophilin 1 and plakoglobin. The C-terminus of desmoplakin binds with intermediate filaments. In the mid-region of desmoplakin, a coiled-coiled rod domain is responsible for homodimerization. Mutations in this gene are the cause of several cardiomyopathies and keratodermas as well as the autoimmune disease paraneoplastic pemphigus. |
| Alias | AA407887 |
|  | DP |
|  | 250/210 kDa paraneoplastic pemphigus antigen |
|  | AW109828 |
|  | desmoplakin I/II |
|  | AA407888 |
|  | 5730453H04Rik |
|  | DPI |
|  | desmoplakin II |
|  | desmosomal cytoskeletal connector molecule |
|  | desmoplakin I |
|  | DSP |
|  | 2300002E22Rik |
|  | DPII |
|  | desmoplakin (DPI, DPII) |


---

|  |  |
| --- | --- |
| GO Component | mitochondrion |
|  | intermediate filament |
|  | basolateral plasma membrane |
|  | cytoskeleton |
|  | cell-cell adherens junction |
|  | desmosome |


---

|  |  |
| --- | --- |
| GO ID | GO:0005856 |
|  | GO:0016323 |
|  | GO:0008544 |
|  | GO:0005913 |
|  | GO:0005739 |
|  | GO:0005882 |
|  | GO:0005200 |
|  | GO:0030057 |


---

|  |  |
| --- | --- |
| MIM | MIM:605676 |
|  | MIM:607450 |
|  | MIM:125647 |
|  | MIM:607655 |


---

|  |  |
| --- | --- |
| Connectivity | 156 |


---

|  |  |
| --- | --- |
| Entrez ID | 109620 |
|  | 1832 |


---

|  |  |
| --- | --- |
| Agilent ID | A\_51\_P201884 |
|  | A\_51\_P101460 |
|  | A\_53\_P113214 |
|  | A\_52\_P549815 |
|  | A\_14\_P122808 |
|  | A\_52\_P242544 |
|  | A\_53\_P111779 |
|  | A\_53\_P156644 |
|  | A\_32\_P157945 |
|  | A\_23\_P31031 |
|  | A\_53\_P115410 |


---

|  |  |
| --- | --- |
| Cellular Localization | Cytoskeleton |
|  | Mitochondrion |
|  | Plasma membrane |
|  | Cytoplasm |
|  | Organelle |
|  | Membrane |
|  | Cell |


---

|  |  |
| --- | --- |
| Pathway | Zn def RIN |
|  | Zn def DIN |


---

|  |  |
| --- | --- |
| GO Process | epidermis development |


---

|  |  |
| --- | --- |
| UniGene | Hs.519873 |
|  | Mm.355327 |


---

|  |  |
| --- | --- |
| Affymetrix Probeset ID | 1427610\_at |
|  | 1435493\_at |
|  | 1435494\_s\_at |
|  | 161892\_r\_at |
|  | 200606\_at |
|  | 36133\_at |
|  | 94247\_at |
|  | aa600542\_s\_at |
|  | aa690887\_at |
|  | g4758199\_3p\_at |
|  | g4758199\_3p\_s\_at |
|  | 240382\_at |
|  | 50625\_at |
|  | Hs.15550.0.A1\_3p\_at |
|  | AA247685\_at |
|  | RC\_H90899\_at |
|  | RC\_T89283\_at |
|  | RC\_W95070\_at |
|  | TC39498\_at |
|  | TC39498\_g\_at |


---

|  |  |
| --- | --- |
| GO Function | structural constituent of cytoskeleton |


---

|  |  |
| --- | --- |
| Nucleotide | AF148514 |
|  | NM\_001008844 |
|  | AB209992 |
|  | J05211 |
|  | AA600542 |
|  | BC033467 |
|  | AK009006 |
|  | NM\_004415 |
|  | AK077574 |
|  | AK130229 |
|  | XM\_621314 |
|  | AL031058 |
|  | M77830 |
|  | AF139065 |


---

|  |  |
| --- | --- |
| Protein | P15924 |
|  | XP\_621314 |
|  | AAF19785 |
|  | AAA85135 |
|  | BAE06074 |
|  | BAB26020 |
|  | AAA35766 |
|  | BAC36872 |
|  | AAF02527 |
|  | NP\_004406 |
|  | CAA19927 |
|  | NP\_001008844 |
|  | BAC85309 |


---

|  |  |
| --- | --- |
| Organism | Mammal |


---

|  |  |
| --- | --- |
| Location | chromosome 6, 6p24 (Homo sapiens) |
|  | chromosome 13, 13 A3.3 (Mus musculus) |


---

|  |  |
| --- | --- |
