# Supplementary material for: Dynamic transcriptomic profiles of zebrafish gills in response to zinc depletion
Source: BMC Genomics. 2010 Oct 8;11:548. doi: 10.1186/1471-2164-11-548 (PMC3091697; doi:10.1186/1471-2164-11-548)
Supplement: Additional file 2 — Figure S1 - Interactive Direct Interaction Network of responses to zinc depletion. Mini web-site containing index.html and hyperlinked pages in subdirectory. The web site is an interactive version of Figure 6A containing curated interactions between regulated genes and respective proteins. Legend: Molecular interactions between zinc and proteins encoded by genes changed under zinc depletion. A Direct Interaction Network was created based on curated interactions contained within the PathwayArchitect database and provided through hyperlinks. Red ovals represent proteins and the blue circle symbolizes Zn(II). Dark blue squares denote 'binding', and light blue squares 'expression'; green squares stand for 'regulation', green diamonds for 'metabolism', and green circles for 'promoter binding'. Arrow heads indicate directionality of the interaction where annotated. [file 1471-2164-11-548-S2.ZIP › PathwayArchitect Zn def DIN2/140624.html]

# PROTEIN: PCMT1

|  |  |
| --- | --- |
| Name | PCMT1 |
| Type | PROTEIN |
| Description | protein-L-isoaspartate (D-aspartate) O-methyltransferase |
| Note | Three classes of protein carboxyl methyltransferases, distinguished by their methyl-acceptor substrate specificity, have been found in prokaryotic and eukaryotic cells. The type II enzyme catalyzes the transfer of a methyl group from S-adenosyl-L-methionine to the free carboxyl groups of D-aspartyl and L-isoaspartyl residues. These methyl-accepting residues result from the spontaneous deamidation, isomerization, and racemization of normal L-aspartyl and L-asparaginyl residues and represent sites of covalent damage to aging proteins PCMT1 (EC 2.1.1.77) is a protein repair enzyme that initiates the conversion of abnormal D-aspartyl and L-isoaspartyl residues to the normal L-aspartyl form.[supplied by OMIM] |
| Alias | Pcmt1 |
|  | protein carboxyl methyltransferase |
|  | PCM |
|  | Protein L- isoaspartyl/D-aspartyl methyltransferase |
|  | L-isoaspartyl/D-aspartyl methyltransferase |
|  | MGC95039 |
|  | PIMT |
|  | L-isoaspartyl protein carboxyl methyltransferase |
|  | Protein-beta-aspartate methyltransferase |
|  | C79501 |


---

|  |  |
| --- | --- |
| GO Component | endoplasmic reticulum |


---

|  |  |
| --- | --- |
| GO ID | GO:0008757 |
|  | GO:0005783 |
|  | GO:0006464 |
|  | GO:0008168 |
|  | GO:0006479 |
|  | GO:0004719 |
|  | GO:0016740 |


---

|  |  |
| --- | --- |
| MIM | MIM:176851 |


---

|  |  |
| --- | --- |
| Connectivity | 54 |


---

|  |  |
| --- | --- |
| Entrez ID | 25604 |
|  | 18537 |
|  | 5110 |


---

|  |  |
| --- | --- |
| Agilent ID | A\_24\_P247608 |
|  | A\_53\_P177172 |
|  | A\_24\_P140827 |
|  | A\_14\_P128975 |
|  | A\_51\_P225852 |
|  | A\_51\_P225853 |
|  | A\_14\_P127971 |
|  | A\_44\_P484806 |
|  | A\_23\_P252866 |
|  | A\_53\_P118090 |
|  | A\_24\_P283320 |
|  | A\_52\_P620009 |
|  | A\_14\_P102172 |
|  | A\_43\_P11695 |


---

|  |  |
| --- | --- |
| Cellular Localization | Endoplasmic reticulum |
|  | Cytoplasm |
|  | Organelle |
|  | Cell |


---

|  |  |
| --- | --- |
| Pathway | Zn def RIN |
|  | Master Regulators |
|  | Zn def DIN |


---

|  |  |
| --- | --- |
| GO Process | protein modification |
|  | protein amino acid methylation |


---

|  |  |
| --- | --- |
| UniGene | Rn.86985 |
|  | Mm.258431 |
|  | Hs.279257 |


---

|  |  |
| --- | --- |
| Affymetrix Probeset ID | 1371966\_at |
|  | 1387258\_a\_at |
|  | 1422665\_a\_at |
|  | 1431085\_a\_at |
|  | 1431086\_s\_at |
|  | 1456604\_a\_at |
|  | 164446\_r\_at |
|  | 164600\_i\_at |
|  | 169033\_r\_at |
|  | 205202\_at |
|  | 208857\_s\_at |
|  | 210156\_s\_at |
|  | 37736\_at |
|  | 37737\_at |
|  | 37738\_g\_at |
|  | 94347\_i\_at |
|  | 94348\_f\_at |
|  | 95512\_at |
|  | aa543694\_i\_at |
|  | aa543694\_r\_at |
|  | aa543694\_s\_at |
|  | D25547\_at |
|  | d38023\_s\_at |
|  | g1332402\_3p\_a\_at |
|  | g180636\_3p\_a\_at |
|  | 113369\_r\_at |
|  | g4885538\_3p\_at |
|  | M26686\_g\_at |
|  | rc\_AA849797\_at |
|  | rc\_AI104517\_at |
|  | rc\_AI178024\_at |
|  | M26686\_at |
|  | 106427\_f\_at |
|  | TC32178\_at |
|  | TC36321\_at |
|  | TC36321\_g\_at |
|  | TC39416\_at |


---

|  |  |
| --- | --- |
| EC Number | EC 2.1.1.77 |


---

|  |  |
| --- | --- |
| GO Function | protein-L-isoaspartate (D-aspartate) O-methyltransferase activity |
|  | S-adenosylmethionine-dependent methyltransferase activity |
|  | methyltransferase activity |
|  | transferase activity |


---

|  |  |
| --- | --- |
| Nucleotide | M60320 |
|  | D25547 |
|  | D13892 |
|  | D38023 |
|  | S73903 |
|  | AK129778 |
|  | BC049613 |
|  | AK208253 |
|  | BC018569 |
|  | AK098739 |
|  | BC008748 |
|  | BC088417 |
|  | D11475 |
|  | AK179354 |
|  | AL355312 |
|  | S73902 |
|  | BC058966 |
|  | BC007501 |
|  | AK075632 |
|  | D25546 |
|  | M93009 |
|  | AK148789 |
|  | S73905 |
|  | U49740 |
|  | M84684 |
|  | AK214928 |
|  | AK003949 |
|  | M93008 |
|  | NM\_008786 |
|  | S72473 |
|  | BC040750 |
|  | NM\_013073 |
|  | D25545 |
|  | NM\_005389 |
|  | AK006162 |


---

|  |  |
| --- | --- |
| Protein | AAH07501 |
|  | P23506 |
|  | AAH88417 |
|  | BAA05030 |
|  | AAH08748 |
|  | BAA02034 |
|  | P22062 |
|  | AAB38386 |
|  | AAC60640 |
|  | NP\_005380 |
|  | BAB24438 |
|  | AAA74565 |
|  | AAH58966 |
|  | CAH72863 |
|  | NP\_032812 |
|  | AAA92742 |
|  | BAA02991 |
|  | CAH72861 |
|  | BAA05029 |
|  | AAC60641 |
|  | CAH72862 |
|  | AAC60639 |
|  | AAA90934 |
|  | P22061 |
|  | AAB31369 |
|  | BAA05028 |
|  | AAA90933 |
|  | NP\_037205 |
|  | AAH49613 |
|  | BAC35869 |


---

|  |  |
| --- | --- |
| Organism | Mammal |


---

|  |  |
| --- | --- |
| Location | 10 7.0 cM (Mus musculus) |
|  | chromosome 6, 6q24-q25 (Homo sapiens) |
|  | chromosome 1, 1p13 (Rattus norvegicus) |
|  | chromosome 10, 10 7.0 cM, 10 A1 (Mus musculus) |


---

|  |  |
| --- | --- |
