# Supplementary material for: Dynamic transcriptomic profiles of zebrafish gills in response to zinc depletion
Source: BMC Genomics. 2010 Oct 8;11:548. doi: 10.1186/1471-2164-11-548 (PMC3091697; doi:10.1186/1471-2164-11-548)
Supplement: Additional file 2 — Figure S1 - Interactive Direct Interaction Network of responses to zinc depletion. Mini web-site containing index.html and hyperlinked pages in subdirectory. The web site is an interactive version of Figure 6A containing curated interactions between regulated genes and respective proteins. Legend: Molecular interactions between zinc and proteins encoded by genes changed under zinc depletion. A Direct Interaction Network was created based on curated interactions contained within the PathwayArchitect database and provided through hyperlinks. Red ovals represent proteins and the blue circle symbolizes Zn(II). Dark blue squares denote 'binding', and light blue squares 'expression'; green squares stand for 'regulation', green diamonds for 'metabolism', and green circles for 'promoter binding'. Arrow heads indicate directionality of the interaction where annotated. [file 1471-2164-11-548-S2.ZIP › PathwayArchitect Zn def DIN2/145831.html]

# PROTEIN: F10

|  |  |
| --- | --- |
| Name | F10 |
| Type | PROTEIN |
| Description | coagulation factor X |
| Note | This gene encodes the vitamin K-dependent coagulation factor X precursor of the blood coagulation cascade. This factor precursor is converted to a mature two-chain form by the excision of the tripeptide RKR. Two chains of the factor are held together by 1 or more disulfide bonds; the light chain contains 2 EGF-like domains, while the heavy chain contains the catalytic domain which is structurally homologous to those of the other hemostatic serine proteases. The mature factor is activated by the cleavage of the activation peptide by factor IXa (in the intrisic pathway), or by factor VIIa (in the extrinsic pathway). The activated factor then converts prothrombin to thrombin in the presence of factor Va, Ca+2, and phospholipid during blood clotting. Mutations of this gene result in factor X deficiency, a hemorrhagic condition of variable severity. |
| Alias | FX |
|  | Cf10 |
|  | fX |
|  | factor Xa |
|  | coagulation factor 10 |
|  | prothrombinase |
|  | Stuart- Prower factor |
|  | F10 |
|  | MGC108722 |
|  | FXA |
|  | Stuart factor |


---

|  |  |
| --- | --- |
| GO Component | extracellular space |
|  | extracellular region |
|  | extrachromosomal DNA |


---

|  |  |
| --- | --- |
| GO ID | GO:0046821 |
|  | GO:0004252 |
|  | GO:0005615 |
|  | GO:0008233 |
|  | GO:0007596 |
|  | GO:0003804 |
|  | GO:0004263 |
|  | GO:0006508 |
|  | GO:0016787 |
|  | GO:0005509 |
|  | GO:0005576 |
|  | GO:0004295 |


---

|  |  |
| --- | --- |
| MIM | MIM:227600 |


---

|  |  |
| --- | --- |
| Connectivity | 757 |


---

|  |  |
| --- | --- |
| Entrez ID | 29243 |
|  | 2159 |
|  | 14058 |


---

|  |  |
| --- | --- |
| Agilent ID | A\_53\_P169822 |
|  | A\_23\_P205177 |
|  | A\_23\_P205172 |
|  | A\_42\_P504656 |
|  | A\_42\_P504653 |
|  | A\_53\_P105796 |
|  | A\_51\_P174961 |
|  | A\_14\_P101102 |


---

|  |  |
| --- | --- |
| Cellular Localization | Extracellular region |
|  | Cell |


---

|  |  |
| --- | --- |
| DbXref | Reactome##172723##Post-translational modification of proteins##http://www.reactome.org/cgi-bin/eventbrowser?DB=gk\_current&ID=172723 |
|  | KEGG pathway##04610##Complement and coagulation cascades##http://www.genome.jp/dbget-bin/show\_pathway?hsa04610+2159 |
|  | KEGG pathway##04610##Complement and coagulation cascades##http://www.genome.jp/dbget-bin/show\_pathway?rno04610+29243 |
|  | Reactome##109582##Hemostasis##http://www.reactome.org/cgi-bin/eventbrowser?DB=gk\_current&ID=109582 |
|  | KEGG pathway##04610##Complement and coagulation cascades##http://www.genome.jp/dbget-bin/show\_pathway?mmu04610+14058 |
|  | Reactome##163841##Post-translational modification of proteins##http://www.reactome.org/cgi-bin/eventbrowser?DB=gk\_current&ID=163841 |
|  | Reactome##172688##Hemostasis##http://www.reactome.org/cgi-bin/eventbrowser?DB=gk\_current&ID=172688 |


---

|  |  |
| --- | --- |
| Pathway | Zn def RIN |
|  | Master Regulators |
|  | Zn def DIN |


---

|  |  |
| --- | --- |
| GO Process | proteolysis and peptidolysis |
|  | blood coagulation |
|  | proteolysis |


---

|  |  |
| --- | --- |
| UniGene | Mm.262589 |
|  | Hs.361463 |
|  | Rn.21393 |


---

|  |  |
| --- | --- |
| Affymetrix Probeset ID | 103977\_at |
|  | 109826\_f\_at |
|  | 1369852\_at |
|  | 137633\_r\_at |
|  | 1418992\_at |
|  | 1418993\_s\_at |
|  | 1449305\_at |
|  | 164522\_r\_at |
|  | 205620\_at |
|  | 39979\_at |
|  | 67990\_at |
|  | 1389428\_at |
|  | AA734591\_at |
|  | D21215cds\_s\_at |
|  | g9961350\_3p\_at |
|  | g9961350\_3p\_x\_at |
|  | L29433\_at |
|  | Msa.15841.0\_s\_at |
|  | X79807\_at |
|  | TC15858\_at |
|  | TC35960\_at |
|  | rc\_AI104431\_at |


---

|  |  |
| --- | --- |
| EC Number | EC 3.4.21.6 |


---

|  |  |
| --- | --- |
| GO Function | hydrolase activity |
|  | coagulation factor Xa activity |
|  | peptidase activity |
|  | trypsin activity |
|  | chymotrypsin activity |
|  | serine-type endopeptidase activity |
|  | calcium ion binding |


---

|  |  |
| --- | --- |
| Nucleotide | AL137002 |
|  | AF087644 |
|  | BC040125 |
|  | M22613 |
|  | AK154100 |
|  | AB005892 |
|  | AF503510 |
|  | K01886 |
|  | L29433 |
|  | M33297 |
|  | AK149449 |
|  | AK154572 |
|  | AF211347 |
|  | M57285 |
|  | BC050219 |
|  | X79807 |
|  | AK171096 |
|  | K03194 |
|  | NM\_007972 |
|  | BC003877 |
|  | BC088151 |
|  | NM\_017143 |
|  | D21215 |
|  | BC046125 |
|  | AJ222677 |
|  | CT010325 |
|  | NM\_000504 |
|  | AK170282 |
|  | L00390 |


---

|  |  |
| --- | --- |
| Protein | AAA52486 |
|  | AAA52421 |
|  | AAM19347 |
|  | AAF22980 |
|  | AAH03877 |
|  | BAE32377 |
|  | CAJ18533 |
|  | BAA04756 |
|  | AAA52764 |
|  | NP\_031998 |
|  | CAA56202 |
|  | O88947 |
|  | AAA52490 |
|  | BAE42245 |
|  | CAI41386 |
|  | BAE41684 |
|  | Q63207 |
|  | AAA51984 |
|  | BAA21634 |
|  | AAC36345 |
|  | BAE28885 |
|  | AAH50219 |
|  | CAA10933 |
|  | AAA52636 |
|  | BAE32684 |
|  | NP\_058839 |
|  | NP\_000495 |
|  | P00742 |
|  | AAH46125 |
|  | AAH88151 |


---

|  |  |
| --- | --- |
| Organism | Mammal |


---

|  |  |
| --- | --- |
| Location | chromosome 16, 16q12.5 (Rattus norvegicus) |
|  | chromosome 13, 13q34 (Homo sapiens) |
|  | chromosome 8, 8 7.0 cM, 8 A1.1 (Mus musculus) |
|  | 8 7.0 cM (Mus musculus) |


---

|  |  |
| --- | --- |
