# Supplementary material for: Dynamic transcriptomic profiles of zebrafish gills in response to zinc depletion
Source: BMC Genomics. 2010 Oct 8;11:548. doi: 10.1186/1471-2164-11-548 (PMC3091697; doi:10.1186/1471-2164-11-548)
Supplement: Additional file 2 — Figure S1 - Interactive Direct Interaction Network of responses to zinc depletion. Mini web-site containing index.html and hyperlinked pages in subdirectory. The web site is an interactive version of Figure 6A containing curated interactions between regulated genes and respective proteins. Legend: Molecular interactions between zinc and proteins encoded by genes changed under zinc depletion. A Direct Interaction Network was created based on curated interactions contained within the PathwayArchitect database and provided through hyperlinks. Red ovals represent proteins and the blue circle symbolizes Zn(II). Dark blue squares denote 'binding', and light blue squares 'expression'; green squares stand for 'regulation', green diamonds for 'metabolism', and green circles for 'promoter binding'. Arrow heads indicate directionality of the interaction where annotated. [file 1471-2164-11-548-S2.ZIP › PathwayArchitect Zn def DIN2/148067.html]

# PROTEIN: ATP6V1E1

|  |  |
| --- | --- |
| Name | ATP6V1E1 |
| Type | PROTEIN |
| Description | ATPase, H+ transporting, lysosomal 31kDa, V1 subunit E isoform 1 |
| Note | This gene encodes a component of vacuolar ATPase (V-ATPase), a multisubunit enzyme that mediates acidification of eukaryotic intracellular organelles. V-ATPase dependent organelle acidification is necessary for such intracellular processes as protein sorting, zymogen activation, receptor-mediated endocytosis, and synaptic vesicle proton gradient generation. V-ATPase is composed of a cytosolic V1 domain and a transmembrane V0 domain. The V1 domain consists of three A and three B subunits, two G subunits plus the C, D, E, F, and H subunits. The V1 domain contains the ATP catalytic site. The V0 domain consists of five different subunits: a, c, c', c", and d. Additional isoforms of many of the V1 and V0 subunit proteins are encoded by multiple genes or alternatively spliced transcript variants. This encoded protein is one of two V1 domain E subunit proteins and is found ubiquitously. Pseudogenes for this gene have been found in the genome. |
| Alias | H(+)-transporting two-sector ATPase, 31kDa subunit |
|  | ATP6V1E1 |
|  | 2410029D23Rik |
|  | lysosomal 31kDa |
|  | V-ATPase E subunit |
|  | Vacuolar proton pump E subunit |
|  | ATP6E2 |
|  | Atp6v1e |
|  | Atp6e2 |
|  | H+ ATPase subunit E |
|  | MGC72933 |
|  | D6Ertd385e |
|  | V-ATPase, subunit E |
|  | ATP6E |
|  | Atp6e |
|  | ATPase, H+ transporting, lysosomal (vacuolar proton pump) 31kD |
|  | ATPase, H+ transporting lysosomal (vacuolar proton pump), 32 kDa |
|  | vacuolar H(+)-ATPase, E subunit |
|  | ATPase, H+ transporting, lysosomal 31kDa, V1 subunit E isoform 1 |
|  | P31 |
|  | H+-transporting ATP synthase chain E, vacuolar |
|  | H(+)-ATPase E-like protein |
|  | ATP6V1E |
|  | E2 |
|  | ATPase, H+ transporting, lysosomal 31kD, V1 subunit E |
|  | Atp6v1e1 |
|  | Vma4 |
|  | ATPase, H+ transporting, lysosomal 31kD, V1 subunit E isoform 1 |
|  | vacuolar proton pump, 31-kd subunit |
|  | V-ATPase 31 kDa subunit |


---

|  |  |
| --- | --- |
| GO Component | mitochondrion |
|  | plasma membrane |
|  | proton-transporting two-sector ATPase complex |
|  | cytoplasm |


---

|  |  |
| --- | --- |
| GO ID | GO:0005737 |
|  | GO:0006754 |
|  | GO:0016787 |
|  | GO:0015078 |
|  | GO:0005739 |
|  | GO:0005886 |
|  | GO:0015992 |
|  | GO:0046933 |
|  | GO:0046961 |
|  | GO:0006810 |
|  | GO:0016469 |
|  | GO:0006811 |
|  | GO:0015986 |
|  | GO:0015991 |
|  | GO:0046872 |
|  | GO:0008553 |


---

|  |  |
| --- | --- |
| MIM | MIM:108746 |


---

|  |  |
| --- | --- |
| Connectivity | 51 |


---

|  |  |
| --- | --- |
| Entrez ID | 297566 |
|  | 529 |
|  | 11973 |


---

|  |  |
| --- | --- |
| Agilent ID | A\_14\_P128184 |
|  | A\_42\_P658815 |
|  | A\_53\_P108140 |
|  | A\_23\_P143551 |
|  | A\_23\_P143545 |
|  | A\_53\_P102375 |
|  | A\_52\_P303388 |
|  | A\_51\_P166434 |


---

|  |  |
| --- | --- |
| Cellular Localization | Cytoplasm |
|  | Mitochondrion |
|  | Plasma membrane |
|  | Membrane |
|  | Cell |
|  | Organelle |


---

|  |  |
| --- | --- |
| DbXref | KEGG pathway##00193##ATP synthesis##http://www.genome.jp/dbget-bin/show\_pathway?rno00193+297566 |
|  | KEGG pathway##00193##ATP synthesis##http://www.genome.jp/dbget-bin/show\_pathway?hsa00193+529 |
|  | KEGG pathway##05110##Cholera - Infection##http://www.genome.jp/dbget-bin/show\_pathway?hsa05110+529 |
|  | KEGG pathway##00190##Oxidative phosphorylation##http://www.genome.jp/dbget-bin/show\_pathway?mmu00190+11973 |
|  | KEGG pathway##00193##ATP synthesis##http://www.genome.jp/dbget-bin/show\_pathway?mmu00193+11973 |
|  | KEGG pathway##00190##Oxidative phosphorylation##http://www.genome.jp/dbget-bin/show\_pathway?rno00190+297566 |
|  | KEGG pathway##00190##Oxidative phosphorylation##http://www.genome.jp/dbget-bin/show\_pathway?hsa00190+529 |


---

|  |  |
| --- | --- |
| Pathway | Zn def RIN |
|  | Master Regulators |
|  | Zn def DIN |


---

|  |  |
| --- | --- |
| GO Process | transport |
|  | proton transport |
|  | ATP hydrolysis coupled proton transport |
|  | ion transport |
|  | ATP biosynthesis |
|  | ATP synthesis coupled proton transport |


---

|  |  |
| --- | --- |
| UniGene | Hs.517338 |
|  | Mm.29045 |
|  | Rn.103171 |


---

|  |  |
| --- | --- |
| Affymetrix Probeset ID | 1371564\_at |
|  | 1420038\_at |
|  | 1449711\_at |
|  | 1449712\_s\_at |
|  | 1457049\_at |
|  | 208678\_at |
|  | 37367\_at |
|  | 94532\_at |
|  | g13325247\_3p\_at |
|  | Msa.11002.0\_at |
|  | Msa.11239.0\_f\_at |
|  | Msa.1869.0\_f\_at |
|  | Msa.23749.0\_f\_at |
|  | rc\_AI169159\_at |
|  | U13841\_f\_at |
|  | X76228\_at |
|  | 1377229\_at |
|  | Msa.24333.0\_f\_at |
|  | RC\_T15595\_at |
|  | TC26272\_at |


---

|  |  |
| --- | --- |
| EC Number | EC 3.6.3.14 |


---

|  |  |
| --- | --- |
| GO Function | hydrogen-transporting ATP synthase activity, rotational mechanism |
|  | hydrolase activity |
|  | hydrogen-transporting ATPase activity, rotational mechanism |
|  | hydrogen ion transporter activity |
|  | hydrogen-exporting ATPase activity, phosphorylative mechanism |
|  | metal ion binding |


---

|  |  |
| --- | --- |
| Nucleotide | AB074758 |
|  | AK189499 |
|  | NM\_001696 |
|  | AK149356 |
|  | BC059155 |
|  | BT007128 |
|  | CR456385 |
|  | BI546032 |
|  | AK186366 |
|  | NM\_007510 |
|  | AK169854 |
|  | X76228 |
|  | AK189578 |
|  | AK208012 |
|  | X71491 |
|  | NM\_198745 |
|  | AK146162 |
|  | AK167644 |
|  | AK196381 |
|  | U13841 |
|  | BC004443 |
|  | BC003421 |
|  | AK029146 |
|  | AK010603 |
|  | AK139791 |
|  | AI565049 |
|  | BC055438 |


---

|  |  |
| --- | --- |
| Protein | AAH03421 |
|  | NP\_001687 |
|  | AAC52412 |
|  | NP\_942040 |
|  | AAP35792 |
|  | P50518 |
|  | AAH04443 |
|  | BAB92084 |
|  | NP\_031536 |
|  | BAE41412 |
|  | AAH59155 |
|  | CAA50592 |
|  | CAA53814 |
|  | P36543 |
|  | CAG30271 |
|  | BAE28832 |
|  | AAH55438 |
|  | BAE26943 |
|  | BAE39695 |


---

|  |  |
| --- | --- |
| Organism | Mammal |


---

|  |  |
| --- | --- |
| Location | chromosome 22, 22pter-q11.2, 22pter-q11.2 (Homo sapiens) |
|  | chromosome 6, 6 54.0 cM, 6 F1 (Mus musculus) |
|  | 6 54.0 cM (Mus musculus) |
|  | 22q11.1 (Homo sapiens) |
|  | chromosome 4, 4q42 (Rattus norvegicus) |


---

|  |  |
| --- | --- |
