# Supplementary material for: Dynamic transcriptomic profiles of zebrafish gills in response to zinc depletion
Source: BMC Genomics. 2010 Oct 8;11:548. doi: 10.1186/1471-2164-11-548 (PMC3091697; doi:10.1186/1471-2164-11-548)
Supplement: Additional file 2 — Figure S1 - Interactive Direct Interaction Network of responses to zinc depletion. Mini web-site containing index.html and hyperlinked pages in subdirectory. The web site is an interactive version of Figure 6A containing curated interactions between regulated genes and respective proteins. Legend: Molecular interactions between zinc and proteins encoded by genes changed under zinc depletion. A Direct Interaction Network was created based on curated interactions contained within the PathwayArchitect database and provided through hyperlinks. Red ovals represent proteins and the blue circle symbolizes Zn(II). Dark blue squares denote 'binding', and light blue squares 'expression'; green squares stand for 'regulation', green diamonds for 'metabolism', and green circles for 'promoter binding'. Arrow heads indicate directionality of the interaction where annotated. [file 1471-2164-11-548-S2.ZIP › PathwayArchitect Zn def DIN2/149815.html]

# PROTEIN: ME2

|  |  |
| --- | --- |
| Name | ME2 |
| Type | PROTEIN |
| Description | malic enzyme 2, NAD(+)-dependent, mitochondrial |
| Note | This gene encodes a mitochondrial NAD-dependent malic enzyme, a homotetrameric protein, that catalyzes the oxidative decarboxylation of malate to pyruvate. It had previously been weakly linked to a syndrome known as Friedreich ataxia that has since been shown to be the result of mutation in a completely different gene. |
| Alias | malate dehydrogenase |
|  | Malic enzyme, mitochondrial |
|  | ME2 |
|  | pyruvic-malic carboxylase |
|  | D030040L20Rik |
|  | Malic enzyme 2 |
|  | Me2 |
|  | AW120568 |
|  | NAD-ME |
|  | malic enzyme 2, mitochondrial |


---

|  |  |
| --- | --- |
| GO Component | mitochondrion |


---

|  |  |
| --- | --- |
| GO ID | GO:0004471 |
|  | GO:0016616 |
|  | GO:0006090 |
|  | GO:0005489 |
|  | GO:0003824 |
|  | GO:0051287 |
|  | GO:0006108 |
|  | GO:0016619 |
|  | GO:0046872 |
|  | GO:0004470 |
|  | GO:0005739 |
|  | GO:0016491 |


---

|  |  |
| --- | --- |
| MIM | MIM:154270 |
|  | MIM:600669 |


---

|  |  |
| --- | --- |
| Connectivity | 95 |


---

|  |  |
| --- | --- |
| Entrez ID | 4200 |
|  | 107029 |


---

|  |  |
| --- | --- |
| Agilent ID | A\_53\_P103296 |
|  | A\_51\_P490817 |
|  | A\_53\_P153239 |
|  | A\_51\_P490823 |
|  | A\_14\_P110781 |
|  | A\_53\_P127744 |
|  | A\_24\_P348090 |
|  | A\_23\_P321261 |
|  | A\_23\_P38748 |
|  | A\_53\_P120916 |


---

|  |  |
| --- | --- |
| Cellular Localization | Mitochondrion |
|  | Cytoplasm |
|  | Organelle |
|  | Cell |


---

|  |  |
| --- | --- |
| DbXref | KEGG pathway##00620##Pyruvate metabolism##http://www.genome.jp/dbget-bin/show\_pathway?mmu00620+107029 |
|  | KEGG pathway##00620##Pyruvate metabolism##http://www.genome.jp/dbget-bin/show\_pathway?hsa00620+4200 |


---

|  |  |
| --- | --- |
| Pathway | Zn def RIN |
|  | Master Regulators |
|  | Zn def DIN |


---

|  |  |
| --- | --- |
| GO Process | malate metabolism |
|  | pyruvate metabolism |


---

|  |  |
| --- | --- |
| UniGene | Mm.36817 |
|  | Hs.233119 |


---

|  |  |
| --- | --- |
| Affymetrix Probeset ID | 112288\_at |
|  | 112289\_g\_at |
|  | 1426572\_at |
|  | 1458172\_at |
|  | 1562612\_at |
|  | 209397\_at |
|  | 210154\_at |
|  | 1439526\_at |
|  | Hs2.318977.1.S1\_3p\_at |
|  | M55905\_at |
|  | 210153\_s\_at |
|  | 1426573\_at |
|  | 36599\_at |
|  | TC20987\_at |
|  | g12652790\_3p\_at |
|  | g187299\_3p\_a\_at |


---

|  |  |
| --- | --- |
| EC Number | EC 1.1.1.38 |


---

|  |  |
| --- | --- |
| GO Function | malate dehydrogenase (oxaloacetate-decarboxylating) activity |
|  | oxidoreductase activity, acting on the CH-OH group of donors, NAD or NADP as acceptor |
|  | malate dehydrogenase (decarboxylating) activity |
|  | catalytic activity |
|  | oxidoreductase activity |
|  | NAD binding |
|  | malic enzyme activity |
|  | electron transporter activity |
|  | metal ion binding |


---

|  |  |
| --- | --- |
| Nucleotide | AK050980 |
|  | BC004709 |
|  | AK050933 |
|  | NM\_002396 |
|  | AK156403 |
|  | AK042289 |
|  | AB045122 |
|  | AK080403 |
|  | AJ294818 |
|  | AK162126 |
|  | BC000147 |
|  | AK043103 |
|  | AK218514 |
|  | AK171157 |
|  | AK034692 |
|  | AK039906 |
|  | AK077746 |
|  | NM\_145494 |
|  | M55905 |


---

|  |  |
| --- | --- |
| Protein | AAA36197 |
|  | P23368 |
|  | BAC31216 |
|  | AAH00147 |
|  | NP\_002387 |
|  | BAC34483 |
|  | BAE36741 |
|  | AAH04709 |
|  | CAC14574 |
|  | BAE33701 |
|  | BAB40980 |
|  | Q99KE1 |
|  | BAE42281 |
|  | NP\_663469 |
|  | BAC34467 |
|  | BAE43371 |


---

|  |  |
| --- | --- |
| Organism | Mammal |


---

|  |  |
| --- | --- |
| Location | chromosome 18, 6p25-p24, 6p25-p24 (Homo sapiens) |
|  | chromosome 18, 18 E2 (Mus musculus) |
|  | 18q21 (Homo sapiens) |


---

|  |  |
| --- | --- |
