# Supplementary material for: Dynamic transcriptomic profiles of zebrafish gills in response to zinc depletion
Source: BMC Genomics. 2010 Oct 8;11:548. doi: 10.1186/1471-2164-11-548 (PMC3091697; doi:10.1186/1471-2164-11-548)
Supplement: Additional file 2 — Figure S1 - Interactive Direct Interaction Network of responses to zinc depletion. Mini web-site containing index.html and hyperlinked pages in subdirectory. The web site is an interactive version of Figure 6A containing curated interactions between regulated genes and respective proteins. Legend: Molecular interactions between zinc and proteins encoded by genes changed under zinc depletion. A Direct Interaction Network was created based on curated interactions contained within the PathwayArchitect database and provided through hyperlinks. Red ovals represent proteins and the blue circle symbolizes Zn(II). Dark blue squares denote 'binding', and light blue squares 'expression'; green squares stand for 'regulation', green diamonds for 'metabolism', and green circles for 'promoter binding'. Arrow heads indicate directionality of the interaction where annotated. [file 1471-2164-11-548-S2.ZIP › PathwayArchitect Zn def DIN2/150162.html]

# PROTEIN: NOTCH1

|  |  |
| --- | --- |
| Name | NOTCH1 |
| Type | PROTEIN |
| Description | Notch homolog 1, translocation-associated (Drosophila) |
| Note | This gene encodes a member of the Notch family. Members of this Type 1 transmembrane protein family share structural characteristics including an extracellular domain consisting of multiple epidermal growth factor-like (EGF) repeats, and an intracellular domain consisting of multiple, different domain types. Notch family members play a role in a variety of developmental processes by controlling cell fate decisions. The Notch signaling network is an evolutionarily conserved intercellular signaling pathway which regulates interactions between physically adjacent cells. In Drosophilia, notch interaction with its cell-bound ligands (delta, serrate) establishes an intercellular signaling pathway that plays a key role in development. Homologues of the notch-ligands have also been identified in human, but precise interactions between these ligands and the human notch homologues remain to be determined. This protein is cleaved in the trans-Golgi network, and presented on the cell surface as a heterodimer. This protein functions as a receptor for membrane bound ligands, and may play multiple roles during development. |
| Alias | Notch1 |
|  | transmembrane receptor Notch1 B |
|  | transmembrane receptor Notch1 |
|  | neurogenic locus notch homolog protein 1 |
|  | Drosophila Notch homolog 1 |
|  | Tan1 |
|  | major type A protein |
|  | Notch 1 |
|  | Notch gene homolog 1 |
|  | transmembrane receptor Notch1 D |
|  | NOTCH |
|  | Notch (Drosophila) homolog 1 (translocation-associated) |
|  | hN1 |
|  | TAN1 |
|  | translocation-associated notch protein TAN-1 |
|  | Mis6 |
|  | Drosophila Notch homolog 1 (controlling the the ectodermal and neural cell fate in Drosophila) |
|  | 9930111A19Rik |
|  | lin-12 |


---

|  |  |
| --- | --- |
| GO Component | extracellular space |
|  | integral to membrane |
|  | membrane |


---

|  |  |
| --- | --- |
| GO ID | GO:0016020 |
|  | GO:0005615 |
|  | GO:0007386 |
|  | GO:0048103 |
|  | GO:0043065 |
|  | GO:0005515 |
|  | GO:0003682 |
|  | GO:0001837 |
|  | GO:0003700 |
|  | GO:0007492 |
|  | GO:0007368 |
|  | GO:0030154 |
|  | GO:0007507 |
|  | GO:0007409 |
|  | GO:0008544 |
|  | GO:0031069 |
|  | GO:0001708 |
|  | GO:0045944 |
|  | GO:0007275 |
|  | GO:0006350 |
|  | GO:0009790 |
|  | GO:0045596 |
|  | GO:0007219 |
|  | GO:0030324 |
|  | GO:0003677 |
|  | GO:0005524 |
|  | GO:0030216 |
|  | GO:0006355 |
|  | GO:0001763 |
|  | GO:0006357 |
|  | GO:0016021 |
|  | GO:0004872 |
|  | GO:0005509 |
|  | GO:0006955 |
|  | GO:0005198 |
|  | GO:0050793 |
|  | GO:0008284 |


---

|  |  |
| --- | --- |
| MIM | MIM:190198 |


---

|  |  |
| --- | --- |
| Connectivity | 577 |


---

|  |  |
| --- | --- |
| Entrez ID | 4851 |
|  | 18128 |
|  | 25496 |


---

|  |  |
| --- | --- |
| Agilent ID | A\_51\_P508510 |
|  | A\_44\_P233932 |
|  | A\_23\_P60387 |
|  | A\_23\_P60393 |
|  | A\_52\_P183088 |
|  | A\_42\_P586380 |
|  | A\_53\_P141501 |
|  | A\_14\_P113317 |
|  | A\_53\_P150452 |
|  | A\_14\_P134768 |


---

|  |  |
| --- | --- |
| Cellular Localization | Membrane |
|  | Cell |
|  | Extracellular region |


---

|  |  |
| --- | --- |
| DbXref | Reactome##157118##Notch Signaling Pathway##http://www.reactome.org/cgi-bin/eventbrowser?DB=gk\_current&ID=157118 |
|  | KEGG pathway##04330##Notch signaling pathway##http://www.genome.jp/dbget-bin/show\_pathway?mmu04330+18128 |
|  | KEGG pathway##04330##Notch signaling pathway##http://www.genome.jp/dbget-bin/show\_pathway?rno04330+25496 |
|  | KEGG pathway##04330##Notch signaling pathway##http://www.genome.jp/dbget-bin/show\_pathway?hsa04330+4851 |
|  | KEGG pathway##04320##Dorso-ventral axis formation##http://www.genome.jp/dbget-bin/show\_pathway?mmu04320+18128 |


---

|  |  |
| --- | --- |
| Pathway | Zn def RIN |
|  | Zn def DIN |


---

|  |  |
| --- | --- |
| GO Process | cell differentiation |
|  | cell fate specification |
|  | immune response |
|  | positive regulation of apoptosis |
|  | regulation of development |
|  | lung development |
|  | branching morphogenesis |
|  | epithelial to mesenchymal transition |
|  | axonogenesis |
|  | compartment specification |
|  | positive regulation of cell proliferation |
|  | keratinocyte differentiation |
|  | hair follicle morphogenesis |
|  | determination of left/right symmetry |
|  | positive regulation of transcription from RNA polymerase II promoter |
|  | negative regulation of cell differentiation |
|  | somatic stem cell division |
|  | regulation of transcription from RNA polymerase II promoter |
|  | regulation of transcription, DNA-dependent |
|  | transcription |
|  | embryonic development |
|  | Notch signaling pathway |
|  | development |
|  | heart development |
|  | endoderm development |
|  | epidermis development |


---

|  |  |
| --- | --- |
| UniGene | Rn.25046 |
|  | Mm.290610 |
|  | Hs.495473 |


---

|  |  |
| --- | --- |
| Affymetrix Probeset ID | 1371491\_at |
|  | 1390426\_at |
|  | 1418633\_at |
|  | 1418634\_at |
|  | 162204\_r\_at |
|  | 218902\_at |
|  | 223508\_at |
|  | 231660\_at |
|  | 65751\_at |
|  | 76569\_at |
|  | 81903\_at |
|  | 86254\_r\_at |
|  | 97497\_at |
|  | aa271199\_s\_at |
|  | g11275979\_3p\_at |
|  | g8923012\_3p\_at |
|  | Msa.18042.0\_s\_at |
|  | Msa.8394.0\_f\_at |
|  | rc\_AI136875\_at |
|  | W74847\_s\_at |
|  | X57405\_at |
|  | X57405\_g\_at |
|  | 100210\_at |
|  | Z11886\_s\_at |
|  | 1391567\_at |
|  | Hs.300181.0.A1\_3p\_at |
|  | RC\_AA278817\_at |
|  | TC20447\_at |


---

|  |  |
| --- | --- |
| GO Function | DNA binding |
|  | ATP binding |
|  | chromatin binding |
|  | protein binding |
|  | structural molecule activity |
|  | transcription factor activity |
|  | receptor activity |
|  | calcium ion binding |


---

|  |  |
| --- | --- |
| Nucleotide | CR457221 |
|  | AK157475 |
|  | AK042197 |
|  | AB101626 |
|  | M73980 |
|  | AB101627 |
|  | AB101628 |
|  | NM\_017617 |
|  | BC010325 |
|  | AK000012 |
|  | X82562 |
|  | AK090118 |
|  | AB101631 |
|  | XM\_342392 |
|  | AB101636 |
|  | AK054089 |
|  | BC013208 |
|  | X68278 |
|  | AB100603 |
|  | NM\_008714 |
|  | AK079454 |
|  | AK149054 |
|  | AB101629 |
|  | AL354671 |
|  | Z11886 |
|  | AJ238029 |
|  | AF508809 |
|  | Z21925 |
|  | L02613 |
|  | AK075572 |
|  | AL592301 |
|  | AB209873 |
|  | BC023886 |
|  | AB101630 |
|  | AF308602 |
|  | AB101625 |
|  | AK004408 |
|  | AB101624 |
|  | AB101637 |
|  | X57405 |
|  | AK154528 |


---

|  |  |
| --- | --- |
| Protein | AAA60614 |
|  | BAE34095 |
|  | BAA90883 |
|  | CAG33502 |
|  | CAA40667 |
|  | CAA48339 |
|  | AAK14898 |
|  | AAG33848 |
|  | BAC31194 |
|  | CAI16149 |
|  | Q01705 |
|  | XP\_342393 |
|  | P46531 |
|  | BAE32653 |
|  | BAD93110 |
|  | CAB40733 |
|  | NP\_060087 |
|  | CAA57909 |
|  | BAC77040 |
|  | CAA77941 |
|  | Q07008 |
|  | CAI13934 |
|  | BAC77038 |
|  | NP\_032740 |
|  | BAC77039 |
|  | AAM28905 |


---

|  |  |
| --- | --- |
| Organism | Mammal |


---

|  |  |
| --- | --- |
| Location | chromosome 9, 9q34.3 (Homo sapiens) |
|  | chromosome 2, 2 15.0 cM, 2 A3 (Mus musculus) |
|  | chromosome 3, 3p13 (Rattus norvegicus) |
|  | 2 15.0 cM (Mus musculus) |


---

|  |  |
| --- | --- |
