# Supplementary material for: Dynamic transcriptomic profiles of zebrafish gills in response to zinc depletion
Source: BMC Genomics. 2010 Oct 8;11:548. doi: 10.1186/1471-2164-11-548 (PMC3091697; doi:10.1186/1471-2164-11-548)
Supplement: Additional file 2 — Figure S1 - Interactive Direct Interaction Network of responses to zinc depletion. Mini web-site containing index.html and hyperlinked pages in subdirectory. The web site is an interactive version of Figure 6A containing curated interactions between regulated genes and respective proteins. Legend: Molecular interactions between zinc and proteins encoded by genes changed under zinc depletion. A Direct Interaction Network was created based on curated interactions contained within the PathwayArchitect database and provided through hyperlinks. Red ovals represent proteins and the blue circle symbolizes Zn(II). Dark blue squares denote 'binding', and light blue squares 'expression'; green squares stand for 'regulation', green diamonds for 'metabolism', and green circles for 'promoter binding'. Arrow heads indicate directionality of the interaction where annotated. [file 1471-2164-11-548-S2.ZIP › PathwayArchitect Zn def DIN2/151168.html]

# PROTEIN: HOXC8

|  |  |
| --- | --- |
| Name | HOXC8 |
| Type | PROTEIN |
| Description | homeo box C8 |
| Note | This gene belongs to the homeobox family of genes. The homeobox genes encode a highly conserved family of transcription factors that play an important role in morphogenesis in all multicellular organisms. Mammals possess four similar homeobox gene clusters, HOXA, HOXB, HOXC and HOXD, located on different chromosomes, consisting of 9 to 11 genes arranged in tandem. This gene is one of several homeobox HOXC genes located in a cluster on chromosome 12. The product of this gene may play a role in the regulation of cartilage differentiation. It could also be involved in chondrodysplasias or other cartilage disorders. |
| Alias | Hox3r4 |
|  | homeobox protein R4 gene |
|  | Hoxc-8 |
|  | Hoxc8 |
|  | M31 |
|  | HOX3 |
|  | D130011F21Rik |
|  | homeobox protein Hox-C8 |
|  | HOXC8 |
|  | HOX3A |
|  | Hox-3A |
|  | Hox-3.1 |
|  | R4 |
|  | Homeobox gene C8 |
|  | Hox-3.1, mouse, homolog of |
|  | Fragment |
|  | homeo box 3A |


---

|  |  |
| --- | --- |
| GO Component | transcription factor complex |
|  | nucleus |


---

|  |  |
| --- | --- |
| GO ID | GO:0030182 |
|  | GO:0005634 |
|  | GO:0003677 |
|  | GO:0006355 |
|  | GO:0007389 |
|  | GO:0009887 |
|  | GO:0006350 |
|  | GO:0007275 |
|  | GO:0005667 |
|  | GO:0003700 |
|  | GO:0045449 |


---

|  |  |
| --- | --- |
| MIM | MIM:142970 |


---

|  |  |
| --- | --- |
| Connectivity | 34 |


---

|  |  |
| --- | --- |
| Entrez ID | 368178 |
|  | 15426 |
|  | 3224 |


---

|  |  |
| --- | --- |
| Agilent ID | A\_23\_P398476 |
|  | A\_44\_P236271 |
|  | A\_51\_P301483 |
|  | A\_14\_P113771 |
|  | A\_24\_P124558 |
|  | A\_53\_P115651 |
|  | A\_14\_P130633 |
|  | A\_23\_P13743 |


---

|  |  |
| --- | --- |
| Cellular Localization | Nucleus |
|  | Organelle |
|  | Cell |


---

|  |  |
| --- | --- |
| Pathway | Zn def RIN |
|  | Master Regulators |
|  | Zn def DIN |


---

|  |  |
| --- | --- |
| GO Process | neuron differentiation |
|  | development |
|  | regulation of transcription, DNA-dependent |
|  | organ morphogenesis |
|  | transcription |
|  | pattern specification |
|  | regulation of transcription |


---

|  |  |
| --- | --- |
| UniGene | Hs.248050 |
|  | Mm.6167 |
|  | Rn.101733 |


---

|  |  |
| --- | --- |
| Affymetrix Probeset ID | 136504\_at |
|  | 1452412\_at |
|  | 221350\_at |
|  | 93378\_at |
|  | g12056968\_3p\_at |
|  | 1371281\_at |
|  | x07439\_at |
|  | 1380442\_at |
|  | 1392183\_at |
|  | rc\_AI235507\_at |
|  | M37568cds\_at |


---

|  |  |
| --- | --- |
| GO Function | transcription factor activity |
|  | DNA binding |


---

|  |  |
| --- | --- |
| Nucleotide | NM\_022658 |
|  | X07439 |
|  | AK083688 |
|  | BU618342 |
|  | M35603 |
|  | BC053898 |
|  | AY014300 |
|  | M37568 |
|  | X03659 |
|  | AF099474 |
|  | XM\_347335 |
|  | X99681 |
|  | AK080104 |
|  | AK051173 |
|  | X07646 |
|  | AK080107 |
|  | X99680 |
|  | NM\_010466 |
|  | AK079948 |
|  | AY014299 |


---

|  |  |
| --- | --- |
| Protein | P18866 |
|  | CAA67997 |
|  | P09025 |
|  | CAA30319 |
|  | BAC37827 |
|  | XP\_347336 |
|  | NP\_073149 |
|  | NP\_034596 |
|  | P31273 |
|  | CAA27294 |
|  | AAG42146 |
|  | BAC34544 |
|  | AAA37856 |
|  | AAA37857 |
|  | CAA30486 |
|  | AAA41344 |
|  | BAC37790 |
|  | CAA67996 |
|  | AAH53898 |


---

|  |  |
| --- | --- |
| Organism | Mammal |


---

|  |  |
| --- | --- |
| Location | 15 57.4 cM (Mus musculus) |
|  | chromosome 15, 15 57.4 cM, 15 F3 (Mus musculus) |
|  | chromosome 12, 12q13.3 (Homo sapiens) |
|  | chromosome 7, 7q35 (Rattus norvegicus) |


---

|  |  |
| --- | --- |
