# Supplementary material for: Dynamic transcriptomic profiles of zebrafish gills in response to zinc depletion
Source: BMC Genomics. 2010 Oct 8;11:548. doi: 10.1186/1471-2164-11-548 (PMC3091697; doi:10.1186/1471-2164-11-548)
Supplement: Additional file 2 — Figure S1 - Interactive Direct Interaction Network of responses to zinc depletion. Mini web-site containing index.html and hyperlinked pages in subdirectory. The web site is an interactive version of Figure 6A containing curated interactions between regulated genes and respective proteins. Legend: Molecular interactions between zinc and proteins encoded by genes changed under zinc depletion. A Direct Interaction Network was created based on curated interactions contained within the PathwayArchitect database and provided through hyperlinks. Red ovals represent proteins and the blue circle symbolizes Zn(II). Dark blue squares denote 'binding', and light blue squares 'expression'; green squares stand for 'regulation', green diamonds for 'metabolism', and green circles for 'promoter binding'. Arrow heads indicate directionality of the interaction where annotated. [file 1471-2164-11-548-S2.ZIP › PathwayArchitect Zn def DIN2/152141.html]

# PROTEIN: POLR2K

|  |  |
| --- | --- |
| Name | POLR2K |
| Type | PROTEIN |
| Description | polymerase (RNA) II (DNA directed) polypeptide K, 7.0kDa |
| Note | This gene encodes one of the smallest subunits of RNA polymerase II, the polymerase responsible for synthesizing messenger RNA in eukaryotes. This subunit is shared by the other two DNA-directed RNA polymerases. |
| Alias | RPB7.0 |
|  | MafY |
|  | metallothionein-I activator |
|  | polymerase (RNA) II (DNA directed) polypeptide K (7.0kD) |
|  | POLR2K |
|  | Metallothionein-I gene transcription activator |
|  | Mt1a |
|  | Polr2k |
|  | RPB12 |
|  | RPABC4 |
|  | DNA directed RNA polymerase II polypeptide K |
|  | DNA directed RNA polymerases I, II, and III 7.0 kda polypeptide |
|  | RPB10alpha |
|  | ABC10-alpha |
|  | hRPB7.0 |
|  | hsRPB10a |


---

|  |  |
| --- | --- |
| GO Component | DNA-directed RNA polymerase III complex |
|  | nucleus |
|  | DNA-directed RNA polymerase II, core complex |


---

|  |  |
| --- | --- |
| GO ID | GO:0005665 |
|  | GO:0005634 |
|  | GO:0003677 |
|  | GO:0016779 |
|  | GO:0005666 |
|  | GO:0006356 |
|  | GO:0006366 |
|  | GO:0003899 |
|  | GO:0046872 |
|  | GO:0006350 |
|  | GO:0008270 |
|  | GO:0016740 |
|  | GO:0006383 |


---

|  |  |
| --- | --- |
| MIM | MIM:606033 |


---

|  |  |
| --- | --- |
| Connectivity | 19 |


---

|  |  |
| --- | --- |
| Entrez ID | 17749 |
|  | 5440 |


---

|  |  |
| --- | --- |
| Agilent ID | A\_53\_P169659 |
|  | A\_53\_P155641 |
|  | A\_51\_P214449 |
|  | A\_51\_P214446 |
|  | A\_23\_P157449 |
|  | A\_23\_P157452 |
|  | A\_14\_P128401 |


---

|  |  |
| --- | --- |
| Cellular Localization | Nucleus |
|  | Organelle |
|  | Cell |


---

|  |  |
| --- | --- |
| DbXref | Reactome##73894##DNA Repair##http://www.reactome.org/cgi-bin/eventbrowser?DB=gk\_current&ID=73894 |
|  | Reactome##112155##RNA Polymerase III Simple Start Sequence Initiation At Type 2 Promoters##http://www.reactome.org/cgi-bin/eventbrowser?DB=gk\_current&ID=112155 |
|  | KEGG pathway##00240##Pyrimidine metabolism##http://www.genome.jp/dbget-bin/show\_pathway?hsa00240+5440 |
|  | Reactome##113409##Abortive termination of early transcription elongation by DSIF##http://www.reactome.org/cgi-bin/eventbrowser?DB=gk\_current&ID=113409 |
|  | Reactome##112386##Pausing and recovery of elongation##http://www.reactome.org/cgi-bin/eventbrowser?DB=gk\_current&ID=112386 |
|  | Reactome##112156##RNA Polymerase III Simple Start Sequence Initiation At Type 3 Promoters##http://www.reactome.org/cgi-bin/eventbrowser?DB=gk\_current&ID=112156 |
|  | Reactome##112054##RNA Polymerase III Abortive Initiation At Type 3 Open Promoters##http://www.reactome.org/cgi-bin/eventbrowser?DB=gk\_current&ID=112054 |
|  | KEGG pathway##03020##RNA polymerase##http://www.genome.jp/dbget-bin/show\_pathway?hsa03020+5440 |
|  | KEGG pathway##00230##Purine metabolism##http://www.genome.jp/dbget-bin/show\_pathway?mmu00230+17749 |
|  | Reactome##75862##Fall Back to Closed Pre-initiation Complex##http://www.reactome.org/cgi-bin/eventbrowser?DB=gk\_current&ID=75862 |
|  | Reactome##74159##Transcription##http://www.reactome.org/cgi-bin/eventbrowser?DB=gk\_current&ID=74159 |
|  | Reactome##112055##RNA Polymerase III Abortive Initiation At Type 1 Open Promoters##http://www.reactome.org/cgi-bin/eventbrowser?DB=gk\_current&ID=112055 |
|  | Reactome##75071##mRNA Processing##http://www.reactome.org/cgi-bin/eventbrowser?DB=gk\_current&ID=75071 |
|  | Reactome##113451##Resumption of RNA Polymerase III Productive Transcription##http://www.reactome.org/cgi-bin/eventbrowser?DB=gk\_current&ID=113451 |
|  | Reactome##113429##Elongating transcript encounters a lesion in the template##http://www.reactome.org/cgi-bin/eventbrowser?DB=gk\_current&ID=113429 |
|  | Reactome##74160##Gene Expression##http://www.reactome.org/cgi-bin/eventbrowser?DB=gk\_current&ID=74160 |
|  | Reactome##73946##Abortive initiation##http://www.reactome.org/cgi-bin/eventbrowser?DB=gk\_current&ID=73946 |
|  | Reactome##112149##RNA Polymerase III Abortive Initiation At Type 2 Open Promoters##http://www.reactome.org/cgi-bin/eventbrowser?DB=gk\_current&ID=112149 |
|  | Reactome##112153##RNA Polymerase III Simple Start Sequence Initiation At Type 1 Promoters##http://www.reactome.org/cgi-bin/eventbrowser?DB=gk\_current&ID=112153 |
|  | KEGG pathway##03020##RNA polymerase##http://www.genome.jp/dbget-bin/show\_pathway?mmu03020+17749 |
|  | Reactome##113442##RNA Polymerase III Retractive RNase Activity at U-tract Pause Sites##http://www.reactome.org/cgi-bin/eventbrowser?DB=gk\_current&ID=113442 |
|  | KEGG pathway##00230##Purine metabolism##http://www.genome.jp/dbget-bin/show\_pathway?hsa00230+5440 |
|  | Reactome##75891##Abortive Initiation After Second Transition##http://www.reactome.org/cgi-bin/eventbrowser?DB=gk\_current&ID=75891 |
|  | Reactome##75856##Abortive Initiation Before Second Transition##http://www.reactome.org/cgi-bin/eventbrowser?DB=gk\_current&ID=75856 |
|  | KEGG pathway##00240##Pyrimidine metabolism##http://www.genome.jp/dbget-bin/show\_pathway?mmu00240+17749 |


---

|  |  |
| --- | --- |
| Pathway | Zn def RIN |
|  | Master Regulators |
|  | Zn xs inventory |
|  | Zn def DIN |
|  | Zn xs DIN |
|  | Zn xs RIN |


---

|  |  |
| --- | --- |
| GO Process | regulation of transcription from RNA polymerase I promoter |
|  | transcription |
|  | transcription from RNA polymerase III promoter |
|  | transcription from RNA polymerase II promoter |


---

|  |  |
| --- | --- |
| UniGene | Mm.27375 |
|  | Hs.351475 |


---

|  |  |
| --- | --- |
| Affymetrix Probeset ID | 1452596\_at |
|  | 170286\_i\_at |
|  | 202635\_s\_at |
|  | 95003\_at |
|  | g4826923\_3p\_a\_at |
|  | Z47727\_at |
|  | 202634\_at |
|  | 141004\_f\_at |
|  | 39766\_r\_at |
|  | Hs.150675.0.S2\_3p\_x\_at |
|  | Msa.26370.0\_s\_at |
|  | D81608\_at |
|  | TC24368\_s\_at |


---

|  |  |
| --- | --- |
| EC Number | EC 2.7.7.6 |


---

|  |  |
| --- | --- |
| GO Function | DNA-directed RNA polymerase activity |
|  | transferase activity |
|  | DNA binding |
|  | zinc ion binding |
|  | nucleotidyltransferase activity |
|  | metal ion binding |


---

|  |  |
| --- | --- |
| Nucleotide | S63758 |
|  | BC028543 |
|  | Z47727 |
|  | AK003023 |
|  | BC018157 |
|  | AK008287 |
|  | AJ252078 |
|  | AK008136 |
|  | NM\_005034 |
|  | AA880275 |
|  | AK018914 |
|  | BC000806 |
|  | CR456870 |
|  | NM\_023127 |


---

|  |  |
| --- | --- |
| Protein | AAH18157 |
|  | Q63871 |
|  | CAG33151 |
|  | BAB25578 |
|  | AAH00806 |
|  | CAA87656 |
|  | P53803 |
|  | BAB25485 |
|  | NP\_005025 |
|  | AAH28543 |
|  | AAB27565 |
|  | BAB31480 |
|  | BAB22516 |
|  | NP\_075616 |
|  | CAB91873 |


---

|  |  |
| --- | --- |
| Organism | Mammal |


---

|  |  |
| --- | --- |
| Location | chromosome 8, 8q22.2 (Homo sapiens) |
|  | chromosome 15, 15 A2 (Mus musculus) |


---

|  |  |
| --- | --- |
