# Supplementary material for: Dynamic transcriptomic profiles of zebrafish gills in response to zinc depletion
Source: BMC Genomics. 2010 Oct 8;11:548. doi: 10.1186/1471-2164-11-548 (PMC3091697; doi:10.1186/1471-2164-11-548)
Supplement: Additional file 2 — Figure S1 - Interactive Direct Interaction Network of responses to zinc depletion. Mini web-site containing index.html and hyperlinked pages in subdirectory. The web site is an interactive version of Figure 6A containing curated interactions between regulated genes and respective proteins. Legend: Molecular interactions between zinc and proteins encoded by genes changed under zinc depletion. A Direct Interaction Network was created based on curated interactions contained within the PathwayArchitect database and provided through hyperlinks. Red ovals represent proteins and the blue circle symbolizes Zn(II). Dark blue squares denote 'binding', and light blue squares 'expression'; green squares stand for 'regulation', green diamonds for 'metabolism', and green circles for 'promoter binding'. Arrow heads indicate directionality of the interaction where annotated. [file 1471-2164-11-548-S2.ZIP › PathwayArchitect Zn def DIN2/154331.html]

# PROTEIN: EPAS1

|  |  |
| --- | --- |
| Name | EPAS1 |
| Type | PROTEIN |
| Description | endothelial PAS domain protein 1 |
| Note | Activates luciferase activity after transfection [RGD] |
| Alias | Hif2a |
|  | EPAS-1 |
|  | Epas1 |
|  | HIF-1alpha-like factor |
|  | Hypoxia-inducible factor 2 alpha |
|  | HLF (HIF1alpha-like factor) |
|  | HIF1alpha-like factor |
|  | HIF2A |
|  | HIF2 alpha |
|  | Hif like protein |
|  | HIF-2alpha |
|  | HIF1 alpha-like factor |
|  | HLF |
|  | MHLF |
|  | hypoxia inducible transcription factor 2alpha |
|  | HRF |
|  | HIF-1 alpha-like factor |
|  | HIF-2 alpha |
|  | MOP2 |
|  | HIF-related factor |
|  | PASD2 |


---

|  |  |
| --- | --- |
| GO Component | nucleus |


---

|  |  |
| --- | --- |
| GO ID | GO:0003677 |
|  | GO:0006355 |
|  | GO:0030528 |
|  | GO:0003705 |
|  | GO:0000160 |
|  | GO:0004871 |
|  | GO:0003700 |
|  | GO:0005634 |
|  | GO:0001525 |
|  | GO:0030154 |
|  | GO:0007165 |
|  | GO:0006366 |
|  | GO:0001666 |
|  | GO:0000155 |
|  | GO:0003713 |
|  | GO:0007275 |
|  | GO:0006350 |
|  | GO:0035035 |
|  | GO:0045449 |


---

|  |  |
| --- | --- |
| MIM | MIM:603349 |


---

|  |  |
| --- | --- |
| Connectivity | 127 |


---

|  |  |
| --- | --- |
| Entrez ID | 2034 |
|  | 29452 |
|  | 13819 |


---

|  |  |
| --- | --- |
| Agilent ID | A\_23\_P210210 |
|  | A\_23\_P430120 |
|  | A\_51\_P307954 |
|  | A\_44\_P177807 |
|  | A\_53\_P145831 |
|  | A\_53\_P126413 |
|  | A\_52\_P293443 |
|  | A\_14\_P116925 |
|  | A\_53\_P161525 |
|  | A\_43\_P12361 |
|  | A\_14\_P101874 |


---

|  |  |
| --- | --- |
| Cellular Localization | Nucleus |
|  | Organelle |
|  | Cell |


---

|  |  |
| --- | --- |
| Pathway | Zn def RIN |
|  | Master Regulators |
|  | Zn def DIN |


---

|  |  |
| --- | --- |
| GO Process | cell differentiation |
|  | angiogenesis |
|  | regulation of transcription, DNA-dependent |
|  | response to hypoxia |
|  | transcription |
|  | two-component signal transduction system (phosphorelay) |
|  | transcription from RNA polymerase II promoter |
|  | regulation of transcription |
|  | signal transduction |
|  | development |


---

|  |  |
| --- | --- |
| UniGene | Mm.1415 |
|  | Hs.468410 |
|  | Rn.55138 |


---

|  |  |
| --- | --- |
| Affymetrix Probeset ID | 241055\_at |
|  | 102698\_at |
|  | 133451\_at |
|  | 1369703\_at |
|  | 1398333\_at |
|  | 1449888\_at |
|  | 200878\_at |
|  | 200879\_s\_at |
|  | 1435436\_at |
|  | 229904\_at |
|  | 230711\_at |
|  | 235963\_at |
|  | 237843\_at |
|  | 242868\_at |
|  | 38092\_at |
|  | 38230\_at |
|  | 44028\_at |
|  | 44832\_at |
|  | 50963\_at |
|  | 69405\_at |
|  | 78015\_at |
|  | d89787\_at |
|  | d89787\_g\_at |
|  | g4503576\_3p\_a\_at |
|  | Hs.197663.0.A1\_3p\_at |
|  | Hs.23666.0.A1\_3p\_at |
|  | Hs.28774.0.A1\_3p\_at |
|  | Hs.37896.0.S1\_3p\_at |
|  | Hs.8136.0.S2\_3p\_at |
|  | rc\_AA964225\_at |
|  | rc\_AI233801\_at |
|  | U81984\_at |
|  | 114665\_at |
|  | 69813\_at |
|  | Hs.201537.0.A1\_3p\_at |
|  | RC\_AA058532\_at |
|  | RC\_R26252\_at |
|  | RC\_W84753\_at |
|  | TC31000\_at |
|  | rc\_AI058681\_at |


---

|  |  |
| --- | --- |
| GO Function | RNA polymerase II transcription factor activity, enhancer binding |
|  | transcription regulator activity |
|  | histone acetyltransferase binding |
|  | DNA binding |
|  | signal transducer activity |
|  | transcription coactivator activity |
|  | two-component sensor molecule activity |
|  | transcription factor activity |


---

|  |  |
| --- | --- |
| Nucleotide | AF045160 |
|  | AJ277828 |
|  | NM\_023090 |
|  | BC015869 |
|  | AK158430 |
|  | AK123845 |
|  | AC016912 |
|  | AK141185 |
|  | AK136911 |
|  | AF052094 |
|  | NM\_010137 |
|  | U51626 |
|  | AK087208 |
|  | U81984 |
|  | CD109908 |
|  | NM\_001430 |
|  | BC051338 |
|  | BC057870 |
|  | D89787 |
|  | U81983 |


---

|  |  |
| --- | --- |
| Protein | AAB41496 |
|  | BAA20130 |
|  | NP\_001421 |
|  | AAY14704 |
|  | BAE34506 |
|  | Q9JHS1 |
|  | AAH51338 |
|  | NP\_034267 |
|  | Q99814 |
|  | AAC51212 |
|  | AAC12871 |
|  | AAB41495 |
|  | AAH15869 |
|  | P97481 |
|  | AAH57870 |
|  | NP\_075578 |
|  | CAB96612 |


---

|  |  |
| --- | --- |
| Organism | Mammal |


---

|  |  |
| --- | --- |
| Location | chromosome 17, 17 E4 (Mus musculus) |
|  | chromosome 2, 2p21-p16 (Homo sapiens) |
|  | chromosome 6, 6q12 (Rattus norvegicus) |


---

|  |  |
| --- | --- |
