# Supplementary material for: Dynamic transcriptomic profiles of zebrafish gills in response to zinc depletion
Source: BMC Genomics. 2010 Oct 8;11:548. doi: 10.1186/1471-2164-11-548 (PMC3091697; doi:10.1186/1471-2164-11-548)
Supplement: Additional file 2 — Figure S1 - Interactive Direct Interaction Network of responses to zinc depletion. Mini web-site containing index.html and hyperlinked pages in subdirectory. The web site is an interactive version of Figure 6A containing curated interactions between regulated genes and respective proteins. Legend: Molecular interactions between zinc and proteins encoded by genes changed under zinc depletion. A Direct Interaction Network was created based on curated interactions contained within the PathwayArchitect database and provided through hyperlinks. Red ovals represent proteins and the blue circle symbolizes Zn(II). Dark blue squares denote 'binding', and light blue squares 'expression'; green squares stand for 'regulation', green diamonds for 'metabolism', and green circles for 'promoter binding'. Arrow heads indicate directionality of the interaction where annotated. [file 1471-2164-11-548-S2.ZIP › PathwayArchitect Zn def DIN2/155328.html]

# PROTEIN: CRSP3

|  |  |
| --- | --- |
| Name | CRSP3 |
| Type | PROTEIN |
| Description | cofactor required for Sp1 transcriptional activation, subunit 3, 130kDa |
| Note | The activation of gene transcription is a multistep process that is triggered by factors that recognize transcriptional enhancer sites in DNA. These factors work with co-activators to direct transcriptional initiation by the RNA polymerase II apparatus. The protein encoded by this gene is a subunit of the CRSP (cofactor required for SP1 activation) complex, which, along with TFIID, is required for efficient activation by SP1. This protein is also a component of other multisubunit complexes e.g. thyroid hormone receptor-(TR-) associated proteins which interact with TR and facilitate TR function on DNA templates in conjunction with initiation factors and cofactors. This protein also acts as a metastasis suppressor. Two alternatively spliced transcript variants encoding different isoforms have been described for this gene. |
| Alias | vitamin D3 receptor interacting protein |
|  | ESTM7 |
|  | Med23 |
|  | Transcriptional coactivator CRSP130 |
|  | 130 kDa transcriptional co-activator |
|  | MED23 |
|  | 130kDa |
|  | EST X83317 |
|  | 133 kDa transcriptional co-activator |
|  | DKFZp434H0117 |
|  | CRSP3 |
|  | Vitamin D3 receptor-interacting protein complex 130 kDa component |
|  | CRSP133 |
|  | DRIP130 |
|  | Activator-recruited cofactor 130 kDa component |
|  | CRSP130 |
|  | ARC130 |
|  | cofactor required for Sp1 transcriptional activation, subunit 3 (130kD) |
|  | SUR2 |
|  | mediator |
|  | mKIAA1216 |
|  | CRSP 130-kD subunit |
|  | KIAA1216 |
|  | transcriptional co-activator CRSP130 |
|  | 3000002A17Rik |
|  | X83317 |


---

|  |  |
| --- | --- |
| GO Component | transcription factor complex |


---

|  |  |
| --- | --- |
| GO ID | GO:0006355 |
|  | GO:0004872 |
|  | GO:0030528 |
|  | GO:0006357 |
|  | GO:0003713 |
|  | GO:0005667 |
|  | GO:0006350 |
|  | GO:0006367 |


---

|  |  |
| --- | --- |
| MIM | MIM:605042 |


---

|  |  |
| --- | --- |
| Connectivity | 26 |


---

|  |  |
| --- | --- |
| Entrez ID | 70208 |
|  | 9439 |


---

|  |  |
| --- | --- |
| Agilent ID | A\_14\_P130107 |
|  | A\_14\_P120780 |
|  | A\_53\_P144670 |
|  | A\_52\_P181506 |
|  | A\_23\_P330999 |
|  | A\_23\_P145501 |
|  | A\_51\_P190124 |
|  | A\_14\_P132192 |
|  | A\_52\_P596054 |
|  | A\_14\_P112086 |
|  | A\_53\_P144973 |


---

|  |  |
| --- | --- |
| Cellular Localization | Nucleus |
|  | Organelle |
|  | Cell |


---

|  |  |
| --- | --- |
| Pathway | Zn def RIN |
|  | Master Regulators |
|  | Zn def DIN |


---

|  |  |
| --- | --- |
| GO Process | transcription initiation from RNA polymerase II promoter |
|  | regulation of transcription, DNA-dependent |
|  | regulation of transcription from RNA polymerase II promoter |
|  | transcription |


---

|  |  |
| --- | --- |
| UniGene | Mm.28020 |
|  | Hs.29679 |


---

|  |  |
| --- | --- |
| Affymetrix Probeset ID | 223947\_s\_at |
|  | 1452064\_at |
|  | 218846\_at |
|  | 223946\_at |
|  | 242706\_s\_at |
|  | 242707\_at |
|  | 44525\_at |
|  | 44894\_at |
|  | 46975\_at |
|  | 79513\_at |
|  | 84917\_at |
|  | 95616\_at |
|  | aa386569\_s\_at |
|  | g12053064\_3p\_at |
|  | g7019352\_3p\_at |
|  | Hs.271535.0.S1\_3p\_at |
|  | Hs.271535.0.S1\_3p\_s\_at |
|  | 79516\_g\_at |
|  | RC\_AA452411\_at |
|  | RC\_AA504481\_at |
|  | RC\_N54839\_at |
|  | TC31239\_at |


---

|  |  |
| --- | --- |
| GO Function | transcription coactivator activity |
|  | transcription regulator activity |
|  | receptor activity |


---

|  |  |
| --- | --- |
| Nucleotide | AJ420587 |
|  | NM\_027347 |
|  | AF105332 |
|  | AF507918 |
|  | AK129312 |
|  | AL121575 |
|  | AF104255 |
|  | BC007088 |
|  | BC005508 |
|  | NM\_004830 |
|  | BC040398 |
|  | AL136776 |
|  | AK042346 |
|  | BC050916 |
|  | AA674714 |
|  | AK028545 |
|  | AB033042 |
|  | BC060759 |
|  | AK013854 |
|  | NM\_015979 |
|  | AF135022 |


---

|  |  |
| --- | --- |
| Protein | AAD31729 |
|  | NP\_004821 |
|  | BAB29019 |
|  | NP\_057063 |
|  | Q9ULK4 |
|  | BAC98122 |
|  | AAH50916 |
|  | CAI23321 |
|  | CAB66710 |
|  | BAA86530 |
|  | AAH05508 |
|  | CAB92072 |
|  | NP\_081623 |
|  | CAI23320 |
|  | CAI23319 |
|  | CAB92073 |
|  | AAD12724 |
|  | AAM28897 |
|  | AAH60759 |
|  | BAC26001 |
|  | AAD30202 |


---

|  |  |
| --- | --- |
| Organism | Mammal |


---

|  |  |
| --- | --- |
| Location | chromosome 10, 10 25.0 cM, 10 A4 (Mus musculus) |
|  | chromosome 6, 6q22.33-q24.1 (Homo sapiens) |
|  | 10 25.0 cM (Mus musculus) |


---

|  |  |
| --- | --- |
