# Supplementary material for: Dynamic transcriptomic profiles of zebrafish gills in response to zinc depletion
Source: BMC Genomics. 2010 Oct 8;11:548. doi: 10.1186/1471-2164-11-548 (PMC3091697; doi:10.1186/1471-2164-11-548)
Supplement: Additional file 2 — Figure S1 - Interactive Direct Interaction Network of responses to zinc depletion. Mini web-site containing index.html and hyperlinked pages in subdirectory. The web site is an interactive version of Figure 6A containing curated interactions between regulated genes and respective proteins. Legend: Molecular interactions between zinc and proteins encoded by genes changed under zinc depletion. A Direct Interaction Network was created based on curated interactions contained within the PathwayArchitect database and provided through hyperlinks. Red ovals represent proteins and the blue circle symbolizes Zn(II). Dark blue squares denote 'binding', and light blue squares 'expression'; green squares stand for 'regulation', green diamonds for 'metabolism', and green circles for 'promoter binding'. Arrow heads indicate directionality of the interaction where annotated. [file 1471-2164-11-548-S2.ZIP › PathwayArchitect Zn def DIN2/155972.html]

# PROTEIN: DDX18

|  |  |
| --- | --- |
| Name | DDX18 |
| Type | PROTEIN |
| Description | DEAD (Asp-Glu-Ala-Asp) box polypeptide 18 |
| Note | DEAD box proteins, characterized by the conserved motif Asp-Glu-Ala-Asp (DEAD), are putative RNA helicases. They are implicated in a number of cellular processes involving alteration of RNA secondary structure such as translation initiation, nuclear and mitochondrial splicing, and ribosome and spliceosome assembly. Based on their distribution patterns, some members of this family are believed to be involved in embryogenesis, spermatogenesis, and cellular growth and division. This gene encodes a DEAD box protein, and it is activated by Myc protein. |
| Alias | Myc-regulated DEAD-box protein |
|  | putative ATP-dependent RNA helicase |
|  | DEAD-box protein 18 |
|  | DDX18 |
|  | 2310005B10Rik |
|  | Ddx18 |
|  | Myc-regulated DEAD box protein |
|  | MGC117904 |
|  | DEAD/H (Asp-Glu-Ala-Asp/His) box polypeptide 18 (Myc-regulated) |
|  | FLJ33908 |
|  | MrDb |


---

|  |  |
| --- | --- |
| GO ID | GO:0003723 |
|  | GO:0004004 |
|  | GO:0016787 |
|  | GO:0005524 |
|  | GO:0000166 |
|  | GO:0004386 |
|  | GO:0003676 |
|  | GO:0008026 |


---

|  |  |
| --- | --- |
| MIM | MIM:606355 |


---

|  |  |
| --- | --- |
| Connectivity | 14 |


---

|  |  |
| --- | --- |
| Entrez ID | 66942 |
|  | 8886 |


---

|  |  |
| --- | --- |
| Agilent ID | A\_53\_P141644 |
|  | A\_24\_P165736 |
|  | A\_23\_P56865 |
|  | A\_53\_P117159 |
|  | A\_14\_P200969 |
|  | A\_14\_P122513 |
|  | A\_51\_P235291 |
|  | A\_14\_P116288 |
|  | A\_51\_P235284 |
|  | A\_53\_P125595 |
|  | A\_53\_P119747 |
|  | A\_14\_P122227 |
|  | A\_14\_P133479 |


---

|  |  |
| --- | --- |
| Pathway | Zn def RIN |
|  | Master Regulators |
|  | Zn def DIN |


---

|  |  |
| --- | --- |
| UniGene | Mm.44219 |
|  | Mm.321701 |
|  | Hs.363492 |


---

|  |  |
| --- | --- |
| Affymetrix Probeset ID | 100037\_at |
|  | 1416070\_a\_at |
|  | 1416071\_at |
|  | 1456000\_at |
|  | 160492\_at |
|  | 205763\_s\_at |
|  | 208896\_at |
|  | 208897\_s\_at |
|  | 398\_at |
|  | g13097182\_3p\_a\_at |
|  | g13787205\_3p\_a\_at |
|  | Hs.100555.1.S3\_3p\_at |
|  | Msa.32763.0\_s\_at |
|  | Msa.8738.0\_s\_at |
|  | X98743\_at |
|  | 208895\_s\_at |
|  | 100038\_at |
|  | Hs.100555.1.S1\_3p\_a\_at |
|  | RC\_AA287907\_s\_at |
|  | RC\_AA452233\_at |
|  | RC\_R08935\_f\_at |
|  | TC18610\_at |
|  | TC18610\_g\_at |
|  | TC40834\_at |


---

|  |  |
| --- | --- |
| EC Number | EC 3.6.1.- |


---

|  |  |
| --- | --- |
| GO Function | hydrolase activity |
|  | helicase activity |
|  | nucleotide binding |
|  | ATP binding |
|  | RNA binding |
|  | ATP-dependent RNA helicase activity |
|  | ATP-dependent helicase activity |
|  | nucleic acid binding |


---

|  |  |
| --- | --- |
| Nucleotide | AK012878 |
|  | X98743 |
|  | BC001238 |
|  | BC103776 |
|  | BC017160 |
|  | AK012706 |
|  | AK091227 |
|  | AK075864 |
|  | BC003360 |
|  | NM\_025860 |
|  | BC024739 |
|  | CR457060 |
|  | AC009312 |
|  | AC009404 |
|  | AB209392 |
|  | NM\_006773 |
|  | AI648005 |
|  | AK001467 |
|  | BC028246 |
|  | AK019845 |
|  | BU599536 |
|  | BX538334 |


---

|  |  |
| --- | --- |
| Protein | AAH24739 |
|  | NP\_006764 |
|  | AAY14819 |
|  | Q8K363 |
|  | CAA67295 |
|  | BAB31877 |
|  | AAX88947 |
|  | Q9NVP1 |
|  | BAB28424 |
|  | BAB28531 |
|  | CAG33341 |
|  | AAI03777 |
|  | BAC36015 |
|  | BAA91709 |
|  | AAH28246 |
|  | AAH01238 |
|  | AAH03360 |
|  | BAD92629 |
|  | NP\_080136 |
|  | BAC03616 |


---

|  |  |
| --- | --- |
| Organism | Mammal |


---

|  |  |
| --- | --- |
| Location | chromosome 2, 2q14.1 (Homo sapiens) |
|  | chromosome 1, 1 E2 (Mus musculus) |


---

|  |  |
| --- | --- |
