# Supplementary material for: Dynamic transcriptomic profiles of zebrafish gills in response to zinc depletion
Source: BMC Genomics. 2010 Oct 8;11:548. doi: 10.1186/1471-2164-11-548 (PMC3091697; doi:10.1186/1471-2164-11-548)
Supplement: Additional file 2 — Figure S1 - Interactive Direct Interaction Network of responses to zinc depletion. Mini web-site containing index.html and hyperlinked pages in subdirectory. The web site is an interactive version of Figure 6A containing curated interactions between regulated genes and respective proteins. Legend: Molecular interactions between zinc and proteins encoded by genes changed under zinc depletion. A Direct Interaction Network was created based on curated interactions contained within the PathwayArchitect database and provided through hyperlinks. Red ovals represent proteins and the blue circle symbolizes Zn(II). Dark blue squares denote 'binding', and light blue squares 'expression'; green squares stand for 'regulation', green diamonds for 'metabolism', and green circles for 'promoter binding'. Arrow heads indicate directionality of the interaction where annotated. [file 1471-2164-11-548-S2.ZIP › PathwayArchitect Zn def DIN2/1583454.html]

# BINDING:

|  |  |
| --- | --- |
| Type | BINDING |
| Effect | None |


---

|  |  |
| --- | --- |
| Score | 0 |


---

|  |  |
| --- | --- |
| Reference Count | 1 |


---

|  |  |
| --- | --- |
| Mechanism | Unknown |


---

|  |  |
| --- | --- |
| Reference:0 || PMID | 14988562 |
| SourceID | 124241 |
| Species | Human |
| Experimental Condition | in-vitro |
| Description | The interaction between HNF4-alpha and MRPS35 was demonstrated by chromatin immunoprecipitation (ChIP) and genomic microarray hybridization. Human ex vivo pancreatic islet cells and hepatocytes were fixed with formaldehyde. Protein cross-linked DNA was enriched by immunoprecipitation with anti-HNF4-alpha. Crosslinking was reversed and the enriched DNA was amplified and fluorescently labelled using ligation-mediated PCR (LM-PCR). Control DNA that had not been enriched by immunoprecipitation was subjected to LM-PCR with a different fluorophore. Immunoprecipitation-enriched and unenriched pools of labelled DNA were hybridized to a single DNA microarray (Hu13K array) containing 13,000 human proximal promoter regions. A whole-chip error model was used to calculate confidence values (P value) for each spot on the microarrays. The data from three independent experiments were combined to obtain a final average ratio and P value for each promoter region. Results were considered positive if the binding P value in the error model was < 0.001 or enrichment was as least 2-fold in the immunoprecipitation. Tables S8, S7. |
| Detection Method | cross-linking |
| Source | BIND |
  |


---

|  |  |
| --- | --- |
