# Supplementary material for: Dynamic transcriptomic profiles of zebrafish gills in response to zinc depletion
Source: BMC Genomics. 2010 Oct 8;11:548. doi: 10.1186/1471-2164-11-548 (PMC3091697; doi:10.1186/1471-2164-11-548)
Supplement: Additional file 2 — Figure S1 - Interactive Direct Interaction Network of responses to zinc depletion. Mini web-site containing index.html and hyperlinked pages in subdirectory. The web site is an interactive version of Figure 6A containing curated interactions between regulated genes and respective proteins. Legend: Molecular interactions between zinc and proteins encoded by genes changed under zinc depletion. A Direct Interaction Network was created based on curated interactions contained within the PathwayArchitect database and provided through hyperlinks. Red ovals represent proteins and the blue circle symbolizes Zn(II). Dark blue squares denote 'binding', and light blue squares 'expression'; green squares stand for 'regulation', green diamonds for 'metabolism', and green circles for 'promoter binding'. Arrow heads indicate directionality of the interaction where annotated. [file 1471-2164-11-548-S2.ZIP › PathwayArchitect Zn def DIN2/159517.html]

# PROTEIN: MAP7

|  |  |
| --- | --- |
| Name | MAP7 |
| Type | PROTEIN |
| Description | microtubule-associated protein 7 |
| Note | The product of this gene is a microtubule-associated protein that is predominantly expressed in cells of epithelial origin. Microtubule-associated proteins are thought to be involved in microtubule dynamics, which is essential for cell polarization and differentiation. This protein has been shown to be able to stabilize microtubules, and may serve to modulate microtubule functions. Studies of the related mouse protein also suggested an essential role in microtubule function required for spermatogenesis. |
| Alias | E-MAP-115 |
|  | dJ325F22.2 (microtubule-associated protein 7 (EMAP115, E-MAP-115)) |
|  | EMAP115 |


---

|  |  |
| --- | --- |
| MIM | MIM:604108 |


---

|  |  |
| --- | --- |
| Connectivity | 7 |


---

|  |  |
| --- | --- |
| Entrez ID | 9053 |


---

|  |  |
| --- | --- |
| Agilent ID | A\_14\_P107735 |
|  | A\_32\_P190416 |
|  | A\_14\_P129293 |
|  | A\_24\_P98021 |
|  | A\_14\_P139455 |
|  | A\_23\_P122736 |
|  | A\_14\_P112057 |


---

|  |  |
| --- | --- |
| Pathway | Master Regulators |
|  | Zn def RIN |
|  | Zn xs inventory |
|  | Zn def DIN |


---

|  |  |
| --- | --- |
| UniGene | Hs.486548 |


---

|  |  |
| --- | --- |
| Affymetrix Probeset ID | 202889\_x\_at |
|  | 202890\_at |
|  | 215471\_s\_at |
|  | 39732\_at |
|  | 90943\_at |
|  | Hs.146388.0.S3\_3p\_at |
|  | Hs.146388.1.S1\_3p\_a\_at |
|  | X73882\_at |
|  | RC\_AA058631\_at |
|  | RC\_T62571\_s\_at |
|  | RC\_W90688\_f\_at |


---

|  |  |
| --- | --- |
| Nucleotide | CB162980 |
|  | AL023284 |
|  | X73882 |
|  | AJ242502 |
|  | AJ242501 |
|  | BC025777 |
|  | AL024508 |
|  | CD557798 |
|  | NM\_003980 |


---

|  |  |
| --- | --- |
| Protein | AAH25777 |
|  | CAB88031 |
|  | CAI20475 |
|  | CAA52086 |
|  | NP\_003971 |
|  | CAD92557 |
|  | CAB88030 |


---

|  |  |
| --- | --- |
| Organism | Mammal |


---

|  |  |
| --- | --- |
| Location | chromosome 6, 6q23.3 (Homo sapiens) |


---

|  |  |
| --- | --- |
