# Supplementary material for: Dynamic transcriptomic profiles of zebrafish gills in response to zinc depletion
Source: BMC Genomics. 2010 Oct 8;11:548. doi: 10.1186/1471-2164-11-548 (PMC3091697; doi:10.1186/1471-2164-11-548)
Supplement: Additional file 2 — Figure S1 - Interactive Direct Interaction Network of responses to zinc depletion. Mini web-site containing index.html and hyperlinked pages in subdirectory. The web site is an interactive version of Figure 6A containing curated interactions between regulated genes and respective proteins. Legend: Molecular interactions between zinc and proteins encoded by genes changed under zinc depletion. A Direct Interaction Network was created based on curated interactions contained within the PathwayArchitect database and provided through hyperlinks. Red ovals represent proteins and the blue circle symbolizes Zn(II). Dark blue squares denote 'binding', and light blue squares 'expression'; green squares stand for 'regulation', green diamonds for 'metabolism', and green circles for 'promoter binding'. Arrow heads indicate directionality of the interaction where annotated. [file 1471-2164-11-548-S2.ZIP › PathwayArchitect Zn def DIN2/161166.html]

# PROTEIN: LGALS1

|  |  |
| --- | --- |
| Name | LGALS1 |
| Type | PROTEIN |
| Description | lectin, galactoside-binding, soluble, 1 (galectin 1) |
| Note | The galectins are a family of beta-galactoside-binding proteins implicated in modulating cell-cell and cell-matrix interactions. LGALS1 may act as an autocrine negative growth factor that regulates cell proliferation. |
| Alias | HPL |
|  | galectin |
|  | Galbp |
|  | L14 |
|  | AA410090 |
|  | beta-galactoside-binding lectin |
|  | Gbp |
|  | LGALS1 |
|  | beta-galactoside binding protein |
|  | Galaptin |
|  | S-Lac lectin 1 |
|  | Lect14 |
|  | Lactose-binding lectin 1 |
|  | galectin-1 |
|  | OTTHUMP00000028982 |
|  | Lgals1 |
|  | Beta-galactoside-binding lectin L-14-I |
|  | L-14.5 |
|  | RL 14.5 |
|  | 14 kDa lectin |
|  | Putative MAPK-activating protein MP12 |
|  | GBP |
|  | HBL |
|  | Lectin, galactose-binding, soluble, 1 |


---

|  |  |
| --- | --- |
| GO Component | extracellular space |


---

|  |  |
| --- | --- |
| GO ID | GO:0043123 |
|  | GO:0045445 |
|  | GO:0005615 |
|  | GO:0005529 |
|  | GO:0004871 |
|  | GO:0007157 |
|  | GO:0006915 |


---

|  |  |
| --- | --- |
| MIM | MIM:150570 |


---

|  |  |
| --- | --- |
| Connectivity | 406 |


---

|  |  |
| --- | --- |
| Entrez ID | 16852 |
|  | 56646 |
|  | 3956 |


---

|  |  |
| --- | --- |
| Agilent ID | A\_42\_P759159 |
|  | A\_23\_P166459 |
|  | A\_14\_P110396 |
|  | A\_44\_P445070 |
|  | A\_51\_P465281 |


---

|  |  |
| --- | --- |
| Cellular Localization | Extracellular region |


---

|  |  |
| --- | --- |
| Pathway | Zn def RIN |
|  | Master Regulators |
|  | Zn def DIN |


---

|  |  |
| --- | --- |
| GO Process | positive regulation of I-kappaB kinase/NF-kappaB cascade |
|  | heterophilic cell adhesion |
|  | myoblast differentiation |
|  | apoptosis |


---

|  |  |
| --- | --- |
| UniGene | Hs.445351 |
|  | Rn.57 |
|  | Mm.43831 |


---

|  |  |
| --- | --- |
| Affymetrix Probeset ID | 1367628\_at |
|  | 1419573\_a\_at |
|  | 1455439\_a\_at |
|  | 164180\_s\_at |
|  | 167131\_at |
|  | 201105\_at |
|  | 216405\_at |
|  | 216500\_at |
|  | 31574\_i\_at |
|  | 31575\_f\_at |
|  | 140033\_at |
|  | 33412\_at |
|  | 80667\_f\_at |
|  | 99669\_at |
|  | g6006015\_3p\_at |
|  | g6006015\_3p\_x\_at |
|  | Hs.287389.0.S1\_3p\_at |
|  | J04456\_at |
|  | Msa.683.0\_f\_at |
|  | rc\_AI172064\_at |
|  | x66532\_f\_at |
|  | RC\_H25999\_s\_at |
|  | TC14730\_at |
|  | TC32858\_f\_at |
|  | TC36394\_f\_at |


---

|  |  |
| --- | --- |
| GO Function | sugar binding |
|  | signal transducer activity |


---

|  |  |
| --- | --- |
| Nucleotide | M19036 |
|  | M33214 |
|  | X66532 |
|  | S44881 |
|  | X51903 |
|  | BC099479 |
|  | U40624 |
|  | BC020675 |
|  | X51579 |
|  | X15256 |
|  | M57678 |
|  | X15986 |
|  | BC002063 |
|  | CR456511 |
|  | X53067 |
|  | X51578 |
|  | J04456 |
|  | AK004298 |
|  | AB097036 |
|  | NM\_019904 |
|  | X51577 |
|  | M57470 |
|  | BC001693 |
|  | Z83844 |
|  | BC058476 |
|  | BT006775 |
|  | NM\_002305 |
|  | X14829 |
|  | NM\_008495 |


---

|  |  |
| --- | --- |
| Protein | AAH20675 |
|  | AAA40822 |
|  | CAA33328 |
|  | CAA34117 |
|  | CAB42897 |
|  | NP\_063969 |
|  | AAH58476 |
|  | P16045 |
|  | CAA47143 |
|  | AAB88582 |
|  | CAG30397 |
|  | AAA37313 |
|  | AAH99479 |
|  | CAA32938 |
|  | AAP35421 |
|  | CAA37242 |
|  | NP\_032521 |
|  | AAH02063 |
|  | P09382 |
|  | P11762 |
|  | CAA36183 |
|  | BAC77389 |
|  | AAA37667 |
|  | AAH01693 |
|  | AAB00777 |
|  | NP\_002296 |
|  | AAA36170 |
|  | CAA35930 |
|  | AAB19412 |


---

|  |  |
| --- | --- |
| Organism | Mammal |


---

|  |  |
| --- | --- |
| Location | chromosome 15, 15 44.9 cM, 15 E (Mus musculus) |
|  | chromosome 22, 22q13.1 (Homo sapiens) |
|  | chromosome 7, 7q34 (Rattus norvegicus) |
|  | 15 44.9 cM (Mus musculus) |


---

|  |  |
| --- | --- |
