# Supplementary material for: Dynamic transcriptomic profiles of zebrafish gills in response to zinc depletion
Source: BMC Genomics. 2010 Oct 8;11:548. doi: 10.1186/1471-2164-11-548 (PMC3091697; doi:10.1186/1471-2164-11-548)
Supplement: Additional file 2 — Figure S1 - Interactive Direct Interaction Network of responses to zinc depletion. Mini web-site containing index.html and hyperlinked pages in subdirectory. The web site is an interactive version of Figure 6A containing curated interactions between regulated genes and respective proteins. Legend: Molecular interactions between zinc and proteins encoded by genes changed under zinc depletion. A Direct Interaction Network was created based on curated interactions contained within the PathwayArchitect database and provided through hyperlinks. Red ovals represent proteins and the blue circle symbolizes Zn(II). Dark blue squares denote 'binding', and light blue squares 'expression'; green squares stand for 'regulation', green diamonds for 'metabolism', and green circles for 'promoter binding'. Arrow heads indicate directionality of the interaction where annotated. [file 1471-2164-11-548-S2.ZIP › PathwayArchitect Zn def DIN2/161626.html]

# PROTEIN: ATIC

|  |  |
| --- | --- |
| Name | ATIC |
| Type | PROTEIN |
| Description | 5-aminoimidazole-4-carboxamide ribonucleotide formyltransferase/IMP cyclohydrolase |
| Note | Catalyzes the final two steps of de novo purine biosynthesis [RGD] |
| Alias | AICARFT/IMPCHASE |
|  | AW212393 |
|  | PURH;ORFNames=OK/SW-cl.86 |
|  | PURH |
|  | Atic |
|  | Purh |
|  | ATIC |
|  | 2610509C24Rik |
|  | AICARFT |
|  | AICAR |
|  | AA536954 |
|  | IMPCHASE |


---

|  |  |
| --- | --- |
| GO ID | GO:0004643 |
|  | GO:0006139 |
|  | GO:0003824 |
|  | GO:0006189 |
|  | GO:0016787 |
|  | GO:0006164 |
|  | GO:0016740 |
|  | GO:0003937 |


---

|  |  |
| --- | --- |
| MIM | MIM:608688 |
|  | MIM:601731 |


---

|  |  |
| --- | --- |
| Connectivity | 97 |


---

|  |  |
| --- | --- |
| Entrez ID | 81643 |
|  | 108147 |
|  | 471 |


---

|  |  |
| --- | --- |
| Agilent ID | A\_51\_P222441 |
|  | A\_23\_P68087 |
|  | A\_44\_P374817 |
|  | A\_53\_P130473 |
|  | A\_14\_P101313 |
|  | A\_14\_P201485 |
|  | A\_53\_P152381 |
|  | A\_51\_P222436 |
|  | A\_42\_P671665 |
|  | A\_14\_P117158 |


---

|  |  |
| --- | --- |
| Pathway | Zn def RIN |
|  | Zn def DIN |


---

|  |  |
| --- | --- |
| GO Process | purine nucleotide biosynthesis |
|  | 'de novo' IMP biosynthesis |
|  | nucleobase, nucleoside, nucleotide and nucleic acid metabolism |


---

|  |  |
| --- | --- |
| DbXref | KEGG pathway##00230##Purine metabolism##http://www.genome.jp/dbget-bin/show\_pathway?hsa00230+471 |
|  | KEGG pathway##00230##Purine metabolism##http://www.genome.jp/dbget-bin/show\_pathway?mmu00230+108147 |
|  | KEGG pathway##00670##One carbon pool by folate##http://www.genome.jp/dbget-bin/show\_pathway?rno00670+81643 |
|  | KEGG pathway##00230##Purine metabolism##http://www.genome.jp/dbget-bin/show\_pathway?rno00230+81643 |
|  | KEGG pathway##00670##One carbon pool by folate##http://www.genome.jp/dbget-bin/show\_pathway?mmu00670+108147 |
|  | Reactome##15869##Nucleotide metabolism##http://www.reactome.org/cgi-bin/eventbrowser?DB=gk\_current&ID=15869 |
|  | KEGG pathway##00670##One carbon pool by folate##http://www.genome.jp/dbget-bin/show\_pathway?hsa00670+471 |


---

|  |  |
| --- | --- |
| UniGene | Rn.15114 |
|  | Mm.38010 |
|  | Hs.90280 |


---

|  |  |
| --- | --- |
| Affymetrix Probeset ID | 112333\_at |
|  | 116572\_at |
|  | 1369962\_at |
|  | 1428506\_at |
|  | 1452811\_at |
|  | 163798\_at |
|  | 208758\_at |
|  | 38811\_at |
|  | 52985\_at |
|  | 76913\_at |
|  | D82348\_at |
|  | D89514\_at |
|  | g2317691\_3p\_at |
|  | rc\_AI102917\_at |
|  | rc\_AI233925\_at |
|  | 108982\_at |
|  | TC20032\_at |
|  | TC30839\_at |
|  | TC35641\_at |


---

|  |  |
| --- | --- |
| GO Function | hydrolase activity |
|  | phosphoribosylaminoimidazolecarboxamide formyltransferase activity |
|  | transferase activity |
|  | IMP cyclohydrolase activity |
|  | catalytic activity |


---

|  |  |
| --- | --- |
| Nucleotide | D89514 |
|  | D89976 |
|  | AK157739 |
|  | NM\_031014 |
|  | D82348 |
|  | AK010449 |
|  | AK076091 |
|  | AK088701 |
|  | AK028930 |
|  | AK012074 |
|  | NM\_026195 |
|  | AK186275 |
|  | AK046353 |
|  | BC008879 |
|  | BC039925 |
|  | AK202783 |
|  | NM\_004044 |
|  | AC073284 |
|  | U37436 |
|  | BC072496 |
|  | AB062403 |
|  | AK010611 |
|  | AK139235 |


---

|  |  |
| --- | --- |
| Protein | AAH08879 |
|  | AAH39925 |
|  | NP\_004035 |
|  | BAA21762 |
|  | NP\_112276 |
|  | AAH72496 |
|  | P31939 |
|  | NP\_080471 |
|  | Q9CWJ9 |
|  | AAY24062 |
|  | AAA97405 |
|  | BAC32688 |
|  | BAB26949 |
|  | BAB93490 |
|  | BAB27060 |
|  | BAC36175 |
|  | BAB28011 |
|  | BAA11559 |
|  | BAA22837 |
|  | BAE23927 |


---

|  |  |
| --- | --- |
| Organism | Mammal |


---

|  |  |
| --- | --- |
| Location | chromosome 9, 9q33 (Rattus norvegicus) |
|  | chromosome 2, 2q35 (Homo sapiens) |
|  | chromosome 1, 1 C3 (Mus musculus) |


---

|  |  |
| --- | --- |
