# Supplementary material for: Dynamic transcriptomic profiles of zebrafish gills in response to zinc depletion
Source: BMC Genomics. 2010 Oct 8;11:548. doi: 10.1186/1471-2164-11-548 (PMC3091697; doi:10.1186/1471-2164-11-548)
Supplement: Additional file 2 — Figure S1 - Interactive Direct Interaction Network of responses to zinc depletion. Mini web-site containing index.html and hyperlinked pages in subdirectory. The web site is an interactive version of Figure 6A containing curated interactions between regulated genes and respective proteins. Legend: Molecular interactions between zinc and proteins encoded by genes changed under zinc depletion. A Direct Interaction Network was created based on curated interactions contained within the PathwayArchitect database and provided through hyperlinks. Red ovals represent proteins and the blue circle symbolizes Zn(II). Dark blue squares denote 'binding', and light blue squares 'expression'; green squares stand for 'regulation', green diamonds for 'metabolism', and green circles for 'promoter binding'. Arrow heads indicate directionality of the interaction where annotated. [file 1471-2164-11-548-S2.ZIP › PathwayArchitect Zn def DIN2/161995.html]

# PROTEIN: NPM1

|  |  |
| --- | --- |
| Name | NPM1 |
| Type | PROTEIN |
| Description | nucleophosmin (nucleolar phosphoprotein B23, numatrin) |
| Note | RNA-associated nucleolar phosphoprotein; involved in ribosome assembly [RGD] |
| Alias | mutation A in NPMc+ AML |
|  | mutation F in NPMc+ AML |
|  | nucleolar protein NO38 |
|  | Nucleolar phosphoprotein B23 |
|  | mutation D in NPMc+ AML |
|  | MGC102162 |
|  | numatrin |
|  | Npm1 |
|  | NO38 |
|  | Nucleolar protein NO38 |
|  | nucleophosmin 1 |
|  | mutation E in NPMc+ AML |
|  | mutation B in NPMc+ AML |
|  | MGC107291 |
|  | nucleophosmin/nucleoplasmin family, member 1 |
|  | Numatrin |
|  | deletion in NPMc- AML |
|  | MGC108517 |
|  | nucleolar protein B23.1 |
|  | B23NP |
|  | NPM |
|  | nucleolar phosphoprotein B23 |
|  | mutation C in NPMc+ AML |
|  | Nucleoplasmin-related protein (Nuclear protein B23 |
|  | B23 |
|  | NPM1 |


---

|  |  |
| --- | --- |
| GO Component | nucleolus |
|  | centrosome |
|  | nucleus |
|  | cytoplasm |


---

|  |  |
| --- | --- |
| GO ID | GO:0003723 |
|  | GO:0007098 |
|  | GO:0046982 |
|  | GO:0006950 |
|  | GO:0005737 |
|  | GO:0005515 |
|  | GO:0005730 |
|  | GO:0042255 |
|  | GO:0008285 |
|  | GO:0019843 |
|  | GO:0005634 |
|  | GO:0051092 |
|  | GO:0007569 |
|  | GO:0005813 |
|  | GO:0042803 |
|  | GO:0003676 |
|  | GO:0006886 |
|  | GO:0006913 |
|  | GO:0051059 |
|  | GO:0003713 |
|  | GO:0051082 |
|  | GO:0030957 |


---

|  |  |
| --- | --- |
| MIM | MIM:601626 |
|  | MIM:164040 |


---

|  |  |
| --- | --- |
| Connectivity | 487 |


---

|  |  |
| --- | --- |
| Entrez ID | 25498 |
|  | 4869 |
|  | 18148 |


---

|  |  |
| --- | --- |
| Agilent ID | A\_52\_P732583 |
|  | A\_14\_P132043 |
|  | A\_53\_P120749 |
|  | A\_53\_P128273 |
|  | A\_44\_P744519 |
|  | A\_32\_P188674 |
|  | A\_14\_P116462 |
|  | A\_14\_P126395 |
|  | A\_43\_P11644 |
|  | A\_24\_P188941 |
|  | A\_23\_P214037 |
|  | A\_32\_P49423 |
|  | A\_44\_P1058389 |
|  | A\_24\_P832113 |
|  | A\_24\_P826646 |
|  | A\_32\_P188677 |
|  | A\_51\_P467410 |
|  | A\_44\_P280759 |
|  | A\_51\_P467412 |
|  | A\_14\_P127485 |
|  | A\_32\_P789921 |


---

|  |  |
| --- | --- |
| Cellular Localization | Cytoplasm |
|  | Nucleolus |
|  | Nucleus |
|  | Centrosome |
|  | Cytoskeleton |
|  | Cell |
|  | Organelle |


---

|  |  |
| --- | --- |
| Pathway | Zn def RIN |
|  | Master Regulators |
|  | Zn def DIN |


---

|  |  |
| --- | --- |
| GO Process | negative regulation of cell proliferation |
|  | activation of NF-kappaB transcription factor |
|  | cell aging |
|  | response to stress |
|  | intracellular protein transport |
|  | centrosome cycle |
|  | ribosome assembly |
|  | nucleocytoplasmic transport |


---

|  |  |
| --- | --- |
| UniGene | Hs.519452 |
|  | Mm.350638 |
|  | Mm.6343 |
|  | Rn.54537 |


---

|  |  |
| --- | --- |
| Affymetrix Probeset ID | 101634\_at |
|  | 1398757\_at |
|  | 1399158\_a\_at |
|  | 1415839\_a\_at |
|  | 1432416\_a\_at |
|  | 165722\_r\_at |
|  | 200063\_s\_at |
|  | 221691\_3p\_x\_at |
|  | 221691\_x\_at |
|  | 221923\_s\_at |
|  | 38542\_at |
|  | 73448\_at |
|  | 78344\_at |
|  | 88066\_s\_at |
|  | 1380637\_at |
|  | g12803184\_3p\_a\_at |
|  | g13536990\_3p\_s\_at |
|  | Hs.9614.1.A1\_3p\_a\_at |
|  | J03969\_at |
|  | J04943\_at |
|  | M23613\_at |
|  | M33212\_s\_at |
|  | 165723\_at |
|  | TC33230\_at |
|  | TC33232\_at |
|  | 1398756\_at |
|  | rc\_AI029096\_at |


---

|  |  |
| --- | --- |
| GO Function | unfolded protein binding |
|  | protein heterodimerization activity |
|  | NF-kappaB binding |
|  | protein binding |
|  | transcription coactivator activity |
|  | Tat protein binding |
|  | RNA binding |
|  | rRNA binding |
|  | protein homodimerization activity |
|  | nucleic acid binding |


---

|  |  |
| --- | --- |
| Nucleotide | AK153901 |
|  | AY347529 |
|  | BC054755 |
|  | AK028253 |
|  | BC008495 |
|  | NM\_002520 |
|  | BC014349 |
|  | AK088923 |
|  | J02590 |
|  | M31004 |
|  | AY740637 |
|  | AY740638 |
|  | AK000472 |
|  | BC012566 |
|  | BC021668 |
|  | BC089546 |
|  | AY740640 |
|  | M37041 |
|  | AY740636 |
|  | BT007011 |
|  | J03969 |
|  | X16934 |
|  | M25062 |
|  | NM\_012992 |
|  | AK005420 |
|  | M23613 |
|  | AY740634 |
|  | D28343 |
|  | AL772409 |
|  | AB042278 |
|  | NM\_008722 |
|  | AK005498 |
|  | BC009623 |
|  | M26697 |
|  | BC003670 |
|  | M33212 |
|  | AK135517 |
|  | BC021983 |
|  | BC085278 |
|  | AK165860 |
|  | AY740635 |
|  | AK145813 |
|  | BC016716 |
|  | BC002398 |
|  | NM\_199185 |
|  | BU661768 |
|  | AK145124 |
|  | M28699 |
|  | BC092378 |
|  | BC060579 |
|  | AY740639 |
|  | BC050628 |
|  | J04944 |
|  | AK150164 |
|  | U89309 |
|  | BC088088 |
|  | J04943 |
|  | BC020467 |
|  | M37039 |
|  | AK028370 |
|  | BC016824 |
|  | BC016768 |
|  | BC090843 |


---

|  |  |
| --- | --- |
| Protein | BAE38420 |
|  | AAW67752 |
|  | AAA40795 |
|  | AAA40794 |
|  | AAA36380 |
|  | AAA40796 |
|  | NP\_037124 |
|  | AAA40793 |
|  | BAE26667 |
|  | NP\_002511 |
|  | AAH50628 |
|  | AAA36385 |
|  | AAH12566 |
|  | AAH90843 |
|  | BAC25910 |
|  | AAW67758 |
|  | AAH08495 |
|  | BAE32244 |
|  | P06748 |
|  | AAA39801 |
|  | AAH16824 |
|  | AAA36474 |
|  | CAI25149 |
|  | AAA58386 |
|  | AAH89546 |
|  | AAH88088 |
|  | AAH21668 |
|  | AAP35657 |
|  | BAA91188 |
|  | NP\_954654 |
|  | AAH54755 |
|  | AAH03670 |
|  | AAQ24860 |
|  | BAE29354 |
|  | BAB40600 |
|  | P13084 |
|  | AAH16768 |
|  | CAA34809 |
|  | NP\_032748 |
|  | AAH14349 |
|  | BAB24014 |
|  | AAW67754 |
|  | AAA36473 |
|  | AAB94739 |
|  | BAC40655 |
|  | BAC25844 |
|  | AAH60579 |
|  | CAI25150 |
|  | AAH21983 |
|  | AAH92378 |
|  | AAH16716 |
|  | BAE22562 |
|  | AAH85278 |
|  | AAW67755 |
|  | Q61937 |
|  | AAW67757 |
|  | AAH09623 |
|  | AAW67753 |
|  | AAA41731 |
|  | AAA41730 |
|  | CAI25151 |
|  | AAW67756 |
|  | BAA05709 |
|  | BAE26248 |
|  | AAH02398 |


---

|  |  |
| --- | --- |
| Organism | Mammal |


---

|  |  |
| --- | --- |
| Location | chromosome 5, 5q35 (Homo sapiens) |
|  | chromosome 10, 10q12 (Rattus norvegicus) |
|  | chromosome 11, 11 A4 (Mus musculus) |


---

|  |  |
| --- | --- |
