# Supplementary material for: Dynamic transcriptomic profiles of zebrafish gills in response to zinc depletion
Source: BMC Genomics. 2010 Oct 8;11:548. doi: 10.1186/1471-2164-11-548 (PMC3091697; doi:10.1186/1471-2164-11-548)
Supplement: Additional file 2 — Figure S1 - Interactive Direct Interaction Network of responses to zinc depletion. Mini web-site containing index.html and hyperlinked pages in subdirectory. The web site is an interactive version of Figure 6A containing curated interactions between regulated genes and respective proteins. Legend: Molecular interactions between zinc and proteins encoded by genes changed under zinc depletion. A Direct Interaction Network was created based on curated interactions contained within the PathwayArchitect database and provided through hyperlinks. Red ovals represent proteins and the blue circle symbolizes Zn(II). Dark blue squares denote 'binding', and light blue squares 'expression'; green squares stand for 'regulation', green diamonds for 'metabolism', and green circles for 'promoter binding'. Arrow heads indicate directionality of the interaction where annotated. [file 1471-2164-11-548-S2.ZIP › PathwayArchitect Zn def DIN2/2097236.html]

# BINDING:

|  |  |
| --- | --- |
| Type | BINDING |
| Effect | None |


---

|  |  |
| --- | --- |
| Score | 0 |


---

|  |  |
| --- | --- |
| Reference Count | 2 |


---

|  |  |
| --- | --- |
| Mechanism | Unknown |


---

|  |  |
| --- | --- |
| Reference:0 || PMID | 15893727 |
| Year | 2005 |
| SourceID | 295459 |
| Species | Rat |
| Experimental Condition | in-vitro |
| Journal | Mol Cell |
| Description | An interaction between B23 from an unspecified species and rat CAD was demonstrated by co-immunoprecipitation. Endogenous CAD was immunoprecipitated from extracts of apoptotic PC12 cells pretreated with staurosporine transfected with Myc-B23 using anti-CAD and precipitates were resolved by SDS-PAGE. Myc-B23 was detected by western blot using anti-Myc and anti-B23. Fig. 7C. The mutants Myc-B23[Lys194Asn, Lys200Asn](K194N, K200N), Myc-B23[Lys228Asn, Lys234Asn](K228N, K234N) and Myc-B23[Lys219Asn, Lys221Asn](K219N, K221N) did not co-immunoprecipitate with CAD. Fig. 7C. |
| Detection Method | coimmunoprecipitation |
| Source | BIND |
  |
|


---

|  |  |
| --- | --- |
 Reference:1 || PMID | 15893727 |
| Year | 2005 |
| SourceID | 295459 |
| Species | Rat |
| Experimental Condition | in-vitro |
| Journal | Mol Cell |
| Description | An interaction between B23 and CAD, both from unspecified species, was demonstrated by GST pull-down assay. Purified CAD was isolated using GST-B23 immobilized on glutathione beads after activating His-DFF45/40 by preincubation with active caspase-3 in the presence of PI(3,4,5)P3. Eluates were resolved by SDS-PAGE and analyzed by Western blot for anti-CAD. Fig. 7A, 7B. CAD was isolated by the truncation mutant GST-B23[1-13, 108-294](B23 delta14-107). Fig. 7A. CAD was not isolated by the truncation mutants GST-B23[1-83, 152-294] (B23 delta84-151), GST-B23[1-116, 187-294] (B23 delta117-186), GST-B23[1-185, 240-294] (B23 delta186-239), GST-B23[240-294] (B23 delta240-294), GST-B23[Lys194Asn, Lys200Asn](K194N, K200N), GST-B23[Lys228Asn, Lys234Asn](K228N, K234N) and GST-B23[Lys219Asn, Lys221Asn](K219N, K221N). Fig. 7A, 7B. |
| Detection Method | affinity chromatography technologies |
| Source | BIND |
  |


---

|  |  |
| --- | --- |
