# Supplementary material for: Dynamic transcriptomic profiles of zebrafish gills in response to zinc depletion
Source: BMC Genomics. 2010 Oct 8;11:548. doi: 10.1186/1471-2164-11-548 (PMC3091697; doi:10.1186/1471-2164-11-548)
Supplement: Additional file 2 — Figure S1 - Interactive Direct Interaction Network of responses to zinc depletion. Mini web-site containing index.html and hyperlinked pages in subdirectory. The web site is an interactive version of Figure 6A containing curated interactions between regulated genes and respective proteins. Legend: Molecular interactions between zinc and proteins encoded by genes changed under zinc depletion. A Direct Interaction Network was created based on curated interactions contained within the PathwayArchitect database and provided through hyperlinks. Red ovals represent proteins and the blue circle symbolizes Zn(II). Dark blue squares denote 'binding', and light blue squares 'expression'; green squares stand for 'regulation', green diamonds for 'metabolism', and green circles for 'promoter binding'. Arrow heads indicate directionality of the interaction where annotated. [file 1471-2164-11-548-S2.ZIP › PathwayArchitect Zn def DIN2/394983.html]

# BINDING:

|  |  |
| --- | --- |
| Type | BINDING |
| Effect | None |


---

|  |  |
| --- | --- |
| Score | 0 |


---

|  |  |
| --- | --- |
| Reference Count | 4 |


---

|  |  |
| --- | --- |
| Mechanism | Unknown |


---

|  |  |
| --- | --- |
| Reference:0 || Sentence | "The DD-RIE can be used for quantitative in vitro studies of the Hb-Hp interactions, for quantitation of Hp's Hb binding capacity and for investigations of haemolytic episodes in patients." |
| PMID | 6652908 |
| Year | 1983 |
| Species | Human |
| Journal | Clin Chim Acta |
| RefScore | 2 |
| Source | PArchNLP |
  |
|


---

|  |  |
| --- | --- |
 Reference:1 || Sentence | "Preincubation of the cells with Hb-Hp resulted in a decrease in binding of the radioactive Hb-Hp to the cell surface, and was accompanied with an accumulation of intracellular receptors." |
| PMID | 1354488 |
| Year | 1992 |
| Species | Human |
| Journal | Biochim Biophys Acta |
| RefScore | 2 |
| Source | PArchNLP |
  ||


---

|  |  |
| --- | --- |
 Reference:2 || Sentence | "Since one alphabeta-Hb half-molecule is known to bind to each Hp beta chain, the beta polypeptide chain content of each of the Hp 2-1 polymers could be estimated by by counting the number of Coomassie blue bands formed after electrophoresis of isolated Hp 2-1 polymers fractionally saturated with cyanmethemoglobin (Hb)." |
| PMID | 972143 |
| Year | 1976 |
| Species | Human |
| Journal | J Biol Chem |
| RefScore | 3 |
| Source | PArchNLP |
  ||


---

|  |  |
| --- | --- |
 Reference:3 || Sentence | "Since the formed Hb-Hp complex is taken up and metabolized by hepatocytes, it has been suggested that in soccer players a redistribution of iron stores occurs among tissue compartments." |
| PMID | 1752710 |
| Year | 1991 |
| Species | Human |
| Journal | Int J Sports Med |
| RefScore | 1 |
| Source | PArchNLP |
  |


---

|  |  |
| --- | --- |
