# Supplementary material for: Dynamic transcriptomic profiles of zebrafish gills in response to zinc depletion
Source: BMC Genomics. 2010 Oct 8;11:548. doi: 10.1186/1471-2164-11-548 (PMC3091697; doi:10.1186/1471-2164-11-548)
Supplement: Additional file 2 — Figure S1 - Interactive Direct Interaction Network of responses to zinc depletion. Mini web-site containing index.html and hyperlinked pages in subdirectory. The web site is an interactive version of Figure 6A containing curated interactions between regulated genes and respective proteins. Legend: Molecular interactions between zinc and proteins encoded by genes changed under zinc depletion. A Direct Interaction Network was created based on curated interactions contained within the PathwayArchitect database and provided through hyperlinks. Red ovals represent proteins and the blue circle symbolizes Zn(II). Dark blue squares denote 'binding', and light blue squares 'expression'; green squares stand for 'regulation', green diamonds for 'metabolism', and green circles for 'promoter binding'. Arrow heads indicate directionality of the interaction where annotated. [file 1471-2164-11-548-S2.ZIP › PathwayArchitect Zn def DIN2/400471.html]

# METABOLISM:

|  |  |
| --- | --- |
| Type | METABOLISM |
| Effect | None |


---

|  |  |
| --- | --- |
| Score | 0 |


---

|  |  |
| --- | --- |
| Reference Count | 58 |


---

|  |  |
| --- | --- |
| Mechanism | Unknown |


---

|  |  |
| --- | --- |
| Reference:0 || Sentence | "Since zinc is reported to be an efficient inducer of metallothionein synthesis, and probably of superoxide dismutase, we evaluated the effect of oral zinc supplementation on metallothionein and superoxide dismutase levels in patients with inflammatory bowel disease." |
| PMID | 7827298 |
| Year | 1994 |
| Species | Human |
| Journal | J Gastroenterol Hepatol |
| RefScore | 2 |
| Source | PArchNLP |
  |
|


---

|  |  |
| --- | --- |
 Reference:1 || Sentence | "One mechanism contributing to the teratogenicity of several developmental toxicants, is chemical-induced changes in maternal zinc (Zn) metabolism which result in an increased synthesis of maternal liver metallothionein (Mt), and a subsequent reduction in Zn delivery to the conceptus." |
| PMID | 11250055 |
| Year | 2001 |
| Species | Rat |
| Journal | Toxicology |
| RefScore | 1 |
| Source | PArchNLP |
  ||


---

|  |  |
| --- | --- |
 Reference:2 || Sentence | "Induction of MT synthesis by Zn contributes to the reduction in free radicals produced by Cu and Fe." |
| PMID | 12242608 |
| Year | 2002 |
| Species | Rat |
| Journal | Arch Toxicol |
| RefScore | 0 |
| Source | PArchNLP |
  ||


---

|  |  |
| --- | --- |
 Reference:3 || Sentence | "Furthermore, induction of metallothionein synthesis by zinc may contribute to the reduction in free radicals." |
| PMID | 2847178 |
| Year | 1988 |
| Species | Rat |
| Journal | Proc Soc Exp Biol Med |
| RefScore | 0 |
| Source | PArchNLP |
  ||


---

|  |  |
| --- | --- |
 Reference:4 || Sentence | "Intestinal metallothionein concentrations were lower and zinc absorption rates from isolated intestinal loops were higher in rats fed a low zinc diet compared with those fed a high zinc diet or given parenteral zinc to induce metallothionein synthesis." |
| PMID | 1729476 |
| Year | 1992 |
| Species | Rat |
| Journal | J Nutr |
| RefScore | 2 |
| Source | PArchNLP |
  ||


---

|  |  |
| --- | --- |
 Reference:5 || Sentence | "The induction of MT synthesis by zinc salts in newborn rats was not affected by the in utero reduction of GSH levels." |
| PMID | 2566683 |
| Year | 1989 |
| Species | Rat |
| Journal | J Pharmacol Exp Ther |
| RefScore | 1 |
| Source | PArchNLP |
  ||


---

|  |  |
| --- | --- |
 Reference:6 || Sentence | "In the chronic therapy, zinc compounds, inducing intestinal and hepatic metallothionein synthesis, have been gaining more common application." |
| PMID | 12607416 |
| Year | 2002 |
| Species | Human |
| Journal | Wiad Lek |
| RefScore | 2 |
| Source | PArchNLP |
  ||


---

|  |  |
| --- | --- |
 Reference:7 || Sentence | "We examined the role of reactive oxygen metabolites and the protective effect of zinc-induced metallothionein (MT) synthesis on gentamicin nephrotoxicity both in vivo and in vitro." |
| PMID | 7800247 |
| Year | 1994 |
| Species | Rat |
| Journal | Nephrol Dial Transplant |
| RefScore | 1 |
| Source | PArchNLP |
  ||


---

|  |  |
| --- | --- |
 Reference:8 || Sentence | "The effect of dopamine in stimulating the synthesis of metallothionein was similar to that of zinc, known to generate the synthesis of metallothionein, and to that of H2O2 and FeS04, known to generate free radicals." |
| PMID | 8281125 |
| Year | 1993 |
| Species | Rat |
|  | Human |
| Journal | Neurochem Int |
| RefScore | 2 |
| Source | PArchNLP |
  ||


---

|  |  |
| --- | --- |
 Reference:9 || Sentence | "It appears that the elevated levels of copper and zinc, rather than gold, are responsible for the induction of MT synthesis." |
| PMID | 3118513 |
| Year | 1987 |
| Species | Rat |
| Journal | Toxicology |
| RefScore | 1 |
| Source | PArchNLP |
  ||


---

|  |  |
| --- | --- |
 Reference:10 || Sentence | "These data indicate that zinc injection can elevate hepatic zinc levels and induce metallothionein synthesis in newborn rats despite high basal levels; cadmium injection does not induce metallothionein synthesis, though cadmium is avidly sequestered by pre-existing metallothionein." |
| PMID | 6883574 |
| Year | 1983 |
| Species | Rat |
| Journal | Chem Biol Interact |
| RefScore | 1 |
| Source | PArchNLP |
  ||


---

|  |  |
| --- | --- |
 Reference:11 || Sentence | "The results indicate that Zn, Cd, Hg, Co, Ni and dexamethasone induce MT in vitro and thus are direct inducers of MT synthesis in hepatic tissue." |
| PMID | 3669099 |
| Year | 1987 |
| Species | Rat |
| Journal | J Toxicol Environ Health |
| RefScore | 1 |
| Source | PArchNLP |
  ||


---

|  |  |
| --- | --- |
 Reference:12 || Sentence | "In order to study whether or not there is a limit of zinc accumulation in livers of zinc-injected rats and establish the biosynthesis relationship of metallothionein by zinc, I examined the accumulated amount of zinc in rat liver after injection of ZnSO4 and the relationship between zinc and metallothionein contents in rat livers." |
| PMID | 7620842 |
| Year | 1995 |
| Species | Rat |
| Journal | Res Commun Mol Pathol Pharmacol |
| RefScore | 2 |
| Source | PArchNLP |
  ||


---

|  |  |
| --- | --- |
 Reference:13 || Sentence | "CONCLUSION: These results suggest that neither the increased synthesis of a metal regulatory factor (MRF) nor an increase in half-life of MT mRNA is involved in the mechanism of increased MT biosynthesis upon addition of Zn." |
| PMID | 8195040 |
| Year | 1994 |
| Species | Human |
| Journal | Int J Radiat Oncol Biol Phys |
| RefScore | 3 |
| Source | PArchNLP |
  ||


---

|  |  |
| --- | --- |
 Reference:14 || Sentence | "Zinc salts induce the synthesis of metallothionein in cells." |
| PMID | 14759316 |
| Year | 2003 |
| Species | Human |
| Journal | Zhonghua Er Ke Za Zhi |
| RefScore | 1 |
| Source | PArchNLP |
  ||


---

|  |  |
| --- | --- |
 Reference:15 || Sentence | "These alterations include 1) decreased skeletal muscle accretion due to increased rates of protein degradation and decreased protein synthesis; 2) increased basal metabolic rate resulting in increased energy utilization; 3) use of dietary amino acids for gluconeogenesis and as an energy source instead of for muscle protein accretion; 4) synthesis by the liver of acute phase proteins; 5) redistribution of iron, zinc, and copper within the body due to the hepatic synthesis of metallothionein, ferritin, and ceruloplasmin; (6) impaired accretion of cartilage and bone; and 7) release of hormones such as insulin, glucagon, and corticosterone." |
| PMID | 1717968 |
| Year | 1991 |
| Species | Human |
| Journal | Poult Sci |
| RefScore | 2 |
| Source | PArchNLP |
  ||


---

|  |  |
| --- | --- |
 Reference:16 || Sentence | "After a 24-h incubation period, the synthesis of Zn-thionein increased markedly at levels of 150 microM Zn or greater, Cd induced metallothionein (MT) synthesis in a dose-dependent manner at 0.44 microM Cd or greater." |
| PMID | 2132395 |
| Year | 1990 |
| Species | Human |
| Journal | Nippon Koshu Eisei Zasshi |
| RefScore | 1 |
| Source | PArchNLP |
  ||


---

|  |  |
| --- | --- |
 Reference:17 || Sentence | "These results show that the concentration of accumulated Zn necessary for initiating production of MT is about six times that of Cd and one molecule of Cd induces thionein about 1.6 times as effectively as one molecule of Zn does." |
| PMID | 2132395 |
| Year | 1990 |
| Species | Human |
| Journal | Nippon Koshu Eisei Zasshi |
| RefScore | 2 |
| Source | PArchNLP |
  ||


---

|  |  |
| --- | --- |
 Reference:18 || Sentence | "Treatment with zinc results in induction of hepatic and intestinal metallothionein synthesis." |
| PMID | 10378366 |
| Year | 1999 |
| Species | Human |
| Journal | Z Gastroenterol |
| RefScore | 2 |
| Source | PArchNLP |
  ||


---

|  |  |
| --- | --- |
 Reference:19 || Sentence | "The lower sensitivity to cadmium of human osteosarcoma cells is attributed, at least partly, to induction of metallothionein synthesis by cadmium and zinc in this cell line; in the rat osteosarcoma cell line, they do not induce metallothionein synthesis." |
| PMID | 8400764 |
| Year | 1993 |
| Species | Rat |
|  | Human |
| Journal | Biometals |
| RefScore | 2 |
| Source | PArchNLP |
  ||


---

|  |  |
| --- | --- |
 Reference:20 || Sentence | "These experiments suggest metallothionein synthesis occurs in erythropoietin-sensitive precursor cells in the marrow in response to increased zinc accessibility." |
| PMID | 8498498 |
| Year | 1993 |
| Species | Human |
|  | Rat |
| Journal | Am J Physiol |
| RefScore | 2 |
| Source | PArchNLP |
  ||


---

|  |  |
| --- | --- |
 Reference:21 || Sentence | "Treatment of THP-1 cells with nontoxic zinc levels increased MT accumulation." |
| PMID | 8291064 |
| Year | 1994 |
| Species | Human |
| Journal | Toxicol Appl Pharmacol |
| RefScore | 1 |
| Source | PArchNLP |
  ||


---

|  |  |
| --- | --- |
 Reference:22 || Sentence | "On the other hand, the synthesis of metallothionein was activated in the presence of 100 microM zinc sulphate and above." |
| PMID | 1640929 |
| Year | 1992 |
| Species | Human |
| Journal | Mol Cell Biochem |
| RefScore | 2 |
| Source | PArchNLP |
  ||


---

|  |  |
| --- | --- |
 Reference:23 || Sentence | "Zinc pretreatment increased the level of MT gene expression as well as MT protein production." |
| PMID | 1561631 |
| Year | 1992 |
| Species | Rat |
| Journal | Toxicol Appl Pharmacol |
| RefScore | 1 |
| Source | PArchNLP |
  ||


---

|  |  |
| --- | --- |
 Reference:24 || Sentence | "Wistar rats were used to study the protective effect of zinc-induced metallothionein (MT) synthesis on gentamicin nephrotoxicity." |
| PMID | 1664118 |
| Year | 1991 |
| Species | Rat |
| Journal | Ren Fail |
| RefScore | 0 |
| Source | PArchNLP |
  ||


---

|  |  |
| --- | --- |
 Reference:25 || Sentence | "In certain experiments, the induction of renal intracellular MT synthesis by zinc pretreatment slightly decreased the renal toxicity of Cd-MT in the BSO-treated rats." |
| PMID | 2718180 |
| Year | 1989 |
| Species | Rat |
| Journal | Toxicol Appl Pharmacol |
| RefScore | 0 |
| Source | PArchNLP |
  ||


---

|  |  |
| --- | --- |
 Reference:26 || Sentence | "To examine the possible protective effect of Zn independent of induction of MT synthesis, CCl4 was administered 2 h following Zn pretreatment and the hepatotoxic response was examined 4 h later." |
| PMID | 3791046 |
| Year | 1986 |
| Species | Rat |
| Journal | Can J Physiol Pharmacol |
| RefScore | 1 |
| Source | PArchNLP |
  ||


---

|  |  |
| --- | --- |
 Reference:27 || Sentence | "Cd and Zn accumulations caused by pretreatment with estradiol in the kidney of male rats treated with Cd or Zn were so low (Cd: 38 ppb, Zn: 1.0 ppb) as to be probably unable to induce the synthesis of MT." |
| PMID | 3401240 |
| Year | 1988 |
| Species | Rat |
| Journal | Biochem Pharmacol |
| RefScore | 1 |
| Source | PArchNLP |
  ||


---

|  |  |
| --- | --- |
 Reference:28 || Sentence | "At higher Cd levels, induction of metallothionein synthesis by Zn increased fractional trapping of Cd." |
| PMID | 3952756 |
| Year | 1986 |
| Species | Rat |
| Journal | Toxicology |
| RefScore | 0 |
| Source | PArchNLP |
  ||


---

|  |  |
| --- | --- |
 Reference:29 || Sentence | "Sera from arthritic rats (14 d post-adjuvant treatment) in the presence of Zn (50 mumol/L)+dexamethasone (Dex; 1 mumol/L) increased metallothionein (MT) accumulation by 34% above that obtained with control rat serum with Zn+Dex." |
| PMID | 8597881 |
| Year | 1995 |
| Species | Rat |
| Journal | Inflamm Res |
| RefScore | 0 |
| Source | PArchNLP |
  ||


---

|  |  |
| --- | --- |
 Reference:30 || Sentence | "The accumulation of MT in hepatocytes in the presence of Zn (10 mumol/L)+Dex (1 mumol/L) was enhanced 29% and 49% by media from lipopolysaccharide (LPS)-stimulated peritoneal macrophage (PMM) and Kupffer cell cultures (KCM), respectively." |
| PMID | 8597881 |
| Year | 1995 |
| Species | Rat |
| Journal | Inflamm Res |
| RefScore | 1 |
| Source | PArchNLP |
  ||


---

|  |  |
| --- | --- |
 Reference:31 || Sentence | "GSH depletion had no effect on Cd- or zinc-induced MT synthesis." |
| PMID | 9242231 |
| Year | 1997 |
| Species | Rat |
| Journal | J Toxicol Environ Health |
| RefScore | 1 |
| Source | PArchNLP |
  ||


---

|  |  |
| --- | --- |
 Reference:32 || Sentence | "The results of this study show that prior exposure of adult rats to cadmium or zinc antagonises the effect of dimethylnitrosamine on lipid peroxidation by inducing increased metallothionein synthesis." |
| PMID | 11148932 |
| Year | 2000 |
| Species | Rat |
| Journal | Arh Hig Rada Toksikol |
| RefScore | 1 |
| Source | PArchNLP |
  ||


---

|  |  |
| --- | --- |
 Reference:33 || Sentence | "Furthermore, the reduction in plasma zinc, which depends on cellular metallothionein synthesis, occurred 4 hr after interleukin-1 administration and 6 hr after lipopolysaccharide injection or partial hepatectomy." |
| PMID | 2114349 |
| Year | 1990 |
| Species | Rat |
| Journal | Hepatology |
| RefScore | 0 |
| Source | PArchNLP |
  ||


---

|  |  |
| --- | --- |
 Reference:34 || Sentence | "In addition, the synthesis of graded amounts of MT, achieved by incubation with various concentrations of Zn or Cu, led to a high correlation between MT levels and the extent of hepatocyte survival." |
| PMID | 8560476 |
| Year | 1996 |
| Species | Rat |
| Journal | Toxicol Appl Pharmacol |
| RefScore | 1 |
| Source | PArchNLP |
  ||


---

|  |  |
| --- | --- |
 Reference:35 || Sentence | "We investigated the reciprocal effects of interleukin-6 (IL-6), glucocorticoid and zinc (Zn) on metallothionein (MT) synthesis in rats." |
| PMID | 8799367 |
| Year | 1996 |
| Species | Rat |
| Journal | Int J Immunopharmacol |
| RefScore | 1 |
| Source | PArchNLP |
  ||


---

|  |  |
| --- | --- |
 Reference:36 || Sentence | "Our data suggest that when compared to liver, the pancreas possesses a markedly higher concentration of MT-bound zinc and a greater propensity to accumulate zinc MT when zinc status is acutely elevated." |
| PMID | 6693978 |
| Year | 1984 |
| Species | Rat |
| Journal | J Nutr |
| RefScore | 2 |
| Source | PArchNLP |
  ||


---

|  |  |
| --- | --- |
 Reference:37 || Sentence | "The interaction of injected zinc salts (Zn) and cadmium salts (Cd) with regard to the synthesis of metallothionein (MT) in adult rat liver was investigated." |
| PMID | 4049424 |
| Year | 1985 |
| Species | Rat |
| Journal | Toxicology |
| RefScore | 1 |
| Source | PArchNLP |
  ||


---

|  |  |
| --- | --- |
 Reference:38 || Sentence | "The influence of a 7.7-mumole (0.5-mg) dose of parenteral zinc on the synthesis of metallothionein in rat kidney was examined." |
| PMID | 7069515 |
| Year | 1982 |
| Species | Rat |
| Journal | J Nutr |
| RefScore | 1 |
| Source | PArchNLP |
  ||


---

|  |  |
| --- | --- |
 Reference:39 || Sentence | "The object of this experiment was to determine the effects of Zn deficiency on the turnover of Cd-induced metallothionein (MT) in rat liver." |
| PMID | 6502271 |
| Year | 1984 |
| Species | Rat |
| Journal | J Nutr |
| RefScore | 1 |
| Source | PArchNLP |
  ||


---

|  |  |
| --- | --- |
 Reference:40 || Sentence | "Zn and dexamethasone induced concentration-dependent increases in the synthesis of MT with maximal increases above the 5-h control of 3.2- and 2.5-fold, respectively." |
| PMID | 7681680 |
| Year | 1993 |
| Species | Rat |
| Journal | Biol Trace Elem Res |
| RefScore | 1 |
| Source | PArchNLP |
  ||


---

|  |  |
| --- | --- |
 Reference:41 || Sentence | "Zinc pretreatment also resulted in increased MT synthesis and decreased etoposide-induced apoptosis." |
| PMID | 12700406 |
| Year | 2003 |
| Species | Human |
| Journal | Toxicol Sci |
| RefScore | 1 |
| Source | PArchNLP |
  ||


---

|  |  |
| --- | --- |
 Reference:42 || Sentence | "CONCLUSION: Zinc administration induces metallothionein synthesis but has little effect on the short-term course of experimental colitis." |
| PMID | 11346141 |
| Year | 2001 |
| Species | Rat |
| Journal | Dig Liver Dis |
| RefScore | 1 |
| Source | PArchNLP |
  ||


---

|  |  |
| --- | --- |
 Reference:43 || Sentence | "These results suggest that Zn acts both to compete for absorption on the luminal side of the intestinal epithelium and to induce the synthesis of MT." |
| PMID | 11914925 |
| Year | 2002 |
| Species | Rat |
| Journal | Histochem Cell Biol |
| RefScore | 1 |
| Source | PArchNLP |
  ||


---

|  |  |
| --- | --- |
 Reference:44 || Sentence | "Since a high-zinc diet has been shown to induce MT synthesis in extracerebral tissues but not in brain, we investigated whether it could provide an experimental basis for decreasing the hematotoxicity of carboplatin without impairing its activity against brain tumors." |
| PMID | 1733555 |
| Year | 1992 |
| Species | Mouse |
|  | Rat |
| Journal | Cancer Chemother Pharmacol |
| RefScore | 0 |
| Source | PArchNLP |
  ||


---

|  |  |
| --- | --- |
 Reference:45 || Sentence | "The amount of zinc injected was insufficient to stimulate significant synthesis of metallothionein, but similar experiments with injection of cadmium did stimulate the synthesis of metallothionein." |
| PMID | 6212927 |
| Year | 1982 |
| Species | Rat |
| Journal | Proc Natl Acad Sci U S A |
| RefScore | 1 |
| Source | PArchNLP |
  ||


---

|  |  |
| --- | --- |
 Reference:46 || Sentence | "We now report normal basal, copper-induced, and zinc-induced MT synthesis in the fibroblasts of two Indian boys and one Irish boy with typical ICC and one Indian boy with copper-associated childhood cirrhosis." |
| PMID | 8581360 |
| Year | 1995 |
| Species | Human |
| Journal | Biochem Mol Med |
| RefScore | 1 |
| Source | PArchNLP |
  ||


---

|  |  |
| --- | --- |
 Reference:47 || Sentence | "Furthermore, the gonadoliberin-A-induced inhibition of Zn-mediated metallothionein accumulation was found to correlate closely with suppresion of cell proliferation and [3H]thymidine uptake in these cells." |
| PMID | 8954150 |
| Year | 1996 |
| Species | Human |
| Journal | Eur J Biochem |
| RefScore | 2 |
| Source | PArchNLP |
  ||


---

|  |  |
| --- | --- |
 Reference:48 || Sentence | "Both GSH and Zn reduced the accumulation of Cd as well as MT in the renal cortex, with Zn causing greater reduction in Cd accumulation than that of MT." |
| PMID | 9707504 |
| Year | 1998 |
| Species | Mouse |
|  | Rat |
| Journal | Toxicol Appl Pharmacol |
| RefScore | 2 |
| Source | PArchNLP |
  ||


---

|  |  |
| --- | --- |
 Reference:49 || Sentence | "MT synthesis is induced by various stimuli such as cadmium, mercury, zinc, oxidative stress, glucocorticoid, and anticancer agents." |
| PMID | 12498322 |
| Year | 2002 |
| Species | Human |
| Journal | Tohoku J Exp Med |
| RefScore | 1 |
| Source | PArchNLP |
  ||


---

|  |  |
| --- | --- |
 Reference:50 || Sentence | "In both NPX and SO rats, zinc pretreatment caused metallothionein synthesis to increase primarily in the renal cortex and renal outer stripe of the outer medulla." |
| PMID | 1729771 |
| Year | 1992 |
| Species | Rat |
| Journal | Toxicology |
| RefScore | 0 |
| Source | PArchNLP |
  ||


---

|  |  |
| --- | --- |
 Reference:51 || Sentence | "The degree of stimulation of metallothionein synthesis in the Chang cells by cadmium and zinc was significantly higher than seen in neuroblastoma cells." |
| PMID | 2484408 |
| Year | 1989 |
| Species | Rat |
| Journal | Biol Trace Elem Res |
| RefScore | 2 |
| Source | PArchNLP |
  ||


---

|  |  |
| --- | --- |
 Reference:52 || Sentence | "Both the uptake of Cd-MT by intestine and the induction of MT synthesis in the intestine by Zn pretreatment were demonstrated by immunohistochemistry using a specific antibody to rat liver MT." |
| PMID | 1987661 |
| Year | 1991 |
| Species | Rat |
| Journal | Toxicol Appl Pharmacol |
| RefScore | 1 |
| Source | PArchNLP |
  ||


---

|  |  |
| --- | --- |
 Reference:53 || Sentence | "Thus, Zn treatment increases transcription of both MT-I and MT-II genes and the synthesis of MT-I and MT-II." |
| PMID | 3341018 |
| Year | 1988 |
| Species | Rat |
| Journal | Toxicol Appl Pharmacol |
| RefScore | 1 |
| Source | PArchNLP |
  ||


---

|  |  |
| --- | --- |
 Reference:54 || Sentence | "These data suggest that the degradation of hepatic MT (i) is primarily regulated by cellular zinc content and (ii) occurs in both lysosomal and nonlysosomal compartments." |
| PMID | 2726778 |
| Year | 1989 |
| Species | Rat |
| Journal | Proc Soc Exp Biol Med |
| RefScore | 0 |
| Source | PArchNLP |
  ||


---

|  |  |
| --- | --- |
 Reference:55 || Sentence | "The results of these studies are interpreted to suggest that zinc enhances the synthesis of MT mRNA and MT in turn may participate in zinc associated functions in neurons." |
| PMID | 8065534 |
| Year | 1994 |
| Species | Rat |
| Journal | Neurochem Res |
| RefScore | 1 |
| Source | PArchNLP |
  ||


---

|  |  |
| --- | --- |
 Reference:56 || Sentence | "Effects of metallothionein (MT) synthesis inhibiting compounds (actinomycin D, cycloheximide), MT synthesis stimulating compounds (dexamethasone, dibu-cAMP) and interfering metals (Cd, Zn) on copper accumulation were investigated in rat hepatoma tissue culture cells." |
| PMID | 7940570 |
| Year | 1994 |
| Species | Rat |
| Journal | Toxicology |
| RefScore | 1 |
| Source | PArchNLP |
  ||


---

|  |  |
| --- | --- |
 Reference:57 || Sentence | "This study provides the first demonstration that gonadoliberin inhibits the zinc-induced production of metallothionein mRNA in HepG2 and HuH7 cells." |
| PMID | 8954150 |
| Year | 1996 |
| Species | Human |
| Journal | Eur J Biochem |
| RefScore | 1 |
| Source | PArchNLP |
  |


---

|  |  |
| --- | --- |
