# Supplementary material for: Dynamic transcriptomic profiles of zebrafish gills in response to zinc depletion
Source: BMC Genomics. 2010 Oct 8;11:548. doi: 10.1186/1471-2164-11-548 (PMC3091697; doi:10.1186/1471-2164-11-548)
Supplement: Additional file 2 — Figure S1 - Interactive Direct Interaction Network of responses to zinc depletion. Mini web-site containing index.html and hyperlinked pages in subdirectory. The web site is an interactive version of Figure 6A containing curated interactions between regulated genes and respective proteins. Legend: Molecular interactions between zinc and proteins encoded by genes changed under zinc depletion. A Direct Interaction Network was created based on curated interactions contained within the PathwayArchitect database and provided through hyperlinks. Red ovals represent proteins and the blue circle symbolizes Zn(II). Dark blue squares denote 'binding', and light blue squares 'expression'; green squares stand for 'regulation', green diamonds for 'metabolism', and green circles for 'promoter binding'. Arrow heads indicate directionality of the interaction where annotated. [file 1471-2164-11-548-S2.ZIP › PathwayArchitect Zn def DIN2/410574.html]

# REGULATION:

|  |  |
| --- | --- |
| Type | REGULATION |
| Effect | Negative |


---

|  |  |
| --- | --- |
| Score | 0 |


---

|  |  |
| --- | --- |
| Reference Count | 9 |


---

|  |  |
| --- | --- |
| Mechanism | Unknown |


---

|  |  |
| --- | --- |
| Reference:0 || Sentence | "Compared with AL and/or PF rats, zinc depletion significantly reduced zinc concentrations in plasma and liver but not in kidney or intestine, and significantly reduced hepatic, renal, and intestinal metallothionein-1 mRNA levels analyzed by competitive reverse transcription-polymerase chain reaction (RT-PCR)." |
| PMID | 9649590 |
| Year | 1998 |
| Species | Rat |
| Journal | J Nutr |
| RefScore | 2 |
| Source | PArchNLP |
  |
|


---

|  |  |
| --- | --- |
 Reference:1 || Sentence | "These results indicate that marginal zinc deficiency markedly increases cadmium accumulation in various organs and reduces zinc content and MT induction in some organs." |
| PMID | 3712492 |
| Year | 1986 |
| Species | Rat |
| Journal | J Toxicol Environ Health |
| RefScore | 1 |
| Source | PArchNLP |
  ||


---

|  |  |
| --- | --- |
 Reference:2 || Sentence | "Zinc pretreatment significantly reduced the increased levels of thiobarbituric acid reactive substance and conjugated diene during ischemia-reperfusion and increased metallothionein levels compared with saline injection." |
| PMID | 10575193 |
| Year | 1999 |
| Species | Rat |
| Journal | Am J Nephrol |
| RefScore | 1 |
| Source | PArchNLP |
  ||


---

|  |  |
| --- | --- |
 Reference:3 || Sentence | "These studies suggest that the hepatoprotective effect of zinc against bromobenzene toxicity does not involve altered binding of the reactive toxic metabolite to glutathione or metallothionein, but it may be mediated by the inhibitory effect of zinc on the microsomal cytochrome P-450-dependent drug metabolizing system." |
| PMID | 3988000 |
| Year | 1985 |
| Species | Rat |
| Journal | Fundam Appl Toxicol |
| RefScore | 1 |
| Source | PArchNLP |
  ||


---

|  |  |
| --- | --- |
 Reference:4 || Sentence | "Prior induction of renal MT by treatment with zinc (20 mg of Zn per kg as ZnSO4, i.p. 16 hr before CdMT injection) markedly reduced non-MT binding of Cd++ in kidneys of treated animals and inhibited the alterations in urine volume and low molecular weight protein reabsorption induced by CdMT." |
| PMID | 6707945 |
| Year | 1984 |
| Species | Rat |
| Journal | J Pharmacol Exp Ther |
| RefScore | 0 |
| Source | PArchNLP |
  ||


---

|  |  |
| --- | --- |
 Reference:5 || Sentence | "Free zinc inhibits respiration at concentrations commensurate with the zinc content of either MT or the isolated beta-domain, indicating that MT inhibition involves zinc delivery to mitochondria." |
| PMID | 11226237 |
| Year | 2001 |
| Species | Rat |
| Journal | Proc Natl Acad Sci U S A |
| RefScore | 1 |
| Source | PArchNLP |
  ||


---

|  |  |
| --- | --- |
 Reference:6 || Sentence | "Marginal zinc deficiency rapidly caused a major decrease in MT-I levels in the blood cells and to a lesser extent in urine." |
| PMID | 3309208 |
| Year | 1987 |
| Species | Rat |
| Journal | J Nutr |
| RefScore | 0 |
| Source | PArchNLP |
  ||


---

|  |  |
| --- | --- |
 Reference:7 || Sentence | "Thus zinc deficiency state reduces zinc and MT concentrations in luminal fluid of epididymis and serum." |
| PMID | 15255602 |
| Year | 2003 |
| Species | Rat |
| Journal | Indian J Exp Biol |
| RefScore | 0 |
| Source | PArchNLP |
  ||


---

|  |  |
| --- | --- |
 Reference:8 || Sentence | RESULTS: The zinc supplement reduced SLC30A1 mRNA (1.4-fold) together with SLC30A1, SLC30A5, and SLC39A4 protein (1.8-fold, 3.7-fold, and to undetectable levels, respectively) in ileal mucosa and increased metallothionein mRNA (1.7-fold). |
| PMID | 15753530 |
| Year | 2005 |
| Species | Human |
| Journal | Gut |
| RefScore | 1 |
| Source | PArchNLP |
  |


---

|  |  |
| --- | --- |
