# Supplementary material for: Dynamic transcriptomic profiles of zebrafish gills in response to zinc depletion
Source: BMC Genomics. 2010 Oct 8;11:548. doi: 10.1186/1471-2164-11-548 (PMC3091697; doi:10.1186/1471-2164-11-548)
Supplement: Additional file 2 — Figure S1 - Interactive Direct Interaction Network of responses to zinc depletion. Mini web-site containing index.html and hyperlinked pages in subdirectory. The web site is an interactive version of Figure 6A containing curated interactions between regulated genes and respective proteins. Legend: Molecular interactions between zinc and proteins encoded by genes changed under zinc depletion. A Direct Interaction Network was created based on curated interactions contained within the PathwayArchitect database and provided through hyperlinks. Red ovals represent proteins and the blue circle symbolizes Zn(II). Dark blue squares denote 'binding', and light blue squares 'expression'; green squares stand for 'regulation', green diamonds for 'metabolism', and green circles for 'promoter binding'. Arrow heads indicate directionality of the interaction where annotated. [file 1471-2164-11-548-S2.ZIP › PathwayArchitect Zn def DIN2/410575.html]

# EXPRESSION:

|  |  |
| --- | --- |
| Type | EXPRESSION |
| Effect | None |


---

|  |  |
| --- | --- |
| Score | 0 |


---

|  |  |
| --- | --- |
| Reference Count | 33 |


---

|  |  |
| --- | --- |
| Mechanism | Unknown |


---

|  |  |
| --- | --- |
| Reference:0 || Sentence | "In conclusion, zinc deficiency induces upregulation of metallothionein-1 gene expression in response to interleukin-1alpha challenge in rats." |
| PMID | 9649590 |
| Year | 1998 |
| Species | Rat |
| Journal | J Nutr |
| RefScore | 0 |
| Source | PArchNLP |
  |
|


---

|  |  |
| --- | --- |
 Reference:1 || Sentence | "Together, these findings provide strong evidence for a regulatory role of metallothionein in zinc absorption and homeostasis, where dietary zinc influences subcellular events in mucosa affecting regulation of the metallothionein gene." |
| PMID | 6115000 |
| Year | 1981 |
| Species | Rat |
| Journal | J Nutr |
| RefScore | 1 |
| Source | PArchNLP |
  ||


---

|  |  |
| --- | --- |
 Reference:2 || Sentence | "Zinc pretreatment had little effect on MT RNA in the testes, and such pretreatments did not alter testicular cadmium-binding protein capacity." |
| PMID | 7515252 |
| Year | 1994 |
| Species | Rat |
| Journal | Hum Exp Toxicol |
| RefScore | 0 |
| Source | PArchNLP |
  ||


---

|  |  |
| --- | --- |
 Reference:3 || Sentence | "In cells stably transfected with a human cyclin D1 cDNA under the control of a metal-inducible metallothionein promoter, cyclin D1 expression was increased 2-4-fold following treatment with zinc." |
| PMID | 9815758 |
| Year | 1997 |
| Species | Human |
| Journal | Clin Cancer Res |
| RefScore | 0 |
| Source | PArchNLP |
  ||


---

|  |  |
| --- | --- |
 Reference:4 || Sentence | "The metallothionein gene is transcriptionally regulated by zinc." |
| PMID | 9521632 |
| Year | 1998 |
| Species | Human |
| Journal | J Nutr |
| RefScore | 1 |
| Source | PArchNLP |
  ||


---

|  |  |
| --- | --- |
 Reference:5 || Sentence | "A mutant strain (LEC) of rats was found to possess the feature of low degree of the zinc-induced hepatic metallothionein (MT) gene expression due to an alteration of the transcription factor concerned in the gene expression." |
| PMID | 7564896 |
| Year | 1995 |
| Species | Rat |
| Journal | Life Sci |
| RefScore | 0 |
| Source | PArchNLP |
  ||


---

|  |  |
| --- | --- |
 Reference:6 || Sentence | "Thus, the effects of Cd on expression of c-myc and c-jun in rat L6 myoblasts, and the effect of preactivation of the MT gene by Zn treatment on such oncogene expression, were studied." |
| PMID | 8691507 |
| Year | 1996 |
| Species | Rat |
| Journal | J Toxicol Environ Health |
| RefScore | 0 |
| Source | PArchNLP |
  ||


---

|  |  |
| --- | --- |
 Reference:7 || Sentence | "Furthermore, they suggest that transcriptional regulation of the metallothionein gene and other genes with metal regulatory elements involves a direct interaction between the dietary supply and intranuclear factors that bind zinc." |
| PMID | 1370327 |
| Year | 1992 |
| Species | Rat |
| Journal | J Nutr |
| RefScore | 1 |
| Source | PArchNLP |
  ||


---

|  |  |
| --- | --- |
 Reference:8 || Sentence | "Metal response element-binding transcription factor-1 (MTF-1) binds specifically to metal response elements (MREs) and transactivates metallothionein (MT) gene expression in response to zinc and cadmium." |
| PMID | 10026134 |
| Year | 1999 |
| Species | Human |
| Journal | J Biol Chem |
| RefScore | 1 |
| Source | PArchNLP |
  ||


---

|  |  |
| --- | --- |
 Reference:9 || Sentence | "Expression of hepatic metallothionein isoform mRNAs suggested MT-1 responded to zinc and MT-2 responded to cytokines." |
| PMID | 8463865 |
| Year | 1993 |
| Species | Rat |
| Journal | J Nutr |
| RefScore | 0 |
| Source | PArchNLP |
  ||


---

|  |  |
| --- | --- |
 Reference:10 || Sentence | "These results indicate that metallothionein gene expression in both the marrow and the liver responds to dietary zinc status." |
| PMID | 8463865 |
| Year | 1993 |
| Species | Rat |
| Journal | J Nutr |
| RefScore | 1 |
| Source | PArchNLP |
  ||


---

|  |  |
| --- | --- |
 Reference:11 || Sentence | "Chromomycin A3 but not Hoechst 33258, strongly inhibited the zinc-dependent transcriptional activity of the sheep metallothionein-Ia promoter in reporter gene assays of transfected cells." |
| PMID | 7510966 |
| Year | 1994 |
| Species | Human |
| Journal | Biochem Pharmacol |
| RefScore | 1 |
| Source | PArchNLP |
  ||


---

|  |  |
| --- | --- |
 Reference:12 || Sentence | "We therefore performed representational difference analysis (RDA) to identify downstream genetic targets of E2A-HLF, using a murine FL5.12 pro-B cell line that had been stably transfected with E2A-HLF cDNA under the control of a zinc-regulated metallothionein promoter." |
| PMID | 11486032 |
| Year | 2001 |
| Species | Mouse |
|  | Human |
| Journal | Mol Cell Biol |
| RefScore | 1 |
| Source | PArchNLP |
  ||


---

|  |  |
| --- | --- |
 Reference:13 || Sentence | "The ability of human monocytes to be activated by bacterial lipopolysaccharide (LPS) treatment provided a model to investigate the effect of zinc on both cellular activation (H2O2 production) and MT expression." |
| PMID | 8291064 |
| Year | 1994 |
| Species | Human |
| Journal | Toxicol Appl Pharmacol |
| RefScore | 2 |
| Source | PArchNLP |
  ||


---

|  |  |
| --- | --- |
 Reference:14 || Sentence | "We conclude that MT expression is associated with monocyte activation, and exposure to zinc or cadmium interferes with the ability of monocytes to respond to activation signals." |
| PMID | 8291064 |
| Year | 1994 |
| Species | Human |
| Journal | Toxicol Appl Pharmacol |
| RefScore | 1 |
| Source | PArchNLP |
  ||


---

|  |  |
| --- | --- |
 Reference:15 || Sentence | "Transcription of metallothionein genes is activated by heavy metals such as zinc and cadmium, and a DNA element called metal responsive element (MRE) is essential for this process." |
| PMID | 1459136 |
| Year | 1992 |
| Species | Human |
| Journal | Eur J Biochem |
| RefScore | 1 |
| Source | PArchNLP |
  ||


---

|  |  |
| --- | --- |
 Reference:16 || Sentence | "Zinc pretreatment, at levels which highly activated MT expression, had no effect on arsenite-induced cytotoxicity." |
| PMID | 9848127 |
| Year | 1998 |
| Species | Rat |
| Journal | Toxicol Sci |
| RefScore | 0 |
| Source | PArchNLP |
  ||


---

|  |  |
| --- | --- |
 Reference:17 || Sentence | "Zinc pretreatment increased the level of MT gene expression as well as MT protein production." |
| PMID | 1561631 |
| Year | 1992 |
| Species | Rat |
| Journal | Toxicol Appl Pharmacol |
| RefScore | 1 |
| Source | PArchNLP |
  ||


---

|  |  |
| --- | --- |
 Reference:18 || Sentence | "The influence of maternal dietary zinc intake and recombinant human interleukin-1 alpha (rhIL-1 alpha) administration on metallothionein gene expression and the distribution of 65Zn were investigated." |
| PMID | 3264852 |
| Year | 1988 |
| Species | Rat |
| Journal | J Nutr |
| RefScore | 0 |
| Source | PArchNLP |
  ||


---

|  |  |
| --- | --- |
 Reference:19 || Sentence | "Exposure of the adult rat brain parenchyma to zinc induces an increase in the intracerebral expression of the metal-binding protein, metallothionein, which is normally confined to astrocytes, ependymal cells, choroid plexus epithelial cells, and brain endothelial cells." |
| PMID | 7782107 |
| Year | 1995 |
| Species | Rat |
| Journal | Glia |
| RefScore | 1 |
| Source | PArchNLP |
  ||


---

|  |  |
| --- | --- |
 Reference:20 || Sentence | "These findings suggest that, in LEC rats, Zn may contribute to cytoprotection through the regulation of MT expression which may provide a cellular defence strategy in response to DNA damage." |
| PMID | 15062872 |
| Year | 2004 |
| Species | Rat |
| Journal | Biochim Biophys Acta |
| RefScore | 0 |
| Source | PArchNLP |
  ||


---

|  |  |
| --- | --- |
 Reference:21 || Sentence | "We have evaluated the effects of IL-6 and IL-1 alpha as well as extracellular zinc and glucocorticoid hormone on metallothionein gene expression and cellular zinc accumulation in rat hepatocyte monolayer cultures." |
| PMID | 2326272 |
| Year | 1990 |
| Species | Rat |
| Journal | Proc Natl Acad Sci U S A |
| RefScore | 0 |
| Source | PArchNLP |
  ||


---

|  |  |
| --- | --- |
 Reference:22 || Sentence | "Synthesis of human growth hormone was induced further by zinc, which normally induces metallothionein gene expression." |
| PMID | 2104209 |
| Year | 1990 |
| Species | Mouse |
|  | Human |
| Journal | Chin J Biotechnol |
| RefScore | 1 |
| Source | PArchNLP |
  ||


---

|  |  |
| --- | --- |
 Reference:23 || Sentence | "The mouse metallothionein promoter was not successful in effecting human PNP expression in CHO cells but provided substantial human PNP activity in mouse cells and was inducible by incubation with zinc." |
| PMID | 3929070 |
| Year | 1985 |
| Species | Mouse |
|  | Human |
| Journal | Mol Cell Biol |
| RefScore | 2 |
| Source | PArchNLP |
  ||


---

|  |  |
| --- | --- |
 Reference:24 || Sentence | "In addition to Cd, Zn and Cu were also able to induce the expression of metallothionein to various degrees." |
| PMID | 8560508 |
| Year | 1995 |
| Species | Rat |
| Journal | Toxicology |
| RefScore | 1 |
| Source | PArchNLP |
  ||


---

|  |  |
| --- | --- |
 Reference:25 || Sentence | "We compared the effects of idiorrhythmic dose-rate feeding and conventional dose-response on the induction of intestinal metallothionein (iMT), expression of aortal heat-shock protein mRNA (HSP70mRNA) induced by restraint stress, and accumulation of Zn in the femur and incisor of young growing male rats." |
| PMID | 9292769 |
| Year | 1997 |
| Species | Rat |
| Journal | Br J Nutr |
| RefScore | 0 |
| Source | PArchNLP |
  ||


---

|  |  |
| --- | --- |
 Reference:26 || Sentence | "Quercetin decreased zinc-stimulated metallothionein expression and had no effect on the cadmium induction of metallothionein." |
| PMID | 11817679 |
| Year | 2001 |
| Species | Human |
| Journal | Biol Trace Elem Res |
| RefScore | 1 |
| Source | PArchNLP |
  ||


---

|  |  |
| --- | --- |
 Reference:27 || Sentence | "Free zinc can control MT gene expression by interacting with metal-sensitive transcription factors." |
| PMID | 11311571 |
| Year | 2001 |
| Species | Human |
|  | Rat |
| Journal | Toxicol Lett |
| RefScore | 1 |
| Source | PArchNLP |
  ||


---

|  |  |
| --- | --- |
 Reference:28 || Sentence | "The released zinc or cadmium probably then stimulates further MT gene expression." |
| PMID | 11311571 |
| Year | 2001 |
| Species | Human |
|  | Rat |
| Journal | Toxicol Lett |
| RefScore | 1 |
| Source | PArchNLP |
  ||


---

|  |  |
| --- | --- |
 Reference:29 || Sentence | "Utilizing in situ hybridization, we show that the IL-1 alpha and beta forms and zinc induce metallothionein mRNA expression TEC." |
| PMID | 1502195 |
| Year | 1992 |
| Species | Human |
| Journal | Proc Natl Acad Sci U S A |
| RefScore | 2 |
| Source | PArchNLP |
  ||


---

|  |  |
| --- | --- |
 Reference:30 || Sentence | "Expression of the metallothionein gene is altered by zinc status and the protein appears to have a function in intestinal cells." |
| PMID | 2420502 |
| Year | 1986 |
| Species | Human |
|  | Rat |
| Journal | Clin Physiol Biochem |
| RefScore | 0 |
| Source | PArchNLP |
  ||


---

|  |  |
| --- | --- |
 Reference:31 || Sentence | "Thus, Zn treatment increases transcription of both MT-I and MT-II genes and the synthesis of MT-I and MT-II." |
| PMID | 3341018 |
| Year | 1988 |
| Species | Rat |
| Journal | Toxicol Appl Pharmacol |
| RefScore | 1 |
| Source | PArchNLP |
  ||


---

|  |  |
| --- | --- |
 Reference:32 || Sentence | "The results indicate that MT gene expression can be induced by zinc during fetal life and that its expression without exogenous inducers cannot be ascribed to circulating corticosteroids." |
| PMID | 8487092 |
| Year | 1993 |
| Species | Rat |
| Journal | J Nutr |
| RefScore | 0 |
| Source | PArchNLP |
  |


---

|  |  |
| --- | --- |
