# Supplementary material for: Dynamic transcriptomic profiles of zebrafish gills in response to zinc depletion
Source: BMC Genomics. 2010 Oct 8;11:548. doi: 10.1186/1471-2164-11-548 (PMC3091697; doi:10.1186/1471-2164-11-548)
Supplement: Additional file 2 — Figure S1 - Interactive Direct Interaction Network of responses to zinc depletion. Mini web-site containing index.html and hyperlinked pages in subdirectory. The web site is an interactive version of Figure 6A containing curated interactions between regulated genes and respective proteins. Legend: Molecular interactions between zinc and proteins encoded by genes changed under zinc depletion. A Direct Interaction Network was created based on curated interactions contained within the PathwayArchitect database and provided through hyperlinks. Red ovals represent proteins and the blue circle symbolizes Zn(II). Dark blue squares denote 'binding', and light blue squares 'expression'; green squares stand for 'regulation', green diamonds for 'metabolism', and green circles for 'promoter binding'. Arrow heads indicate directionality of the interaction where annotated. [file 1471-2164-11-548-S2.ZIP › PathwayArchitect Zn def DIN2/410864.html]

# METABOLISM:

|  |  |
| --- | --- |
| Type | METABOLISM |
| Effect | None |


---

|  |  |
| --- | --- |
| Score | 0 |


---

|  |  |
| --- | --- |
| Reference Count | 14 |


---

|  |  |
| --- | --- |
| Mechanism | Unknown |


---

|  |  |
| --- | --- |
| Reference:0 || Sentence | "Induced MT may be involved in Zn metabolism and transport in the prostate gland." |
| PMID | 11368269 |
| Year | 2001 |
| Species | Rat |
| Journal | Cytobios |
| RefScore | 0 |
| Source | PArchNLP |
  |
|


---

|  |  |
| --- | --- |
 Reference:1 || Sentence | "These results suggest that zinc accumulation by liver cells is mediated by metallothionein produced in response to a macrophage factor, which is elicited by endotoxin." |
| PMID | 3365257 |
| Year | 1988 |
| Species | Rat |
| Journal | Biochem Biophys Res Commun |
| RefScore | 0 |
| Source | PArchNLP |
  ||


---

|  |  |
| --- | --- |
 Reference:2 || Sentence | "These results suggest that both MT isoforms play a similar role in the metabolism of Zn during early development and the relative abundance of the MT isoforms may be a species-specific phenomenon." |
| PMID | 8398071 |
| Year | 1993 |
| Species | Rat |
| Journal | Biochem Cell Biol |
| RefScore | 1 |
| Source | PArchNLP |
  ||


---

|  |  |
| --- | --- |
 Reference:3 || Sentence | "Regulation of metallothionein synthesis and concomitant changes in the kinetics of zinc metabolism are influenced by dibutyryl cAMP, epinephrine, glucagon and dexamethasone in both intact rats and isolated rat liver parenchymal cells." |
| PMID | 2822465 |
| Year | 1987 |
| Species | Rat |
| Journal | Experientia Suppl |
| RefScore | 1 |
| Source | PArchNLP |
  ||


---

|  |  |
| --- | --- |
 Reference:4 || Sentence | "In late gestation, MT serves to bind Cu and Zn from the pre-existing pools of these metals, as well as to accumulate additional amounts of Zn." |
| PMID | 2959540 |
| Year | 1987 |
| Species | Rat |
| Journal | Experientia Suppl |
| RefScore | 1 |
| Source | PArchNLP |
  ||


---

|  |  |
| --- | --- |
 Reference:5 || Sentence | "We investigated the reciprocal effects of interleukin-6 (IL-6), glucocorticoid and zinc (Zn) on metallothionein (MT) synthesis in rats." |
| PMID | 8799367 |
| Year | 1996 |
| Species | Rat |
| Journal | Int J Immunopharmacol |
| RefScore | 1 |
| Source | PArchNLP |
  ||


---

|  |  |
| --- | --- |
 Reference:6 || Sentence | "Our data suggest that when compared to liver, the pancreas possesses a markedly higher concentration of MT-bound zinc and a greater propensity to accumulate zinc MT when zinc status is acutely elevated." |
| PMID | 6693978 |
| Year | 1984 |
| Species | Rat |
| Journal | J Nutr |
| RefScore | 2 |
| Source | PArchNLP |
  ||


---

|  |  |
| --- | --- |
 Reference:7 || Sentence | "To investigate the relationship between glutathione (GSH) depletion and metallothionein (MT) synthesis, the effects of substrates and an inhibitor of GSH S-transferases on concentrations of hepatic GSH, zinc (Zn) and MT were studied in rats." |
| PMID | 7586048 |
| Year | 1995 |
| Species | Rat |
| Journal | Chem Biol Interact |
| RefScore | 1 |
| Source | PArchNLP |
  ||


---

|  |  |
| --- | --- |
 Reference:8 || Sentence | "Based on previous findings that liver zinc and metallothionein (MT) levels increase after tumor transplantation, zinc metabolism in tumor-bearing mice was studied to clarify the role of zinc-MT in host defense systems." |
| PMID | 10772964 |
| Year | 2000 |
| Species | Mouse |
|  | Rat |
| Journal | Biochem Biophys Res Commun |
| RefScore | 2 |
| Source | PArchNLP |
  ||


---

|  |  |
| --- | --- |
 Reference:9 || Sentence | "This zinc accumulation is made possible by high expression of the zinc-binding protein, metallothionein (MT)." |
| PMID | 11208584 |
| Year | 2001 |
| Species | Mouse |
|  | Human |
| Journal | Am J Physiol Regul Integr Comp Physiol |
| RefScore | 2 |
| Source | PArchNLP |
  ||


---

|  |  |
| --- | --- |
 Reference:10 || Sentence | "Zinc metabolism in the cells is largely regulated by ubiquitous small proteins, metallothioneins (MT)." |
| PMID | 11994011 |
| Year | 2002 |
| Species | Human |
| Journal | Biochemistry |
| RefScore | 2 |
| Source | PArchNLP |
  ||


---

|  |  |
| --- | --- |
 Reference:11 || Sentence | "Metallothioneins (MTs), which probably participate in zinc metabolism, were induced by endotoxin administration." |
| PMID | 2822467 |
| Year | 1987 |
| Species | Rat |
|  | Human |
| Journal | Experientia Suppl |
| RefScore | 0 |
| Source | PArchNLP |
  ||


---

|  |  |
| --- | --- |
 Reference:12 || Sentence | "These findings offer support for the involvement of MTN in zinc metabolism." |
| PMID | 978266 |
| Year | 1976 |
| Species | Rat |
| Journal | J Nutr |
| RefScore | 0 |
| Source | PArchNLP |
  ||


---

|  |  |
| --- | --- |
 Reference:13 || Sentence | Metallothionein immunoperoxidase staining technique was used to localize the accumulation of Cd and Zn in the nephrons. |
| PMID | 16959594 |
| Year | 2006 |
| Species | Human |
| Journal | J Trace Elem Med Biol |
| RefScore | 2 |
| Source | PArchNLP |
  |


---

|  |  |
| --- | --- |
