# Supplementary material for: Dynamic transcriptomic profiles of zebrafish gills in response to zinc depletion
Source: BMC Genomics. 2010 Oct 8;11:548. doi: 10.1186/1471-2164-11-548 (PMC3091697; doi:10.1186/1471-2164-11-548)
Supplement: Additional file 2 — Figure S1 - Interactive Direct Interaction Network of responses to zinc depletion. Mini web-site containing index.html and hyperlinked pages in subdirectory. The web site is an interactive version of Figure 6A containing curated interactions between regulated genes and respective proteins. Legend: Molecular interactions between zinc and proteins encoded by genes changed under zinc depletion. A Direct Interaction Network was created based on curated interactions contained within the PathwayArchitect database and provided through hyperlinks. Red ovals represent proteins and the blue circle symbolizes Zn(II). Dark blue squares denote 'binding', and light blue squares 'expression'; green squares stand for 'regulation', green diamonds for 'metabolism', and green circles for 'promoter binding'. Arrow heads indicate directionality of the interaction where annotated. [file 1471-2164-11-548-S2.ZIP › PathwayArchitect Zn def DIN2/410865.html]

# BINDING:

|  |  |
| --- | --- |
| Type | BINDING |
| Effect | None |


---

|  |  |
| --- | --- |
| Score | 0 |


---

|  |  |
| --- | --- |
| Reference Count | 45 |


---

|  |  |
| --- | --- |
| Mechanism | Unknown |


---

|  |  |
| --- | --- |
| Reference:0 || Sentence | "Binding Zn to MT may indicate the involvement of MT in the metabolism and transport of zinc, an effect which may be modified by treatment." |
| PMID | 11368269 |
| Year | 2001 |
| Species | Rat |
| Journal | Cytobios |
| RefScore | 1 |
| Source | PArchNLP |
  |
|


---

|  |  |
| --- | --- |
 Reference:1 || Sentence | "The interactions between two essential metals, Cu and Zn, and the localization and concentration of metallothionein have been studied in rat liver and kidney." |
| PMID | 11432643 |
| Year | 2001 |
| Species | Rat |
| Journal | Histochem J |
| RefScore | 0 |
| Source | PArchNLP |
  ||


---

|  |  |
| --- | --- |
 Reference:2 || Sentence | "In view of the fact that the bovine pineal gland and retina continually synthesize metallothionein and other low molecular weight zinc binding proteins, we propose that zinc and metallothionein participate in signal transduction in the retina and pineal gland." |
| PMID | 1564629 |
| Year | 1992 |
| Species | Rat |
| Journal | J Pineal Res |
| RefScore | 1 |
| Source | PArchNLP |
  ||


---

|  |  |
| --- | --- |
 Reference:3 || Sentence | "Relative zinc-binding activities of high-molecular-weight zinc-binding ligand (HMW-ZBL), metallothionein (MT) and low-molecular-weight zinc-binding ligand (LMW-ZBL) in the cytosols of rat small intestines under various experimental conditions were examined." |
| PMID | 2879687 |
| Year | 1986 |
| Species | Rat |
| Journal | Comp Biochem Physiol C |
| RefScore | 1 |
| Source | PArchNLP |
  ||


---

|  |  |
| --- | --- |
 Reference:4 || Sentence | "Zinc-binding activities of MT decreased and those of LMW-ZBL increased in the intestinal cytosols from most of the experimental rat groups after incubating at 37 degrees C for 2 hr." |
| PMID | 2879687 |
| Year | 1986 |
| Species | Rat |
| Journal | Comp Biochem Physiol C |
| RefScore | 0 |
| Source | PArchNLP |
  ||


---

|  |  |
| --- | --- |
 Reference:5 || Sentence | "The relative zinc-binding activity of MT increased with increasing doses of injected zinc and decreased with orally-administered zinc." |
| PMID | 2879687 |
| Year | 1986 |
| Species | Rat |
| Journal | Comp Biochem Physiol C |
| RefScore | 2 |
| Source | PArchNLP |
  ||


---

|  |  |
| --- | --- |
 Reference:6 || Sentence | "Maternal and fetal tissues were assessed for copper concentrations, the activities of the cuproenzymes copper, zinc superoxide dismutase and ceruloplasmin, and the copper binding protein metallothionein." |
| PMID | 8118109 |
| Year | 1993 |
| Species | Rat |
| Journal | Reprod Toxicol |
| RefScore | 1 |
| Source | PArchNLP |
  ||


---

|  |  |
| --- | --- |
 Reference:7 || Sentence | "Metallothionein is a low molecular mass protein inducible mainly by heavy metals, having high affinity for binding cadmium, zinc and copper." |
| PMID | 7857203 |
| Year | 1994 |
| Species | Rat |
| Journal | Arch Toxicol |
| RefScore | 0 |
| Source | PArchNLP |
  ||


---

|  |  |
| --- | --- |
 Reference:8 || Sentence | "The present experiments examine the interaction of CRIP and metallothionein with zinc to evaluate their potential roles in the mechanism of zinc absorption." |
| PMID | 1729476 |
| Year | 1992 |
| Species | Rat |
| Journal | J Nutr |
| RefScore | 1 |
| Source | PArchNLP |
  ||


---

|  |  |
| --- | --- |
 Reference:9 || Sentence | "The temporal accumulation of zinc is correlated with the induction of MT, and the accumulated zinc binds to MT." |
| PMID | 3365257 |
| Year | 1988 |
| Species | Rat |
| Journal | Biochem Biophys Res Commun |
| RefScore | 1 |
| Source | PArchNLP |
  ||


---

|  |  |
| --- | --- |
 Reference:10 || Sentence | "Metallothionein is a cysteine-rich, low molecular weight protein that binds zinc, copper and cadmium." |
| PMID | 6413769 |
| Year | 1983 |
| Species | Rat |
|  | Human |
| Journal | J Inherit Metab Dis |
| RefScore | 0 |
| Source | PArchNLP |
  ||


---

|  |  |
| --- | --- |
 Reference:11 || Sentence | "Metallothionein (MT) is a cysteine-rich, low molecular weight protein that binds zinc, copper, and cadmium." |
| PMID | 8236816 |
| Year | 1993 |
| Species | Human |
| Journal | Virchows Arch A Pathol Anat Histopathol |
| RefScore | 1 |
| Source | PArchNLP |
  ||


---

|  |  |
| --- | --- |
 Reference:12 || Sentence | "In late gestation, MT serves to bind Cu and Zn from the preexisting hepatic pools of these metals, as well as to accumulate additional amounts of both metals." |
| PMID | 3986661 |
| Year | 1985 |
| Species | Rat |
| Journal | Can J Biochem Cell Biol |
| RefScore | 0 |
| Source | PArchNLP |
  ||


---

|  |  |
| --- | --- |
 Reference:13 || Sentence | "The interaction of injected zinc and cadmium with metallothionein was investigated in newborn rats." |
| PMID | 6883574 |
| Year | 1983 |
| Species | Rat |
| Journal | Chem Biol Interact |
| RefScore | 1 |
| Source | PArchNLP |
  ||


---

|  |  |
| --- | --- |
 Reference:14 || Sentence | "The Zn bound to Cd-induced MT was reduced to 30% in the liver and to 60% in the kidney of the Zn-deficient rats (-Zn + Cd) as compared with that of the Zn-adequate rats (+Zn + Cd)." |
| PMID | 2774670 |
| Year | 1989 |
| Species | Rat |
| Journal | Arch Environ Contam Toxicol |
| RefScore | 2 |
| Source | PArchNLP |
  ||


---

|  |  |
| --- | --- |
 Reference:15 || Sentence | "To assay metallothioneins and copper, cadmium, and zinc bound to metallothioneins, we used high-performance liquid chromatography directly coupled to flame atomic absorption spectrometry." |
| PMID | 1497498 |
| Year | 1992 |
| Species | Human |
| Journal | Arch Neurol |
| RefScore | 1 |
| Source | PArchNLP |
  ||


---

|  |  |
| --- | --- |
 Reference:16 || Sentence | "Zinc acts by inducing intestinal cell metallothionein, which binds copper with high affinity, blocking its absorption, and causing its excretion in the stool." |
| PMID | 2258535 |
| Year | 1990 |
| Species | Human |
| Journal | J Am Coll Nutr |
| RefScore | 0 |
| Source | PArchNLP |
  ||


---

|  |  |
| --- | --- |
 Reference:17 || Sentence | "This study supported previous findings that MT was secreted predominantly from the prostate and induced by inflammation of the prostate gland or seminal vesicles; the findings suggest that MT binds mainly to zinc and is one of the zinc-binding proteins in seminal plasma." |
| PMID | 7614399 |
| Year | 1994 |
| Species | Human |
| Journal | Int J Urol |
| RefScore | 1 |
| Source | PArchNLP |
  ||


---

|  |  |
| --- | --- |
 Reference:18 || Sentence | "The Menkes' cells preferentially take up not only copper but also, on exposure to elevated metal concentrations, the other metallothionein-binding metals, zinc and cadmium." |
| PMID | 6279642 |
| Year | 1982 |
| Species | Human |
| Journal | J Biol Chem |
| RefScore | 3 |
| Source | PArchNLP |
  ||


---

|  |  |
| --- | --- |
 Reference:19 || Sentence | "Metallothionein (MT) is a two-domain protein with zinc thiolate clusters that bind and release zinc depending on the redox states of the sulfur ligands." |
| PMID | 12081484 |
| Year | 2002 |
| Species | Human |
| Journal | Biochemistry |
| RefScore | 2 |
| Source | PArchNLP |
  ||


---

|  |  |
| --- | --- |
 Reference:20 || Sentence | "We have thus demonstrated that cadmium-induced metallothionein binds zinc in the trophoblast, making this essential element less available to the fetal circulation." |
| PMID | 1566278 |
| Year | 1992 |
| Species | Human |
| Journal | Toxicology |
| RefScore | 1 |
| Source | PArchNLP |
  ||


---

|  |  |
| --- | --- |
 Reference:21 || Sentence | "Metallothioneins that bind copper and zinc have an Mr of 6500 daltons, consist of a single polypeptide chain of 61 amino acids, 25-30 percent of whose residues are cysteine, have a metal-binding capacity of between 5 and 7 g atoms/mol, and contain no disulfide bonds or aromatic amino acids." |
| PMID | 3283743 |
| Year | 1988 |
| Species | Human |
| Journal | Prog Clin Biol Res |
| RefScore | 2 |
| Source | PArchNLP |
  ||


---

|  |  |
| --- | --- |
 Reference:22 || Sentence | "In late gestation, MT serves to bind Cu and Zn from the pre-existing pools of these metals, as well as to accumulate additional amounts of Zn." |
| PMID | 2959540 |
| Year | 1987 |
| Species | Rat |
| Journal | Experientia Suppl |
| RefScore | 1 |
| Source | PArchNLP |
  ||


---

|  |  |
| --- | --- |
 Reference:23 || Sentence | "The hypothesis that two of these cytosolic zinc-binding species, a metallothionein-like protein and a putative zinc-glutathione complex, may be responsible for the sequestration of zinc in the hippocampus was tested." |
| PMID | 6726352 |
| Year | 1984 |
| Species | Rat |
| Journal | J Neurosci |
| RefScore | 2 |
| Source | PArchNLP |
  ||


---

|  |  |
| --- | --- |
 Reference:24 || Sentence | "The results show the transfer of the essential metal, zinc from hepatic MT to other proteins and the specific binding of cadmium, the non-essential metal to MT during postnatal development in rats." |
| PMID | 4024125 |
| Year | 1985 |
| Species | Rat |
| Journal | Toxicology |
| RefScore | 1 |
| Source | PArchNLP |
  ||


---

|  |  |
| --- | --- |
 Reference:25 || Sentence | "Metallothionein (MT), which binds zinc and other metals, was localized within the rat prostatic complex at light and electron microscopic levels utilizing immunocytochemistry." |
| PMID | 3537994 |
| Year | 1986 |
| Species | Rat |
| Journal | Prostate |
| RefScore | 0 |
| Source | PArchNLP |
  ||


---

|  |  |
| --- | --- |
 Reference:26 || Sentence | "These localizations suggest that MT binds zinc both intra- and extracellularly, where it may function in zinc storage and metabolism." |
| PMID | 3537994 |
| Year | 1986 |
| Species | Rat |
| Journal | Prostate |
| RefScore | 2 |
| Source | PArchNLP |
  ||


---

|  |  |
| --- | --- |
 Reference:27 || Sentence | "Liver cytosolic Sephadex G-75 patterns of rats injected with both zinc and arsenic gave separate peaks for arsenic and zinc which suggest that in vivo arsenic binding to MT may be insignificant." |
| PMID | 8014637 |
| Year | 1994 |
| Species | Rat |
| Journal | J Inorg Biochem |
| RefScore | 1 |
| Source | PArchNLP |
  ||


---

|  |  |
| --- | --- |
 Reference:28 || Sentence | "The interaction of dietary Cd and Zn with Cu, Hg, and Ag in relation to tissue metallothionein (MT) was studied with rats." |
| PMID | 7288903 |
| Year | 1981 |
| Species | Rat |
| Journal | J Toxicol Environ Health |
| RefScore | 0 |
| Source | PArchNLP |
  ||


---

|  |  |
| --- | --- |
 Reference:29 || Sentence | "This study was undertaken in order to investigate the effect of zinc (Zn) administration on induction of Zn-binding metallothionein in rat liver with thioacetamide-induced cirrhosis, and the localization of metallothionein in the liver." |
| PMID | 11140829 |
| Year | 2000 |
| Species | Rat |
| Journal | Pharmacol Toxicol |
| RefScore | 0 |
| Source | PArchNLP |
  ||


---

|  |  |
| --- | --- |
 Reference:30 || Sentence | "Metallothioneins are small, cysteine-rich proteins that avidly bind heavy metals such as zinc, copper, and cadmium to reduce their concentration to a physiological or nontoxic level." |
| PMID | 14612393 |
| Year | 2003 |
| Species | Human |
| Journal | Mol Cell Biol |
| RefScore | 0 |
| Source | PArchNLP |
  ||


---

|  |  |
| --- | --- |
 Reference:31 || Sentence | The result showed that liver and kidney induced large amount of metallothioneins that was found to bind to mercury, copper and zinc after mercury intake in stomach and intestines. |
| Year | 2005 |
| PMID | 16395915 |
| Journal | Guang Pu Xue Yu Guang Pu Fen Xi |
| RefScore | 1 |
| Source | PArchNLP |
  ||


---

|  |  |
| --- | --- |
 Reference:32 || Sentence | Indeed (1) in vitro, high IL-6 provokes strong accumulation of MT, impaired cytotoxicity and low zinc ion bioavailability in liver NK1.1(+)TCR gamma/delta(+) cells exclusively from old and MT-I\* mice. (2) The ratio total/endogen PARP-1 activity is higher in very old than in old and MT-I\* mice, suggesting a higher capacity of PARP-1 in base excision DNA-repair in very old age thanks to low zinc-bound MT. |
| Year | 2004 |
| PMID | 15130672 |
| Species | Mouse |
| Journal | Exp Gerontol |
| RefScore | 1 |
| Source | PArchNLP |
  ||


---

|  |  |
| --- | --- |
 Reference:33 || Sentence | Therefore, zinc-bound MT homeostasis is crucial in conferring liver immune plasticity with subsequent successful aging. |
| Year | 2004 |
| PMID | 15247005 |
| Species | Mouse |
| Journal | Ann N Y Acad Sci |
| RefScore | 1 |
| Source | PArchNLP |
  ||


---

|  |  |
| --- | --- |
 Reference:34 || Sentence | The MT sensor chip binds cadmium (Cd), zinc (Zn) or nickel (Ni), but not magnesium (Mg), manganese (Mn) and calcium (Ca). |
| PMID | 15522603 |
| Year | 2004 |
| Journal | Biosens Bioelectron |
| RefScore | 1 |
| Source | PArchNLP |
  ||


---

|  |  |
| --- | --- |
 Reference:35 || Sentence | We have successively studied the influence of the pH value of metmyoglobin solutions (pH 6, 7 and 8) and the influence of the metals nature (Zn,Cu,Cd) bound to metallothioneins. |
| PMID | 15953680 |
| Year | 2005 |
| Journal | Biochim Biophys Acta |
| RefScore | 1 |
| Source | PArchNLP |
  ||


---

|  |  |
| --- | --- |
 Reference:36 || Sentence | One explanation for these observations is that Zn induces the synthesis of metallothionein, which binds Cu for which it has a higher affinity. |
| PMID | 16083871 |
| Year | 2005 |
| Species | Rat |
| Journal | Chem Biol Interact |
| RefScore | 1 |
| Source | PArchNLP |
  ||


---

|  |  |
| --- | --- |
 Reference:37 || Sentence | Zn, Cu, Cd and Hg binding to metallothioneins in harbour porpoises Phocoena phocoena from the southern North Sea. |
| PMID | 16464247 |
| Year | 2006 |
| Journal | BMC Ecol |
| RefScore | 1 |
| Source | PArchNLP |
  ||


---

|  |  |
| --- | --- |
 Reference:38 || Sentence | Concomitant increases in total Zn concentration and Zn bound to MTs were observed in the liver, whereas Zn concentration bound to high molecular weight proteins remained constant. |
| PMID | 16464247 |
| Year | 2006 |
| Journal | BMC Ecol |
| RefScore | 2 |
| Source | PArchNLP |
  ||


---

|  |  |
| --- | --- |
 Reference:39 || Sentence | In addition, a main kidney metallothionein isoform, containing Cd and Zn, was isolated. |
| PMID | 16095669 |
| Year | 2006 |
| Species | Human |
| Journal | Sci Total Environ |
| RefScore | 2 |
| Source | PArchNLP |
  ||


---

|  |  |
| --- | --- |
 Reference:40 || Sentence | In addition, interferonbeta may promote the translocation of metallothionein-bound zinc from cytoplasm to S-phase nuclei. |
| PMID | 16884910 |
| Year | 2006 |
| Species | Human |
| Journal | Cytokine |
| RefScore | 1 |
| Source | PArchNLP |
  ||


---

|  |  |
| --- | --- |
 Reference:41 || Sentence | MT-disulfide can be reduced by glutathione in the presence of selenium catalyst, restoring the capacity of the protein to bind zinc. |
| PMID | 17018867 |
| Year | 2006 |
| Journal | Exp Biol Med (Maywood) |
| RefScore | 2 |
| Source | PArchNLP |
  ||


---

|  |  |
| --- | --- |
 Reference:42 || Sentence | In this study, we investigated the interaction between zinc, MTs, and other components of the antioxidant defense system in HepG2 cells. |
| PMID | 17018880 |
| Year | 2006 |
| Species | Human |
| Journal | Exp Biol Med (Maywood) |
| RefScore | 0 |
| Source | UserNLP |
  ||


---

|  |  |
| --- | --- |
 Reference:43 || Sentence | We previously showed that the major Zn-binding protein, metallothionein (MT) is a critical target for nitric oxide (NO) with resultant increases in labile Zn. |
| PMID | 16423564 |
| Year | 2006 |
| Species | Mouse |
| Journal | Vascul Pharmacol |
| RefScore | 1 |
| Source | UserNLP |
  ||


---

|  |  |
| --- | --- |
 Reference:44 || Sentence | Metallothioneins are small, cysteine-rich proteins that avidly bind heavy metals such as zinc, copper, and cadmium to reduce their concentration to a physiological or nontoxic level. |
| PMID | 14612393 |
| Year | 2003 |
| Species | Human |
| Journal | Mol Cell Biol |
| RefScore | 0 |
| Source | UserNLP |
  |


---

|  |  |
| --- | --- |
