# Supplementary material for: Dynamic transcriptomic profiles of zebrafish gills in response to zinc depletion
Source: BMC Genomics. 2010 Oct 8;11:548. doi: 10.1186/1471-2164-11-548 (PMC3091697; doi:10.1186/1471-2164-11-548)
Supplement: Additional file 2 — Figure S1 - Interactive Direct Interaction Network of responses to zinc depletion. Mini web-site containing index.html and hyperlinked pages in subdirectory. The web site is an interactive version of Figure 6A containing curated interactions between regulated genes and respective proteins. Legend: Molecular interactions between zinc and proteins encoded by genes changed under zinc depletion. A Direct Interaction Network was created based on curated interactions contained within the PathwayArchitect database and provided through hyperlinks. Red ovals represent proteins and the blue circle symbolizes Zn(II). Dark blue squares denote 'binding', and light blue squares 'expression'; green squares stand for 'regulation', green diamonds for 'metabolism', and green circles for 'promoter binding'. Arrow heads indicate directionality of the interaction where annotated. [file 1471-2164-11-548-S2.ZIP › PathwayArchitect Zn def DIN2/411884.html]

# REGULATION:

|  |  |
| --- | --- |
| Type | REGULATION |
| Effect | Positive |


---

|  |  |
| --- | --- |
| Score | 0 |


---

|  |  |
| --- | --- |
| Reference Count | 4 |


---

|  |  |
| --- | --- |
| Mechanism | Unknown |


---

|  |  |
| --- | --- |
| Reference:0 || Sentence | "Since the PACAP-27 effect on TyrOH was abolished when homogenate or pellet of the nucleus accumbens were coincubated with CHAPS, the peptide effect appears to be receptor mediated." |
| PMID | 10657530 |
| Year | 1999 |
| Species | Rat |
| Journal | Neuropeptides |
| RefScore | 1 |
| Source | PArchNLP |
  |
|


---

|  |  |
| --- | --- |
 Reference:1 || Sentence | "This report's data suggest that PACAP-27 activates TyrOH in the rat nucleus accumbens through receptor-mediated cAMP formation." |
| PMID | 10657530 |
| Year | 1999 |
| Species | Rat |
| Journal | Neuropeptides |
| RefScore | 1 |
| Source | PArchNLP |
  ||


---

|  |  |
| --- | --- |
 Reference:2 || Sentence | "In contrast to TaClo, however, 3 and 4 showed biphasic effects after TH activation with PACAP-27, inducing a marked increase of enzyme activity in the nanomolar range (<0.1 microM), while TH activity was nearly completely blocked at high concentrations (IC(100)=0.1mM)." |
| PMID | 11983518 |
| Year | 2002 |
| Species | Rat |
| Journal | Bioorg Med Chem |
| RefScore | 1 |
| Source | PArchNLP |
  ||


---

|  |  |
| --- | --- |
 Reference:3 || Sentence | "When TH was activated by PACAP-27, TaClo, 2-Me-TaClo, or 1-CCl(2) -THbetaC also inhibited activated enzyme activity in high concentrations." |
| PMID | 12065640 |
| Year | 2002 |
| Species | Human |
|  | Rat |
| Journal | J Neurochem |
| RefScore | 0 |
| Source | PArchNLP |
  |


---

|  |  |
| --- | --- |
