# Supplementary material for: Dynamic transcriptomic profiles of zebrafish gills in response to zinc depletion
Source: BMC Genomics. 2010 Oct 8;11:548. doi: 10.1186/1471-2164-11-548 (PMC3091697; doi:10.1186/1471-2164-11-548)
Supplement: Additional file 2 — Figure S1 - Interactive Direct Interaction Network of responses to zinc depletion. Mini web-site containing index.html and hyperlinked pages in subdirectory. The web site is an interactive version of Figure 6A containing curated interactions between regulated genes and respective proteins. Legend: Molecular interactions between zinc and proteins encoded by genes changed under zinc depletion. A Direct Interaction Network was created based on curated interactions contained within the PathwayArchitect database and provided through hyperlinks. Red ovals represent proteins and the blue circle symbolizes Zn(II). Dark blue squares denote 'binding', and light blue squares 'expression'; green squares stand for 'regulation', green diamonds for 'metabolism', and green circles for 'promoter binding'. Arrow heads indicate directionality of the interaction where annotated. [file 1471-2164-11-548-S2.ZIP › PathwayArchitect Zn def DIN2/416456.html]

# REGULATION:

|  |  |
| --- | --- |
| Type | REGULATION |
| Effect | Positive |


---

|  |  |
| --- | --- |
| Score | 0 |


---

|  |  |
| --- | --- |
| Reference Count | 4 |


---

|  |  |
| --- | --- |
| Mechanism | Unknown |


---

|  |  |
| --- | --- |
| Reference:0 || Sentence | "For 2,113 couples, total blood count, Hb A2 and Hb F levels were determined and hemoglobin electrophoresis was performed." |
| PMID | 8666411 |
| Year | 1996 |
| Species | Human |
| Journal | Hum Hered |
| RefScore | 1 |
| Source | PArchNLP |
  |
|


---

|  |  |
| --- | --- |
 Reference:1 || Sentence | "Chromatographic analyses indicated that the Hb F1 zone can be formed both by glycosylation and acetylation of Hb F, and that pre-F1 zones can be products of the reaction of Hb F with phosphorylated glycolytic intermediates." |
| PMID | 427212 |
| Year | 1979 |
| Species | Human |
| Journal | Biochim Biophys Acta |
| RefScore | 2 |
| Source | PArchNLP |
  ||


---

|  |  |
| --- | --- |
 Reference:2 || Sentence | "Fetal hemoglobin (Hb F) is known to interfere with polymerization of Hb S in erythrocytes." |
| PMID | 12673846 |
| Year | 2001 |
| Species | Human |
| Journal | Pediatr Pathol Mol Med |
| RefScore | 2 |
| Source | PArchNLP |
  ||


---

|  |  |
| --- | --- |
 Reference:3 || Sentence | "This population exhibits both elevated and normal levels of Hb A2 associated with high levels of Hb F in homozygotes." |
| PMID | 6206028 |
| Year | 1984 |
| Species | Human |
| Journal | Hemoglobin |
| RefScore | 3 |
| Source | PArchNLP |
  |


---

|  |  |
| --- | --- |
