# Supplementary material for: Dynamic transcriptomic profiles of zebrafish gills in response to zinc depletion
Source: BMC Genomics. 2010 Oct 8;11:548. doi: 10.1186/1471-2164-11-548 (PMC3091697; doi:10.1186/1471-2164-11-548)
Supplement: Additional file 2 — Figure S1 - Interactive Direct Interaction Network of responses to zinc depletion. Mini web-site containing index.html and hyperlinked pages in subdirectory. The web site is an interactive version of Figure 6A containing curated interactions between regulated genes and respective proteins. Legend: Molecular interactions between zinc and proteins encoded by genes changed under zinc depletion. A Direct Interaction Network was created based on curated interactions contained within the PathwayArchitect database and provided through hyperlinks. Red ovals represent proteins and the blue circle symbolizes Zn(II). Dark blue squares denote 'binding', and light blue squares 'expression'; green squares stand for 'regulation', green diamonds for 'metabolism', and green circles for 'promoter binding'. Arrow heads indicate directionality of the interaction where annotated. [file 1471-2164-11-548-S2.ZIP › PathwayArchitect Zn def DIN2/422187.html]

# REGULATION:

|  |  |
| --- | --- |
| Type | REGULATION |
| Effect | None |


---

|  |  |
| --- | --- |
| Score | 0 |


---

|  |  |
| --- | --- |
| Reference Count | 4 |


---

|  |  |
| --- | --- |
| Mechanism | Unknown |


---

|  |  |
| --- | --- |
| Reference:0 || Sentence | "In protein/DNA binding reactions in vitro, the purified human MTF-1 was activated by zinc but not by other metallothionein-inducing heavy metals, supporting the idea that zinc is the direct modulator of human MTF-1." |
| PMID | 10092847 |
| Year | 1999 |
| Species | Human |
| Journal | Eur J Biochem |
| RefScore | 1 |
| Source | PArchNLP |
  |
|


---

|  |  |
| --- | --- |
 Reference:1 || Sentence | "Recombinant mouse and human MTF-1 were also dependent on exogenous zinc for DNA binding activity." |
| PMID | 9507026 |
| Year | 1998 |
| Species | Mouse |
|  | Human |
| Journal | J Biol Chem |
| RefScore | 0 |
| Source | PArchNLP |
  ||


---

|  |  |
| --- | --- |
 Reference:2 || Sentence | "Manipulating the Zn concentration in the low microM range reversibly modulates the DNA-binding activity of the mammalian MTF-1; this effect is inhibited at low temperature." |
| PMID | 11007174 |
| Year | 2000 |
| Species | Mouse |
| Journal | Comp Biochem Physiol B Biochem Mol Biol |
| RefScore | 1 |
| Source | PArchNLP |
  ||


---

|  |  |
| --- | --- |
 Reference:3 || Sentence | Detailed spectral density and hydrodynamic analysis of (15)N relaxation data revealed quasi-ordered anisotropic rotational diffusion properties of the six F1-6 zinc fingers that could influence MTF-1 DNA binding function. |
| PMID | 16055450 |
| Year | 2005 |
| Species | Mouse |
| Journal | J Biol Chem |
| RefScore | 1 |
| Source | PArchNLP |
  |


---

|  |  |
| --- | --- |
