# Supplementary material for: Dynamic transcriptomic profiles of zebrafish gills in response to zinc depletion
Source: BMC Genomics. 2010 Oct 8;11:548. doi: 10.1186/1471-2164-11-548 (PMC3091697; doi:10.1186/1471-2164-11-548)
Supplement: Additional file 2 — Figure S1 - Interactive Direct Interaction Network of responses to zinc depletion. Mini web-site containing index.html and hyperlinked pages in subdirectory. The web site is an interactive version of Figure 6A containing curated interactions between regulated genes and respective proteins. Legend: Molecular interactions between zinc and proteins encoded by genes changed under zinc depletion. A Direct Interaction Network was created based on curated interactions contained within the PathwayArchitect database and provided through hyperlinks. Red ovals represent proteins and the blue circle symbolizes Zn(II). Dark blue squares denote 'binding', and light blue squares 'expression'; green squares stand for 'regulation', green diamonds for 'metabolism', and green circles for 'promoter binding'. Arrow heads indicate directionality of the interaction where annotated. [file 1471-2164-11-548-S2.ZIP › PathwayArchitect Zn def DIN2/432096.html]

# REGULATION:

|  |  |
| --- | --- |
| Type | REGULATION |
| Effect | Positive |


---

|  |  |
| --- | --- |
| Score | 0 |


---

|  |  |
| --- | --- |
| Reference Count | 105 |


---

|  |  |
| --- | --- |
| Mechanism | Unknown |


---

|  |  |
| --- | --- |
| Reference:0 || Sentence | "It is well known that Zn induces MT and has the ability to prevent redox-active metals, Cu and Fe, binding to and causing oxidative damage at active sites of Zn metalloenzymes and nonspecific binding sites on proteins." |
| PMID | 12242608 |
| Year | 2002 |
| Species | Rat |
| Journal | Arch Toxicol |
| RefScore | 2 |
| Source | PArchNLP |
  |
|


---

|  |  |
| --- | --- |
 Reference:1 || Sentence | "We have used the monocytic cell line THP-1 as a model in which to study both the responsiveness of metallothionein and ZIP2 transporter expression to zinc depletion induced by the intracellular zinc chelator TPEN [N,N,N',N'-tetrakis(2-pyridylmethyl) ethylenediamine] and the extent of concomitant apoptosis." |
| PMID | 11590192 |
| Year | 2001 |
| Species | Human |
| Journal | J Leukoc Biol |
| RefScore | 0 |
| Source | PArchNLP |
  ||


---

|  |  |
| --- | --- |
 Reference:2 || Sentence | "To examine their interaction with known MT inducers, cadmium (Cd), zinc (Zn), or dexamethasone (Dex) were added to some cultures." |
| PMID | 1597404 |
| Year | 1992 |
| Species | Rat |
| Journal | In Vitro Cell Dev Biol |
| RefScore | 0 |
| Source | PArchNLP |
  ||


---

|  |  |
| --- | --- |
 Reference:3 || Sentence | "Pretreatment with Zn(OAc)2, a potent inducer of renal and hepatic MT, reduced the nephrotoxicity of HgCl2, but did not alter HgCl2-mediated renal EH increases in either strain." |
| PMID | 3338102 |
| Year | 1988 |
| Species | Rat |
| Journal | Carcinogenesis |
| RefScore | 0 |
| Source | PArchNLP |
  ||


---

|  |  |
| --- | --- |
 Reference:4 || Sentence | "Endotoxin reduced serum zinc levels while elevating zinc associated with hepatic metallothionein (Zn-MT) in control, fasted, and zinc-depleted rats." |
| PMID | 6388345 |
| Year | 1984 |
| Species | Rat |
| Journal | Am J Physiol |
| RefScore | 1 |
| Source | PArchNLP |
  ||


---

|  |  |
| --- | --- |
 Reference:5 || Sentence | "This investigation was designed to assess the effects of subadequate dietary zinc intake on the accumulation of dietary cadmium and on metallothionein (MT) and zinc concentrations in target organs of cadmium toxicity." |
| PMID | 3712492 |
| Year | 1986 |
| Species | Rat |
| Journal | J Toxicol Environ Health |
| RefScore | 1 |
| Source | PArchNLP |
  ||


---

|  |  |
| --- | --- |
 Reference:6 || Sentence | "Zinc may have an antioxidant effect mediated by induction of metallothionein." |
| PMID | 10575193 |
| Year | 1999 |
| Species | Rat |
| Journal | Am J Nephrol |
| RefScore | 1 |
| Source | PArchNLP |
  ||


---

|  |  |
| --- | --- |
 Reference:7 || Sentence | "These findings suggest that zinc has an antioxidant effect mediated through the induction of metallothionein, but appears only to have a minor protective effect on renal function induced by renal ischemia-reperfusion injury." |
| PMID | 10575193 |
| Year | 1999 |
| Species | Rat |
| Journal | Am J Nephrol |
| RefScore | 0 |
| Source | PArchNLP |
  ||


---

|  |  |
| --- | --- |
 Reference:8 || Sentence | "In addition, adrenalectomy only partially inhibited the ability of IL-1 to depress serum zinc levels and increase the amount of zinc associated with hepatic metallothionein." |
| PMID | 6333572 |
| Year | 1984 |
| Species | Rat |
| Journal | Life Sci |
| RefScore | 2 |
| Source | PArchNLP |
  ||


---

|  |  |
| --- | --- |
 Reference:9 || Sentence | "Zinc induced the hepatic levels of metallothionein but [14C]BB did not bind to this sulfhydryl rich protein." |
| PMID | 3988000 |
| Year | 1985 |
| Species | Rat |
| Journal | Fundam Appl Toxicol |
| RefScore | 0 |
| Source | PArchNLP |
  ||


---

|  |  |
| --- | --- |
 Reference:10 || Sentence | "Therefore, Zn pretreatment, possibly by providing elevated MT protein levels at the point of Cd exposure, inhibited the Cd-induced c-myc and c-jun proto-oncogene expression." |
| PMID | 8691507 |
| Year | 1996 |
| Species | Rat |
| Journal | J Toxicol Environ Health |
| RefScore | 1 |
| Source | PArchNLP |
  ||


---

|  |  |
| --- | --- |
 Reference:11 || Sentence | "The Zn-pretreatment significantly increased the binding of Zn and Cu to both the renal and hepatic metallothioneins (MT), but the proportion of cytosolic Pt associated with MT in the kidney tissue was slightly reduced, and that in the liver showed no difference in comparison to rats treated with cis-DDP only." |
| PMID | 6539966 |
| Year | 1984 |
| Species | Rat |
| Journal | Toxicology |
| RefScore | 0 |
| Source | PArchNLP |
  ||


---

|  |  |
| --- | --- |
 Reference:12 || Sentence | "Metallothionein in the translation products was identified on the basis of high cysteine and serine incorporation and absence of leucine incorporation as well as comigration with authentic zinc-induced rat-liver metallothionein on SDS-polyacrylamide gels." |
| PMID | 6796153 |
| Year | 1981 |
| Species | Rat |
| Journal | Biosci Rep |
| RefScore | 1 |
| Source | PArchNLP |
  ||


---

|  |  |
| --- | --- |
 Reference:13 || Sentence | "Gel filtration showed that both Cu and Zn were associated with the plasma MT of pregnant rats." |
| PMID | 8456105 |
| Year | 1993 |
| Species | Rat |
| Journal | Proc Soc Exp Biol Med |
| RefScore | 2 |
| Source | PArchNLP |
  ||


---

|  |  |
| --- | --- |
 Reference:14 || Sentence | "These results indicate that supplementation of cystine to the diet can induce kidney metallothionein through a mechanism involving altered zinc metabolism." |
| PMID | 7738676 |
| Year | 1995 |
| Species | Rat |
| Journal | J Nutr |
| RefScore | 0 |
| Source | PArchNLP |
  ||


---

|  |  |
| --- | --- |
 Reference:15 || Sentence | "Radioactive zinc (65Zn) included in the diet rapidly associated with newly formed metallothionein." |
| PMID | 7223884 |
| Year | 1981 |
| Species | Rat |
| Journal | Am J Physiol |
| RefScore | 1 |
| Source | PArchNLP |
  ||


---

|  |  |
| --- | --- |
 Reference:16 || Sentence | "Unlike Zn2+, 65Zn exhibited marked fluctuations within the 24-h period following feeding, indicating that zinc associated with metallothionein may be capable of exchange and/or dissociation." |
| PMID | 7223884 |
| Year | 1981 |
| Species | Rat |
| Journal | Am J Physiol |
| RefScore | 2 |
| Source | PArchNLP |
  ||


---

|  |  |
| --- | --- |
 Reference:17 || Sentence | "The protective effects of ZnCl2 may be due to lower iron uptake into hepatocytes and by the induction of zinc metallothionein, which can serve as a scavenger for oxygen radicals." |
| PMID | 1917562 |
| Year | 1991 |
| Species | Rat |
| Journal | Histochem J |
| RefScore | 0 |
| Source | PArchNLP |
  ||


---

|  |  |
| --- | --- |
 Reference:18 || Sentence | "Furthermore, purification of the zinc-induced metallothionein by ion exchange chromatography on DEAE-Sephadex A-25 columns produced two isoforms, eluting, respectively, at 68 and 130 mM of Tris-acetate buffer, pH 7.5." |
| PMID | 2710279 |
| Year | 1989 |
| Species | Rat |
| Journal | Neurochem Res |
| RefScore | 1 |
| Source | PArchNLP |
  ||


---

|  |  |
| --- | --- |
 Reference:19 || Sentence | "In this paper, we report that zinc-induced metallothionein produces also two distinct isoforms on reverse phase high performance liquid chromatography that exhibit retention times of 17.23 and 18.53 minutes, respectively." |
| PMID | 2710279 |
| Year | 1989 |
| Species | Rat |
| Journal | Neurochem Res |
| RefScore | 0 |
| Source | PArchNLP |
  ||


---

|  |  |
| --- | --- |
 Reference:20 || Sentence | "Using zinc, a metallothionein inducer, we observed a slight increase in the growth rate of some transfectants, which can be measured by thymidine incorporation." |
| PMID | 3743660 |
| Year | 1986 |
| Species | Human |
|  | Mouse |
|  | Rat |
| Journal | Exp Cell Res |
| RefScore | 0 |
| Source | PArchNLP |
  ||


---

|  |  |
| --- | --- |
 Reference:21 || Sentence | "Zinc administration resulted in an elevation of metallothionein mRNA activity to 11% of the total polyribosomal mRNA activity." |
| PMID | 7470077 |
| Year | 1980 |
| Species | Rat |
| Journal | Biochem J |
| RefScore | 0 |
| Source | PArchNLP |
  ||


---

|  |  |
| --- | --- |
 Reference:22 || Sentence | "These data indicate that the induction of metallothionein mRNA by zinc involves only free polyribosomes and suggest that the function of metallothionein is limited to intracellular processes." |
| PMID | 7470077 |
| Year | 1980 |
| Species | Rat |
| Journal | Biochem J |
| RefScore | 1 |
| Source | PArchNLP |
  ||


---

|  |  |
| --- | --- |
 Reference:23 || Sentence | "Similarly, metals such as cadmium and zinc, will increase the levels of metallothionein (MT)." |
| PMID | 8365580 |
| Year | 1993 |
| Species | Rat |
| Journal | Fundam Appl Toxicol |
| RefScore | 1 |
| Source | PArchNLP |
  ||


---

|  |  |
| --- | --- |
 Reference:24 || Sentence | "Zn, stress and endotoxin increased liver MT levels significantly, by 12-, 5- and 8-fold, respectively." |
| PMID | 8599853 |
| Year | 1996 |
| Species | Rat |
| Journal | Chem Biol Interact |
| RefScore | 1 |
| Source | PArchNLP |
  ||


---

|  |  |
| --- | --- |
 Reference:25 || Sentence | "Treatment with 20 mg/kg Zn prior to DNR, dramatically induced metallothionein-1 (MT-1) mRNA and MT protein in both heart and liver while DNR alone induced MT, but to a much lower degree than Zn." |
| PMID | 12204545 |
| Year | 2002 |
| Species | Rat |
| Journal | Toxicology |
| RefScore | 2 |
| Source | PArchNLP |
  ||


---

|  |  |
| --- | --- |
 Reference:26 || Sentence | "Our results indicate that MT induction by Zn is a highly effective approach in preventing cardiotoxicity and hepatotoxicity caused by DNR." |
| PMID | 12204545 |
| Year | 2002 |
| Species | Rat |
| Journal | Toxicology |
| RefScore | 0 |
| Source | PArchNLP |
  ||


---

|  |  |
| --- | --- |
 Reference:27 || Sentence | "Trientine gives a strong, fast, negative copper balance, and zinc induces hepatic metallothionein, which sequesters hepatic copper." |
| PMID | 8521757 |
| Year | 1995 |
| Species | Human |
| Journal | Drugs |
| RefScore | 1 |
| Source | PArchNLP |
  ||


---

|  |  |
| --- | --- |
 Reference:28 || Sentence | "OBJECTIVE: Wilson's disease is effectively treated by zinc administration which, in vitro, increases metallothionein concentrations." |
| PMID | 10022625 |
| Year | 1999 |
| Species | Human |
| Journal | Am J Gastroenterol |
| RefScore | 2 |
| Source | PArchNLP |
  ||


---

|  |  |
| --- | --- |
 Reference:29 || Sentence | "Although the same level of MT-2 as that of MT-1 was induced by zinc, the level of MT-2 was 3 to 4 fold higher than that of MT-1 by administrations of endotoxin or glucocorticoid hormone." |
| PMID | 2822467 |
| Year | 1987 |
| Species | Rat |
|  | Human |
| Journal | Experientia Suppl |
| RefScore | 2 |
| Source | PArchNLP |
  ||


---

|  |  |
| --- | --- |
 Reference:30 || Sentence | "Injection of zinc or copper intracerebroventricularly and the use of a zinc-deficient diet increased and decreased MT levels, respectively, in some but not all brain areas." |
| PMID | 8203514 |
| Year | 1994 |
| Species | Human |
|  | Rat |
| Journal | Am J Physiol |
| RefScore | 1 |
| Source | PArchNLP |
  ||


---

|  |  |
| --- | --- |
 Reference:31 || Sentence | "Zinc induced the transferred MT promoter activity by approximately 2-fold or 10-fold when administered systemically and topically, respectively." |
| PMID | 11678883 |
| Year | 2001 |
| Species | Human |
|  | Rat |
| Journal | Clin Exp Dermatol |
| RefScore | 1 |
| Source | PArchNLP |
  ||


---

|  |  |
| --- | --- |
 Reference:32 || Sentence | "In the exocrine cells of the pancreas, MT was induced by various stresses such as zinc, STZ, alloxan and 4-aminopyrazolo-(3,4-d) pyrimidine, but the effects of those stresses were not clear in the endocrine cells." |
| PMID | 10723270 |
| Year | 2000 |
| Species | Human |
|  | Rat |
| Journal | Yakugaku Zasshi |
| RefScore | 0 |
| Source | PArchNLP |
  ||


---

|  |  |
| --- | --- |
 Reference:33 || Sentence | "Daily supplementation of control subjects with zinc (50 mg/day) increased erythrocyte metallothionein to a 7-fold maximum within 7 days." |
| PMID | 2304897 |
| Year | 1990 |
| Species | Rat |
|  | Human |
| Journal | Proc Natl Acad Sci U S A |
| RefScore | 0 |
| Source | PArchNLP |
  ||


---

|  |  |
| --- | --- |
 Reference:34 || Sentence | "Induction of marrow metallothionein by zinc in nonanemic rats required prior treatment with erythropoietin." |
| PMID | 8498498 |
| Year | 1993 |
| Species | Human |
|  | Rat |
| Journal | Am J Physiol |
| RefScore | 1 |
| Source | PArchNLP |
  ||


---

|  |  |
| --- | --- |
 Reference:35 || Sentence | "Experiments with primary cultures enriched in neurons or astrocytes indicate that MT is present in both cell types and that responds to the well-known MT inducers zinc, copper and glucocorticoids." |
| PMID | 7834015 |
| Year | 1994 |
| Species | Human |
|  | Rat |
| Journal | Biol Signals |
| RefScore | 1 |
| Source | PArchNLP |
  ||


---

|  |  |
| --- | --- |
 Reference:36 || Sentence | "Cells were treated with Zn to induce MT and then treated with six RA concentrations." |
| PMID | 9400029 |
| Year | 1997 |
| Species | Human |
| Journal | J Pharmacol Exp Ther |
| RefScore | 1 |
| Source | PArchNLP |
  ||


---

|  |  |
| --- | --- |
 Reference:37 || Sentence | "MT was significantly increased by Zn treatment in BT-20 cells but not in MCF7 cells." |
| PMID | 9400029 |
| Year | 1997 |
| Species | Human |
| Journal | J Pharmacol Exp Ther |
| RefScore | 0 |
| Source | PArchNLP |
  ||


---

|  |  |
| --- | --- |
 Reference:38 || Sentence | "The results presented here show that both monocyte metallothionein mRNA and erythrocyte metallothionein protein concentrations change in human subjects in response to elevated dietary zinc intake and that monocyte metallothionein mRNA responds more rapidly to elevation of dietary zinc status than erythrocyte metallothionein protein." |
| PMID | 9521632 |
| Year | 1998 |
| Species | Human |
| Journal | J Nutr |
| RefScore | 3 |
| Source | PArchNLP |
  ||


---

|  |  |
| --- | --- |
 Reference:39 || Sentence | "On the basis of animal studies we postulate that this sequestered copper is primarily bound to the high levels of hepatic metallothionein induced by zinc." |
| PMID | 2592853 |
| Year | 1989 |
| Species | Human |
| Journal | J Lab Clin Med |
| RefScore | 1 |
| Source | PArchNLP |
  ||


---

|  |  |
| --- | --- |
 Reference:40 || Sentence | "The results show that pretreatment of tumor-bearing mice with zinc salts increased MT content, both in normal and tumor tissues, with a marked reduction in the antitumor activity of cisplatin, Adriamycin, and melphalan." |
| PMID | 7923149 |
| Year | 1994 |
| Species | Human |
|  | Mouse |
| Journal | Cancer Res |
| RefScore | 1 |
| Source | PArchNLP |
  ||


---

|  |  |
| --- | --- |
 Reference:41 || Sentence | "Injection of propargylglycine, an inhibitor of cystathionase, decreased MT induction by zinc in the tumor and diminished the resistance to these drugs." |
| PMID | 7923149 |
| Year | 1994 |
| Species | Human |
|  | Mouse |
| Journal | Cancer Res |
| RefScore | 0 |
| Source | PArchNLP |
  ||


---

|  |  |
| --- | --- |
 Reference:42 || Sentence | "The cadmium exposure increased hepatic and renal zinc and renal copper levels, probably as a result of cadmium-induced MT, and some of the levels were normalized considerably by the subsequent treatment with cysteine, DMPS or to a lesser extent N-acetyl cysteine and their combinations, showing their protective effects against cadmium toxicity." |
| PMID | 11807931 |
| Year | 2002 |
| Species | Rat |
| Journal | J Appl Toxicol |
| RefScore | 0 |
| Source | PArchNLP |
  ||


---

|  |  |
| --- | --- |
 Reference:43 || Sentence | "An increased body retention of Cd in rats orally pretreated with Cd or Zn is explained by induction of hepatic and renal metallothionein." |
| PMID | 12504351 |
| Year | 2002 |
| Species | Rat |
| Journal | Toxicology |
| RefScore | 2 |
| Source | PArchNLP |
  ||


---

|  |  |
| --- | --- |
 Reference:44 || Sentence | "The zinc-stimulated protein incorporates 35S cysteine 24-fold higher than the native, unstimulated protein; is blocked by actinomycin D; produces two isoforms by ion exchange chromatography on DEAE Sephadex A 25 columns; and by high performance liquid chromatography, depicts a similar but not identical profile to zinc-stimulated hepatic metallothionein." |
| PMID | 3788711 |
| Year | 1986 |
| Species | Human |
| Journal | Adv Exp Med Biol |
| RefScore | 3 |
| Source | PArchNLP |
  ||


---

|  |  |
| --- | --- |
 Reference:45 || Sentence | "Cadmium, Zn, and Cd-induced MT concentrations in the liver and kidney were lower in the Zn-deficient rats (-Zn + Cd) than in the Zn-adequate rats (+Zn + Cd), while the content of Cd bound to high molecular weight proteins (HMWP) was greater in the Zn-deficient rats (-Zn + Cd)." |
| PMID | 2774670 |
| Year | 1989 |
| Species | Rat |
| Journal | Arch Environ Contam Toxicol |
| RefScore | 1 |
| Source | PArchNLP |
  ||


---

|  |  |
| --- | --- |
 Reference:46 || Sentence | "Additional experiments indicated that Cd (1-30 mumol/kg), Zn (100-3000 mumol/kg), and dexamethasone (0.3-10 mumol/kg) increased hepatic concentrations of MT-I and MT-II and their respective mRNAs in 14-day-old rats, despite the preexisting high levels of protein and mRNA at this time." |
| PMID | 3341019 |
| Year | 1988 |
| Species | Rat |
| Journal | Toxicol Appl Pharmacol |
| RefScore | 0 |
| Source | PArchNLP |
  ||


---

|  |  |
| --- | --- |
 Reference:47 || Sentence | "Zn pretreatment increased the hepatic MT concentrations markedly and reduced the magnitudes of the CCl4-induced reduction of cytochrome P450 concentration as well as elevation of serum alanine aminotransferase and aspartate aminotransferase activities when determined at 4 or 24 h following CCl4 treatment." |
| PMID | 3791046 |
| Year | 1986 |
| Species | Rat |
| Journal | Can J Physiol Pharmacol |
| RefScore | 1 |
| Source | PArchNLP |
  ||


---

|  |  |
| --- | --- |
 Reference:48 || Sentence | "Since MT is a zinc binding protein and certain inducers of MT including zinc play a role in apoptosis, studies were undertaken to compare the expression of MT and the presence of apoptotic cells (APPC) in both primary HCC and metastatic adenocarcinoma." |
| PMID | 9602331 |
| Year | 1998 |
| Species | Human |
| Journal | Histopathology |
| RefScore | 3 |
| Source | PArchNLP |
  ||


---

|  |  |
| --- | --- |
 Reference:49 || Sentence | "In JIB rats, however, liver copper, MT protein, and MT mRNA were significantly elevated, and a high proportion of the intracellular zinc and copper was associated with MT." |
| PMID | 11090006 |
| Year | 2000 |
| Species | Rat |
| Journal | Am Surg |
| RefScore | 2 |
| Source | PArchNLP |
  ||


---

|  |  |
| --- | --- |
 Reference:50 || Sentence | "In the present study, the effects of oral Cd exposure (0, 50, 100, 200 ppm in drinking water) on serum sex hormone levels, the expression of MT-I and MT-II mRNA, and the zinc content of rat prostate were assessed." |
| PMID | 12604174 |
| Year | 2003 |
| Species | Rat |
| Journal | Toxicology |
| RefScore | 0 |
| Source | PArchNLP |
  ||


---

|  |  |
| --- | --- |
 Reference:51 || Sentence | "Prior induction of renal MT by treatment with zinc (20 mg of Zn per kg as ZnSO4, i.p. 16 hr before CdMT injection) markedly reduced non-MT binding of Cd++ in kidneys of treated animals and inhibited the alterations in urine volume and low molecular weight protein reabsorption induced by CdMT." |
| PMID | 6707945 |
| Year | 1984 |
| Species | Rat |
| Journal | J Pharmacol Exp Ther |
| RefScore | 0 |
| Source | PArchNLP |
  ||


---

|  |  |
| --- | --- |
 Reference:52 || Sentence | "Pre-induction of metallothionein in hepatocytes by zinc treatment in vivo of donor rats protected hepatocytes in vitro from cadmium-induced inhibition of protein synthesis." |
| PMID | 4052053 |
| Year | 1985 |
| Species | Rat |
| Journal | Biochem J |
| RefScore | 0 |
| Source | PArchNLP |
  ||


---

|  |  |
| --- | --- |
 Reference:53 || Sentence | "Moreover, it was demonstrated that CCl4, after metabolic activation, reduced the cadmium binding capacity of Zn-induced hepatic MT in vitro." |
| PMID | 3791046 |
| Year | 1986 |
| Species | Rat |
| Journal | Can J Physiol Pharmacol |
| RefScore | 0 |
| Source | PArchNLP |
  ||


---

|  |  |
| --- | --- |
 Reference:54 || Sentence | "Incubation with zinc, which induces metallothionein, results in an increase in 67Cu associated with the nonextractable pool, suggesting that 67Cu-metallothionein constitutes at least part of the nonextractable pool." |
| PMID | 9227475 |
| Year | 1997 |
| Species | Rat |
| Journal | Am J Physiol |
| RefScore | 2 |
| Source | PArchNLP |
  ||


---

|  |  |
| --- | --- |
 Reference:55 || Sentence | "HPLC fractionation of samples from maternal livers showed a higher percentage of Zn, Cu and 65Zn associated with the metallothionein peak in samples from untreated diabetic dams compared with other groups, whereas the percentages of Zn, Cu and 65Zn per fraction in fetal liver were similar." |
| PMID | 1527640 |
| Year | 1992 |
| Species | Rat |
| Journal | J Nutr |
| RefScore | 1 |
| Source | PArchNLP |
  ||


---

|  |  |
| --- | --- |
 Reference:56 || Sentence | "Compared to controls, liver and kidneys of diabetic dams showed an increased concentration of zinc and copper that was associated with metallothionein." |
| PMID | 2484570 |
| Year | 1988 |
| Species | Rat |
| Journal | Biol Trace Elem Res |
| RefScore | 2 |
| Source | PArchNLP |
  ||


---

|  |  |
| --- | --- |
 Reference:57 || Sentence | "Conversely, mercury only partially replaced the zinc associated with metallothionein, and a bolus of zinc was completely unable to bind to the already zinc-saturated metallothionein." |
| PMID | 2778848 |
| Year | 1989 |
| Species | Rat |
| Journal | J Toxicol Environ Health |
| RefScore | 2 |
| Source | PArchNLP |
  ||


---

|  |  |
| --- | --- |
 Reference:58 || Sentence | "The results suggest renal metallothionein is induced by zinc or cadmium through a mechanism that requires altered expression of the metallothionein gene(s)." |
| PMID | 7069515 |
| Year | 1982 |
| Species | Rat |
| Journal | J Nutr |
| RefScore | 2 |
| Source | PArchNLP |
  ||


---

|  |  |
| --- | --- |
 Reference:59 || Sentence | "The ATP data suggest significant but comparable Zn effects on cellular metabolism in both cell types, notwithstanding the large differences in cellular Zn, MT, and GSH levels." |
| PMID | 8442003 |
| Year | 1993 |
| Species | Rat |
| Journal | Toxicol Appl Pharmacol |
| RefScore | 1 |
| Source | PArchNLP |
  ||


---

|  |  |
| --- | --- |
 Reference:60 || Sentence | "Addition of Cu(II) to Zn-MT (more than 6 mol Cu/mol MT) in non-reducing conditions modifies the response of the antibody, probably because of Cu(II) oxidation and later MT polymerization." |
| PMID | 8294566 |
| Year | 1993 |
| Species | Rat |
| Journal | J Immunoassay |
| RefScore | 1 |
| Source | PArchNLP |
  ||


---

|  |  |
| --- | --- |
 Reference:61 || Sentence | "In this study, a two-by-six factorial design was used to investigate the interactive effects of all-trans-RA and zinc (Zn)-induced MT on the growth of two human breast cancer cell lines differing in basal expression of MT and estrogen receptors; MCF7 cells express estrogen receptor, BT-20 cells do not." |
| PMID | 9400029 |
| Year | 1997 |
| Species | Human |
| Journal | J Pharmacol Exp Ther |
| RefScore | 2 |
| Source | PArchNLP |
  ||


---

|  |  |
| --- | --- |
 Reference:62 || Sentence | "Plasma Zn decreased after AJ injection (60% of control values at 8 h), and this was associated with a 4.5-fold increase in hepatic MT at 8 h." |
| PMID | 1384614 |
| Year | 1992 |
| Species | Rat |
| Journal | Biol Trace Elem Res |
| RefScore | 2 |
| Source | PArchNLP |
  ||


---

|  |  |
| --- | --- |
 Reference:63 || Sentence | "In an effort to delineate the regulation of the synthesis of the recently identified brain metallothionein-like protein, a study was undertaken to compare the induction of metallothionein in human neuroblastoma IMR-32 cells by zinc, cadmium, and dexamethasone using the human Chang liver cells as a control." |
| PMID | 2484408 |
| Year | 1989 |
| Species | Rat |
| Journal | Biol Trace Elem Res |
| RefScore | 1 |
| Source | PArchNLP |
  ||


---

|  |  |
| --- | --- |
 Reference:64 || Sentence | "Among milk proteins, zinc was associated with casein, albumin, lactoferrin, and metallothionein, whereas among LMW substances a zinc peak could be observed exclusively with citrate." |
| PMID | 1822333 |
| Year | 1991 |
| Species | Human |
| Journal | J Trace Elem Electrolytes Health Dis |
| RefScore | 2 |
| Source | PArchNLP |
  ||


---

|  |  |
| --- | --- |
 Reference:65 || Sentence | "In contrast, the levels of zinc-induced MT mRNAs gradually declined after about 4 h, despite substantial transcription." |
| PMID | 2555705 |
| Year | 1989 |
| Species | Human |
| Journal | Mol Cell Biol |
| RefScore | 1 |
| Source | PArchNLP |
  ||


---

|  |  |
| --- | --- |
 Reference:66 || Sentence | "The results indicate that zinc not only induces metallothionein, but also increases protective enzyme activities and glutathione content, which would tend to inhibit lipid peroxidation and suppress mercury toxicity." |
| PMID | 6232736 |
| Year | 1984 |
| Species | Rat |
| Journal | Toxicol Appl Pharmacol |
| RefScore | 1 |
| Source | PArchNLP |
  ||


---

|  |  |
| --- | --- |
 Reference:67 || Sentence | "Presence of zinc had no effect on MT levels nor on total Cu and 64Cu levels, in contrast with cadmium which drastically enhanced copper accumulation and MT levels in the cells." |
| PMID | 7940570 |
| Year | 1994 |
| Species | Rat |
| Journal | Toxicology |
| RefScore | 2 |
| Source | PArchNLP |
  ||


---

|  |  |
| --- | --- |
 Reference:68 || Sentence | "Zinc supplementation was associated with increased concentrations of hepatic metallothioneins together with decreased concentrations of proline-hydroxylase and collagen but to a lesser degree than in alcoholic animals." |
| PMID | 7657481 |
| Year | 1995 |
| Species | Rat |
| Journal | Int J Vitam Nutr Res |
| RefScore | 2 |
| Source | PArchNLP |
  ||


---

|  |  |
| --- | --- |
 Reference:69 || Sentence | "Administration of gonadoliberin agonist (gonadoliberin-A) inhibited the Zn-induced metallothionein mRNA level in a time-related and dose-related manner." |
| PMID | 8954150 |
| Year | 1996 |
| Species | Human |
| Journal | Eur J Biochem |
| RefScore | 1 |
| Source | PArchNLP |
  ||


---

|  |  |
| --- | --- |
 Reference:70 || Sentence | "The contents of zinc, copper and cadmium, which are primary inducers of MT, in the incubation medium of macrophages in the presence of endotoxin were not different from those in the absence of endotoxin." |
| PMID | 3494383 |
| Year | 1987 |
| Species | Rat |
| Journal | Acta Med Okayama |
| RefScore | 0 |
| Source | PArchNLP |
  ||


---

|  |  |
| --- | --- |
 Reference:71 || Sentence | "Zinc markedly increased liver metallothionein levels whereas 13-cis-retinoic acid was a much less potent inducer of the protein in liver." |
| PMID | 3857634 |
| Year | 1985 |
| Species | Rat |
| Journal | Proc Soc Exp Biol Med |
| RefScore | 1 |
| Source | PArchNLP |
  ||


---

|  |  |
| --- | --- |
 Reference:72 || Sentence | "Zn concentration was increased by metallothionein induction." |
| PMID | 12498321 |
| Year | 2002 |
| Species | Mouse |
|  | Human |
| Journal | Tohoku J Exp Med |
| RefScore | 2 |
| Source | PArchNLP |
  ||


---

|  |  |
| --- | --- |
 Reference:73 || Sentence | "Intestinal metallothionein mRNA and metallothionein protein were not affected by dexamethasone or interleukin 1 alpha, but were markedly increased by parenteral zinc." |
| PMID | 1880617 |
| Year | 1991 |
| Species | Rat |
| Journal | J Nutr |
| RefScore | 1 |
| Source | PArchNLP |
  ||


---

|  |  |
| --- | --- |
 Reference:74 || Sentence | "Reintroducing zinc at concentrations of 16 or 48 mumols Zn/L to hepatocytes after the initial 3 h of culture in basal medium significantly increased cell zinc, MT and MTmRNA levels and fully restored delta-ALA-D activity by 24 h." |
| PMID | 2033469 |
| Year | 1991 |
| Species | Rat |
| Journal | J Nutr |
| RefScore | 1 |
| Source | PArchNLP |
  ||


---

|  |  |
| --- | --- |
 Reference:75 || Sentence | "Treatment with Zn salts increased MT concentrations in both liver and pancreas." |
| PMID | 2219126 |
| Year | 1990 |
| Species | Rat |
| Journal | Toxicology |
| RefScore | 0 |
| Source | PArchNLP |
  ||


---

|  |  |
| --- | --- |
 Reference:76 || Sentence | "In kidney, although Cd or Zn treatment separately had no effect on MT or MT-1 mRNA content, injection of Cd followed by Zn resulted in significantly increased levels of renal MT and MT-1 mRNA." |
| PMID | 2219126 |
| Year | 1990 |
| Species | Rat |
| Journal | Toxicology |
| RefScore | 2 |
| Source | PArchNLP |
  ||


---

|  |  |
| --- | --- |
 Reference:77 || Sentence | "Zn increased MT in every organ examined except brain." |
| PMID | 4007305 |
| Year | 1985 |
| Species | Rat |
| Journal | Fundam Appl Toxicol |
| RefScore | 0 |
| Source | PArchNLP |
  ||


---

|  |  |
| --- | --- |
 Reference:78 || Sentence | "There were also 2- and 3-fold increases of urinary metallothionein by Cu and Zn treatments for 5 days, respectively." |
| PMID | 6623481 |
| Year | 1983 |
| Species | Rat |
| Journal | Toxicology |
| RefScore | 2 |
| Source | PArchNLP |
  ||


---

|  |  |
| --- | --- |
 Reference:79 || Sentence | "Thus, urinary metallothionein levels were elevated in response to Cd, Hg, Cu and Zn, but not Pb; Hg had the most profound effect at equimolar doses." |
| PMID | 6623481 |
| Year | 1983 |
| Species | Rat |
| Journal | Toxicology |
| RefScore | 1 |
| Source | PArchNLP |
  ||


---

|  |  |
| --- | --- |
 Reference:80 || Sentence | "Zinc increased MT content in eight tissues, being most effective in pancreas, liver, and small intestine." |
| PMID | 3994507 |
| Year | 1985 |
| Species | Rat |
| Journal | Arch Toxicol |
| RefScore | 0 |
| Source | PArchNLP |
  ||


---

|  |  |
| --- | --- |
 Reference:81 || Sentence | "It is concluded that induction of MT by zinc and in particular by organic chemicals such as IA without exogenously supplied metals interferes with zinc homeostasis and the physiological role of MT in liver and extrahepatic tissues of the rat." |
| PMID | 3994507 |
| Year | 1985 |
| Species | Rat |
| Journal | Arch Toxicol |
| RefScore | 1 |
| Source | PArchNLP |
  ||


---

|  |  |
| --- | --- |
 Reference:82 || Sentence | "The presence of the T cell mitogen phytohemagglutinin (PHA) was found not to be necessary for this Mt induction by Zn." |
| PMID | 2279691 |
| Year | 1990 |
| Species | Human |
| Journal | Gen Pharmacol |
| RefScore | 2 |
| Source | PArchNLP |
  ||


---

|  |  |
| --- | --- |
 Reference:83 || Sentence | "The levels of zinc or cadmium ions needed to induce metallothionein in Menkes' cells were similar to those in normal cells." |
| PMID | 3494730 |
| Year | 1987 |
| Species | Human |
| Journal | J Biol Chem |
| RefScore | 3 |
| Source | PArchNLP |
  ||


---

|  |  |
| --- | --- |
 Reference:84 || Sentence | "The parotid MT was elevated 5.9 and 17 times following Zn treatment at doses of 16 and 80 mg/kg respectively, whereas 4 mg/kg of Cd increased MT 14.4 times in this gland." |
| PMID | 3416991 |
| Year | 1988 |
| Species | Rat |
| Journal | Experientia |
| RefScore | 1 |
| Source | PArchNLP |
  ||


---

|  |  |
| --- | --- |
 Reference:85 || Sentence | "In this study, induction of the isoforms of metallothionein (MT) by Cd and Zn was determined with a high-performance liquid chromatography method." |
| PMID | 3564038 |
| Year | 1987 |
| Species | Rat |
| Journal | Toxicol Appl Pharmacol |
| RefScore | 1 |
| Source | PArchNLP |
  ||


---

|  |  |
| --- | --- |
 Reference:86 || Sentence | "In pancreas, Zn (300-10,000 mumol/kg) induced MT-I and MT-II to similar levels." |
| PMID | 3564038 |
| Year | 1987 |
| Species | Rat |
| Journal | Toxicol Appl Pharmacol |
| RefScore | 0 |
| Source | PArchNLP |
  ||


---

|  |  |
| --- | --- |
 Reference:87 || Sentence | "Exposure of hepatocytes to the established direct inducers Zn and DEX of MT resulted in a manifold increase in MT, independent of whether the cultures were FCS pretreated or not." |
| PMID | 8237077 |
| Year | 1993 |
| Species | Rat |
| Journal | Z Ernahrungswiss |
| RefScore | 2 |
| Source | PArchNLP |
  ||


---

|  |  |
| --- | --- |
 Reference:88 || Sentence | "Time-course studies also revealed a good correlation between the onset of MT induction by Zn (> 3 hr) and that of protection against MNNG (> 3 hr)." |
| PMID | 8560476 |
| Year | 1996 |
| Species | Rat |
| Journal | Toxicol Appl Pharmacol |
| RefScore | 0 |
| Source | PArchNLP |
  ||


---

|  |  |
| --- | --- |
 Reference:89 || Sentence | "In addition to the usual two rat metallothionein (MT) isoforms (MT-I and MT-II), a third isoform of metallothionein (MT-II) is known to be induced by zinc in the liver of rats and mice, and by epidermal growth factors in cultured cells." |
| PMID | 10100506 |
| Year | 1998 |
| Species | Rat |
| Journal | Res Commun Mol Pathol Pharmacol |
| RefScore | 0 |
| Source | PArchNLP |
  ||


---

|  |  |
| --- | --- |
 Reference:90 || Sentence | "To study the effect of Zn on the induction of MT and acute phase proteins, Zn, IL-6 and Dex were administered in various concentrations." |
| PMID | 8799367 |
| Year | 1996 |
| Species | Rat |
| Journal | Int J Immunopharmacol |
| RefScore | 1 |
| Source | PArchNLP |
  ||


---

|  |  |
| --- | --- |
 Reference:91 || Sentence | "Zn administration significantly increased the levels of both metallothionein and reduced glutathione in the pancreas; the metallothionein levels reached a peak of 83-fold of normal levels after 24 h." |
| PMID | 8829186 |
| Year | 1996 |
| Species | Rat |
| Journal | Pancreas |
| RefScore | 1 |
| Source | PArchNLP |
  ||


---

|  |  |
| --- | --- |
 Reference:92 || Sentence | "These findings suggested that Zn increased both metallothionein and glutathione levels in the pancreas and exerted a beneficial effect against ceruleinor taurocholate-induced acute pancreatitis in rats." |
| PMID | 8829186 |
| Year | 1996 |
| Species | Rat |
| Journal | Pancreas |
| RefScore | 0 |
| Source | PArchNLP |
  ||


---

|  |  |
| --- | --- |
 Reference:93 || Sentence | Basal and zinc-induced metallothionein in resistance to cadmium, cisplatin, zinc, and tertbutyl hydroperoxide: studies using MT knockout and antisense-downregulated MT in mammalian cells. |
| Year | 2005 |
| PMID | 16150881 |
| Species | Human |
|  | Mouse |
| Journal | Toxicol Sci |
| RefScore | 1 |
| Source | PArchNLP |
  ||


---

|  |  |
| --- | --- |
 Reference:94 || Sentence | We have shown the protection of human central nervous system (CNS) cultures by zinc (Zn) or cadmium (Cd)-pre-induced metallothionein (MT) synthesis from radiation-induced cytotoxicity (lactate dehydrogenase (LDH) release and neuronal dendritic injury). |
| Year | 2004 |
| PMID | 14687759 |
| Species | Human |
| Journal | Toxicol Lett |
| RefScore | 1 |
| Source | PArchNLP |
  ||


---

|  |  |
| --- | --- |
 Reference:95 || Sentence | The effects of Zn on the activities of protective antioxidant enzymes (superoxide dismutase, catalase, and glutathione peroxidase, both total and selenium-dependent), lipid peroxidation, and metallothionein induction were followed in the gills and digestive gland of these clams. |
| Year | 2004 |
| PMID | 15041262 |
| Journal | Ecotoxicol Environ Saf |
| RefScore | 1 |
| Source | PArchNLP |
  ||


---

|  |  |
| --- | --- |
 Reference:96 || Sentence | Zinc is known to protect cells from peroxidative damage by inducing metallothionein and maintaining the sulfhydryl group stability. |
| Year | 2004 |
| PMID | 15147971 |
| Species | Rat |
| Journal | Biochem Biophys Res Commun |
| RefScore | 2 |
| Source | PArchNLP |
  ||


---

|  |  |
| --- | --- |
 Reference:97 || Sentence | At inflammatory sites, both metallothionein and inducible nitric oxide synthase (iNOS) are induced by the same factors and the zinc released from metallothionein by NO suppresses both the induction and activity of iNOS. |
| Year | 2004 |
| PMID | 15182356 |
| Species | Mouse |
| Journal | Eur J Biochem |
| RefScore | 0 |
| Source | PArchNLP |
  ||


---

|  |  |
| --- | --- |
 Reference:98 || Sentence | RESULTS: The zinc supplement reduced SLC30A1 mRNA (1.4-fold) together with SLC30A1, SLC30A5, and SLC39A4 protein (1.8-fold, 3.7-fold, and to undetectable levels, respectively) in ileal mucosa and increased metallothionein mRNA (1.7-fold). |
| PMID | 15753530 |
| Year | 2005 |
| Species | Human |
| Journal | Gut |
| RefScore | 1 |
| Source | PArchNLP |
  ||


---

|  |  |
| --- | --- |
 Reference:99 || Sentence | The actual mechanism of reduction of Cu concentration of jejunum in rats fed a Zn-overload diet might involve the modulation or inhibition of a Cu transporter protein by Zn and not by the induction of MT. |
| PMID | 16217140 |
| Year | 2005 |
| Species | Rat |
| Journal | Biol Trace Elem Res |
| RefScore | 1 |
| Source | PArchNLP |
  ||


---

|  |  |
| --- | --- |
 Reference:100 || Sentence | Zinc induces metallothionein, a highly effective detoxification protein that binds copper. |
| PMID | 16466879 |
| Year | 2006 |
| Species | Human |
| Journal | Brain Dev |
| RefScore | 1 |
| Source | PArchNLP |
  ||


---

|  |  |
| --- | --- |
 Reference:101 || Sentence | Exposure to Zn alone led to a significant increase in MT concentrations only in C. |
| PMID | 16841249 |
| Year | 2006 |
| Journal | Biometals |
| RefScore | 2 |
| Source | PArchNLP |
  ||


---

|  |  |
| --- | --- |
 Reference:102 || Sentence | Effects of zinc on the induction of metallothionein isoforms in hippocampus in stress rats. |
| PMID | 17018881 |
| Year | 2006 |
| Species | Rat |
| Journal | Exp Biol Med (Maywood) |
| RefScore | 1 |
| Source | PArchNLP |
  ||


---

|  |  |
| --- | --- |
 Reference:103 || Sentence | Metal response element (MRE) binding transcription factor-1 (MTF1) is a six Cys(2)His(2) zinc finger-containing transcription factor required for basal and zinc-induced transcription of metallothionein genes. |
| PMID | 15122909 |
| Year | 2004 |
| Species | Mouse |
| Journal | Biochemistry |
| RefScore | 0 |
| Source | UserNLP |
  ||


---

|  |  |
| --- | --- |
 Reference:104 || Sentence | Zinc stabilizes MT molecule, enhances some of their functional activities as a scavenger of metal ions, of free radicals, toxins and xenobiotics. |
| PMID | 14681972 |
| Year | 2003 |
| Species | Human |
| Journal | Ukr Biokhim Zh |
| RefScore | 0 |
| Source | UserNLP |
  |


---

|  |  |
| --- | --- |
