# Supplementary material for: Dynamic transcriptomic profiles of zebrafish gills in response to zinc depletion
Source: BMC Genomics. 2010 Oct 8;11:548. doi: 10.1186/1471-2164-11-548 (PMC3091697; doi:10.1186/1471-2164-11-548)
Supplement: Additional file 2 — Figure S1 - Interactive Direct Interaction Network of responses to zinc depletion. Mini web-site containing index.html and hyperlinked pages in subdirectory. The web site is an interactive version of Figure 6A containing curated interactions between regulated genes and respective proteins. Legend: Molecular interactions between zinc and proteins encoded by genes changed under zinc depletion. A Direct Interaction Network was created based on curated interactions contained within the PathwayArchitect database and provided through hyperlinks. Red ovals represent proteins and the blue circle symbolizes Zn(II). Dark blue squares denote 'binding', and light blue squares 'expression'; green squares stand for 'regulation', green diamonds for 'metabolism', and green circles for 'promoter binding'. Arrow heads indicate directionality of the interaction where annotated. [file 1471-2164-11-548-S2.ZIP › PathwayArchitect Zn def DIN2/447669.html]

# REGULATION:

|  |  |
| --- | --- |
| Type | REGULATION |
| Effect | Positive |


---

|  |  |
| --- | --- |
| Score | 0 |


---

|  |  |
| --- | --- |
| Reference Count | 9 |


---

|  |  |
| --- | --- |
| Mechanism | Unknown |


---

|  |  |
| --- | --- |
| Reference:0 || Sentence | "Zinc or cadmium treatment up-regulated MTs, MTF-1 and ZnT-1 gene expression levels in both cell lines." |
| PMID | 14568174 |
| Year | 2003 |
| Species | Human |
| Journal | Cancer Lett |
| RefScore | 1 |
| Source | PArchNLP |
  |
|


---

|  |  |
| --- | --- |
 Reference:1 || Sentence | "Metal response element-binding transcription factor-1 (MTF-1) binds specifically to metal response elements (MREs) and transactivates metallothionein (MT) gene expression in response to zinc and cadmium." |
| PMID | 10026134 |
| Year | 1999 |
| Species | Human |
| Journal | J Biol Chem |
| RefScore | 1 |
| Source | PArchNLP |
  ||


---

|  |  |
| --- | --- |
 Reference:2 || Sentence | "However, it has recently been shown that MTF-1, functioning as a metalloregulatory transcription factor, activates metallothionein genes in response to the essential metal zinc." |
| PMID | 11230134 |
| Year | 2001 |
| Species | Mouse |
| Journal | EMBO J |
| RefScore | 0 |
| Source | PArchNLP |
  ||


---

|  |  |
| --- | --- |
 Reference:3 || Sentence | "Of the several transition metals (zinc, cadmium, nickel, silver, copper, and cobalt) examined, only zinc facilitated activation of the DNA binding activity of recombinant MTF-1." |
| PMID | 9507026 |
| Year | 1998 |
| Species | Mouse |
|  | Human |
| Journal | J Biol Chem |
| RefScore | 1 |
| Source | PArchNLP |
  ||


---

|  |  |
| --- | --- |
 Reference:4 || Sentence | "After Zn activation, mouse MTF-1 binding activity was more sensitive to EDTA and was stabilized by DNA binding relative to the Zn finger transcription factor Sp1." |
| PMID | 9111349 |
| Year | 1997 |
| Species | Mouse |
|  | Human |
| Journal | Mol Cell Biol |
| RefScore | 1 |
| Source | PArchNLP |
  ||


---

|  |  |
| --- | --- |
 Reference:5 || Sentence | "Notably, increased MTF-1 DNA binding in response to zinc and MTF-1 nuclear localization was not inhibited in cells preincubated with the different kinase inhibitors despite strong inhibition of MTF-1-mediated gene expression." |
| PMID | 11551972 |
| Year | 2001 |
| Species | Mouse |
| Journal | J Biol Chem |
| RefScore | 2 |
| Source | PArchNLP |
  ||


---

|  |  |
| --- | --- |
 Reference:6 || Sentence | "Our studies suggest that the DNA-binding activity of MTF-1 in vivo and in vitro is reversibly activated by zinc interactions with the zinc-finger domain." |
| PMID | 10605938 |
| Year | 2000 |
| Species | Mouse |
| Journal | Biochem Pharmacol |
| RefScore | 2 |
| Source | PArchNLP |
  ||


---

|  |  |
| --- | --- |
 Reference:7 || Sentence | Each of these stresses may increase labile cellular zinc, leading to nuclear translocation, DNA binding, and transcriptional activation of metallothionein genes (MT genes) by MTF-1. |
| PMID | 16847313 |
| Year | 2006 |
| Species | Mouse |
| Journal | Mol Cell Biol |
| RefScore | 1 |
| Source | UserNLP |
  ||


---

|  |  |
| --- | --- |
 Reference:8 || Sentence | Zinc or cadmium treatment up-regulated MTs, MTF-1 and ZnT-1 gene expression levels in both cell lines. |
| PMID | 14568174 |
| Year | 2003 |
| Species | Human |
| Journal | Cancer Lett |
| RefScore | 0 |
| Source | UserNLP |
  |


---

|  |  |
| --- | --- |
