# Supplementary material for: Dynamic transcriptomic profiles of zebrafish gills in response to zinc depletion
Source: BMC Genomics. 2010 Oct 8;11:548. doi: 10.1186/1471-2164-11-548 (PMC3091697; doi:10.1186/1471-2164-11-548)
Supplement: Additional file 2 — Figure S1 - Interactive Direct Interaction Network of responses to zinc depletion. Mini web-site containing index.html and hyperlinked pages in subdirectory. The web site is an interactive version of Figure 6A containing curated interactions between regulated genes and respective proteins. Legend: Molecular interactions between zinc and proteins encoded by genes changed under zinc depletion. A Direct Interaction Network was created based on curated interactions contained within the PathwayArchitect database and provided through hyperlinks. Red ovals represent proteins and the blue circle symbolizes Zn(II). Dark blue squares denote 'binding', and light blue squares 'expression'; green squares stand for 'regulation', green diamonds for 'metabolism', and green circles for 'promoter binding'. Arrow heads indicate directionality of the interaction where annotated. [file 1471-2164-11-548-S2.ZIP › PathwayArchitect Zn def DIN2/457254.html]

# REGULATION:

|  |  |
| --- | --- |
| Type | REGULATION |
| Effect | None |


---

|  |  |
| --- | --- |
| Score | 0 |


---

|  |  |
| --- | --- |
| Reference Count | 13 |


---

|  |  |
| --- | --- |
| Mechanism | Unknown |


---

|  |  |
| --- | --- |
| Reference:0 || Sentence | "Zinc uptake into liver metallothionein from both plasma and other zinc pools within the liver was increased, whereas the release of metallothionein-bound zinc to plasma decreased." |
| PMID | 2538073 |
| Year | 1989 |
| Species | Rat |
| Journal | Am J Physiol |
| RefScore | 1 |
| Source | PArchNLP |
  |
|  |  |


---

|  |  |
| --- | --- |
 Reference:1 || Sentence | "Compared to controls, gel chromatography of maternal liver from valproic acid-treated dams showed higher 65Zn counts associated with a protein peak of molecular weight of 6,500, the approximate molecular weight of the Zn-binding protein metallothionein." |
| PMID | 2495343 |
| Year | 1989 |
| Species | Rat |
| Journal | J Nutr |
| RefScore | 0 |
| Source | PArchNLP |
  ||  |  |


---

|  |  |
| --- | --- |
 Reference:2 || Sentence | "The results suggest that MT is involved in the regulation of Cu and Zn metabolism during fetal and neonatal development." |
| PMID | 2017784 |
| Year | 1991 |
| Species | Human |
| Journal | Toxicol Lett |
| RefScore | 1 |
| Source | PArchNLP |
  ||  |  |


---

|  |  |
| --- | --- |
 Reference:3 || Sentence | "BACKGROUND: Intracellular zinc concentration and localization are strictly regulated by two main protein components, metallothioneins and membrane transporters." |
| PMID | 15154973 |
| Year | 2004 |
| Species | Human |
| Journal | BMC Genomics |
| RefScore | 2 |
| Source | PArchNLP |
  ||  |  |


---

|  |  |
| --- | --- |
 Reference:4 || Sentence | "The results suggest renal metallothionein is induced by zinc or cadmium through a mechanism that requires altered expression of the metallothionein gene(s)." |
| PMID | 7069515 |
| Year | 1982 |
| Species | Rat |
| Journal | J Nutr |
| RefScore | 2 |
| Source | PArchNLP |
  ||  |  |


---

|  |  |
| --- | --- |
 Reference:5 || Sentence | "Furthermore, the mammalian hippocampi contain metallothionein (MT) isoforms which regulate the steady state concentration of zinc, an important antioxidant." |
| PMID | 10872749 |
| Year | 2000 |
| Species | Rat |
| Journal | Cell Mol Biol (Noisy-le-grand) |
| RefScore | 0 |
| Source | PArchNLP |
  ||  |  |


---

|  |  |
| --- | --- |
 Reference:6 || Sentence | "Recent evidence suggests that metallothionein is a critical determinant in the homeostasis of zinc." |
| PMID | 162047 |
| Year | 1979 |
| Species | Human |
| Journal | Ciba Found Symp |
| RefScore | 2 |
| Source | PArchNLP |
  ||  |  |


---

|  |  |
| --- | --- |
 Reference:7 || Sentence | "This indicates MT may be an important physiological regulator of intracellular zinc and/or other metals." |
| PMID | 8635592 |
| Year | 1996 |
| Species | Human |
| Journal | FEBS Lett |
| RefScore | 2 |
| Source | PArchNLP |
  ||  |  |


---

|  |  |
| --- | --- |
 Reference:8 || Sentence | CONCLUSION: MTs have a key role in Zn and Cu homeostasis in harbour porpoises. |
| PMID | 16464247 |
| Year | 2006 |
| Journal | BMC Ecol |
| RefScore | 0 |
| Source | PArchNLP |
  ||  |  |


---

|  |  |
| --- | --- |
 Reference:9 || Sentence | Zip14 siRNA treatment also decreased metallothionein mRNA levels, suggesting that compensatory mechanisms were not sufficient to restore intracellular zinc. |
| PMID | 16950869 |
| Year | 2006 |
| Species | Mouse |
|  | Human |
| Journal | Proc Natl Acad Sci U S A |
| RefScore | 2 |
| Source | PArchNLP |
  ||  |  |


---

|  |  |
| --- | --- |
 Reference:10 || Sentence | Many studies have suggested that MT plays a role in the homeostasis of essential metals such as zinc and copper, detoxification of toxic metals such as cadmium, and protection against oxidative stress. |
| PMID | 17018867 |
| Year | 2006 |
| Journal | Exp Biol Med (Maywood) |
| RefScore | 1 |
| Source | PArchNLP |
  ||  |  |


---

|  |  |
| --- | --- |
 Reference:11 || Sentence | The role of MT in regulating intracellular labile zinc, thus influencing zinc (Zn)-modulated protein activity, may be a key factor in the response to stress and other metabolic challenges. |
| PMID | 17018878 |
| Year | 2006 |
| Species | Mouse |
| Journal | Exp Biol Med (Maywood) |
| RefScore | 1 |
| Source | PArchNLP |
  ||  |  |


---

|  |  |
| --- | --- |
 Reference:12 || Sentence | Thus, both ZnT1 and MT genes contribute to zinc resistance in BHK cells, whereas ZnT1 plays a larger role in regulating free zinc levels. |
| PMID | 15041749 |
| Year | 2004 |
| Journal | Proc Natl Acad Sci U S A |
| RefScore | 0 |
| Source | UserNLP |
  |


---

|  |  |
| --- | --- |
