# Supplementary material for: Dynamic transcriptomic profiles of zebrafish gills in response to zinc depletion
Source: BMC Genomics. 2010 Oct 8;11:548. doi: 10.1186/1471-2164-11-548 (PMC3091697; doi:10.1186/1471-2164-11-548)
Supplement: Additional file 2 — Figure S1 - Interactive Direct Interaction Network of responses to zinc depletion. Mini web-site containing index.html and hyperlinked pages in subdirectory. The web site is an interactive version of Figure 6A containing curated interactions between regulated genes and respective proteins. Legend: Molecular interactions between zinc and proteins encoded by genes changed under zinc depletion. A Direct Interaction Network was created based on curated interactions contained within the PathwayArchitect database and provided through hyperlinks. Red ovals represent proteins and the blue circle symbolizes Zn(II). Dark blue squares denote 'binding', and light blue squares 'expression'; green squares stand for 'regulation', green diamonds for 'metabolism', and green circles for 'promoter binding'. Arrow heads indicate directionality of the interaction where annotated. [file 1471-2164-11-548-S2.ZIP › PathwayArchitect Zn def DIN2/464575.html]

# REGULATION:

|  |  |
| --- | --- |
| Type | REGULATION |
| Effect | Positive |


---

|  |  |
| --- | --- |
| Score | 0 |


---

|  |  |
| --- | --- |
| Reference Count | 34 |


---

|  |  |
| --- | --- |
| Mechanism | Unknown |


---

|  |  |
| --- | --- |
| Reference:0 || Sentence | "Such a release by oxidants and the preservation of zinc binding by antioxidants place MT under the control of the cellular redox state and, consequently, energy metabolism." |
| PMID | 10801959 |
| Year | 2000 |
| Species | Human |
| Journal | J Nutr |
| RefScore | 2 |
| Source | PArchNLP |
  |
|  |  |


---

|  |  |
| --- | --- |
 Reference:1 || Sentence | "Endotoxin reduced serum zinc levels while elevating zinc associated with hepatic metallothionein (Zn-MT) in control, fasted, and zinc-depleted rats." |
| PMID | 6388345 |
| Year | 1984 |
| Species | Rat |
| Journal | Am J Physiol |
| RefScore | 1 |
| Source | PArchNLP |
  ||  |  |


---

|  |  |
| --- | --- |
 Reference:2 || Sentence | "Unlike zinc, copper in the serum and that associated with metallothionein showed little response to endotoxin." |
| PMID | 6388345 |
| Year | 1984 |
| Species | Rat |
| Journal | Am J Physiol |
| RefScore | 1 |
| Source | PArchNLP |
  ||  |  |


---

|  |  |
| --- | --- |
 Reference:3 || Sentence | "In addition, adrenalectomy only partially inhibited the ability of IL-1 to depress serum zinc levels and increase the amount of zinc associated with hepatic metallothionein." |
| PMID | 6333572 |
| Year | 1984 |
| Species | Rat |
| Journal | Life Sci |
| RefScore | 2 |
| Source | PArchNLP |
  ||  |  |


---

|  |  |
| --- | --- |
 Reference:4 || Sentence | "Gel filtration showed that both Cu and Zn were associated with the plasma MT of pregnant rats." |
| PMID | 8456105 |
| Year | 1993 |
| Species | Rat |
| Journal | Proc Soc Exp Biol Med |
| RefScore | 2 |
| Source | PArchNLP |
  ||  |  |


---

|  |  |
| --- | --- |
 Reference:5 || Sentence | "Unlike Zn2+, 65Zn exhibited marked fluctuations within the 24-h period following feeding, indicating that zinc associated with metallothionein may be capable of exchange and/or dissociation." |
| PMID | 7223884 |
| Year | 1981 |
| Species | Rat |
| Journal | Am J Physiol |
| RefScore | 2 |
| Source | PArchNLP |
  ||  |  |


---

|  |  |
| --- | --- |
 Reference:6 || Sentence | "The comparative influence of dietary zinc status and recombinant human interleukin-1 alpha (rhIL-1 alpha) and recombinant human interleukin-6 (rhIL-6) on metallothionein (MT) gene expression was examined in rat bone marrow and liver." |
| PMID | 8463865 |
| Year | 1993 |
| Species | Rat |
| Journal | J Nutr |
| RefScore | 0 |
| Source | PArchNLP |
  ||  |  |


---

|  |  |
| --- | --- |
 Reference:7 || Sentence | "Both Zn and MT levels increased in pancreas, liver, and kidney of rats injected with Zn or Zn+STZ." |
| PMID | 8015348 |
| Year | 1994 |
| Species | Rat |
| Journal | Life Sci |
| RefScore | 1 |
| Source | PArchNLP |
  ||  |  |


---

|  |  |
| --- | --- |
 Reference:8 || Sentence | "The induction of metallothionein likely has a critical influence on the zinc economy of the RPE." |
| PMID | 1572208 |
| Year | 1992 |
| Species | Human |
| Journal | Curr Eye Res |
| RefScore | 1 |
| Source | PArchNLP |
  ||  |  |


---

|  |  |
| --- | --- |
 Reference:9 || Sentence | "An increased body retention of Cd in rats orally pretreated with Cd or Zn is explained by induction of hepatic and renal metallothionein." |
| PMID | 12504351 |
| Year | 2002 |
| Species | Rat |
| Journal | Toxicology |
| RefScore | 2 |
| Source | PArchNLP |
  ||  |  |


---

|  |  |
| --- | --- |
 Reference:10 || Sentence | "Since MT is a zinc binding protein and certain inducers of MT including zinc play a role in apoptosis, studies were undertaken to compare the expression of MT and the presence of apoptotic cells (APPC) in both primary HCC and metastatic adenocarcinoma." |
| PMID | 9602331 |
| Year | 1998 |
| Species | Human |
| Journal | Histopathology |
| RefScore | 3 |
| Source | PArchNLP |
  ||  |  |


---

|  |  |
| --- | --- |
 Reference:11 || Sentence | "As the amount of MT in cells increased upon exposure to 50 and 100 micromol zinc/L, the rate of 67Cu transport decreased." |
| PMID | 8648446 |
| Year | 1996 |
| Species | Human |
| Journal | J Nutr |
| RefScore | 1 |
| Source | PArchNLP |
  ||  |  |


---

|  |  |
| --- | --- |
 Reference:12 || Sentence | "Metallothionein also appears to participate in metabolic interactions between zinc and copper." |
| PMID | 3303133 |
| Year | 1987 |
| Species | Human |
| Journal | Prog Food Nutr Sci |
| RefScore | 2 |
| Source | PArchNLP |
  ||  |  |


---

|  |  |
| --- | --- |
 Reference:13 || Sentence | "In JIB rats, however, liver copper, MT protein, and MT mRNA were significantly elevated, and a high proportion of the intracellular zinc and copper was associated with MT." |
| PMID | 11090006 |
| Year | 2000 |
| Species | Rat |
| Journal | Am Surg |
| RefScore | 2 |
| Source | PArchNLP |
  ||  |  |


---

|  |  |
| --- | --- |
 Reference:14 || Sentence | "In the present study, the effects of oral Cd exposure (0, 50, 100, 200 ppm in drinking water) on serum sex hormone levels, the expression of MT-I and MT-II mRNA, and the zinc content of rat prostate were assessed." |
| PMID | 12604174 |
| Year | 2003 |
| Species | Rat |
| Journal | Toxicology |
| RefScore | 0 |
| Source | PArchNLP |
  ||  |  |


---

|  |  |
| --- | --- |
 Reference:15 || Sentence | "HPLC fractionation of samples from maternal livers showed a higher percentage of Zn, Cu and 65Zn associated with the metallothionein peak in samples from untreated diabetic dams compared with other groups, whereas the percentages of Zn, Cu and 65Zn per fraction in fetal liver were similar." |
| PMID | 1527640 |
| Year | 1992 |
| Species | Rat |
| Journal | J Nutr |
| RefScore | 1 |
| Source | PArchNLP |
  ||  |  |


---

|  |  |
| --- | --- |
 Reference:16 || Sentence | "Compared to controls, liver and kidneys of diabetic dams showed an increased concentration of zinc and copper that was associated with metallothionein." |
| PMID | 2484570 |
| Year | 1988 |
| Species | Rat |
| Journal | Biol Trace Elem Res |
| RefScore | 2 |
| Source | PArchNLP |
  ||  |  |


---

|  |  |
| --- | --- |
 Reference:17 || Sentence | "It suggested that MT played an important role in the modulation and maintenance of intracellular zinc homeostasis of osteoblasts." |
| PMID | 11321951 |
| Year | 2001 |
| Species | Rat |
| Journal | Wei Sheng Yan Jiu |
| RefScore | 1 |
| Source | PArchNLP |
  ||  |  |


---

|  |  |
| --- | --- |
 Reference:18 || Sentence | "The increase in the zinc-thionein pool by exposure to ZnCl2 in vivo was determined to be primarily due to a stimulation of metallothionein biosynthesis." |
| PMID | 697759 |
| Year | 1978 |
| Species | Rat |
| Journal | Biochem J |
| RefScore | 1 |
| Source | PArchNLP |
  ||  |  |


---

|  |  |
| --- | --- |
 Reference:19 || Sentence | "We have recently found that the synthesis of metallothionein in the renal cortex increases in NPX and SO rats given zinc." |
| PMID | 1729759 |
| Year | 1992 |
| Species | Rat |
| Journal | Toxicology |
| RefScore | 1 |
| Source | PArchNLP |
  ||  |  |


---

|  |  |
| --- | --- |
 Reference:20 || Sentence | "Conversely, mercury only partially replaced the zinc associated with metallothionein, and a bolus of zinc was completely unable to bind to the already zinc-saturated metallothionein." |
| PMID | 2778848 |
| Year | 1989 |
| Species | Rat |
| Journal | J Toxicol Environ Health |
| RefScore | 2 |
| Source | PArchNLP |
  ||  |  |


---

|  |  |
| --- | --- |
 Reference:21 || Sentence | "These findings indicate that the hepatic zinc response via MT induction influences zinc metabolism in the body after tumor transplantation." |
| PMID | 10772964 |
| Year | 2000 |
| Species | Mouse |
|  | Rat |
| Journal | Biochem Biophys Res Commun |
| RefScore | 1 |
| Source | PArchNLP |
  ||  |  |


---

|  |  |
| --- | --- |
 Reference:22 || Sentence | "Oral intake of zinc sulphate induces the expression of metallothioneins, enzymes that decrease oxidative stress, and leads to higher serum zinc, and lower copper levels." |
| PMID | 14625013 |
| Year | 2003 |
| Species | Mouse |
|  | Human |
| Journal | Neurosci Lett |
| RefScore | 2 |
| Source | PArchNLP |
  ||  |  |


---

|  |  |
| --- | --- |
 Reference:23 || Sentence | "This observation suggests that MT is capable of influencing gene expression, perhaps by regulating the level of intracellular free zinc." |
| PMID | 10774926 |
| Year | 2000 |
| Species | Mouse |
|  | Human |
| Journal | Cell Mol Biol (Noisy-le-grand) |
| RefScore | 2 |
| Source | PArchNLP |
  ||  |  |


---

|  |  |
| --- | --- |
 Reference:24 || Sentence | "Metallothioneins (MTs) may modulate a variety of cellular processes by regulating the activity of zinc-binding proteins." |
| PMID | 10023665 |
| Year | 1999 |
| Species | Human |
| Journal | Oncogene |
| RefScore | 2 |
| Source | PArchNLP |
  ||  |  |


---

|  |  |
| --- | --- |
 Reference:25 || Sentence | "Therefore, during periods of active DNA synthesis there is likely to be an increased demand for Zn, which could be met by elevated MT synthesis." |
| PMID | 3600629 |
| Year | 1987 |
| Species | Human |
| Journal | Mol Cell Biol |
| RefScore | 1 |
| Source | PArchNLP |
  ||  |  |


---

|  |  |
| --- | --- |
 Reference:26 || Sentence | "Plasma Zn decreased after AJ injection (60% of control values at 8 h), and this was associated with a 4.5-fold increase in hepatic MT at 8 h." |
| PMID | 1384614 |
| Year | 1992 |
| Species | Rat |
| Journal | Biol Trace Elem Res |
| RefScore | 2 |
| Source | PArchNLP |
  ||  |  |


---

|  |  |
| --- | --- |
 Reference:27 || Sentence | "Among milk proteins, zinc was associated with casein, albumin, lactoferrin, and metallothionein, whereas among LMW substances a zinc peak could be observed exclusively with citrate." |
| PMID | 1822333 |
| Year | 1991 |
| Species | Human |
| Journal | J Trace Elem Electrolytes Health Dis |
| RefScore | 2 |
| Source | PArchNLP |
  ||  |  |


---

|  |  |
| --- | --- |
 Reference:28 || Sentence | "Zinc supplementation was associated with increased concentrations of hepatic metallothioneins together with decreased concentrations of proline-hydroxylase and collagen but to a lesser degree than in alcoholic animals." |
| PMID | 7657481 |
| Year | 1995 |
| Species | Rat |
| Journal | Int J Vitam Nutr Res |
| RefScore | 2 |
| Source | PArchNLP |
  ||  |  |


---

|  |  |
| --- | --- |
 Reference:29 || Sentence | "Zinc's mechanism of action involves induction of intestinal cell metallothionein (Mt), which blocks copper absorption from the intestinal track." |
| PMID | 11585025 |
| Year | 2001 |
| Species | Human |
| Journal | Expert Opin Pharmacother |
| RefScore | 0 |
| Source | PArchNLP |
  ||  |  |


---

|  |  |
| --- | --- |
 Reference:30 || Sentence | "Zn concentration was increased by metallothionein induction." |
| PMID | 12498321 |
| Year | 2002 |
| Species | Mouse |
|  | Human |
| Journal | Tohoku J Exp Med |
| RefScore | 2 |
| Source | PArchNLP |
  ||  |  |


---

|  |  |
| --- | --- |
 Reference:31 || Sentence | "In kidney, although Cd or Zn treatment separately had no effect on MT or MT-1 mRNA content, injection of Cd followed by Zn resulted in significantly increased levels of renal MT and MT-1 mRNA." |
| PMID | 2219126 |
| Year | 1990 |
| Species | Rat |
| Journal | Toxicology |
| RefScore | 2 |
| Source | PArchNLP |
  ||  |  |


---

|  |  |
| --- | --- |
 Reference:32 || Sentence | "It is concluded that induction of MT by zinc and in particular by organic chemicals such as IA without exogenously supplied metals interferes with zinc homeostasis and the physiological role of MT in liver and extrahepatic tissues of the rat." |
| PMID | 3994507 |
| Year | 1985 |
| Species | Rat |
| Journal | Arch Toxicol |
| RefScore | 1 |
| Source | PArchNLP |
  ||  |  |


---

|  |  |
| --- | --- |
 Reference:33 || Sentence | "To study the effect of Zn on the induction of MT and acute phase proteins, Zn, IL-6 and Dex were administered in various concentrations." |
| PMID | 8799367 |
| Year | 1996 |
| Species | Rat |
| Journal | Int J Immunopharmacol |
| RefScore | 1 |
| Source | PArchNLP |
  |


---

|  |  |
| --- | --- |
