# Supplementary material for: Dynamic transcriptomic profiles of zebrafish gills in response to zinc depletion
Source: BMC Genomics. 2010 Oct 8;11:548. doi: 10.1186/1471-2164-11-548 (PMC3091697; doi:10.1186/1471-2164-11-548)
Supplement: Additional file 2 — Figure S1 - Interactive Direct Interaction Network of responses to zinc depletion. Mini web-site containing index.html and hyperlinked pages in subdirectory. The web site is an interactive version of Figure 6A containing curated interactions between regulated genes and respective proteins. Legend: Molecular interactions between zinc and proteins encoded by genes changed under zinc depletion. A Direct Interaction Network was created based on curated interactions contained within the PathwayArchitect database and provided through hyperlinks. Red ovals represent proteins and the blue circle symbolizes Zn(II). Dark blue squares denote 'binding', and light blue squares 'expression'; green squares stand for 'regulation', green diamonds for 'metabolism', and green circles for 'promoter binding'. Arrow heads indicate directionality of the interaction where annotated. [file 1471-2164-11-548-S2.ZIP › PathwayArchitect Zn def DIN2/470083.html]

# REGULATION:

|  |  |
| --- | --- |
| Type | REGULATION |
| Effect | None |


---

|  |  |
| --- | --- |
| Score | 0 |


---

|  |  |
| --- | --- |
| Reference Count | 10 |


---

|  |  |
| --- | --- |
| Mechanism | Unknown |


---

|  |  |
| --- | --- |
| Reference:0 || Sentence | "Mucosal zinc concentration rose to a maximum by 6 hours after feeding and plateaued at slightly lower levels thereafter, due to association of this zinc with metallothionein." |
| PMID | 6115000 |
| Year | 1981 |
| Species | Rat |
| Journal | J Nutr |
| RefScore | 2 |
| Source | PArchNLP |
  |
|  |  |


---

|  |  |
| --- | --- |
 Reference:1 || Sentence | "Exposure to 80 microM Zn for 24 hr produced a 70-fold increase in MT." |
| PMID | 3762311 |
| Year | 1986 |
| Species | Human |
| Journal | Life Sci |
| RefScore | 2 |
| Source | PArchNLP |
  ||  |  |


---

|  |  |
| --- | --- |
 Reference:2 || Sentence | "As the amount of MT in cells increased upon exposure to 50 and 100 micromol zinc/L, the rate of 67Cu transport decreased." |
| PMID | 8648446 |
| Year | 1996 |
| Species | Human |
| Journal | J Nutr |
| RefScore | 1 |
| Source | PArchNLP |
  ||  |  |


---

|  |  |
| --- | --- |
 Reference:3 || Sentence | "Maternal zinc supply is therefore a major determinant of MT-I concentrations in neonatal tissues, and assay of this protein in blood plasma and red cells may give a useful indication of zinc status." |
| PMID | 3655938 |
| Year | 1987 |
| Species | Rat |
| Journal | J Nutr |
| RefScore | 1 |
| Source | PArchNLP |
  ||  |  |


---

|  |  |
| --- | --- |
 Reference:4 || Sentence | "Since treatment with zinc results in a large increase in hepatic concentration of metallothionein and that some product of 14CCl4 appeared to bind to zinc-induced metallothionein, it was suggested that metallothionein may protect against CCl4-induced liver damage by sequestering reactive metabolites of CCl4." |
| PMID | 7428956 |
| Year | 1980 |
| Species | Rat |
| Journal | Fed Proc |
| RefScore | 1 |
| Source | PArchNLP |
  ||  |  |


---

|  |  |
| --- | --- |
 Reference:5 || Sentence | "The interaction of Zn, stress and endotoxin on liver metallothionein (MT) regulation has been studied in the rat." |
| PMID | 8599853 |
| Year | 1996 |
| Species | Rat |
| Journal | Chem Biol Interact |
| RefScore | 1 |
| Source | PArchNLP |
  ||  |  |


---

|  |  |
| --- | --- |
 Reference:6 || Sentence | "A pronounced increase in intestinal metallothionein levels and a sharp drop in 64Cu absorption were found 4 to 5 days after the initiation of zinc treatment." |
| PMID | 1517684 |
| Year | 1992 |
| Species | Human |
| Journal | J Lab Clin Med |
| RefScore | 2 |
| Source | PArchNLP |
  ||  |  |


---

|  |  |
| --- | --- |
 Reference:7 || Sentence | To confirm our previous observations on the effectiveness of long term treatment with Zn on Long-Evans Cinnamon (LEC) rats, we extended these studies determining the effects of Zn on trace elements, metallothionein (MT) concentrations and immunolocalization, and on the levels of both MT-1 and MT-2 mRNAs in the LEC rat kidneys. |
| Year | 2004 |
| PMID | 15062872 |
| Species | Rat |
| Journal | Biochim Biophys Acta |
| RefScore | 1 |
| Source | PArchNLP |
  ||  |  |


---

|  |  |
| --- | --- |
 Reference:8 || Sentence | After intestinal zinc perfusion for 80 min, the concentrations of zinc and metallothionein (MT) in the intestinal mucosal and liver tissue were assayed. |
| Year | 2004 |
| PMID | 15122057 |
| Species | Rat |
| Journal | Nephron Physiol |
| RefScore | 1 |
| Source | PArchNLP |
  ||  |  |


---

|  |  |
| --- | --- |
 Reference:9 || Sentence | Tissue metallothionein (MT) significantly increased in the treated rats and positively correlated with Zn concentrations within the liver. |
| PMID | 15998441 |
| Year | 2005 |
| Species | Rat |
| Journal | Liver Int |
| RefScore | 2 |
| Source | PArchNLP |
  |


---

|  |  |
| --- | --- |
