# Supplementary material for: Dynamic transcriptomic profiles of zebrafish gills in response to zinc depletion
Source: BMC Genomics. 2010 Oct 8;11:548. doi: 10.1186/1471-2164-11-548 (PMC3091697; doi:10.1186/1471-2164-11-548)
Supplement: Additional file 2 — Figure S1 - Interactive Direct Interaction Network of responses to zinc depletion. Mini web-site containing index.html and hyperlinked pages in subdirectory. The web site is an interactive version of Figure 6A containing curated interactions between regulated genes and respective proteins. Legend: Molecular interactions between zinc and proteins encoded by genes changed under zinc depletion. A Direct Interaction Network was created based on curated interactions contained within the PathwayArchitect database and provided through hyperlinks. Red ovals represent proteins and the blue circle symbolizes Zn(II). Dark blue squares denote 'binding', and light blue squares 'expression'; green squares stand for 'regulation', green diamonds for 'metabolism', and green circles for 'promoter binding'. Arrow heads indicate directionality of the interaction where annotated. [file 1471-2164-11-548-S2.ZIP › PathwayArchitect Zn def DIN2/522357.html]

# BINDING:

|  |  |
| --- | --- |
| Type | BINDING |
| Effect | None |


---

|  |  |
| --- | --- |
| Score | 0 |


---

|  |  |
| --- | --- |
| Reference Count | 6 |


---

|  |  |
| --- | --- |
| Mechanism | Unknown |


---

|  |  |
| --- | --- |
| Reference:0 || Sentence | "The C6HC consensus pattern C-x(4)-C-x(14-30)-C-x(1-4)-C-x(4)-C-x(2)-C-x(4)-H-x(4)-C defines this structure as the fourth family member of the zinc-binding RING, LIM, and LAP/PHD fingers." |
| PMID | 10422847 |
| Year | 1999 |
| Species | Human |
| Journal | Protein Sci |
| RefScore | 0 |
| Source | PArchNLP |
  |
|  |  |


---

|  |  |
| --- | --- |
 Reference:1 || Sentence | "The structural similarities of N- and C-terminal zinc-binding modules of the LIM domains, despite limited sequence identity, lead to the proposal of a single zinc-binding motif in LIM domains." |
| PMID | 8841116 |
| Year | 1996 |
| Species | Human |
| Journal | Biochemistry |
| RefScore | 2 |
| Source | PArchNLP |
  ||  |  |


---

|  |  |
| --- | --- |
 Reference:2 || Sentence | "Comparisons of the NF structure with those of other C4-type zinc binding motifs, including hormone receptor and LIM domains, also reveal substantial structural homology." |
| PMID | 10212985 |
| Year | 1999 |
| Species | Mouse |
| Journal | J Biomol NMR |
| RefScore | 1 |
| Source | PArchNLP |
  ||  |  |


---

|  |  |
| --- | --- |
 Reference:3 || Sentence | "LMO4 belongs to a family of transcriptional regulators that comprises two zinc-binding LIM domains." |
| PMID | 14966286 |
| Year | 2004 |
| Species | Mouse |
| Journal | Mol Cell Biol |
| RefScore | 0 |
| Source | PArchNLP |
  ||  |  |


---

|  |  |
| --- | --- |
 Reference:4 || Sentence | "The zinc-binding LIM domains mediate protein-protein interactions, and interactions between nuclear LIM proteins and transcription factors with restricted expression patterns have been demonstrated." |
| PMID | 8876198 |
| Year | 1996 |
| Species | Human |
|  | Rat |
|  | Mouse |
| Journal | Proc Natl Acad Sci U S A |
| RefScore | 1 |
| Source | PArchNLP |
  ||  |  |


---

|  |  |
| --- | --- |
 Reference:5 || Sentence | "Sequence analysis has shown that this human cysteine-rich heart protein (hCRHP) is a protein of 77 amino acids and possesses a LIM motif which is considered to be able to bind zinc." |
| PMID | 7999070 |
| Year | 1994 |
| Species | Human |
|  | Rat |
| Journal | Biochem Biophys Res Commun |
| RefScore | 0 |
| Source | PArchNLP |
  |


---

|  |  |
| --- | --- |
