# Supplementary material for: Dynamic transcriptomic profiles of zebrafish gills in response to zinc depletion
Source: BMC Genomics. 2010 Oct 8;11:548. doi: 10.1186/1471-2164-11-548 (PMC3091697; doi:10.1186/1471-2164-11-548)
Supplement: Additional file 2 — Figure S1 - Interactive Direct Interaction Network of responses to zinc depletion. Mini web-site containing index.html and hyperlinked pages in subdirectory. The web site is an interactive version of Figure 6A containing curated interactions between regulated genes and respective proteins. Legend: Molecular interactions between zinc and proteins encoded by genes changed under zinc depletion. A Direct Interaction Network was created based on curated interactions contained within the PathwayArchitect database and provided through hyperlinks. Red ovals represent proteins and the blue circle symbolizes Zn(II). Dark blue squares denote 'binding', and light blue squares 'expression'; green squares stand for 'regulation', green diamonds for 'metabolism', and green circles for 'promoter binding'. Arrow heads indicate directionality of the interaction where annotated. [file 1471-2164-11-548-S2.ZIP › PathwayArchitect Zn def DIN2/539233.html]

# TRANSPORT:

|  |  |
| --- | --- |
| Type | TRANSPORT |
| Effect | None |


---

|  |  |
| --- | --- |
| Score | 0 |


---

|  |  |
| --- | --- |
| Reference Count | 10 |


---

|  |  |
| --- | --- |
| Mechanism | Unknown |


---

|  |  |
| --- | --- |
| Reference:0 || Sentence | "Metallothionein (MT), despite its high metal binding constant (KZn = 3.2 x 10(13) M-1 at pH 7.4), can transfer zinc to the apoforms of zinc enzymes that have inherently lower stability constants." |
| PMID | 9520393 |
| Year | 1998 |
| Species | Human |
| Journal | Proc Natl Acad Sci U S A |
| RefScore | 2 |
| Source | PArchNLP |
  |
|  |  |


---

|  |  |
| --- | --- |
 Reference:1 || Sentence | "However, excess zinc can also be cytotoxic, and zinc transporters as well as metallothioneins serve as zinc detoxificating systems." |
| PMID | 12699353 |
| Year | 2003 |
| Species | Human |
| Journal | Curr Drug Targets |
| RefScore | 1 |
| Source | PArchNLP |
  ||  |  |


---

|  |  |
| --- | --- |
 Reference:2 || Sentence | "Metallothionein (MT) is a two-domain protein with zinc thiolate clusters that bind and release zinc depending on the redox states of the sulfur ligands." |
| PMID | 12081484 |
| Year | 2002 |
| Species | Human |
| Journal | Biochemistry |
| RefScore | 2 |
| Source | PArchNLP |
  ||  |  |


---

|  |  |
| --- | --- |
 Reference:3 || Sentence | "In addition, selenium compounds that have the capacity to form selenol(ate)s catalytically couple with the glutathione/glutathione disulfide and metallothionein/thionein redox pairs to either release or bind zinc." |
| PMID | 12730443 |
| Year | 2003 |
| Species | Human |
| Journal | J Nutr |
| RefScore | 1 |
| Source | PArchNLP |
  ||  |  |


---

|  |  |
| --- | --- |
 Reference:4 || Sentence | "The results show the transfer of the essential metal, zinc from hepatic MT to other proteins and the specific binding of cadmium, the non-essential metal to MT during postnatal development in rats." |
| PMID | 4024125 |
| Year | 1985 |
| Species | Rat |
| Journal | Toxicology |
| RefScore | 1 |
| Source | PArchNLP |
  ||  |  |


---

|  |  |
| --- | --- |
 Reference:5 || Sentence | "On the basis of these results, a reaction mechanism involving electrophilic attack of NO on thiolate groups and subsequent formation of a nitrogen-centred radical, MTSN(. )OH, as intermediate is proposed for the reaction of NO with MT that leads to zinc release." |
| PMID | 10548558 |
| Year | 1999 |
| Species | Human |
| Journal | Biochem J |
| RefScore | 2 |
| Source | PArchNLP |
  ||  |  |


---

|  |  |
| --- | --- |
 Reference:6 || Sentence | "Metallothionein is thought to be involved in the transfer of zinc to thymulin." |
| PMID | 1502195 |
| Year | 1992 |
| Species | Human |
| Journal | Proc Natl Acad Sci U S A |
| RefScore | 2 |
| Source | PArchNLP |
  ||  |  |


---

|  |  |
| --- | --- |
 Reference:7 || Sentence | "We suggest that this 6-MP-associated redistribution of zinc into maternal liver may be due to induction of maternal metallothionein synthesis by the drug." |
| PMID | 2749581 |
| Year | 1989 |
| Species | Rat |
| Journal | Teratology |
| RefScore | 1 |
| Source | PArchNLP |
  ||  |  |


---

|  |  |
| --- | --- |
 Reference:8 || Sentence | However, in liver and perhaps other cells, Zn(7)-MT is probably important in the cytosolic trafficking of zinc to the mitochondria for the uptake of zinc into the mitochondrial matrix by the putative zinc uptake transporter. |
| Year | 2004 |
| PMID | 15041247 |
| Species | Human |
|  | Rat |
| Journal | J Inorg Biochem |
| RefScore | 2 |
| Source | PArchNLP |
  ||  |  |


---

|  |  |
| --- | --- |
 Reference:9 || Sentence | Indeed, these polymorphisms are associated with chronic inflammation, low zinc ion bioavailability, depressed innate immune response and high gene expression of metallothioneins, which have a limited zinc release for an optimal innate immune response in ageing. |
| PMID | 16513158 |
| Year | 2006 |
| Species | Human |
| Journal | Mech Ageing Dev |
| RefScore | 1 |
| Source | PArchNLP |
  |


---

|  |  |
| --- | --- |
