# Supplementary material for: Dynamic transcriptomic profiles of zebrafish gills in response to zinc depletion
Source: BMC Genomics. 2010 Oct 8;11:548. doi: 10.1186/1471-2164-11-548 (PMC3091697; doi:10.1186/1471-2164-11-548)
Supplement: Additional file 2 — Figure S1 - Interactive Direct Interaction Network of responses to zinc depletion. Mini web-site containing index.html and hyperlinked pages in subdirectory. The web site is an interactive version of Figure 6A containing curated interactions between regulated genes and respective proteins. Legend: Molecular interactions between zinc and proteins encoded by genes changed under zinc depletion. A Direct Interaction Network was created based on curated interactions contained within the PathwayArchitect database and provided through hyperlinks. Red ovals represent proteins and the blue circle symbolizes Zn(II). Dark blue squares denote 'binding', and light blue squares 'expression'; green squares stand for 'regulation', green diamonds for 'metabolism', and green circles for 'promoter binding'. Arrow heads indicate directionality of the interaction where annotated. [file 1471-2164-11-548-S2.ZIP › PathwayArchitect Zn def DIN2/565193.html]

# TRANSPORT:

|  |  |
| --- | --- |
| Type | TRANSPORT |
| Effect | None |


---

|  |  |
| --- | --- |
| Score | 0 |


---

|  |  |
| --- | --- |
| Reference Count | 9 |


---

|  |  |
| --- | --- |
| Mechanism | Unknown |


---

|  |  |
| --- | --- |
| Reference:0 || Sentence | "Both proteins have been shown to be expressed in the brain, but only DCT1 is clearly demonstrated to be a transport protein, whereas zinc transporter 1 may only modulate zinc transport in association with as-yet-unidentified proteins." |
| PMID | 10801963 |
| Year | 2000 |
| Species | Human |
|  | Rat |
| Journal | J Nutr |
| RefScore | 1 |
| Source | PArchNLP |
  |
|  |  |


---

|  |  |
| --- | --- |
 Reference:1 || Sentence | "The cloning of human Zn transporters ZnT-like transporter 1 (hZTL1)/ZnT5 (SLC30A5) and hZIP4 (SLC39A4) were major advances in the understanding of the molecular mechanisms of dietary Zn absorption." |
| PMID | 15070437 |
| Year | 2004 |
| Species | Human |
| Journal | Proc Nutr Soc |
| RefScore | 2 |
| Source | PArchNLP |
  ||  |  |


---

|  |  |
| --- | --- |
 Reference:2 || Sentence | "We have examined in rats the comparative response of zinc transporters 1, 2, and 4 (ZnT-1, ZnT-2 and ZnT-4) to dietary zinc." |
| PMID | 11208937 |
| Year | 2001 |
| Species | Rat |
| Journal | J Nutr |
| RefScore | 2 |
| Source | PArchNLP |
  ||  |  |


---

|  |  |
| --- | --- |
 Reference:3 || Sentence | "The cysteine-rich MTs function as an intracellular metal-chelators that bind zinc with high affinity, whereas the transmembrane protein ZnT1 exports zinc from the cell." |
| PMID | 11831458 |
| Year | 2001 |
| Species | Mouse |
| Journal | Biometals |
| RefScore | 0 |
| Source | PArchNLP |
  ||  |  |


---

|  |  |
| --- | --- |
 Reference:4 || Sentence | "The accumulation of zinc in the cell is a sum of influx and efflux processes via transporter proteins, like the four Zn transporters (ZnT1-4), the divalent cation transporter 1 (DCT1) and of storage processes mainly bound to metallothionein (MT)." |
| PMID | 14531638 |
| Year | 2003 |
| Species | Mouse |
|  | Human |
|  | Rat |
| Journal | J Trace Elem Med Biol |
| RefScore | 0 |
| Source | PArchNLP |
  ||  |  |


---

|  |  |
| --- | --- |
 Reference:5 || Sentence | "We propose that ZnT-1 transports zinc out of cells and that its absence accounts for the increased sensitivity of mutant cells to zinc toxicity." |
| PMID | 7882967 |
| Year | 1995 |
| Species | Rat |
|  | Mouse |
| Journal | EMBO J |
| RefScore | 1 |
| Source | PArchNLP |
  ||  |  |


---

|  |  |
| --- | --- |
 Reference:6 || Sentence | Differential regulation of zinc efflux transporters ZnT-1, ZnT-5 and ZnT-7 gene expression by zinc levels: a real-time RT-PCR study. |
| Year | 2004 |
| PMID | 15276077 |
| Species | Human |
| Journal | Biochem Pharmacol |
| RefScore | 1 |
| Source | PArchNLP |
  ||  |  |


---

|  |  |
| --- | --- |
 Reference:7 || Sentence | Our study aim was to investigate the effect of acute and chronic exposure to various glucose concentrations on zinc in secretory vesicles, the relation between zinc and insulin, and the presence of two zinc transporters, ZnT1 and ZnT4, in INS-1E cells. |
| PMID | 15824966 |
| Year | 2005 |
| Species | Rat |
| Journal | Horm Metab Res |
| RefScore | 0 |
| Source | PArchNLP |
  ||  |  |


---

|  |  |
| --- | --- |
 Reference:8 || Sentence | The cysteine-rich MTs function as an intracellular metal-chelators that bind zinc with high affinity, whereas the transmembrane protein ZnT1 exports zinc from the cell. |
| PMID | 11831458 |
| Year | 2001 |
| Species | Mouse |
| Journal | Biometals |
| RefScore | 0 |
| Source | UserNLP |
  |


---

|  |  |
| --- | --- |
