# Supplementary material for: Dynamic transcriptomic profiles of zebrafish gills in response to zinc depletion
Source: BMC Genomics. 2010 Oct 8;11:548. doi: 10.1186/1471-2164-11-548 (PMC3091697; doi:10.1186/1471-2164-11-548)
Supplement: Additional file 2 — Figure S1 - Interactive Direct Interaction Network of responses to zinc depletion. Mini web-site containing index.html and hyperlinked pages in subdirectory. The web site is an interactive version of Figure 6A containing curated interactions between regulated genes and respective proteins. Legend: Molecular interactions between zinc and proteins encoded by genes changed under zinc depletion. A Direct Interaction Network was created based on curated interactions contained within the PathwayArchitect database and provided through hyperlinks. Red ovals represent proteins and the blue circle symbolizes Zn(II). Dark blue squares denote 'binding', and light blue squares 'expression'; green squares stand for 'regulation', green diamonds for 'metabolism', and green circles for 'promoter binding'. Arrow heads indicate directionality of the interaction where annotated. [file 1471-2164-11-548-S2.ZIP › PathwayArchitect Zn def DIN2/617213.html]

# BINDING:

|  |  |
| --- | --- |
| Type | BINDING |
| Effect | None |


---

|  |  |
| --- | --- |
| Score | 0 |


---

|  |  |
| --- | --- |
| Reference Count | 3 |


---

|  |  |
| --- | --- |
| Mechanism | Unknown |


---

|  |  |
| --- | --- |
| Reference:0 || Sentence | "The results of molecular genetics studies have shown that familial hypertrophic cardiomyopathy is a disease of the sarcomere involving mutations in 7 different genes encoding proteins of the myofibrillar apparatus: ss-myosin heavy chain, ventricular myosin essential light chain, ventricular myosin regulatory light chain, cardiac troponin T, cardiac troponin I, alpha-tropomyosin, and cardiac myosin binding protein C." |
| PMID | 9742053 |
| Year | 1998 |
| Species | Human |
| Journal | Circ Res |
| RefScore | 2 |
| Source | PArchNLP |
  |
|  |  |


---

|  |  |
| --- | --- |
 Reference:1 || PMID | 9560191 |
| SourceID | 118899 |
| Experimental Condition | in-vitro |
| Description | The three dimensional structure of the 46kda Domain of Human Cardiac Troponin in the Ca2+ Saturated Form was determined by crystallography. The crystal was grown by hanging-drop and vapour diffusion using Troponin C, Troponin T, Troponin I, PEG3350, Lithium Chloride, Tris- HCl, Calcium Chloride, Glycerol pH 8.0, at 293K. X-ray data were collected using synchrotron radiation and were 96.7% complete to a resolution limit of 2.600 angstroms with an overall Rsym of 4.2%. This crystal contained 6 chains per asymmetric unit and belonged to the space group P 1 21 1. The structure was determined using MAD and the modeled structure was refined to an R-factor = 26.4% and had an R-free = 29.8%. PDB: 1J1D, MMDB: 24424. |
| Detection Method | three-dimensional-structure |
| Source | BIND |
  ||  |  |


---

|  |  |
| --- | --- |
 Reference:2 || PMID | 9560191 |
| SourceID | 118905 |
| Experimental Condition | in-vitro |
| Description | The three dimensional structure of the 52kda Domain of Human Cardiac Troponin in the Ca2+ Saturated Form was determined by crystallography. The crystal was grown by hanging-drop and vapour diffusion using Troponin C, Troponin T, Troponin I, PEG3350, Lithium Chloride, Tris- HCl, Calcium Chloride, Glycerol pH 8.0, at 293K. X-ray data were collected using synchrotron radiation and were 99.3% complete to a resolution limit of 3.300 angstroms with an overall Rsym of 5.2%. This crystal contained 6 chains per asymmetric unit and belonged to the space group P 1 21 1. The structure was determined using Molecular Replacement and the modeled structure was refined to an R-factor = 25.1% and had an R-free = 30.8%. PDB: 1J1E, MMDB: 24425. |
| Detection Method | three-dimensional-structure |
| Source | BIND |
  |


---

|  |  |
| --- | --- |
