# Supplementary material for: Dynamic transcriptomic profiles of zebrafish gills in response to zinc depletion
Source: BMC Genomics. 2010 Oct 8;11:548. doi: 10.1186/1471-2164-11-548 (PMC3091697; doi:10.1186/1471-2164-11-548)
Supplement: Additional file 2 — Figure S1 - Interactive Direct Interaction Network of responses to zinc depletion. Mini web-site containing index.html and hyperlinked pages in subdirectory. The web site is an interactive version of Figure 6A containing curated interactions between regulated genes and respective proteins. Legend: Molecular interactions between zinc and proteins encoded by genes changed under zinc depletion. A Direct Interaction Network was created based on curated interactions contained within the PathwayArchitect database and provided through hyperlinks. Red ovals represent proteins and the blue circle symbolizes Zn(II). Dark blue squares denote 'binding', and light blue squares 'expression'; green squares stand for 'regulation', green diamonds for 'metabolism', and green circles for 'promoter binding'. Arrow heads indicate directionality of the interaction where annotated. [file 1471-2164-11-548-S2.ZIP › PathwayArchitect Zn def DIN2/657478.html]

# REGULATION:

|  |  |
| --- | --- |
| Type | REGULATION |
| Effect | Positive |


---

|  |  |
| --- | --- |
| Score | 0 |


---

|  |  |
| --- | --- |
| Reference Count | 5 |


---

|  |  |
| --- | --- |
| Mechanism | Unknown |


---

|  |  |
| --- | --- |
| Reference:0 || Sentence | "On day 1, 28% of the patients had increased cardiospecific troponin T values and 18% had increased creatine kinase MB values." |
| PMID | 9503183 |
| Year | 1998 |
| Species | Human |
| Journal | Eur Heart J |
| RefScore | 1 |
| Source | PArchNLP |
  |
|  |  |


---

|  |  |
| --- | --- |
 Reference:1 || Sentence | "For all pts serum enzyme CPK, CPK MB, SGOT and Troponin T was determined 24 hours before and after the operation." |
| PMID | 8540272 |
| Year | 1995 |
| Species | Human |
| Journal | Acta Cardiol |
| RefScore | 0 |
| Source | PArchNLP |
  ||  |  |


---

|  |  |
| --- | --- |
 Reference:2 || Sentence | "Positive troponin T (Tn-T) test and increased CK-Mb activity (p < 0.22) were observed in 8 patients (group 1), while in group II Tn-T test was negative." |
| PMID | 12187589 |
| Year | 2002 |
| Species | Human |
| Journal | Klin Lab Diagn |
| RefScore | 1 |
| Source | PArchNLP |
  ||  |  |


---

|  |  |
| --- | --- |
 Reference:3 || Sentence | "Within 4 and between 4-8 hours after onset of chest pain, the frequency of elevated results for creatine kinase-MB mass concentration and troponin T in patients with acute myocardial infarction was considerably higher (20-30%) than for creatine kinase and creatine kinase-MB activity." |
| PMID | 8305615 |
| Year | 1993 |
| Species | Human |
| Journal | Eur J Clin Chem Clin Biochem |
| RefScore | 2 |
| Source | PArchNLP |
  ||  |  |


---

|  |  |
| --- | --- |
 Reference:4 || Sentence | "TnT elevation was not invariably accompanied by CK or CK-MB elevation, or by angiographically visible side branch occlusion." |
| PMID | 9687822 |
| Year | 1998 |
| Species | Human |
| Journal | Jpn Heart J |
| RefScore | 1 |
| Source | PArchNLP |
  |


---

|  |  |
| --- | --- |
