# Supplementary material for: Dynamic transcriptomic profiles of zebrafish gills in response to zinc depletion
Source: BMC Genomics. 2010 Oct 8;11:548. doi: 10.1186/1471-2164-11-548 (PMC3091697; doi:10.1186/1471-2164-11-548)
Supplement: Additional file 2 — Figure S1 - Interactive Direct Interaction Network of responses to zinc depletion. Mini web-site containing index.html and hyperlinked pages in subdirectory. The web site is an interactive version of Figure 6A containing curated interactions between regulated genes and respective proteins. Legend: Molecular interactions between zinc and proteins encoded by genes changed under zinc depletion. A Direct Interaction Network was created based on curated interactions contained within the PathwayArchitect database and provided through hyperlinks. Red ovals represent proteins and the blue circle symbolizes Zn(II). Dark blue squares denote 'binding', and light blue squares 'expression'; green squares stand for 'regulation', green diamonds for 'metabolism', and green circles for 'promoter binding'. Arrow heads indicate directionality of the interaction where annotated. [file 1471-2164-11-548-S2.ZIP › PathwayArchitect Zn def DIN2/721829.html]

# REGULATION:

|  |  |
| --- | --- |
| Type | REGULATION |
| Effect | None |


---

|  |  |
| --- | --- |
| Score | 0 |


---

|  |  |
| --- | --- |
| Reference Count | 4 |


---

|  |  |
| --- | --- |
| Mechanism | Unknown |


---

|  |  |
| --- | --- |
| Reference:0 || Sentence | "RESULTS: Depending on the effectiveness of shocks and the energy applied, the cardiac-specific markers cTnT and cTnI, as well as CK-MB mass, showed a significant increase compared with the baseline value before testing and peaked for the most part 4 h after shock application." |
| PMID | 11238297 |
| Year | 2001 |
| Species | Human |
| Journal | Clin Chem |
| RefScore | 1 |
| Source | PArchNLP |
  |
|  |  |


---

|  |  |
| --- | --- |
 Reference:1 || Sentence | "A backward stepwise linear regression model was applied to assess the association between the markers of myocardial injury (CK-MB and cTnT) and the above clinical variables." |
| PMID | 9863571 |
| Year | 1998 |
| Species | Human |
| Journal | Resuscitation |
| RefScore | 0 |
| Source | PArchNLP |
  ||  |  |


---

|  |  |
| --- | --- |
 Reference:2 || Sentence | "There was a significant association between post-procedural cardiac marker concentrations of CK-MB, cTnT and cTnI and the occurrence of procedural angiographic complications (P=0.0003, 0.0002, 0.001, respectively)." |
| PMID | 14659867 |
| Year | 2003 |
| Species | Human |
| Journal | Int J Cardiol |
| RefScore | 0 |
| Source | PArchNLP |
  ||  |  |


---

|  |  |
| --- | --- |
 Reference:3 || Sentence | CK-MB and TnT had higher specificities (73-100%) but significantly lower positive rates (22-27%) than the others (61-68%), resulting in greater sensitivities of H-FABP and myoglobin (75-80%) than those of CK-MB and TnT (29-35%). |
| PMID | 16565558 |
| Year | 2006 |
| Species | Human |
| Journal | Circ J |
| RefScore | 2 |
| Source | PArchNLP |
  |


---

|  |  |
| --- | --- |
