# Supplementary material for: Dynamic transcriptomic profiles of zebrafish gills in response to zinc depletion
Source: BMC Genomics. 2010 Oct 8;11:548. doi: 10.1186/1471-2164-11-548 (PMC3091697; doi:10.1186/1471-2164-11-548)
Supplement: Additional file 2 — Figure S1 - Interactive Direct Interaction Network of responses to zinc depletion. Mini web-site containing index.html and hyperlinked pages in subdirectory. The web site is an interactive version of Figure 6A containing curated interactions between regulated genes and respective proteins. Legend: Molecular interactions between zinc and proteins encoded by genes changed under zinc depletion. A Direct Interaction Network was created based on curated interactions contained within the PathwayArchitect database and provided through hyperlinks. Red ovals represent proteins and the blue circle symbolizes Zn(II). Dark blue squares denote 'binding', and light blue squares 'expression'; green squares stand for 'regulation', green diamonds for 'metabolism', and green circles for 'promoter binding'. Arrow heads indicate directionality of the interaction where annotated. [file 1471-2164-11-548-S2.ZIP › PathwayArchitect Zn def DIN2/734209.html]

# REGULATION:

|  |  |
| --- | --- |
| Type | REGULATION |
| Effect | Positive |


---

|  |  |
| --- | --- |
| Score | 0 |


---

|  |  |
| --- | --- |
| Reference Count | 4 |


---

|  |  |
| --- | --- |
| Mechanism | Unknown |


---

|  |  |
| --- | --- |
| Reference:0 || Sentence | "Zinc or cadmium treatment of cell lines rapidly (3 h) and dramatically (about 12-fold) induced ZnT1 mRNA levels." |
| PMID | 10952993 |
| Year | 2000 |
| Species | Mouse |
| Journal | J Biol Chem |
| RefScore | 1 |
| Source | PArchNLP |
  |
|  |  |


---

|  |  |
| --- | --- |
 Reference:1 || Sentence | "Zinc has been shown to modify specifically the metabolism of cGMP, the activities of protein kinase C and mitogen activated protein kinases, and the activity of transcription factor MTF-1 which controls the transcription of the genes for metallothionein and the zinc transporter ZnT-1." |
| PMID | 11831463 |
| Year | 2001 |
| Species | Human |
| Journal | Biometals |
| RefScore | 0 |
| Source | PArchNLP |
  ||  |  |


---

|  |  |
| --- | --- |
 Reference:2 || Sentence | "ZnT-1 was the first cloned and is associated with zinc efflux." |
| PMID | 9521625 |
| Year | 1998 |
| Species | Human |
| Journal | J Nutr |
| RefScore | 2 |
| Source | PArchNLP |
  ||  |  |


---

|  |  |
| --- | --- |
 Reference:3 || Sentence | "Dietary zinc supplementation elevated the level of intestinal ZnT-1 mRNA and protein approximately 50% and 10%, respectively, but had no effect in the liver." |
| PMID | 9560190 |
| Year | 1998 |
| Species | Rat |
| Journal | Proc Natl Acad Sci U S A |
| RefScore | 1 |
| Source | PArchNLP |
  |


---

|  |  |
| --- | --- |
