# Supplementary material for: Dynamic transcriptomic profiles of zebrafish gills in response to zinc depletion
Source: BMC Genomics. 2010 Oct 8;11:548. doi: 10.1186/1471-2164-11-548 (PMC3091697; doi:10.1186/1471-2164-11-548)
Supplement: Additional file 2 — Figure S1 - Interactive Direct Interaction Network of responses to zinc depletion. Mini web-site containing index.html and hyperlinked pages in subdirectory. The web site is an interactive version of Figure 6A containing curated interactions between regulated genes and respective proteins. Legend: Molecular interactions between zinc and proteins encoded by genes changed under zinc depletion. A Direct Interaction Network was created based on curated interactions contained within the PathwayArchitect database and provided through hyperlinks. Red ovals represent proteins and the blue circle symbolizes Zn(II). Dark blue squares denote 'binding', and light blue squares 'expression'; green squares stand for 'regulation', green diamonds for 'metabolism', and green circles for 'promoter binding'. Arrow heads indicate directionality of the interaction where annotated. [file 1471-2164-11-548-S2.ZIP › PathwayArchitect Zn def DIN2/79437.html]

# PROTEIN: HBG1

|  |  |
| --- | --- |
| Name | HBG1 |
| Type | PROTEIN |
| Description | hemoglobin, gamma A |
| Note | The gamma globin genes (HBG1 and HBG2) are normally expressed in the fetal liver, spleen and bone marrow. Two gamma chains together with two alpha chains constitute fetal hemoglobin (HbF) which is normally replaced by adult hemoglobin (HbA) at birth. In some beta-thalassemias and related conditions, gamma chain production continues into adulthood. The two types of gamma chains differ at residue 136 where glycine is found in the G-gamma product (HBG2) and alanine is found in the A-gamma product (HBG1). The former is predominant at birth. The order of the genes in the beta-globin cluster is: 5'-epsilon -- gamma-G -- gamma-A -- delta -- beta--3' |
| Alias | A-gamma globin |
|  | Hbb-y |
|  | HBG1 |
|  | hemoglobin Y, beta-like embryonic chain |
|  | hemoglobin epsilon 1 |
|  | hemoglobin, epsilon 1 |
|  | Hb F Agamma |
|  | Hbe1 |
|  | HBGR |
|  | gamma A hemoglobin |
|  | HSGGL1 |
|  | HBGA |
|  | Hemoglobin, gamma, regulator of |
|  | hemoglobin gamma-a chain |
|  | epsilon 3 globin gene |
|  | hemoglobin Y beta-like embryonic chain |
|  | PRO2979 |
|  | gamma globin |
|  | Hemoglobin gamma-A chain |


---

|  |  |
| --- | --- |
| GO Component | hemoglobin complex |


---

|  |  |
| --- | --- |
| GO ID | GO:0006810 |
|  | GO:0015671 |
|  | GO:0020037 |
|  | GO:0005506 |
|  | GO:0005833 |
|  | GO:0019825 |
|  | GO:0046872 |
|  | GO:0005344 |


---

|  |  |
| --- | --- |
| MIM | MIM:142200 |


---

|  |  |
| --- | --- |
| Connectivity | 278 |


---

|  |  |
| --- | --- |
| Entrez ID | 94164 |
|  | 3047 |


---

|  |  |
| --- | --- |
| Agilent ID | A\_44\_P743882 |
|  | A\_24\_P289709 |
|  | A\_23\_P53137 |
|  | A\_23\_P64539 |
|  | A\_42\_P591944 |
|  | A\_23\_P64538 |


---

|  |  |
| --- | --- |
| Cellular Localization | Cytosol |
|  | Cytoplasm |
|  | Cell |


---

|  |  |
| --- | --- |
| Pathway | Zn def RIN |
|  | Zn def DIN |


---

|  |  |
| --- | --- |
| GO Process | oxygen transport |
|  | transport |


---

|  |  |
| --- | --- |
| UniGene | Rn.118 |
|  | Hs.530049 |


---

|  |  |
| --- | --- |
| Affymetrix Probeset ID | 237739\_at |
|  | 1388269\_at |
|  | 204848\_x\_at |
|  | 1561855\_x\_at |
|  | 1561854\_at |
|  | 204419\_x\_at |
|  | 213515\_x\_at |
|  | 38585\_at |
|  | g4504352\_3p\_s\_at |
|  | rc\_AA875199\_f\_at |
|  | 217683\_at |
|  | 56724\_at |
|  | Hs.295459.0.A1\_3p\_at |
|  | Hs.323423.0.S1\_3p\_at |
|  | Hs2.375752.1.S1\_3p\_x\_at |
|  | M91036\_rna1\_at |
|  | RC\_AA115963\_at |
|  | RC\_H47526\_at |
|  | RC\_H77414\_f\_at |
|  | RC\_H94956\_at |
|  | RC\_R92458\_f\_at |
|  | X56327cds\_s\_at |


---

|  |  |
| --- | --- |
| GO Function | oxygen transporter activity |
|  | iron ion binding |
|  | heme binding |
|  | oxygen binding |
|  | metal ion binding |


---

|  |  |
| --- | --- |
| Nucleotide | NM\_000559 |
|  | V00512 |
|  | AF487523 |
|  | M32249 |
|  | AF130098 |
|  | X55655 |
|  | BC029387 |
|  | V00514 |
|  | X56328 |
|  | NM\_172093 |
|  | M32724 |
|  | U01317 |
|  | J00176 |
|  | M91037 |
|  | BC010913 |
|  | M11427 |
|  | AY534688 |
|  | X60730 |
|  | NG\_000007 |
|  | BC020719 |
|  | BC010914 |
|  | V00513 |
|  | M91036 |


---

|  |  |
| --- | --- |
| Protein | AAA52637 |
|  | AAS48085 |
|  | AAA58609 |
|  | CAA39188 |
|  | CAA23772 |
|  | NP\_742090 |
|  | AAA16332 |
|  | CAA43138 |
|  | AAL99545 |
|  | AAG35523 |
|  | AAH10914 |
|  | AAB59429 |
|  | AAH29387 |
|  | AAH10913 |
|  | AAA35957 |
|  | AAH20719 |
|  | AAA58493 |
|  | AAA35953 |
|  | CAA23770 |
|  | CAA23771 |
|  | NP\_000550 |
|  | CAA39767 |
|  | P69891 |


---

|  |  |
| --- | --- |
| Organism | Mammal |


---

|  |  |
| --- | --- |
| Location | chromosome 11, 11p15.5 (Homo sapiens) |
|  | chromosome 1, 1q32 (Rattus norvegicus) |


---

|  |  |
| --- | --- |
