# Supplementary material for: Dynamic transcriptomic profiles of zebrafish gills in response to zinc depletion
Source: BMC Genomics. 2010 Oct 8;11:548. doi: 10.1186/1471-2164-11-548 (PMC3091697; doi:10.1186/1471-2164-11-548)
Supplement: Additional file 2 — Figure S1 - Interactive Direct Interaction Network of responses to zinc depletion. Mini web-site containing index.html and hyperlinked pages in subdirectory. The web site is an interactive version of Figure 6A containing curated interactions between regulated genes and respective proteins. Legend: Molecular interactions between zinc and proteins encoded by genes changed under zinc depletion. A Direct Interaction Network was created based on curated interactions contained within the PathwayArchitect database and provided through hyperlinks. Red ovals represent proteins and the blue circle symbolizes Zn(II). Dark blue squares denote 'binding', and light blue squares 'expression'; green squares stand for 'regulation', green diamonds for 'metabolism', and green circles for 'promoter binding'. Arrow heads indicate directionality of the interaction where annotated. [file 1471-2164-11-548-S2.ZIP › PathwayArchitect Zn def DIN2/80305.html]

# PROTEIN: CBX3

|  |  |
| --- | --- |
| Name | CBX3 |
| Type | PROTEIN |
| Description | chromobox homolog 3 (HP1 gamma homolog, Drosophila) |
| Note | At the nuclear envelope, the nuclear lamina and heterochromatin are adjacent to the inner nuclear membrane. The protein encoded by this gene binds DNA and is a component of heterochromatin. This protein also can bind lamin B receptor, an integral membrane protein found in the inner nuclear membrane. The dual binding functions of the encoded protein may explain the association of heterochromatin with the inner nuclear membrane. Two transcript variants encoding the same protein but differing in the 5' UTR, have been found for this gene. |
| Alias | CBX3 |
|  | HP1-GAMMA |
|  | MGC105793 |
|  | Cbx3 |
|  | HP1Hs-gamma |
|  | Hp1-gamma |
|  | chromobox homolog 3 |
|  | HP1 gamma homolog |
|  | HP1g |
|  | MGC105792 |
|  | heterochromatin-like protein 1 |
|  | heterochromatin protein HP1 gamma |
|  | chromobox homolog 3 (Drosophila HP1 gamma) |
|  | Modifier 2 protein |
|  | M32 |
|  | HP1 gamma |
|  | HECH |
|  | heterochromatin protein 1 gamma |
|  | Heterochromatin protein 1 homolog gamma |


---

|  |  |
| --- | --- |
| GO Component | polytene chromosome chromocenter |
|  | chromatin |
|  | nucleus |


---

|  |  |
| --- | --- |
| GO ID | GO:0005634 |
|  | GO:0006333 |
|  | GO:0006355 |
|  | GO:0000785 |
|  | GO:0005515 |
|  | GO:0005701 |
|  | GO:0003682 |
|  | GO:0006350 |
|  | GO:0016568 |


---

|  |  |
| --- | --- |
| MIM | MIM:604477 |


---

|  |  |
| --- | --- |
| Connectivity | 56 |


---

|  |  |
| --- | --- |
| Entrez ID | 297093 |
|  | 12417 |
|  | 11335 |


---

|  |  |
| --- | --- |
| Agilent ID | A\_53\_P129813 |
|  | A\_44\_P448113 |
|  | A\_53\_P150532 |
|  | A\_14\_P119057 |
|  | A\_53\_P178483 |
|  | A\_53\_P101850 |
|  | A\_23\_P31315 |
|  | A\_52\_P397231 |
|  | A\_51\_P117162 |
|  | A\_51\_P160328 |
|  | A\_42\_P634231 |
|  | A\_43\_P20299 |


---

|  |  |
| --- | --- |
| Cellular Localization | Nucleus |
|  | Chromosome |
|  | Organelle |
|  | Cell |


---

|  |  |
| --- | --- |
| Pathway | Zn def RIN |
|  | Master Regulators |
|  | Zn def DIN |


---

|  |  |
| --- | --- |
| GO Process | chromatin assembly or disassembly |
|  | regulation of transcription, DNA-dependent |
|  | chromatin modification |
|  | transcription |


---

|  |  |
| --- | --- |
| UniGene | Hs.381189 |
|  | Mm.371551 |


---

|  |  |
| --- | --- |
| Affymetrix Probeset ID | 100405\_at |
|  | 1371395\_at |
|  | 1374843\_at |
|  | 1432628\_at |
|  | 160272\_at |
|  | 1454305\_at |
|  | 1555920\_at |
|  | 1448504\_a\_at |
|  | 200037\_s\_at |
|  | 201091\_s\_at |
|  | 1439421\_x\_at |
|  | 230998\_at |
|  | 1416884\_at |
|  | 38084\_at |
|  | 38085\_at |
|  | 47980\_at |
|  | 54190\_at |
|  | 73344\_s\_at |
|  | 82714\_at |
|  | 89994\_r\_at |
|  | g7705406\_3p\_s\_at |
|  | Hs2.411153.1.A1\_3p\_at |
|  | Hs.278554.0.A1\_3p\_x\_at |
|  | Msa.2457.0\_at |
|  | rc\_AA851329\_at |
|  | U26312\_s\_at |
|  | 140977\_at |
|  | Hs.145199.0.A1\_3p\_at |
|  | Hs.145199.0.A1\_3p\_x\_at |
|  | 131700\_at |
|  | AA092473\_at |
|  | RC\_H78370\_at |
|  | RC\_W69106\_at |
|  | aa521730\_at |


---

|  |  |
| --- | --- |
| GO Function | protein binding |
|  | chromatin binding |


---

|  |  |
| --- | --- |
| Nucleotide | AJ278616 |
|  | AB026127 |
|  | AK017958 |
|  | AJ278617 |
|  | NM\_007276 |
|  | NM\_016587 |
|  | AK135997 |
|  | AJ278618 |
|  | AF136630 |
|  | U26312 |
|  | BC059831 |
|  | BX648807 |
|  | AK083957 |
|  | AK146376 |
|  | BX647444 |
|  | AJ278619 |
|  | BC000954 |
|  | NM\_001008313 |
|  | NM\_007624 |
|  | AB030905 |
|  | AK002910 |
|  | BC086595 |
|  | BC018354 |
|  | AL542267 |
|  | BC086601 |
|  | BC014380 |
|  | CH236948 |
|  | X56683 |


---

|  |  |
| --- | --- |
| Protein | AAH86595 |
|  | BAC39078 |
|  | AAF62370 |
|  | CAA40012 |
|  | NP\_001008314 |
|  | Q13185 |
|  | BAB22450 |
|  | AAH18354 |
|  | AAB48101 |
|  | EAL24235 |
|  | BAA83340 |
|  | AAH59831 |
|  | NP\_031650 |
|  | NP\_057671 |
|  | P23198 |
|  | AAH86601 |
|  | AAH00954 |
|  | BAE27125 |
|  | AAH14380 |
|  | CAC42944 |
|  | NP\_009207 |


---

|  |  |
| --- | --- |
| Organism | Mammal |


---

|  |  |
| --- | --- |
| Location | chromosome 4, 4q24 (Rattus norvegicus) |
|  | chromosome 7, 7p15.2 (Homo sapiens) |
|  | 6 26.0 cM (Mus musculus) |
|  | chromosome 6, 6 26.0 cM, 6 B-C (Mus musculus) |


---

|  |  |
| --- | --- |
