# Supplementary material for: Dynamic transcriptomic profiles of zebrafish gills in response to zinc depletion
Source: BMC Genomics. 2010 Oct 8;11:548. doi: 10.1186/1471-2164-11-548 (PMC3091697; doi:10.1186/1471-2164-11-548)
Supplement: Additional file 2 — Figure S1 - Interactive Direct Interaction Network of responses to zinc depletion. Mini web-site containing index.html and hyperlinked pages in subdirectory. The web site is an interactive version of Figure 6A containing curated interactions between regulated genes and respective proteins. Legend: Molecular interactions between zinc and proteins encoded by genes changed under zinc depletion. A Direct Interaction Network was created based on curated interactions contained within the PathwayArchitect database and provided through hyperlinks. Red ovals represent proteins and the blue circle symbolizes Zn(II). Dark blue squares denote 'binding', and light blue squares 'expression'; green squares stand for 'regulation', green diamonds for 'metabolism', and green circles for 'promoter binding'. Arrow heads indicate directionality of the interaction where annotated. [file 1471-2164-11-548-S2.ZIP › PathwayArchitect Zn def DIN2/80486.html]

# PROTEIN: TH

|  |  |
| --- | --- |
| Name | TH |
| Type | PROTEIN |
| Description | tyrosine hydroxylase |
| Note | Tyrosine hydroxylase (EC 1.14.16.2) is involved in the conversion of phenylalanine to dopamine. As the rate-limiting enzyme in the synthesis of catecholamines, tyrosine hydroxylase has a key role in the physiology of adrenergic neurons.[supplied by OMIM] |
| Alias | TYH |
|  | Tyrosine 3-hydroxylase |
|  | TH |
|  | tyrosine hydroxylase (AA 1-30) |
|  | Th |
|  | The |


---

|  |  |
| --- | --- |
| GO Component | nucleus |
|  | cytoplasm |


---

|  |  |
| --- | --- |
| GO ID | GO:0009072 |
|  | GO:0042423 |
|  | GO:0005506 |
|  | GO:0004511 |
|  | GO:0006950 |
|  | GO:0005737 |
|  | GO:0008016 |
|  | GO:0016491 |
|  | GO:0042136 |
|  | GO:0009653 |
|  | GO:0005634 |
|  | GO:0007507 |
|  | GO:0006585 |
|  | GO:0009887 |
|  | GO:0046872 |
|  | GO:0007268 |
|  | GO:0004497 |


---

|  |  |
| --- | --- |
| MIM | MIM:191290 |


---

|  |  |
| --- | --- |
| Connectivity | 1381 |


---

|  |  |
| --- | --- |
| Entrez ID | 25085 |
|  | 7054 |
|  | 21823 |


---

|  |  |
| --- | --- |
| Agilent ID | A\_23\_P258633 |
|  | A\_53\_P144971 |
|  | A\_53\_P160841 |
|  | A\_53\_P132982 |
|  | A\_14\_P136904 |
|  | A\_44\_P301729 |
|  | A\_43\_P11541 |
|  | A\_23\_P95891 |
|  | A\_53\_P119384 |
|  | A\_24\_P924602 |
|  | A\_51\_P486223 |


---

|  |  |
| --- | --- |
| Cellular Localization | Cytoplasm |
|  | Nucleus |
|  | Cell |
|  | Organelle |


---

|  |  |
| --- | --- |
| DbXref | KEGG pathway##00350##Tyrosine metabolism##http://www.genome.jp/dbget-bin/show\_pathway?rno00350+25085 |
|  | KEGG pathway##05020##Parkinson's disease##http://www.genome.jp/dbget-bin/show\_pathway?hsa05020+7054 |
|  | KEGG pathway##05020##Parkinson's disease##http://www.genome.jp/dbget-bin/show\_pathway?mmu05020+21823 |
|  | KEGG pathway##05020##Parkinson's disease##http://www.genome.jp/dbget-bin/show\_pathway?rno05020+25085 |
|  | KEGG pathway##00350##Tyrosine metabolism##http://www.genome.jp/dbget-bin/show\_pathway?mmu00350+21823 |
|  | KEGG pathway##00350##Tyrosine metabolism##http://www.genome.jp/dbget-bin/show\_pathway?hsa00350+7054 |


---

|  |  |
| --- | --- |
| Pathway | Zn def RIN |
|  | Zn def DIN |


---

|  |  |
| --- | --- |
| GO Process | regulation of heart contraction rate |
|  | response to stress |
|  | dopamine biosynthesis from tyrosine |
|  | catecholamine biosynthesis |
|  | aromatic amino acid family metabolism |
|  | organogenesis |
|  | organ morphogenesis |
|  | synaptic transmission |
|  | neurotransmitter biosynthesis |
|  | heart development |
|  | morphogenesis |


---

|  |  |
| --- | --- |
| UniGene | Mm.1292 |
|  | Hs.435609 |
|  | Rn.11082 |


---

|  |  |
| --- | --- |
| Affymetrix Probeset ID | 100690\_at |
|  | 1373443\_a\_at |
|  | 1387075\_at |
|  | 1420546\_at |
|  | 208291\_s\_at |
|  | 32300\_s\_at |
|  | 1391766\_at |
|  | g4507480\_3p\_a\_at |
|  | M10244\_at |
|  | M69200\_s\_at |
|  | rc\_AI104389\_g\_at |
|  | Y00414\_s\_at |
|  | RC\_AA447751\_s\_at |
|  | rc\_AI059946\_at |


---

|  |  |
| --- | --- |
| EC Number | EC 1.14.16.2 |


---

|  |  |
| --- | --- |
| GO Function | tyrosine 3-monooxygenase activity |
|  | oxidoreductase activity |
|  | monooxygenase activity |
|  | iron ion binding |
|  | metal ion binding |


---

|  |  |
| --- | --- |
| Nucleotide | X04914 |
|  | NM\_199293 |
|  | M69200 |
|  | AY144495 |
|  | NM\_199292 |
|  | M24791 |
|  | M23597 |
|  | M24790 |
|  | X53503 |
|  | M17589 |
|  | AY144494 |
|  | AF415235 |
|  | NM\_000360 |
|  | AF536811 |
|  | AY211521 |
|  | M20912 |
|  | NM\_012740 |
|  | Y00414 |
|  | M23598 |
|  | AK139568 |
|  | U62763 |
|  | M10244 |
|  | X05290 |
|  | NM\_009377 |
|  | M18116 |
|  | M20911 |
|  | L22651 |


---

|  |  |
| --- | --- |
| Protein | P24529 |
|  | CAA28908 |
|  | CAA68472 |
|  | AAN39538 |
|  | NP\_000351 |
|  | AAP43671 |
|  | AAN73290 |
|  | AAA77649 |
|  | AAA61168 |
|  | AAA77650 |
|  | AAA61174 |
|  | AAA40434 |
|  | BAE24067 |
|  | AAA42257 |
|  | NP\_954987 |
|  | NP\_036872 |
|  | AAB59722 |
|  | AAN73289 |
|  | AAA61170 |
|  | CAA28584 |
|  | NP\_033403 |
|  | AAA61172 |
|  | AAL09025 |
|  | P04177 |
|  | AAA61167 |
|  | P07101 |
|  | AAA42258 |
|  | AAA77651 |
|  | AAA61173 |
|  | AAN39539 |
|  | NP\_954986 |
|  | AAA61179 |
|  | AAA61171 |
|  | CAA37580 |


---

|  |  |
| --- | --- |
| Organism | Mammal |


---

|  |  |
| --- | --- |
| Location | 7 69.2 cM (Mus musculus) |
|  | chromosome 7, 7 69.2 cM, 7 F5 (Mus musculus) |
|  | chromosome 1, 1q41 (Rattus norvegicus) |
|  | chromosome 11, 11p15.5 (Homo sapiens) |


---

|  |  |
| --- | --- |
