# Supplementary material for: Dynamic transcriptomic profiles of zebrafish gills in response to zinc depletion
Source: BMC Genomics. 2010 Oct 8;11:548. doi: 10.1186/1471-2164-11-548 (PMC3091697; doi:10.1186/1471-2164-11-548)
Supplement: Additional file 2 — Figure S1 - Interactive Direct Interaction Network of responses to zinc depletion. Mini web-site containing index.html and hyperlinked pages in subdirectory. The web site is an interactive version of Figure 6A containing curated interactions between regulated genes and respective proteins. Legend: Molecular interactions between zinc and proteins encoded by genes changed under zinc depletion. A Direct Interaction Network was created based on curated interactions contained within the PathwayArchitect database and provided through hyperlinks. Red ovals represent proteins and the blue circle symbolizes Zn(II). Dark blue squares denote 'binding', and light blue squares 'expression'; green squares stand for 'regulation', green diamonds for 'metabolism', and green circles for 'promoter binding'. Arrow heads indicate directionality of the interaction where annotated. [file 1471-2164-11-548-S2.ZIP › PathwayArchitect Zn def DIN2/80691.html]

# PROTEIN: DUSP7

|  |  |
| --- | --- |
| Name | DUSP7 |
| Type | PROTEIN |
| Description | dual specificity phosphatase 7 |
| Note | Protein phosphatase; suppresses MAP kinase activation state [RGD] |
| Alias | Dual-specificity phosphatase-7 |
|  | Mkpx |
|  | AU015694 |
|  | Dual specificity protein phosphatase MKP-X |
|  | MKPX |
|  | Dual specificity protein phosphatase PYST2 |
|  | mkp-X |
|  | PYST2 |
|  | MAP kinase phosphatase |
|  | MKP-X |
|  | Fragment |
|  | Dusp7 |
|  | DUSP7 |


---

|  |  |
| --- | --- |
| GO Component | cytosol |
|  | cellular component unknown |


---

|  |  |
| --- | --- |
| GO ID | GO:0004725 |
|  | GO:0000004 |
|  | GO:0004722 |
|  | GO:0008372 |
|  | GO:0017017 |
|  | GO:0016787 |
|  | GO:0004721 |
|  | GO:0005554 |
|  | GO:0008138 |
|  | GO:0005829 |
|  | GO:0006470 |


---

|  |  |
| --- | --- |
| MIM | MIM:602749 |


---

|  |  |
| --- | --- |
| Connectivity | 16 |


---

|  |  |
| --- | --- |
| Entrez ID | 235584 |
|  | 300980 |
|  | 1849 |


---

|  |  |
| --- | --- |
| Agilent ID | A\_51\_P266043 |
|  | A\_52\_P173197 |
|  | A\_43\_P15887 |
|  | A\_53\_P169554 |
|  | A\_24\_P51855 |
|  | A\_44\_P463702 |
|  | A\_53\_P177307 |


---

|  |  |
| --- | --- |
| Cellular Localization | Cytosol |
|  | Cytoplasm |
|  | Cell |


---

|  |  |
| --- | --- |
| DbXref | KEGG pathway##04010##MAPK signaling pathway##http://www.genome.jp/dbget-bin/show\_pathway?mmu04010+235584 |
|  | KEGG pathway##04010##MAPK signaling pathway##http://www.genome.jp/dbget-bin/show\_pathway?rno04010+300980 |
|  | KEGG pathway##04010##MAPK signaling pathway##http://www.genome.jp/dbget-bin/show\_pathway?hsa04010+1849 |


---

|  |  |
| --- | --- |
| Pathway | Zn def RIN |
|  | Zn def DIN |


---

|  |  |
| --- | --- |
| GO Process | biological process unknown |
|  | protein amino acid dephosphorylation |


---

|  |  |
| --- | --- |
| UniGene | Hs.3843 |
|  | Rn.141044 |
|  | Mm.275584 |
|  | Rn.104502 |


---

|  |  |
| --- | --- |
| Affymetrix Probeset ID | 108056\_at |
|  | 1427416\_x\_at |
|  | 1452097\_a\_at |
|  | 1460393\_a\_at |
|  | 213848\_at |
|  | 214793\_at |
|  | 39932\_at |
|  | 39933\_at |
|  | 1377992\_at |
|  | Hs.296938.0.S1\_3p\_at |
|  | Hs.3843.0.A1\_3p\_at |
|  | Hs.3843.0.A1\_3p\_x\_at |
|  | Msa.16748.0\_s\_at |
|  | X93921\_at |
|  | X94186\_at |
|  | TC15711\_s\_at |
|  | TC36167\_at |


---

|  |  |
| --- | --- |
| EC Number | EC 3.1.3.16 |
|  | EC 3.1.3.48 |


---

|  |  |
| --- | --- |
| GO Function | MAP kinase phosphatase activity |
|  | hydrolase activity |
|  | protein tyrosine phosphatase activity |
|  | protein tyrosine/serine/threonine phosphatase activity |
|  | phosphoprotein phosphatase activity |
|  | protein serine/threonine phosphatase activity |
|  | molecular function unknown |


---

|  |  |
| --- | --- |
| Nucleotide | BC025048 |
|  | AK158817 |
|  | XM\_238551 |
|  | X93921 |
|  | NM\_001947 |
|  | AK140372 |
|  | AK211556 |
|  | AF508727 |
|  | BC019107 |
|  | X94186 |
|  | BC010207 |
|  | NM\_153459 |


---

|  |  |
| --- | --- |
| Protein | AAH10207 |
|  | NP\_001938 |
|  | AAH25048 |
|  | CAA63814 |
|  | XP\_238551 |
|  | Q16829 |
|  | AAH19107 |
|  | NP\_703189 |
|  | AAM77606 |
|  | Q91Z46 |
|  | CAA63896 |
|  | BAE24360 |
|  | Q63340 |
|  | BAE34680 |


---

|  |  |
| --- | --- |
| Organism | Mammal |


---

|  |  |
| --- | --- |
| Location | chromosome 3, 3p21 (Homo sapiens) |
|  | chromosome 9, 9 F1 (Mus musculus) |
|  | chromosome 8, 8q32 (Rattus norvegicus) |


---

|  |  |
| --- | --- |
