# Supplementary material for: Dynamic transcriptomic profiles of zebrafish gills in response to zinc depletion
Source: BMC Genomics. 2010 Oct 8;11:548. doi: 10.1186/1471-2164-11-548 (PMC3091697; doi:10.1186/1471-2164-11-548)
Supplement: Additional file 2 — Figure S1 - Interactive Direct Interaction Network of responses to zinc depletion. Mini web-site containing index.html and hyperlinked pages in subdirectory. The web site is an interactive version of Figure 6A containing curated interactions between regulated genes and respective proteins. Legend: Molecular interactions between zinc and proteins encoded by genes changed under zinc depletion. A Direct Interaction Network was created based on curated interactions contained within the PathwayArchitect database and provided through hyperlinks. Red ovals represent proteins and the blue circle symbolizes Zn(II). Dark blue squares denote 'binding', and light blue squares 'expression'; green squares stand for 'regulation', green diamonds for 'metabolism', and green circles for 'promoter binding'. Arrow heads indicate directionality of the interaction where annotated. [file 1471-2164-11-548-S2.ZIP › PathwayArchitect Zn def DIN2/845008.html]

# REGULATION:

|  |  |
| --- | --- |
| Type | REGULATION |
| Effect | Negative |


---

|  |  |
| --- | --- |
| Score | 0 |


---

|  |  |
| --- | --- |
| Reference Count | 4 |


---

|  |  |
| --- | --- |
| Mechanism | Unknown |


---

|  |  |
| --- | --- |
| Reference:0 || Sentence | "Cupric ions replaced Zn in MT after being reduced by thiol groups, and MT, worked as an antioxidant." |
| PMID | 8917695 |
| Year | 1996 |
| Species | Rat |
| Journal | Toxicol Appl Pharmacol |
| RefScore | 1 |
| Source | PArchNLP |
  |
|  |  |


---

|  |  |
| --- | --- |
 Reference:1 || Sentence | "A part of the liver zinc, however, was bound to newly synthesized MT to prevent greater zinc loss." |
| PMID | 8155985 |
| Year | 1993 |
| Species | Rat |
| Journal | J Trace Elem Electrolytes Health Dis |
| RefScore | 1 |
| Source | PArchNLP |
  ||  |  |


---

|  |  |
| --- | --- |
 Reference:2 || Sentence | "Thus the rapid degradation of hepatic MT in zinc deficiency that may occur to maintain the required zinc levels in other tissues supports the role of MT as a zinc storage protein in newborn rats." |
| PMID | 3585520 |
| Year | 1987 |
| Species | Rat |
| Journal | J Nutr |
| RefScore | 1 |
| Source | PArchNLP |
  ||  |  |


---

|  |  |
| --- | --- |
 Reference:3 || Sentence | "The results showed that antisense MT oligonucleotide markedly decreased the resistance of osteoblast to zinc excess and zinc deficiency." |
| PMID | 11321951 |
| Year | 2001 |
| Species | Rat |
| Journal | Wei Sheng Yan Jiu |
| RefScore | 1 |
| Source | PArchNLP |
  |


---

|  |  |
| --- | --- |
