# Supplementary material for: Dynamic transcriptomic profiles of zebrafish gills in response to zinc depletion
Source: BMC Genomics. 2010 Oct 8;11:548. doi: 10.1186/1471-2164-11-548 (PMC3091697; doi:10.1186/1471-2164-11-548)
Supplement: Additional file 2 — Figure S1 - Interactive Direct Interaction Network of responses to zinc depletion. Mini web-site containing index.html and hyperlinked pages in subdirectory. The web site is an interactive version of Figure 6A containing curated interactions between regulated genes and respective proteins. Legend: Molecular interactions between zinc and proteins encoded by genes changed under zinc depletion. A Direct Interaction Network was created based on curated interactions contained within the PathwayArchitect database and provided through hyperlinks. Red ovals represent proteins and the blue circle symbolizes Zn(II). Dark blue squares denote 'binding', and light blue squares 'expression'; green squares stand for 'regulation', green diamonds for 'metabolism', and green circles for 'promoter binding'. Arrow heads indicate directionality of the interaction where annotated. [file 1471-2164-11-548-S2.ZIP › PathwayArchitect Zn def DIN2/85243.html]

# PROTEIN: SLC39A3

|  |  |
| --- | --- |
| Name | SLC39A3 |
| Type | PROTEIN |
| Description | solute carrier family 39 (zinc transporter), member 3 |
| Alias | MGC7461 |
|  | Zip3 |
|  | ZIP3 |
|  | AI845814 |
|  | zinc transporter |
|  | MGC109671 |


---

|  |  |
| --- | --- |
| GO Component | extracellular space |
|  | integral to membrane |
|  | membrane |


---

|  |  |
| --- | --- |
| GO ID | GO:0016020 |
|  | GO:0046873 |
|  | GO:0005615 |
|  | GO:0016021 |
|  | GO:0030001 |
|  | GO:0005385 |
|  | GO:0006829 |


---

|  |  |
| --- | --- |
| Connectivity | 16 |


---

|  |  |
| --- | --- |
| Entrez ID | 29985 |
|  | 106947 |


---

|  |  |
| --- | --- |
| Group | Extracellular proteins |


---

|  |  |
| --- | --- |
| Agilent ID | A\_23\_P388170 |
|  | A\_53\_P172086 |
|  | A\_14\_P102028 |
|  | A\_53\_P126428 |
|  | A\_24\_P382467 |
|  | A\_51\_P387253 |
|  | A\_53\_P179213 |
|  | A\_51\_P387248 |
|  | A\_24\_P160460 |
|  | A\_14\_P118089 |


---

|  |  |
| --- | --- |
| Cellular Localization | Membrane |
|  | Cell |
|  | Extracellular region |


---

|  |  |
| --- | --- |
| Pathway | MTF1 pathway NLP-enriched |
|  | Master Regulators |
|  | Zn def RIN |
|  | Zn def DIN |


---

|  |  |
| --- | --- |
| GO Process | zinc ion transport |
|  | metal ion transport |


---

|  |  |
| --- | --- |
| UniGene | Hs.515046 |
|  | Mm.5353 |


---

|  |  |
| --- | --- |
| Affymetrix Probeset ID | 1417881\_at |
|  | 1417882\_at |
|  | 165796\_at |
|  | 223917\_s\_at |
|  | 229677\_at |
|  | 232115\_at |
|  | 232713\_3p\_at |
|  | 232713\_at |
|  | 53046\_r\_at |
|  | 53333\_at |
|  | 59542\_at |
|  | 93173\_at |
|  | D18934\_rc\_at |
|  | g12652672\_3p\_a\_at |
|  | Hs.174308.0.S1\_3p\_at |
|  | Hs.306422.0.S1\_3p\_at |
|  | Hs.306422.0.S1\_3p\_x\_at |
|  | Hs.30901.0.S1\_3p\_at |
|  | Hs.30901.0.S1\_3p\_s\_at |
|  | 130464\_at |
|  | RC\_AA350268\_at |
|  | RC\_AA432197\_f\_at |
|  | RC\_AA450097\_f\_at |
|  | RC\_H05464\_at |
|  | RC\_N59534\_f\_at |
|  | RC\_N59534\_r\_at |
|  | RC\_N67815\_f\_at |
|  | RC\_R49689\_f\_at |
|  | RC\_T23724\_f\_at |
|  | TC41152\_at |


---

|  |  |
| --- | --- |
| GO Function | metal ion transporter activity |
|  | zinc ion transporter activity |


---

|  |  |
| --- | --- |
| Nucleotide | AK087519 |
|  | NM\_213568 |
|  | BG719479 |
|  | BQ898834 |
|  | BC085175 |
|  | AK002044 |
|  | BC020571 |
|  | BC005869 |
|  | NM\_134135 |
|  | BC005502 |
|  | AF052125 |
|  | BC017009 |
|  | NM\_144564 |


---

|  |  |
| --- | --- |
| Protein | AAH20571 |
|  | NP\_998733 |
|  | AAH05869 |
|  | NP\_653165 |
|  | NP\_598896 |
|  | AAH05502 |


---

|  |  |
| --- | --- |
| Organism | Mammal |


---

|  |  |
| --- | --- |
| Location | chromosome 19, 19p13.3 (Homo sapiens) |
|  | chromosome 10, 10 C1 (Mus musculus) |


---

|  |  |
| --- | --- |
