# Supplementary material for: Dynamic transcriptomic profiles of zebrafish gills in response to zinc depletion
Source: BMC Genomics. 2010 Oct 8;11:548. doi: 10.1186/1471-2164-11-548 (PMC3091697; doi:10.1186/1471-2164-11-548)
Supplement: Additional file 2 — Figure S1 - Interactive Direct Interaction Network of responses to zinc depletion. Mini web-site containing index.html and hyperlinked pages in subdirectory. The web site is an interactive version of Figure 6A containing curated interactions between regulated genes and respective proteins. Legend: Molecular interactions between zinc and proteins encoded by genes changed under zinc depletion. A Direct Interaction Network was created based on curated interactions contained within the PathwayArchitect database and provided through hyperlinks. Red ovals represent proteins and the blue circle symbolizes Zn(II). Dark blue squares denote 'binding', and light blue squares 'expression'; green squares stand for 'regulation', green diamonds for 'metabolism', and green circles for 'promoter binding'. Arrow heads indicate directionality of the interaction where annotated. [file 1471-2164-11-548-S2.ZIP › PathwayArchitect Zn def DIN2/85841.html]

# PROTEIN: NR5A2

|  |  |
| --- | --- |
| Name | NR5A2 |
| Type | PROTEIN |
| Description | nuclear receptor subfamily 5, group A, member 2 |
| Note | An orphan nuclear receptor that may bind DNA and activate gene transcription [RGD] |
| Alias | FTZ-F1 beta1 protein |
|  | Alpha-1-fetoprotein transcription factor |
|  | FTF |
|  | FTZ-F1 beta2 protein |
|  | nuclear receptor NR5A2 |
|  | B1-binding factor |
|  | B1F2 |
|  | FTZ-F1beta |
|  | Lrh1 |
|  | Nr5a2 |
|  | Lrh-1 |
|  | CYP7A promoter-binding factor |
|  | NR5A2 |
|  | liver receptor homolog 1 |
|  | b1-binding factor, hepatocyte transcription factor which activates enhancer II of hepatitis B virus |
|  | Liver receptor homolog |
|  | hB1F |
|  | Hepatocytic transcription factor |
|  | Ftf |
|  | hB1F-2 |
|  | B1F |
|  | LRH-1 |
|  | CYP7A promoter binding factor |
|  | fetoprotein-alpha 1 (AFP) transcription factor |
|  | D1Ertd308e |
|  | fetoprotein transcription factor |
|  | FTZ-F1 |
|  | FTZ-F1 beta |
|  | CPF |


---

|  |  |
| --- | --- |
| GO Component | transcription factor complex |
|  | nucleus |


---

|  |  |
| --- | --- |
| GO ID | GO:0003707 |
|  | GO:0003677 |
|  | GO:0006355 |
|  | GO:0003705 |
|  | GO:0005667 |
|  | GO:0042632 |
|  | GO:0003700 |
|  | GO:0009653 |
|  | GO:0005634 |
|  | GO:0004872 |
|  | GO:0016563 |
|  | GO:0004879 |
|  | GO:0006366 |
|  | GO:0046872 |
|  | GO:0006350 |
|  | GO:0008206 |


---

|  |  |
| --- | --- |
| MIM | MIM:604453 |


---

|  |  |
| --- | --- |
| Connectivity | 648 |


---

|  |  |
| --- | --- |
| Entrez ID | 60349 |
|  | 2494 |
|  | 26424 |


---

|  |  |
| --- | --- |
| Agilent ID | A\_14\_P202308 |
|  | A\_53\_P119561 |
|  | A\_43\_P12110 |
|  | A\_14\_P131751 |
|  | A\_24\_P920090 |
|  | A\_14\_P134233 |
|  | A\_14\_P109851 |
|  | A\_51\_P514449 |
|  | A\_23\_P160996 |
|  | A\_32\_P863837 |
|  | A\_14\_P116960 |
|  | A\_23\_P161004 |
|  | A\_53\_P141840 |
|  | A\_14\_P126842 |
|  | A\_23\_P993 |


---

|  |  |
| --- | --- |
| Cellular Localization | Nucleus |
|  | Organelle |
|  | Cell |


---

|  |  |
| --- | --- |
| Pathway | Zn def RIN |
|  | Master Regulators |
|  | Zn def DIN |


---

|  |  |
| --- | --- |
| GO Process | cholesterol homeostasis |
|  | bile acid metabolism |
|  | regulation of transcription, DNA-dependent |
|  | transcription |
|  | transcription from RNA polymerase II promoter |
|  | morphogenesis |


---

|  |  |
| --- | --- |
| UniGene | Mm.16794 |
|  | Rn.42941 |
|  | Hs.33446 |


---

|  |  |
| --- | --- |
| Affymetrix Probeset ID | 102932\_at |
|  | 115599\_at |
|  | 1387573\_a\_at |
|  | 1420410\_at |
|  | 1449706\_s\_at |
|  | 1449707\_at |
|  | 1560469\_at |
|  | 208337\_s\_at |
|  | 208343\_s\_at |
|  | 210174\_at |
|  | 35865\_at |
|  | 45918\_at |
|  | 59241\_at |
|  | 89703\_at |
|  | 90139\_at |
|  | AB012960\_s\_at |
|  | c80532\_rc\_at |
|  | g4504342\_3p\_a\_at |
|  | g5002241\_3p\_a\_at |
|  | Hs.183123.1.S4\_3p\_at |
|  | Hs2.385611.1.S1\_3p\_at |
|  | Msa.911.0\_at |
|  | U93553\_at |
|  | RC\_C20936\_at |
|  | RC\_N53560\_at |
|  | RC\_R10138\_at |
|  | c77555\_rc\_at |


---

|  |  |
| --- | --- |
| GO Function | ligand-dependent nuclear receptor activity |
|  | RNA polymerase II transcription factor activity, enhancer binding |
|  | DNA binding |
|  | transcriptional activator activity |
|  | transcription factor activity |
|  | steroid hormone receptor activity |
|  | receptor activity |
|  | metal ion binding |


---

|  |  |
| --- | --- |
| Nucleotide | AB019246 |
|  | BC027893 |
|  | C80272 |
|  | U80251 |
|  | AF228413 |
|  | AB012961 |
|  | AF124247 |
|  | NM\_030676 |
|  | AF190464 |
|  | NM\_003822 |
|  | NM\_205860 |
|  | AB012960 |
|  | M81385 |
|  | AF239709 |
|  | AK054487 |
|  | U47280 |
|  | AF049102 |
|  | NM\_021742 |
|  | AF319643 |
|  | CA776772 |
|  | CB162803 |
|  | U93553 |
|  | AF146343 |


---

|  |  |
| --- | --- |
| Protein | AAL37171 |
|  | AAC78727 |
|  | BAA34092 |
|  | NP\_068510 |
|  | BAA36339 |
|  | BAA36340 |
|  | NP\_995582 |
|  | AAG17125 |
|  | P45448 |
|  | AAA39447 |
|  | AAD03155 |
|  | AAC52645 |
|  | AAG17124 |
|  | AAD37378 |
|  | NP\_109601 |
|  | AAD03248 |
|  | NP\_003813 |
|  | O00482 |
|  | AAD26565 |
|  | AAK00806 |


---

|  |  |
| --- | --- |
| Organism | Mammal |


---

|  |  |
| --- | --- |
| Location | 1 78.0 cM (Mus musculus) |
|  | chromosome 13, 13q13 (Rattus norvegicus) |
|  | chromosome 1, 1 78.0 cM, 1 E4 (Mus musculus) |
|  | chromosome 1, 1q32.1 (Homo sapiens) |


---

|  |  |
| --- | --- |
