# Supplementary material for: Dynamic transcriptomic profiles of zebrafish gills in response to zinc depletion
Source: BMC Genomics. 2010 Oct 8;11:548. doi: 10.1186/1471-2164-11-548 (PMC3091697; doi:10.1186/1471-2164-11-548)
Supplement: Additional file 2 — Figure S1 - Interactive Direct Interaction Network of responses to zinc depletion. Mini web-site containing index.html and hyperlinked pages in subdirectory. The web site is an interactive version of Figure 6A containing curated interactions between regulated genes and respective proteins. Legend: Molecular interactions between zinc and proteins encoded by genes changed under zinc depletion. A Direct Interaction Network was created based on curated interactions contained within the PathwayArchitect database and provided through hyperlinks. Red ovals represent proteins and the blue circle symbolizes Zn(II). Dark blue squares denote 'binding', and light blue squares 'expression'; green squares stand for 'regulation', green diamonds for 'metabolism', and green circles for 'promoter binding'. Arrow heads indicate directionality of the interaction where annotated. [file 1471-2164-11-548-S2.ZIP › PathwayArchitect Zn def DIN2/87175.html]

# PROTEIN: SLC7A9

|  |  |
| --- | --- |
| Name | SLC7A9 |
| Type | PROTEIN |
| Description | solute carrier family 7 (cationic amino acid transporter, y+ system), member 9 |
| Note | Amino acid transporter protein; exhibits high-affinity sodium-independent transport of cystine; may mediate reabsorption of cystine in the kidney proximal tubule [RGD] |
| Alias | Slc7a9 |
|  | glycoprotein-associated amino acid transporter b0,+AT1 |
|  | b, +AT |
|  | solute carrier family 7, member 9 |
|  | B(0,+)AT |
|  | CSNU3 |
|  | Bat1 |
|  | b, + amino acid transporter |
|  | MGC114282 |
|  | Glycoprotein- associated amino acid transporter b0,+AT1 |


---

|  |  |
| --- | --- |
| GO Component | integral to membrane |
|  | plasma membrane |
|  | integral to plasma membrane |
|  | membrane |


---

|  |  |
| --- | --- |
| GO ID | GO:0006810 |
|  | GO:0016020 |
|  | GO:0006520 |
|  | GO:0006865 |
|  | GO:0005887 |
|  | GO:0016021 |
|  | GO:0005279 |
|  | GO:0015184 |
|  | GO:0005886 |
|  | GO:0006461 |
|  | GO:0015171 |


---

|  |  |
| --- | --- |
| MIM | MIM:604144 |


---

|  |  |
| --- | --- |
| Connectivity | 12 |


---

|  |  |
| --- | --- |
| Entrez ID | 30962 |
|  | 11136 |
|  | 116726 |


---

|  |  |
| --- | --- |
| Agilent ID | A\_51\_P223203 |
|  | A\_42\_P509967 |
|  | A\_53\_P127792 |
|  | A\_52\_P474145 |
|  | A\_53\_P119957 |
|  | A\_14\_P111477 |
|  | A\_14\_P131740 |
|  | A\_23\_P142250 |
|  | A\_14\_P132195 |
|  | A\_53\_P153789 |
|  | A\_14\_P111534 |


---

|  |  |
| --- | --- |
| Cellular Localization | Membrane |
|  | Plasma membrane |
|  | Cell |


---

|  |  |
| --- | --- |
| Pathway | Zn def RIN |
|  | Master Regulators |
|  | Zn def DIN |


---

|  |  |
| --- | --- |
| GO Process | amino acid metabolism |
|  | amino acid transport |
|  | transport |
|  | protein complex assembly |


---

|  |  |
| --- | --- |
| UniGene | Hs.408567 |
|  | Mm.45874 |
|  | Rn.32225 |


---

|  |  |
| --- | --- |
| Affymetrix Probeset ID | 1368727\_at |
|  | 1448783\_at |
|  | 220135\_s\_at |
|  | 85677\_r\_at |
|  | 89096\_at |
|  | 92699\_at |
|  | g7657590\_3p\_a\_at |
|  | TC17589\_at |
|  | TC17589\_g\_at |
|  | TC38192\_at |


---

|  |  |
| --- | --- |
| GO Function | amino acid transporter activity |
|  | L-cystine transporter activity |
|  | amino acid-polyamine transporter activity |


---

|  |  |
| --- | --- |
| Nucleotide | NM\_053929 |
|  | AF421181 |
|  | AB029559 |
|  | BC017962 |
|  | BC029802 |
|  | AJ249199 |
|  | AF192310 |
|  | AJ249198 |
|  | NM\_021291 |
|  | AF141289 |
|  | BC098909 |
|  | AK223147 |
|  | AK026446 |
|  | AK164714 |
|  | AK142541 |
|  | BC081750 |
|  | BC010746 |
|  | AB033548 |
|  | NM\_014270 |


---

|  |  |
| --- | --- |
| Protein | AAH17962 |
|  | BAB16840 |
|  | AAH10746 |
|  | BAD96867 |
|  | AAG28396 |
|  | NP\_067266 |
|  | P82252 |
|  | BAE25099 |
|  | P82251 |
|  | NP\_055085 |
|  | CAB54042 |
|  | AAN40878 |
|  | CAB54003 |
|  | Q9QXA6 |
|  | AAH98909 |
|  | NP\_446381 |
|  | BAA85186 |
|  | AAD55898 |


---

|  |  |
| --- | --- |
| Organism | Mammal |


---

|  |  |
| --- | --- |
| Location | chromosome 1, 1q21 (Rattus norvegicus) |
|  | chromosome 7, 7 B1 (Mus musculus) |
|  | chromosome 19, 19q13.1 (Homo sapiens) |


---

|  |  |
| --- | --- |
