# Supplementary material for: Dynamic transcriptomic profiles of zebrafish gills in response to zinc depletion
Source: BMC Genomics. 2010 Oct 8;11:548. doi: 10.1186/1471-2164-11-548 (PMC3091697; doi:10.1186/1471-2164-11-548)
Supplement: Additional file 2 — Figure S1 - Interactive Direct Interaction Network of responses to zinc depletion. Mini web-site containing index.html and hyperlinked pages in subdirectory. The web site is an interactive version of Figure 6A containing curated interactions between regulated genes and respective proteins. Legend: Molecular interactions between zinc and proteins encoded by genes changed under zinc depletion. A Direct Interaction Network was created based on curated interactions contained within the PathwayArchitect database and provided through hyperlinks. Red ovals represent proteins and the blue circle symbolizes Zn(II). Dark blue squares denote 'binding', and light blue squares 'expression'; green squares stand for 'regulation', green diamonds for 'metabolism', and green circles for 'promoter binding'. Arrow heads indicate directionality of the interaction where annotated. [file 1471-2164-11-548-S2.ZIP › PathwayArchitect Zn def DIN2/87454.html]

# PROTEIN: HIST1H4B

|  |  |
| --- | --- |
| Name | HIST1H4B |
| Type | PROTEIN |
| Description | histone 1, H4b |
| Note | Histones are basic nuclear proteins that are responsible for the nucleosome structure of the chromosomal fiber in eukaryotes. Two molecules of each of the four core histones (H2A, H2B, H3, and H4) form an octamer, around which approximately 146 bp of DNA is wrapped in repeating units, called nucleosomes. The linker histone, H1, interacts with linker DNA between nucleosomes and functions in the compaction of chromatin into higher order structures. This gene is intronless and encodes a member of the histone H4 family. Transcripts from this gene lack polyA tails but instead contain a palindromic termination element. This gene is found in the large histone gene cluster on chromosome 6. |
| Alias | histone H4 |
|  | H4ft |
|  | H1ft |
|  | H4 histone family, member I |
|  | H4/I |
|  | H4FI |
|  | germinal histone H4 gene |
|  | Hist4 |
|  | Histone H4 |


---

|  |  |
| --- | --- |
| GO Component | chromosome |
|  | nucleus |
|  | nucleosome |


---

|  |  |
| --- | --- |
| GO ID | GO:0005634 |
|  | GO:0003677 |
|  | GO:0006334 |
|  | GO:0007001 |
|  | GO:0005694 |
|  | GO:0000786 |


---

|  |  |
| --- | --- |
| MIM | MIM:602829 |


---

|  |  |
| --- | --- |
| Connectivity | 42 |


---

|  |  |
| --- | --- |
| Entrez ID | 64627 |
|  | 8366 |
|  | 326620 |


---

|  |  |
| --- | --- |
| Agilent ID | A\_43\_P15563 |
|  | A\_42\_P532700 |
|  | A\_44\_P742272 |
|  | A\_24\_P166407 |
|  | A\_23\_P81850 |
|  | A\_44\_P552794 |


---

|  |  |
| --- | --- |
| Cellular Localization | Nucleus |
|  | Chromosome |
|  | Organelle |
|  | Cell |


---

|  |  |
| --- | --- |
| Pathway | Zn def RIN |
|  | Master Regulators |
|  | Zn def DIN |


---

|  |  |
| --- | --- |
| GO Process | nucleosome assembly |
|  | chromosome organization and biogenesis (sensu Eukaryota) |


---

|  |  |
| --- | --- |
| UniGene | Mm.261642 |
|  | Hs.143080 |
|  | Rn.1659 |


---

|  |  |
| --- | --- |
| Affymetrix Probeset ID | 1422948\_s\_at |
|  | 214516\_at |
|  | 33049\_at |
|  | 1385402\_at |
|  | Hs.143080.0.S1\_3p\_at |
|  | 165373\_at |
|  | 1369728\_at |
|  | M27433\_at |
|  | X67081\_at |
|  | 1372726\_at |
|  | 1374600\_at |
|  | AA848573\_at |
|  | rc\_AI176792\_at |
|  | 1422947\_at |
|  | Msa.2339.0\_at |


---

|  |  |
| --- | --- |
| GO Function | DNA binding |


---

|  |  |
| --- | --- |
| Nucleotide | AY128655 |
|  | NM\_003544 |
|  | NM\_022686 |
|  | AY158964 |
|  | X13236 |
|  | NM\_178193 |
|  | AL590388 |
|  | BC100619 |
|  | NG\_001335 |
|  | M28409 |
|  | AK133404 |
|  | X67081 |
|  | AY936209 |
|  | X13554 |
|  | M27433 |
|  | BC069467 |


---

|  |  |
| --- | --- |
| Protein | CAA31906 |
|  | NP\_073177 |
|  | NP\_003535 |
|  | AAA60735 |
|  | AAH69467 |
|  | P62806 |
|  | P62805 |
|  | AAA41306 |
|  | CAA31622 |
|  | AAO06274 |
|  | AAN01440 |
|  | NP\_835500 |
|  | CAA47464 |
|  | AAX28930 |
|  | AAI00620 |
|  | BAE21638 |
|  | P62804 |
|  | CAI25839 |


---

|  |  |
| --- | --- |
| Organism | Mammal |


---

|  |  |
| --- | --- |
| Location | chromosome 6, 6p21.3 (Homo sapiens) |
|  | chromosome 13, 13 A2-A3 (Mus musculus) |
|  | chromosome 17, 17p11 (Rattus norvegicus) |


---

|  |  |
| --- | --- |
