# Supplementary material for: Dynamic transcriptomic profiles of zebrafish gills in response to zinc depletion
Source: BMC Genomics. 2010 Oct 8;11:548. doi: 10.1186/1471-2164-11-548 (PMC3091697; doi:10.1186/1471-2164-11-548)
Supplement: Additional file 2 — Figure S1 - Interactive Direct Interaction Network of responses to zinc depletion. Mini web-site containing index.html and hyperlinked pages in subdirectory. The web site is an interactive version of Figure 6A containing curated interactions between regulated genes and respective proteins. Legend: Molecular interactions between zinc and proteins encoded by genes changed under zinc depletion. A Direct Interaction Network was created based on curated interactions contained within the PathwayArchitect database and provided through hyperlinks. Red ovals represent proteins and the blue circle symbolizes Zn(II). Dark blue squares denote 'binding', and light blue squares 'expression'; green squares stand for 'regulation', green diamonds for 'metabolism', and green circles for 'promoter binding'. Arrow heads indicate directionality of the interaction where annotated. [file 1471-2164-11-548-S2.ZIP › PathwayArchitect Zn def DIN2/88324.html]

# PROTEIN: ADCYAP1

|  |  |
| --- | --- |
| Name | ADCYAP1 |
| Type | PROTEIN |
| Description | adenylate cyclase activating polypeptide 1 (pituitary) |
| Note | This gene encodes adenylate cyclase activating polypeptide 1. Mediated by adenylate cyclase activating polypeptide 1 receptors, this polypeptide stimulates adenylate cyclase and subsequently increases the cAMP level in target cells. Adenylate cyclase activating polypeptide 1 is not only a hypophysiotropic hormone, but also functions as a neurotransmitter and neuromodulator. In addition, it plays a role in paracrine and autocrine regulation of certain types of cells. This gene is composed of five exons. Exons 1 and 2 encode the 5' UTR and signal peptide, respectively; exon 4 encodes an adenylate cyclase activating polypeptide 1-related peptide; and exon 5 encodes the mature peptide and 3' UTR. This gene encodes three different mature peptides, including two isotypes: a shorter form and a longer form. |
| Alias | adenylate cyclase activating polypeptide |
|  | PACAP |
|  | Pacap |
|  | rat pituitary adenylate cyclase activating polypeptide 1 |
|  | Adcyap1 |
|  | pituitary adenylate cyclase activating polypeptide 1 |
|  | pituitary adenylate cyclase-activating polypeptide precursor (Pacap) gene |


---

|  |  |
| --- | --- |
| GO Component | extracellular space |
|  | extracellular region |
|  | soluble fraction |


---

|  |  |
| --- | --- |
| GO ID | GO:0007565 |
|  | GO:0005615 |
|  | GO:0005057 |
|  | GO:0045786 |
|  | GO:0007267 |
|  | GO:0005625 |
|  | GO:0005184 |
|  | GO:0005179 |
|  | GO:0007190 |
|  | GO:0005576 |
|  | GO:0007218 |


---

|  |  |
| --- | --- |
| MIM | MIM:102980 |


---

|  |  |
| --- | --- |
| Connectivity | 255 |


---

|  |  |
| --- | --- |
| Entrez ID | 24166 |
|  | 11516 |
|  | 116 |


---

|  |  |
| --- | --- |
| Agilent ID | A\_44\_P240731 |
|  | A\_53\_P101243 |
|  | A\_51\_P110155 |
|  | A\_24\_P335781 |
|  | A\_14\_P133819 |
|  | A\_44\_P142168 |
|  | A\_14\_P132696 |
|  | A\_53\_P170247 |
|  | A\_53\_P103447 |
|  | A\_52\_P425667 |
|  | A\_44\_P522701 |
|  | A\_53\_P131583 |
|  | A\_42\_P541718 |


---

|  |  |
| --- | --- |
| Cellular Localization | Extracellular region |
|  | Cell |


---

|  |  |
| --- | --- |
| DbXref | KEGG pathway##04080##Neuroactive ligand-receptor interaction##http://www.genome.jp/dbget-bin/show\_pathway?mmu04080+11516 |
|  | KEGG pathway##04080##Neuroactive ligand-receptor interaction##http://www.genome.jp/dbget-bin/show\_pathway?hsa04080+116 |
|  | KEGG pathway##04080##Neuroactive ligand-receptor interaction##http://www.genome.jp/dbget-bin/show\_pathway?rno04080+24166 |


---

|  |  |
| --- | --- |
| Pathway | Zn def RIN |
|  | Zn def DIN |


---

|  |  |
| --- | --- |
| GO Process | neuropeptide signaling pathway |
|  | pregnancy |
|  | negative regulation of progression through cell cycle |
|  | adenylate cyclase activation |
|  | cell-cell signaling |


---

|  |  |
| --- | --- |
| UniGene | Hs.531719 |
|  | Rn.37400 |
|  | Mm.3407 |


---

|  |  |
| --- | --- |
| Affymetrix Probeset ID | 1368892\_at |
|  | 1394908\_at |
|  | 1423427\_at |
|  | 1441778\_at |
|  | 206281\_at |
|  | 230237\_at |
|  | 36754\_at |
|  | 48934\_at |
|  | 92200\_at |
|  | D14716\_s\_at |
|  | g10947062\_3p\_at |
|  | Hs.95469.0.A1\_3p\_at |
|  | M63006\_at |
|  | rc\_AI228407\_s\_at |
|  | S83513\_s\_at |
|  | X80290cds\_s\_at |
|  | RC\_AA084322\_at |


---

|  |  |
| --- | --- |
| GO Function | neuropeptide hormone activity |
|  | hormone activity |
|  | receptor signaling protein activity |


---

|  |  |
| --- | --- |
| Nucleotide | S83513 |
|  | AF252409 |
|  | NM\_001117 |
|  | AY592943 |
|  | NM\_016989 |
|  | D14716 |
|  | AK134669 |
|  | M63006 |
|  | AK142765 |
|  | AK079530 |
|  | AB010149 |
|  | X60435 |
|  | AY592948 |
|  | NM\_009625 |
|  | Z64808 |
|  | BC093837 |
|  | BC057344 |


---

|  |  |
| --- | --- |
| Protein | AAB21470 |
|  | AAH57344 |
|  | AAT92256 |
|  | NP\_058685 |
|  | BAA28355 |
|  | P13589 |
|  | AAA41791 |
|  | CAA42962 |
|  | AAH93837 |
|  | BAE22234 |
|  | BAC37673 |
|  | O70176 |
|  | NP\_001108 |
|  | AAT92261 |
|  | P18509 |
|  | NP\_033755 |


---

|  |  |
| --- | --- |
| Organism | Mammal |


---

|  |  |
| --- | --- |
| Location | chromosome 18, 18p11 (Homo sapiens) |
|  | chromosome 17, 17 E5 (Mus musculus) |
|  | chromosome 9, 9q37 (Rattus norvegicus) |


---

|  |  |
| --- | --- |
