# Supplementary material for: Dynamic transcriptomic profiles of zebrafish gills in response to zinc depletion
Source: BMC Genomics. 2010 Oct 8;11:548. doi: 10.1186/1471-2164-11-548 (PMC3091697; doi:10.1186/1471-2164-11-548)
Supplement: Additional file 2 — Figure S1 - Interactive Direct Interaction Network of responses to zinc depletion. Mini web-site containing index.html and hyperlinked pages in subdirectory. The web site is an interactive version of Figure 6A containing curated interactions between regulated genes and respective proteins. Legend: Molecular interactions between zinc and proteins encoded by genes changed under zinc depletion. A Direct Interaction Network was created based on curated interactions contained within the PathwayArchitect database and provided through hyperlinks. Red ovals represent proteins and the blue circle symbolizes Zn(II). Dark blue squares denote 'binding', and light blue squares 'expression'; green squares stand for 'regulation', green diamonds for 'metabolism', and green circles for 'promoter binding'. Arrow heads indicate directionality of the interaction where annotated. [file 1471-2164-11-548-S2.ZIP › PathwayArchitect Zn def DIN2/88858.html]

# PROTEIN: SLC39A10

|  |  |
| --- | --- |
| Name | SLC39A10 |
| Type | PROTEIN |
| Description | solute carrier family 39 (zinc transporter), member 10 |
| Note | Zinc is an essential cofactor for hundreds of enzymes. It is involved in protein, nucleic acid, carbohydrate, and lipid metabolism, as well as in the control of gene transcription, growth, development, and differentiation. SLC39A10 belongs to a subfamily of proteins that show structural characteristics of zinc transporters (Taylor and Nicholson, 2003).[supplied by OMIM] |
| Alias | LZT-Hs2 |
|  | DKFZp781L10106 |
|  | mKIAA1265 |
|  | solute carrier family 39 (metal ion transporter), member 10 |
|  | 2900042E17Rik |
|  | 5430433I10 |


---

|  |  |
| --- | --- |
| GO Component | integral to membrane |
|  | membrane |


---

|  |  |
| --- | --- |
| GO ID | GO:0016020 |
|  | GO:0046873 |
|  | GO:0016021 |
|  | GO:0030001 |


---

|  |  |
| --- | --- |
| MIM | MIM:608733 |


---

|  |  |
| --- | --- |
| Connectivity | 5 |


---

|  |  |
| --- | --- |
| Entrez ID | 57181 |
|  | 227059 |


---

|  |  |
| --- | --- |
| Agilent ID | A\_53\_P108590 |
|  | A\_51\_P223498 |
|  | A\_23\_P119778 |
|  | A\_53\_P140089 |


---

|  |  |
| --- | --- |
| Cellular Localization | Membrane |
|  | Cell |


---

|  |  |
| --- | --- |
| Pathway | Zn def RIN |
|  | Master Regulators |
|  | Zn xs inventory |
|  | Zn xs RIN |
|  | Zn xs DIN |
|  | Zn def DIN |


---

|  |  |
| --- | --- |
| GO Process | metal ion transport |


---

|  |  |
| --- | --- |
| UniGene | Mm.233889 |
|  | Hs.413434 |


---

|  |  |
| --- | --- |
| Affymetrix Probeset ID | 112853\_at |
|  | 116979\_at |
|  | 132374\_at |
|  | 1433751\_at |
|  | 1439041\_at |
|  | 225295\_at |
|  | 226444\_at |
|  | 238968\_at |
|  | 50402\_at |
|  | 51222\_at |
|  | 82788\_at |
|  | Hs.169375.0.A1\_3p\_at |
|  | Hs.24936.0.S1\_3p\_at |
|  | Hs.32793.0.A1\_3p\_at |
|  | 53024\_at |
|  | RC\_AA609943\_at |
|  | RC\_N73808\_f\_at |
|  | TC35290\_at |


---

|  |  |
| --- | --- |
| GO Function | metal ion transporter activity |


---

|  |  |
| --- | --- |
| Nucleotide | BC073909 |
|  | BC062918 |
|  | NM\_172653 |
|  | BC052880 |
|  | AK049099 |
|  | AB033091 |
|  | BC059214 |
|  | AK074996 |
|  | AK030685 |
|  | AK122483 |
|  | AL050294 |
|  | AK136117 |
|  | NM\_020342 |
|  | BC047493 |
|  | CR749813 |
|  | AK013638 |
|  | AK021167 |


---

|  |  |
| --- | --- |
| Protein | AAH62918 |
|  | AAH59214 |
|  | BAC11343 |
|  | CAH18673 |
|  | AAH73909 |
|  | NP\_766241 |
|  | CAB43393 |
|  | BAC33542 |
|  | AAH52880 |
|  | BAC27077 |
|  | BAA86579 |
|  | NP\_065075 |
|  | BAC65765 |


---

|  |  |
| --- | --- |
| Organism | Mammal |


---

|  |  |
| --- | --- |
| Location | chromosome 1, 1 C1.1 (Mus musculus) |
|  | chromosome 2, 2q32.3 (Homo sapiens) |


---

|  |  |
| --- | --- |
