# Supplementary material for: Dynamic transcriptomic profiles of zebrafish gills in response to zinc depletion
Source: BMC Genomics. 2010 Oct 8;11:548. doi: 10.1186/1471-2164-11-548 (PMC3091697; doi:10.1186/1471-2164-11-548)
Supplement: Additional file 2 — Figure S1 - Interactive Direct Interaction Network of responses to zinc depletion. Mini web-site containing index.html and hyperlinked pages in subdirectory. The web site is an interactive version of Figure 6A containing curated interactions between regulated genes and respective proteins. Legend: Molecular interactions between zinc and proteins encoded by genes changed under zinc depletion. A Direct Interaction Network was created based on curated interactions contained within the PathwayArchitect database and provided through hyperlinks. Red ovals represent proteins and the blue circle symbolizes Zn(II). Dark blue squares denote 'binding', and light blue squares 'expression'; green squares stand for 'regulation', green diamonds for 'metabolism', and green circles for 'promoter binding'. Arrow heads indicate directionality of the interaction where annotated. [file 1471-2164-11-548-S2.ZIP › PathwayArchitect Zn def DIN2/90512.html]

# PROTEIN: COX7A2L

|  |  |
| --- | --- |
| Name | COX7A2L |
| Type | PROTEIN |
| Description | cytochrome c oxidase subunit VIIa polypeptide 2 like |
| Note | Cytochrome c oxidase (COX), the terminal component of the mitochondrial respiratory chain, catalyzes the electron transfer from reduced cytochrome c to oxygen. This component is a heteromeric complex consisting of 3 catalytic subunits encoded by mitochondrial genes and multiple structural subunits encoded by nuclear genes. The mitochondrially-encoded subunits function in electron transfer, and the nuclear-encoded subunits may function in the regulation and assembly of the complex. This nuclear gene encodes a protein similar to polypeptides 1 and 2 of subunit VIIa in the C-terminal region, and also highly similar to the mouse Sig81 protein sequence. This gene is expressed in all tissues, and upregulated in a breast cancer cell line after estrogen treatment. It is possible that this gene represents a regulatory subunit of COX and mediates the higher level of energy production in target cells by estrogen. |
| Alias | EB1 |
|  | MGC103208 |
|  | Silica-induced gene 81 protein |
|  | COX7RP |
|  | cytochrome c oxidase subunit VII-related protein |
|  | Silg81 |
|  | SIG-81 |
|  | estrogen receptor binding CpG island |
|  | Cox7a2l |
|  | silica-induced gene 81 |
|  | COX7AR |
|  | Cox7rp |
|  | SIG81 |


---

|  |  |
| --- | --- |
| GO Component | mitochondrion |
|  | integral to membrane |


---

|  |  |
| --- | --- |
| GO ID | GO:0004129 |
|  | GO:0016021 |
|  | GO:0005489 |
|  | GO:0006118 |
|  | GO:0005739 |


---

|  |  |
| --- | --- |
| MIM | MIM:605771 |


---

|  |  |
| --- | --- |
| Connectivity | 11 |


---

|  |  |
| --- | --- |
| Entrez ID | 9167 |
|  | 20463 |


---

|  |  |
| --- | --- |
| Agilent ID | A\_23\_P210348 |
|  | A\_53\_P150498 |
|  | A\_53\_P109628 |
|  | A\_24\_P88800 |
|  | A\_53\_P134587 |
|  | A\_51\_P434803 |
|  | A\_53\_P162048 |
|  | A\_23\_P210343 |
|  | A\_14\_P102229 |
|  | A\_51\_P434806 |


---

|  |  |
| --- | --- |
| Cellular Localization | Mitochondrion |
|  | Cytoplasm |
|  | Membrane |
|  | Organelle |
|  | Cell |


---

|  |  |
| --- | --- |
| DbXref | KEGG pathway##00190##Oxidative phosphorylation##http://www.genome.jp/dbget-bin/show\_pathway?hsa00190+9167 |
|  | KEGG pathway##00190##Oxidative phosphorylation##http://www.genome.jp/dbget-bin/show\_pathway?mmu00190+20463 |


---

|  |  |
| --- | --- |
| Pathway | Master Regulators |
|  | Zn def RIN |
|  | Zn def DIN |


---

|  |  |
| --- | --- |
| GO Process | electron transport |


---

|  |  |
| --- | --- |
| UniGene | Mm.30072 |
|  | Hs.339639 |
|  | Mm.362119 |


---

|  |  |
| --- | --- |
| Affymetrix Probeset ID | 1421772\_a\_at |
|  | 1432263\_a\_at |
|  | 1432264\_x\_at |
|  | 160383\_at |
|  | 201256\_at |
|  | 34330\_at |
|  | 73280\_at |
|  | 83313\_at |
|  | g4758041\_3p\_at |
|  | Msa.3097.0\_f\_at |
|  | X80899\_f\_at |
|  | 96877\_at |
|  | RC\_AA025213\_at |
|  | RC\_R10896\_at |
|  | TC14444\_f\_at |
|  | TC14445\_at |
|  | TC32794\_f\_at |
|  | TC32794\_i\_at |


---

|  |  |
| --- | --- |
| GO Function | cytochrome-c oxidase activity |
|  | electron transporter activity |


---

|  |  |
| --- | --- |
| Nucleotide | AC006038 |
|  | AF127788 |
|  | AK166761 |
|  | AK216357 |
|  | AK184659 |
|  | AK216072 |
|  | BC004715 |
|  | BC085296 |
|  | NM\_009187 |
|  | AK214575 |
|  | AK150034 |
|  | NM\_004718 |
|  | AK167062 |
|  | AK217042 |
|  | AK133520 |
|  | BC005251 |
|  | AK152553 |
|  | CR542208 |
|  | AK169619 |
|  | AK130281 |
|  | AK159441 |
|  | AB007620 |
|  | AK092911 |
|  | BT007371 |
|  | AY007643 |
|  | AK009614 |
|  | X80899 |
|  | AK217377 |
|  | AB007618 |
|  | AK217840 |
|  | BC007095 |


---

|  |  |
| --- | --- |
| Protein | AAH07095 |
|  | Q61387 |
|  | BAE39224 |
|  | BAA22571 |
|  | AAY15087 |
|  | AAH85296 |
|  | AAG32129 |
|  | NP\_004709 |
|  | O14548 |
|  | BAE35087 |
|  | AAP36035 |
|  | AAF72746 |
|  | AAH05251 |
|  | BAE21701 |
|  | NP\_033213 |
|  | CAA56861 |
|  | BAE39000 |
|  | CAG47004 |
|  | AAH04715 |
|  | BAE31307 |
|  | BAE29256 |


---

|  |  |
| --- | --- |
| Organism | Mammal |


---

|  |  |
| --- | --- |
| Location | chromosome 17, 17 E4 (Mus musculus) |
|  | chromosome 2, 2p21 (Homo sapiens) |


---

|  |  |
| --- | --- |
