# Supplementary material for: Dynamic transcriptomic profiles of zebrafish gills in response to zinc depletion
Source: BMC Genomics. 2010 Oct 8;11:548. doi: 10.1186/1471-2164-11-548 (PMC3091697; doi:10.1186/1471-2164-11-548)
Supplement: Additional file 2 — Figure S1 - Interactive Direct Interaction Network of responses to zinc depletion. Mini web-site containing index.html and hyperlinked pages in subdirectory. The web site is an interactive version of Figure 6A containing curated interactions between regulated genes and respective proteins. Legend: Molecular interactions between zinc and proteins encoded by genes changed under zinc depletion. A Direct Interaction Network was created based on curated interactions contained within the PathwayArchitect database and provided through hyperlinks. Red ovals represent proteins and the blue circle symbolizes Zn(II). Dark blue squares denote 'binding', and light blue squares 'expression'; green squares stand for 'regulation', green diamonds for 'metabolism', and green circles for 'promoter binding'. Arrow heads indicate directionality of the interaction where annotated. [file 1471-2164-11-548-S2.ZIP › PathwayArchitect Zn def DIN2/91056.html]

# PROTEIN: PP

|  |  |
| --- | --- |
| Name | PP |
| Type | PROTEIN |
| Description | pyrophosphatase (inorganic) |
| Note | The protein encoded by this gene is a member of the inorganic pyrophosphatase (PPase) family. PPases catalyze the hydrolysis of pyrophosphate to inorganic phosphate, which is important for the phosphate metabolism of cells. Studies of a similar protein in bovine suggested a cytoplasmic localization of this enzyme. |
| Alias | inorganic pyrophosphatase 1 |
|  | PP1 |
|  | IOPPP |
|  | Pyrophosphatase, inorganic |
|  | PPA1 |
|  | Pyrophosphate phospho- hydrolase |
|  | PP |
|  | pyrophosphate phospho-hydrolase |
|  | inorganic pyrophosphatase |
|  | cytosolic inorganic pyrophosphatase |
|  | SID6-8061 |
|  | Pyrophosphatase inorganic |
|  | PPase |


---

|  |  |
| --- | --- |
| GO Component | membrane |
|  | cytoplasm |


---

|  |  |
| --- | --- |
| GO ID | GO:0000287 |
|  | GO:0016020 |
|  | GO:0008152 |
|  | GO:0016462 |
|  | GO:0004427 |
|  | GO:0005737 |
|  | GO:0016787 |
|  | GO:0008306 |
|  | GO:0006796 |


---

|  |  |
| --- | --- |
| MIM | MIM:179030 |


---

|  |  |
| --- | --- |
| Connectivity | 784 |


---

|  |  |
| --- | --- |
| Entrez ID | 294504 |
|  | 104145 |
|  | 5464 |


---

|  |  |
| --- | --- |
| Agilent ID | A\_23\_P161338 |
|  | A\_14\_P118565 |
|  | A\_42\_P548520 |
|  | A\_23\_P161336 |


---

|  |  |
| --- | --- |
| Cellular Localization | Membrane |
|  | Cytoplasm |
|  | Cell |


---

|  |  |
| --- | --- |
| DbXref | KEGG pathway##00190##Oxidative phosphorylation##http://www.genome.jp/dbget-bin/show\_pathway?hsa00190+5464 |
|  | Reactome##71732##pyrophosphate + H2O => 2 orthophosphate##http://www.reactome.org/cgi-bin/eventbrowser?DB=gk\_current&ID=71732 |


---

|  |  |
| --- | --- |
| Pathway | Long-term Potentiation |
|  | Zn def RIN |
|  | Master Regulators |
|  | Zn def DIN |


---

|  |  |
| --- | --- |
| GO Process | metabolism |
|  | associative learning |
|  | phosphate metabolism |


---

|  |  |
| --- | --- |
| UniGene | Rn.106916 |
|  | Hs.437403 |


---

|  |  |
| --- | --- |
| Affymetrix Probeset ID | 217848\_s\_at |
|  | 59718\_at |
|  | 62391\_f\_at |
|  | 62392\_r\_at |
|  | 64333\_s\_at |
|  | g11056043\_3p\_a\_at |
|  | rc\_AA891311\_at |
|  | rc\_AA965166\_at |
|  | 1388960\_at |
|  | C00032\_at |
|  | RC\_F04258\_s\_at |
|  | Z48605\_at |


---

|  |  |
| --- | --- |
| EC Number | EC 3.6.1.1 |


---

|  |  |
| --- | --- |
| GO Function | hydrolase activity |
|  | inorganic diphosphatase activity |
|  | pyrophosphatase activity |
|  | magnesium ion binding |


---

|  |  |
| --- | --- |
| Nucleotide | AF119665 |
|  | NM\_021129 |
|  | AB026723 |
|  | BC001022 |
|  | BU520836 |
|  | AK058193 |
|  | BC061581 |
|  | XM\_215416 |
|  | AF154065 |
|  | BC099794 |
|  | AL731540 |
|  | AF108211 |
|  | AF217186 |
|  | AF092439 |


---

|  |  |
| --- | --- |
| Protein | XP\_215416 |
|  | NP\_066952 |
|  | Q15181 |
|  | BAA84702 |
|  | AAF17222 |
|  | AAD34643 |
|  | CAI13693 |
|  | AAH01022 |
|  | AAP97214 |
|  | AAD24964 |
|  | AAH99794 |
|  | AAH61581 |
|  | AAG36780 |
|  | CAI13692 |


---

|  |  |
| --- | --- |
| Organism | Mammal |


---

|  |  |
| --- | --- |
| Location | chromosome 20, 20q11 (Rattus norvegicus) |
|  | chromosome 10, 10q11.1-q24 (Homo sapiens) |


---

|  |  |
| --- | --- |
