# Supplementary material for: Dynamic transcriptomic profiles of zebrafish gills in response to zinc depletion
Source: BMC Genomics. 2010 Oct 8;11:548. doi: 10.1186/1471-2164-11-548 (PMC3091697; doi:10.1186/1471-2164-11-548)
Supplement: Additional file 2 — Figure S1 - Interactive Direct Interaction Network of responses to zinc depletion. Mini web-site containing index.html and hyperlinked pages in subdirectory. The web site is an interactive version of Figure 6A containing curated interactions between regulated genes and respective proteins. Legend: Molecular interactions between zinc and proteins encoded by genes changed under zinc depletion. A Direct Interaction Network was created based on curated interactions contained within the PathwayArchitect database and provided through hyperlinks. Red ovals represent proteins and the blue circle symbolizes Zn(II). Dark blue squares denote 'binding', and light blue squares 'expression'; green squares stand for 'regulation', green diamonds for 'metabolism', and green circles for 'promoter binding'. Arrow heads indicate directionality of the interaction where annotated. [file 1471-2164-11-548-S2.ZIP › PathwayArchitect Zn def DIN2/913906.html]

# BINDING:

|  |  |
| --- | --- |
| Type | BINDING |
| Effect | None |


---

|  |  |
| --- | --- |
| Score | 0 |


---

|  |  |
| --- | --- |
| Reference Count | 10 |


---

|  |  |
| --- | --- |
| Mechanism | Unknown |


---

|  |  |
| --- | --- |
| Reference:0 || Sentence | "Fluorescence emission from cardiac troponin C(F20W/N51C)(AEDANS) complexed to cardiac troponin I(1-80) was used to monitor binding of cardiac troponin I(129-166) to the regulatory domain of cardiac troponin C." |
| PMID | 11352734 |
| Year | 2001 |
| Journal | Biochemistry |
| RefScore | 1 |
| Source | PArchNLP |
  |
|  |  |


---

|  |  |
| --- | --- |
 Reference:1 || Sentence | "A similar pattern of chemical shift perturbations was observed for interaction of the troponin I cardiac-specific amino-terminus with the cardiac troponin C regulatory domain." |
| PMID | 11591369 |
| Year | 2001 |
| Journal | FEBS Lett |
| RefScore | 0 |
| Source | PArchNLP |
  ||  |  |


---

|  |  |
| --- | --- |
 Reference:2 || PMID | 12060657 |
| SourceID | 110072 |
| Species | Human |
| Experimental Condition | in-vitro |
| Description | The three dimensional solution structure of the Regulatory N-Domain of Human Cardiac Troponin C in Complex with Human Cardiac Troponin-I(147- 163) and Bepridil was determined by using NMR spectroscopy. Troponin C, Slow Skeletal and Cardiac Muscles, Troponin I, Cardiac Muscle was dissolved in a solution containing 1mM Cntnc, 3mM Ctni147-163, 1.5mM Bepridil, 5mM DTT, 10 KCl, 10mM Imdz, Trace Amoun of Nan3, 50um Dss, 90% H2O, 10% D2O, 0.1 pH 6.7 at 303K. The NMR experiments conducted were 3D\_15N-Separated\_NOESY, 3D\_ 15N-Separated\_dipsi, HCCH- TOCSY, 3D\_13C,15N-Separated NOESY, Cbcaconnh, Hncacb, 2 13C, 15N-Filtered NOESY, 2D 13C, 15N-Filtered Dipsi, 3D 13C, 15N-Filtered, Edited N using field strength(s) of 500 MHz, 600 MHz, 800 MHz. The structure was determined by Simulated Annealing. 30 structures were calculated and 30 structures were submitted. PDB: 1LXF, MMDB: 21426. |
| Detection Method | three-dimensional-structure |
| Source | BIND |
  ||  |  |


---

|  |  |
| --- | --- |
 Reference:3 || PMID | 9560191 |
| SourceID | 118900 |
| Experimental Condition | in-vitro |
| Description | The three dimensional structure of the 46kda Domain of Human Cardiac Troponin in the Ca2+ Saturated Form was determined by crystallography. The crystal was grown by hanging-drop and vapour diffusion using Troponin C, Troponin T, Troponin I, PEG3350, Lithium Chloride, Tris- HCl, Calcium Chloride, Glycerol pH 8.0, at 293K. X-ray data were collected using synchrotron radiation and were 96.7% complete to a resolution limit of 2.600 angstroms with an overall Rsym of 4.2%. This crystal contained 6 chains per asymmetric unit and belonged to the space group P 1 21 1. The structure was determined using MAD and the modeled structure was refined to an R-factor = 26.4% and had an R-free = 29.8%. PDB: 1J1D, MMDB: 24424. |
| Detection Method | three-dimensional-structure |
| Source | BIND |
  ||  |  |


---

|  |  |
| --- | --- |
 Reference:4 || PMID | 9560191 |
| SourceID | 118906 |
| Experimental Condition | in-vitro |
| Description | The three dimensional structure of the 52kda Domain of Human Cardiac Troponin in the Ca2+ Saturated Form was determined by crystallography. The crystal was grown by hanging-drop and vapour diffusion using Troponin C, Troponin T, Troponin I, PEG3350, Lithium Chloride, Tris- HCl, Calcium Chloride, Glycerol pH 8.0, at 293K. X-ray data were collected using synchrotron radiation and were 99.3% complete to a resolution limit of 3.300 angstroms with an overall Rsym of 5.2%. This crystal contained 6 chains per asymmetric unit and belonged to the space group P 1 21 1. The structure was determined using Molecular Replacement and the modeled structure was refined to an R-factor = 25.1% and had an R-free = 30.8%. PDB: 1J1E, MMDB: 24425. |
| Detection Method | three-dimensional-structure |
| Source | BIND |
  ||  |  |


---

|  |  |
| --- | --- |
 Reference:5 || PMID | 12732641 |
| SourceID | 119893 |
| Species | Human |
| Experimental Condition | in-vitro |
| Description | The three dimensional solution structure of C-Domain of Human Cardiac Troponin C in Complex with the Inhibitory Region of Human Cardiac Troponin I was determined by using NMR spectroscopy. Troponin C, Slow Skeletal and Cardiac Muscles, Troponin I, Cardiac Muscle was dissolved in a solution containing 13C15N-Cctnc in Complex Wi 13C15N-Cip, 0.1 pH 6.7 at 303K. The NMR experiments conducted were 3D\_13C-Separated\_NOESY, HNH 3D\_15N-Separated\_NOESY, 3D\_ -TOCSY, 3D\_15N-TOCSY using field strength(s) of 800 MHz, 500 MHz, 600 MHz. The structure was determined by 100 structures Calculated Using Cns Simulated Anneali Protocol Starting from Extended structures. 100 structures were calculated and 30 structures were submitted based on the structures with the Lowest Energy. PDB: 1OZS, MMDB: 24731. |
| Detection Method | three-dimensional-structure |
| Source | BIND |
  ||  |  |


---

|  |  |
| --- | --- |
 Reference:6 || Sentence | Structure of the Mg2+-loaded C-lobe of cardiac troponin C bound to the N-domain of cardiac troponin I: comparison with the Ca2+-loaded structure. |
| Year | 2004 |
| PMID | 15350124 |
| Species | Mouse |
| Journal | Biochemistry |
| RefScore | 0 |
| Source | PArchNLP |
  ||  |  |


---

|  |  |
| --- | --- |
 Reference:7 || Sentence | Thus, conformational plasticity in the Ca(2+)/Mg(2+)-dependent binding loops may represent a mechanism to modulate C-lobe cTnC interactions with the N domain of cTnI. |
| Year | 2004 |
| PMID | 15350124 |
| Species | Mouse |
| Journal | Biochemistry |
| RefScore | 1 |
| Source | PArchNLP |
  ||  |  |


---

|  |  |
| --- | --- |
 Reference:8 || Sentence | The results indicate that in the presence of cTnI, W7 no longer binds to multiple sites of cTnC but instead binds specifically to the N-domain, and the binding (K(D) = 0.5 +/- 0.1 mM) can occur together with the switch region of cTnI. |
| PMID | 16893183 |
| Year | 2006 |
| Species | Human |
| Journal | Biochemistry |
| RefScore | 1 |
| Source | PArchNLP |
  ||  |  |


---

|  |  |
| --- | --- |
 Reference:9 || Sentence | Hence, W7 may play a role in directly modulating the Ca(2+) sensitivity of the regulatory domain of cTnC and the interaction of the switch region of cTnI and cTnC. |
| PMID | 16893183 |
| Year | 2006 |
| Species | Human |
| Journal | Biochemistry |
| RefScore | 2 |
| Source | PArchNLP |
  |


---

|  |  |
| --- | --- |
