# Supplementary material for: Dynamic transcriptomic profiles of zebrafish gills in response to zinc depletion
Source: BMC Genomics. 2010 Oct 8;11:548. doi: 10.1186/1471-2164-11-548 (PMC3091697; doi:10.1186/1471-2164-11-548)
Supplement: Additional file 2 — Figure S1 - Interactive Direct Interaction Network of responses to zinc depletion. Mini web-site containing index.html and hyperlinked pages in subdirectory. The web site is an interactive version of Figure 6A containing curated interactions between regulated genes and respective proteins. Legend: Molecular interactions between zinc and proteins encoded by genes changed under zinc depletion. A Direct Interaction Network was created based on curated interactions contained within the PathwayArchitect database and provided through hyperlinks. Red ovals represent proteins and the blue circle symbolizes Zn(II). Dark blue squares denote 'binding', and light blue squares 'expression'; green squares stand for 'regulation', green diamonds for 'metabolism', and green circles for 'promoter binding'. Arrow heads indicate directionality of the interaction where annotated. [file 1471-2164-11-548-S2.ZIP › PathwayArchitect Zn def DIN2/924939.html]

# REGULATION:

|  |  |
| --- | --- |
| Type | REGULATION |
| Effect | Negative |


---

|  |  |
| --- | --- |
| Score | 0 |


---

|  |  |
| --- | --- |
| Reference Count | 5 |


---

|  |  |
| --- | --- |
| Mechanism | Unknown |


---

|  |  |
| --- | --- |
| Reference:0 || Sentence | "The down-regulation of beta-catenin-TCF signaling was independent of APC, which we characterized as having a nonsense mutation in codon 1367 in Caco-2 cells, but was associated with a decrease in TCF-4 protein levels." |
| PMID | 11309309 |
| Year | 2001 |
| Species | Human |
| Journal | Cancer Res |
| RefScore | 2 |
| Source | PArchNLP |
  |
|  |  |


---

|  |  |
| --- | --- |
 Reference:1 || Sentence | "Importantly, down-regulation of beta-catenin-TCF signaling in undifferentiated Caco-2 cells by three different mechanisms, ectopic expression of E-cadherin, wild-type APC, or dominant negative TCF-4, resulted in an increase in the promoter activities of two genes that are well-established markers of cell differentiation, alkaline phosphatase and intestinal fatty acid binding protein." |
| PMID | 11309309 |
| Year | 2001 |
| Species | Human |
| Journal | Cancer Res |
| RefScore | 0 |
| Source | PArchNLP |
  ||  |  |


---

|  |  |
| --- | --- |
 Reference:2 || Sentence | "Thus, expression of dominant-negative Tcf4 blocks EGF-mediated Tcf/Lef transcriptional activity and bromodeoxyuridine uptake." |
| PMID | 15026412 |
| Year | 2004 |
| Species | Human |
| Journal | J Biol Chem |
| RefScore | 1 |
| Source | PArchNLP |
  ||  |  |


---

|  |  |
| --- | --- |
 Reference:3 || Sentence | Thus, expression of dominant-negative Tcf4 blocks EGF-mediated Tcf/Lef transcriptional activity and bromodeoxyuridine uptake. |
| Year | 2004 |
| PMID | 15026412 |
| Species | Human |
| Journal | J Biol Chem |
| RefScore | 1 |
| Source | PArchNLP |
  ||  |  |


---

|  |  |
| --- | --- |
 Reference:4 || Sentence | Inhibition of beta-catenin/Tcf signaling by ectopic expression of dominant negative Tcf4 resulted in significant inhibition of growth in squamous cell carcinoma cells. |
| PMID | 15765534 |
| Year | 2005 |
| Species | Mouse |
| Journal | Mol Carcinog |
| RefScore | 1 |
| Source | PArchNLP |
  |


---

|  |  |
| --- | --- |
