# Supplementary material for: Dynamic transcriptomic profiles of zebrafish gills in response to zinc depletion
Source: BMC Genomics. 2010 Oct 8;11:548. doi: 10.1186/1471-2164-11-548 (PMC3091697; doi:10.1186/1471-2164-11-548)
Supplement: Additional file 2 — Figure S1 - Interactive Direct Interaction Network of responses to zinc depletion. Mini web-site containing index.html and hyperlinked pages in subdirectory. The web site is an interactive version of Figure 6A containing curated interactions between regulated genes and respective proteins. Legend: Molecular interactions between zinc and proteins encoded by genes changed under zinc depletion. A Direct Interaction Network was created based on curated interactions contained within the PathwayArchitect database and provided through hyperlinks. Red ovals represent proteins and the blue circle symbolizes Zn(II). Dark blue squares denote 'binding', and light blue squares 'expression'; green squares stand for 'regulation', green diamonds for 'metabolism', and green circles for 'promoter binding'. Arrow heads indicate directionality of the interaction where annotated. [file 1471-2164-11-548-S2.ZIP › PathwayArchitect Zn def DIN2/95690.html]

# PROTEIN: PDLIM5

|  |  |
| --- | --- |
| Name | PDLIM5 |
| Type | PROTEIN |
| Description | PDZ and LIM domain 5 |
| Note | The protein encoded by this gene is a LIM domain protein. LIM domains are cysteine-rich double zinc fingers composed of 50 to 60 amino acids that are involved in protein-protein interactions. LIM domain-containing proteins are scaffolds for the formation of multiprotein complexes. The proteins are involved in cytoskeleton organization, cell lineage specification, organ development, and oncogenesis. The encoded protein is also a member of the Enigma class of proteins, a family of proteins that possess a 100-amino acid PDZ domain in the N terminus and 1 to 3 LIM domains in the C terminus. Multiple transcript variants encoding different isoforms have been found for this gene, although not all of them have been fully characterized. |
| Alias | AI987914 |
|  | Enh2 |
|  | Enh3 |
|  | 1110001A05Rik |
|  | LIM |
|  | ENH |
|  | Enh1 |
|  | enigma-like LIM domain protein |
|  | Enigma-like PDZ and LIM domains protein |
|  | Enh |
|  | L9 |
|  | enigma homolog |
|  | C87059 |


---

|  |  |
| --- | --- |
| GO Component | Z disc |
|  | cytosol |
|  | membrane fraction |


---

|  |  |
| --- | --- |
| GO ID | GO:0007507 |
|  | GO:0000004 |
|  | GO:0042805 |
|  | GO:0003779 |
|  | GO:0030159 |
|  | GO:0046872 |
|  | GO:0005080 |
|  | GO:0030018 |
|  | GO:0005515 |
|  | GO:0005829 |
|  | GO:0008270 |
|  | GO:0005624 |


---

|  |  |
| --- | --- |
| MIM | MIM:605904 |


---

|  |  |
| --- | --- |
| Connectivity | 147 |


---

|  |  |
| --- | --- |
| Entrez ID | 10611 |
|  | 56376 |


---

|  |  |
| --- | --- |
| Agilent ID | A\_14\_P122181 |
|  | A\_14\_P101352 |
|  | A\_53\_P169124 |
|  | A\_14\_P121824 |
|  | A\_14\_P125063 |
|  | A\_24\_P24263 |
|  | A\_53\_P125381 |
|  | A\_52\_P923158 |
|  | A\_53\_P101263 |
|  | A\_32\_P14610 |
|  | A\_51\_P408881 |
|  | A\_52\_P649177 |
|  | A\_23\_P170761 |
|  | A\_52\_P339246 |
|  | A\_52\_P662600 |
|  | A\_52\_P149438 |
|  | A\_14\_P136807 |
|  | A\_24\_P174641 |


---

|  |  |
| --- | --- |
| Cellular Localization | Cytosol |
|  | Cytoplasm |
|  | Cell |


---

|  |  |
| --- | --- |
| Pathway | Master Regulators |
|  | Zn def RIN |
|  | Zn def DIN |


---

|  |  |
| --- | --- |
| GO Process | biological process unknown |
|  | heart development |


---

|  |  |
| --- | --- |
| UniGene | Hs.480311 |
|  | Mm.117709 |


---

|  |  |
| --- | --- |
| Affymetrix Probeset ID | 106642\_at |
|  | 1421413\_a\_at |
|  | 1422861\_s\_at |
|  | 1429783\_at |
|  | 1438186\_at |
|  | 1442710\_at |
|  | 1446339\_at |
|  | 1450786\_x\_at |
|  | 1563487\_at |
|  | 163704\_at |
|  | 203242\_s\_at |
|  | 203243\_3p\_s\_at |
|  | 203243\_s\_at |
|  | 211681\_s\_at |
|  | 212412\_at |
|  | 213684\_s\_at |
|  | 216803\_at |
|  | 216804\_s\_at |
|  | 221994\_at |
|  | 241208\_at |
|  | 37366\_at |
|  | 40058\_s\_at |
|  | 40059\_r\_at |
|  | 40060\_r\_at |
|  | 47108\_at |
|  | 53417\_at |
|  | 72076\_s\_at |
|  | 72079\_r\_at |
|  | 72189\_at |
|  | 80698\_at |
|  | AFFX-hum\_alu\_at |
|  | g5453713\_3p\_x\_at |
|  | g7959908\_RC\_3p\_s\_at |
|  | Hs.154103.0.S1\_3p\_s\_at |
|  | Hs.154103.1.S1\_3p\_a\_at |
|  | Hs.154103.2.S1\_3p\_at |
|  | Hs2.376881.1.S1\_3p\_at |
|  | Hs.254458.0.A1\_3p\_at |
|  | Hs.7780.0.S1\_3p\_at |
|  | hum\_alu\_at |
|  | 1422862\_at |
|  | 1422863\_s\_at |
|  | 107972\_at |
|  | 59607\_f\_at |
|  | 63729\_f\_at |
|  | 75847\_r\_at |
|  | AA045870\_at |
|  | R09286\_at |
|  | RC\_AA056482\_at |
|  | RC\_AA253314\_at |
|  | RC\_AA430209\_at |
|  | RC\_AA447977\_s\_at |
|  | RC\_F01813\_s\_at |
|  | RC\_N52271\_at |
|  | RC\_R09181\_at |
|  | TC34300\_s\_at |
|  | TC40860\_at |


---

|  |  |
| --- | --- |
| GO Function | protein binding |
|  | protein kinase C binding |
|  | actin binding |
|  | receptor signaling complex scaffold activity |
|  | actinin binding |
|  | zinc ion binding |
|  | metal ion binding |


---

|  |  |
| --- | --- |
| Nucleotide | AL833151 |
|  | AB016588 |
|  | AL832686 |
|  | BC008741 |
|  | BC037476 |
|  | NM\_022554 |
|  | BC026029 |
|  | NM\_019809 |
|  | AC093778 |
|  | BT007328 |
|  | AY598328 |
|  | AB016586 |
|  | NM\_001011515 |
|  | BC017902 |
|  | NM\_001011516 |
|  | AL833438 |
|  | NM\_001011514 |
|  | AK003194 |
|  | AK009464 |
|  | AB016587 |
|  | NM\_019808 |
|  | AK089779 |
|  | NM\_006457 |
|  | AK147999 |
|  | AK027217 |
|  | AL833439 |
|  | AL832157 |
|  | AL049969 |
|  | AK129886 |
|  | NM\_001011513 |
|  | BC020145 |
|  | AF061258 |


---

|  |  |
| --- | --- |
| Protein | Q8CI51 |
|  | AAH37476 |
|  | AAP35992 |
|  | NP\_062783 |
|  | AAC15767 |
|  | NP\_001011516 |
|  | NP\_072048 |
|  | BAA88828 |
|  | AAH20145 |
|  | AAT06739 |
|  | AAH17902 |
|  | AAH08741 |
|  | NP\_006448 |
|  | NP\_001011513 |
|  | AAY40898 |
|  | BAB26304 |
|  | NP\_062782 |
|  | BAE28279 |
|  | NP\_001011514 |
|  | NP\_001011515 |
|  | BAB22633 |
|  | BAA88827 |
|  | BAA88829 |
|  | Q96HC4 |


---

|  |  |
| --- | --- |
| Organism | Mammal |


---

|  |  |
| --- | --- |
| Location | chromosome 3, 3 H3 (Mus musculus) |
|  | chromosome 4, 4q22 (Homo sapiens) |


---

|  |  |
| --- | --- |
