# Supplementary material for: Dynamic transcriptomic profiles of zebrafish gills in response to zinc depletion
Source: BMC Genomics. 2010 Oct 8;11:548. doi: 10.1186/1471-2164-11-548 (PMC3091697; doi:10.1186/1471-2164-11-548)
Supplement: Additional file 2 — Figure S1 - Interactive Direct Interaction Network of responses to zinc depletion. Mini web-site containing index.html and hyperlinked pages in subdirectory. The web site is an interactive version of Figure 6A containing curated interactions between regulated genes and respective proteins. Legend: Molecular interactions between zinc and proteins encoded by genes changed under zinc depletion. A Direct Interaction Network was created based on curated interactions contained within the PathwayArchitect database and provided through hyperlinks. Red ovals represent proteins and the blue circle symbolizes Zn(II). Dark blue squares denote 'binding', and light blue squares 'expression'; green squares stand for 'regulation', green diamonds for 'metabolism', and green circles for 'promoter binding'. Arrow heads indicate directionality of the interaction where annotated. [file 1471-2164-11-548-S2.ZIP › PathwayArchitect Zn def DIN2/958611.html]

# REGULATION:

|  |  |
| --- | --- |
| Type | REGULATION |
| Effect | None |


---

|  |  |
| --- | --- |
| Score | 0 |


---

|  |  |
| --- | --- |
| Reference Count | 3 |


---

|  |  |
| --- | --- |
| Mechanism | Unknown |


---

|  |  |
| --- | --- |
| Reference:0 || Sentence | "STUDY OBJECTIVE: To investigate whether there is an association between Troponin T (TnT), reflecting myocardial cell injury, and cortisol, reflecting the degree of surgical trauma and associated stress, in light of our recent evaluation of TnT as a marker of perioperative myocardial cell injury.DESIGN: Prospective, cohort study.PATIENTS: 70 patients (67.4 +/- 8.7 yrs) with definite or at-risk coronary artery disease (CAD) undergoing elective noncardiac surgery (vascular n = 38, abdominal n = 21, orthopedic n = 8) with general (n = 63) or regional (n = 4) anesthesia with postoperative on-demand analgesia.MEASUREMENTS AND MAIN RESULTS: Morning blood samples for TnT (upper limit of normal: <0.2 ng/mL), CK-MB (reference range |
| PMID | 10869919 |
| Year | 2000 |
| Species | Human |
| Journal | J Clin Anesth |
| RefScore | 1 |
| Source | PArchNLP |
  |
|  |  |


---

|  |  |
| --- | --- |
 Reference:1 || Sentence | "The association of different cutoff levels of CK-MB, troponin, and ischemia duration with long-term survival was investigated." |
| PMID | 14607436 |
| Year | 2003 |
| Species | Human |
| Journal | J Am Coll Cardiol |
| RefScore | 1 |
| Source | PArchNLP |
  ||  |  |


---

|  |  |
| --- | --- |
 Reference:2 || Sentence | CK-MB and TnT had higher specificities (73-100%) but significantly lower positive rates (22-27%) than the others (61-68%), resulting in greater sensitivities of H-FABP and myoglobin (75-80%) than those of CK-MB and TnT (29-35%). |
| PMID | 16565558 |
| Year | 2006 |
| Species | Human |
| Journal | Circ J |
| RefScore | 2 |
| Source | PArchNLP |
  |


---

|  |  |
| --- | --- |
