# Supplementary material for: Dynamic transcriptomic profiles of zebrafish gills in response to zinc depletion
Source: BMC Genomics. 2010 Oct 8;11:548. doi: 10.1186/1471-2164-11-548 (PMC3091697; doi:10.1186/1471-2164-11-548)
Supplement: Additional file 2 — Figure S1 - Interactive Direct Interaction Network of responses to zinc depletion. Mini web-site containing index.html and hyperlinked pages in subdirectory. The web site is an interactive version of Figure 6A containing curated interactions between regulated genes and respective proteins. Legend: Molecular interactions between zinc and proteins encoded by genes changed under zinc depletion. A Direct Interaction Network was created based on curated interactions contained within the PathwayArchitect database and provided through hyperlinks. Red ovals represent proteins and the blue circle symbolizes Zn(II). Dark blue squares denote 'binding', and light blue squares 'expression'; green squares stand for 'regulation', green diamonds for 'metabolism', and green circles for 'promoter binding'. Arrow heads indicate directionality of the interaction where annotated. [file 1471-2164-11-548-S2.ZIP › PathwayArchitect Zn def DIN2/97579.html]

# PROTEIN: MRPS28

|  |  |
| --- | --- |
| Name | MRPS28 |
| Type | PROTEIN |
| Description | mitochondrial ribosomal protein S28 |
| Note | Mammalian mitochondrial ribosomal proteins are encoded by nuclear genes and help in protein synthesis within the mitochondrion. Mitochondrial ribosomes (mitoribosomes) consist of a small 28S subunit and a large 39S subunit. They have an estimated 75% protein to rRNA composition compared to prokaryotic ribosomes, where this ratio is reversed. Another difference between mammalian mitoribosomes and prokaryotic ribosomes is that the latter contain a 5S rRNA. Among different species, the proteins comprising the mitoribosome differ greatly in sequence, and sometimes in biochemical properties, which prevents easy recognition by sequence homology. This gene encodes a 28S subunit protein that has been called mitochondrial ribosomal protein S35 in the literature. |
| Alias | 1500012D08Rik |
|  | MRP-S28 |
|  | FLJ22853 |
|  | HSPC007 |
|  | S28mt |
|  | mitochondrial 28S ribosomal protein S35 |
|  | MRP-S35 |
|  | MRPS35;ORFNames=HSPC007 |
|  | MRPS28 |
|  | MRPS35 |
|  | AW061224 |


---

|  |  |
| --- | --- |
| GO Component | mitochondrion |
|  | ribonucleoprotein complex |
|  | mitochondrial small ribosomal subunit |


---

|  |  |
| --- | --- |
| GO ID | GO:0003723 |
|  | GO:0005554 |
|  | GO:0030529 |
|  | GO:0005763 |
|  | GO:0003735 |
|  | GO:0005739 |
|  | GO:0000004 |


---

|  |  |
| --- | --- |
| Connectivity | 1 |


---

|  |  |
| --- | --- |
| Entrez ID | 28957 |
|  | 66230 |


---

|  |  |
| --- | --- |
| Agilent ID | A\_14\_P114329 |
|  | A\_53\_P127704 |
|  | A\_23\_P169050 |
|  | A\_14\_P115759 |
|  | A\_53\_P148464 |
|  | A\_51\_P282394 |
|  | A\_53\_P112840 |


---

|  |  |
| --- | --- |
| Cellular Localization | Mitochondrion |
|  | Cytoplasm |
|  | Cell |
|  | Ribosome |
|  | Organelle |


---

|  |  |
| --- | --- |
| Pathway | Zn def RIN |
|  | Master Regulators |
|  | Zn def DIN |


---

|  |  |
| --- | --- |
| GO Process | biological process unknown |


---

|  |  |
| --- | --- |
| UniGene | Mm.38460 |
|  | Hs.521124 |


---

|  |  |
| --- | --- |
| Affymetrix Probeset ID | 1452585\_at |
|  | 164442\_f\_at |
|  | 219819\_s\_at |
|  | 236955\_at |
|  | 237854\_at |
|  | 43095\_at |
|  | 53106\_at |
|  | 75369\_at |
|  | 79653\_at |
|  | 82649\_at |
|  | 86423\_at |
|  | 86452\_r\_at |
|  | 98610\_at |
|  | g7661729\_3p\_a\_at |
|  | Hs.184387.0.A1\_3p\_at |
|  | Hs.196561.0.A1\_3p\_at |
|  | 244242\_at |
|  | Hs.244358.0.A1\_3p\_at |
|  | RC\_AA425543\_at |
|  | RC\_W32415\_at |
|  | TC40252\_at |


---

|  |  |
| --- | --- |
| GO Function | structural constituent of ribosome |
|  | RNA binding |
|  | molecular function unknown |


---

|  |  |
| --- | --- |
| Nucleotide | AK011036 |
|  | NM\_025434 |
|  | BC010150 |
|  | AF070663 |
|  | AK005229 |
|  | AB061209 |
|  | NM\_014018 |
|  | AK026506 |
|  | AK088314 |
|  | AK223069 |
|  | BC028530 |


---

|  |  |
| --- | --- |
| Protein | Q9Y2Q9 |
|  | AAH28530 |
|  | BAB54959 |
|  | NP\_079710 |
|  | BAD96789 |
|  | BAB27349 |
|  | AAD20969 |
|  | NP\_054737 |
|  | Q9CY16 |
|  | AAH10150 |


---

|  |  |
| --- | --- |
| Organism | Mammal |


---

|  |  |
| --- | --- |
| Location | chromosome 3, 3 A1 (Mus musculus) |
|  | chromosome 8, 8q21.1-q21.2 (Homo sapiens) |


---

|  |  |
| --- | --- |
