# Supplementary material for: Dynamic transcriptomic profiles of zebrafish gills in response to zinc depletion
Source: BMC Genomics. 2010 Oct 8;11:548. doi: 10.1186/1471-2164-11-548 (PMC3091697; doi:10.1186/1471-2164-11-548)
Supplement: Additional file 2 — Figure S1 - Interactive Direct Interaction Network of responses to zinc depletion. Mini web-site containing index.html and hyperlinked pages in subdirectory. The web site is an interactive version of Figure 6A containing curated interactions between regulated genes and respective proteins. Legend: Molecular interactions between zinc and proteins encoded by genes changed under zinc depletion. A Direct Interaction Network was created based on curated interactions contained within the PathwayArchitect database and provided through hyperlinks. Red ovals represent proteins and the blue circle symbolizes Zn(II). Dark blue squares denote 'binding', and light blue squares 'expression'; green squares stand for 'regulation', green diamonds for 'metabolism', and green circles for 'promoter binding'. Arrow heads indicate directionality of the interaction where annotated. [file 1471-2164-11-548-S2.ZIP › PathwayArchitect Zn def DIN2/99982.html]

# PROTEIN: MFI2

|  |  |
| --- | --- |
| Name | MFI2 |
| Type | PROTEIN |
| Description | antigen p97 (melanoma associated) identified by monoclonal antibodies 133.2 and 96.5 |
| Note | The protein encoded by this gene is a cell-surface glycoprotein found on melanoma cells. The protein shares sequence similarity and iron-binding properties with members of the transferrin superfamily. The importance of the iron binding function has not yet been identified. This gene resides in the same region of chromosome 3 as members of the transferrin superfamily. Alternative splicing results in two transcript variants. |
| Alias | MFI2 |
|  | CD228 |
|  | melanotransferrin |
|  | MTF1 |
|  | Mtf |
|  | Mfi2 |
|  | Melanoma-associated antigen p97 |
|  | MTf |
|  | Membrane-bound transferrin-like protein p97 |
|  | melanoma-associated antigen p97 |
|  | MGC4856 |
|  | MAP97 |
|  | CD228 antigen |


---

|  |  |
| --- | --- |
| GO Component | extracellular space |
|  | extracellular region |
|  | integral to plasma membrane |
|  | membrane |


---

|  |  |
| --- | --- |
| GO ID | GO:0006879 |
|  | GO:0006810 |
|  | GO:0016020 |
|  | GO:0005615 |
|  | GO:0005506 |
|  | GO:0008199 |
|  | GO:0046872 |
|  | GO:0005887 |
|  | GO:0006826 |
|  | GO:0005576 |


---

|  |  |
| --- | --- |
| MIM | MIM:155750 |


---

|  |  |
| --- | --- |
| Connectivity | 36 |


---

|  |  |
| --- | --- |
| Entrez ID | 30060 |
|  | 4241 |


---

|  |  |
| --- | --- |
| Agilent ID | A\_23\_P212042 |
|  | A\_53\_P108278 |
|  | A\_51\_P324351 |
|  | A\_23\_P386320 |
|  | A\_14\_P102948 |
|  | A\_53\_P136221 |


---

|  |  |
| --- | --- |
| Cellular Localization | Membrane |
|  | Extracellular region |
|  | Cell |
|  | Plasma membrane |


---

|  |  |
| --- | --- |
| Pathway | Zn def RIN |
|  | Zn xs inventory |
|  | Zn def DIN |
|  | Zn xs DIN |
|  | Zn xs RIN |


---

|  |  |
| --- | --- |
| GO Process | transport |
|  | iron ion transport |
|  | iron ion homeostasis |


---

|  |  |
| --- | --- |
| UniGene | Hs.184727 |
|  | Mm.30988 |


---

|  |  |
| --- | --- |
| Affymetrix Probeset ID | 1556538\_at |
|  | 220043\_s\_at |
|  | 223723\_at |
|  | 1419755\_at |
|  | 83377\_at |
|  | aa674893\_at |
|  | g12804858\_3p\_at |
|  | g5729923\_3p\_s\_at |
|  | Hs2.348653.1.S1\_3p\_at |
|  | Msa.25882.0\_s\_at |
|  | 235911\_at |
|  | 239521\_at |
|  | 242087\_x\_at |
|  | 243629\_x\_at |
|  | 76563\_at |
|  | 88946\_at |
|  | Hs.128852.0.A1\_3p\_a\_at |
|  | Hs.128852.0.A1\_3p\_at |
|  | Hs.128852.1.A1\_3p\_x\_at |
|  | Hs.128852.2.A1\_3p\_x\_at |
|  | 85279\_at |
|  | 97970\_at |
|  | Hs.184727.0.A1\_3p\_at |
|  | Msa.6814.0\_s\_at |
|  | TC18213\_at |


---

|  |  |
| --- | --- |
| GO Function | ferric iron binding |
|  | iron ion binding |
|  | metal ion binding |


---

|  |  |
| --- | --- |
| Nucleotide | AB024336 |
|  | BC002623 |
|  | M12154 |
|  | AK036444 |
|  | BC040347 |
|  | BC007550 |
|  | AB047799 |
|  | BC093920 |
|  | A00127 |
|  | NM\_005929 |
|  | BC071910 |
|  | BC001875 |
|  | BC053510 |
|  | NM\_033316 |
|  | NM\_013900 |
|  | BC032752 |
|  | BT007377 |


---

|  |  |
| --- | --- |
| Protein | NP\_005920 |
|  | NP\_201573 |
|  | AAP36041 |
|  | P08582 |
|  | CAA00012 |
|  | AAH07550 |
|  | BAB41139 |
|  | BAA86655 |
|  | AAH02623 |
|  | AAH71910 |
|  | AAA59992 |
|  | AAH40347 |
|  | NP\_038928 |
|  | AAH93920 |
|  | Q9R0R1 |
|  | AAH01875 |
|  | BAC29432 |


---

|  |  |
| --- | --- |
| Organism | Mammal |


---

|  |  |
| --- | --- |
| Location | chromosome 3, 3q28-q29 (Homo sapiens) |
|  | chromosome 16, 16 B3 (Mus musculus) |


---

|  |  |
| --- | --- |
